# Short, Divergent, and Enantioselective Total Synthesis of Bioactive *ent*-Pimaranes

**DOI:** 10.1021/acs.orglett.2c02843

**Published:** 2022-09-28

**Authors:** Immanuel Plangger, Klaus Wurst, Thomas Magauer

**Affiliations:** † Institute of Organic Chemistry and Center for Molecular Biosciences, Leopold-Franzens-University Innsbruck, Innrain 80−82, 6020 Innsbruck, Austria; ‡ Institute of General, Inorganic and Theoretical Chemistry, Leopold-Franzens-University Innsbruck, Innrain 80−82, 6020 Innsbruck, Austria

## Abstract

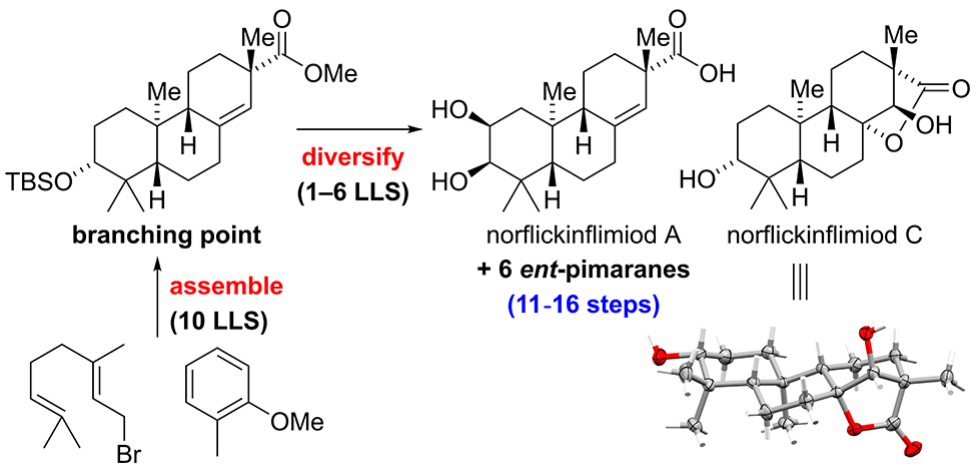

We present the first total synthesis of eight *ent*-pimaranes via a short and enantioselective route (11–16 steps). Key features of the divergent synthesis are a Sharpless asymmetric dihydroxylation, a Brønsted acid catalyzed cationic bicyclization, and a mild Rh-catalyzed arene hydrogenation for rapid access to a late synthetic branching point. From there on, selective functional group manipulations enable the synthesis of *ent*-pimaranes bearing different modifications in the A- and C-rings.

Pimarane natural products represent a large class of diterpenoids sharing a common 6,6,6-carbocyclic scaffold and exhibit diverse bioactivities including anti-inflammatory and anticancer properties (e.g., natural products **1**–**5**, Scheme 1A).^1^ To date, few total syntheses of pimaranes and the closely related isopimaranes (C13 epimer) have been reported, most of which rely either on condensation reactions (e.g., Robinson annulations) or on Diels–Alder cycloadditions to provide the requisite tricyclic architecture.^2^ In 1975, van Tamelen disclosed a hallmark synthesis of the isopimarane araucarol (**8**) involving a unique head-to-tail/tail-to-head polyene cyclization of racemic carbonate **6** (Scheme 1B).^3^ However, the reaction provided tricycle **7** as a mixture of double bond isomers in just 7% yield. To the best of our knowledge, there are only two other total syntheses of pimaranes—one of them by our group—which employ polyene cyclizations to selectively generate the underlying *trans*-decalin motif.^4^ As part of our continuing interest in developing cationic cyclization reactions, we sought to devise a scalable and concise synthetic entry point into the *ent*-pimarane natural product family. Within this study, we focused on previously inaccessible *ent*-pimaranes bearing diverse modifications in the A- and C-rings.

**Scheme 1:**
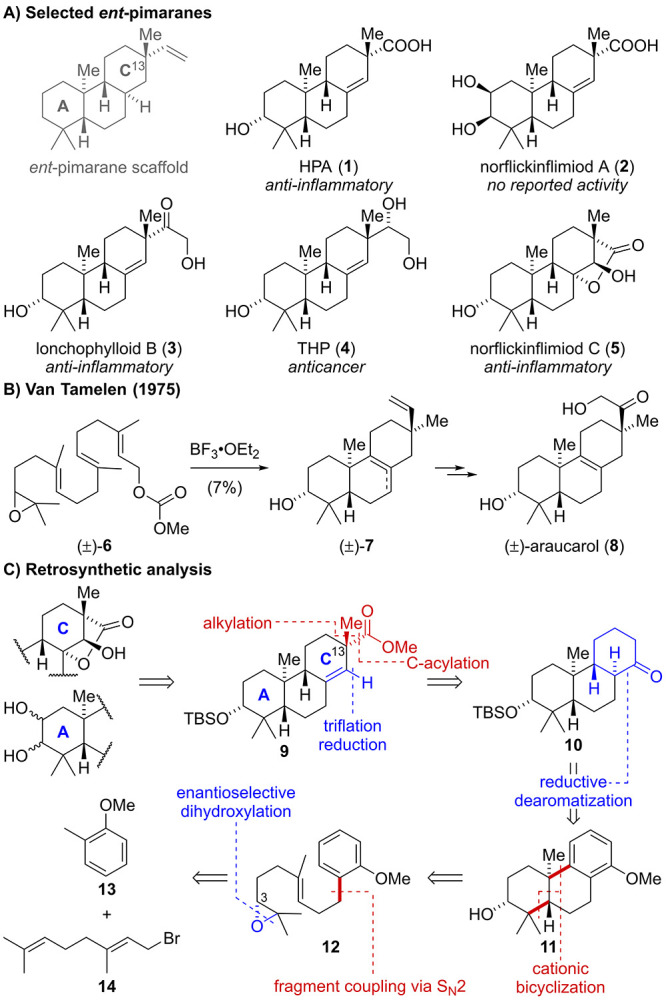
(A) Selected Structures of *ent*-Pimaranes, (B) Previous Work, and (C) Synthetic Strategy

From a structural perspective, the targeted *ent*-pimaranes feature five to seven stereocenters, two of which are quaternary, and further differ by the oxidation pattern around the eastern and western periphery, rendering adiversity-oriented total synthesis approach highly attractive (Scheme 1C). Retrosynthetically, we envisioned generation of the A- and C-ring oxidation patterns in a few steps via selective functionalization of advanced key intermediate **9**. For the installation of the C13 quaternary center of **9**, we identified a substrate-controlled α-alkylation/acylation sequence as the most versatile and strategic bond disconnection. The resulting ketone **10** was anticipated to be accessed through a reductive dearomatization of the structurally simplified tricyclic anisole **11**. Enantioselective construction of the requisite 6,6,6-carbocyclic scaffold **11** was envisioned in four steps from commercially available geranyl bromide (**14**) and 2-methyl anisole (**13**) involving Sharpless asymmetric dihydroxylation to set the stereochemistry at C3 and a cationic bicyclization of epoxide **12**.

Our synthesis commenced with a nucleophilic substitution reaction employing geranyl bromide (**14**) and the respective benzyl lithium species of 2-methyl anisole (**13**) to furnish geranyl arene **15** in 80% yield (Scheme 2A).^5^ The use of *sec*-butyllithium along with a slow warm-up from −78 to −20 °C was found to be essential for efficient benzylic lithiation. Subsequent Sharpless asymmetric dihydroxylation employing commercial ligands such as (DHQ)_2_PHAL and (DHQ)_2_AQN gave excellent enantioselectivities (91% *ee* for (DHQ)_2_PHAL and 93% *ee* for (DHQ)_2_AQN).^6^ However, those reactions suffered from poor regioselectivity and were also plagued by exhaustive dihydroxylation, resulting in low isolated yields for the desired diol **17** (20–25%, see the Supporting Information). Ultimately, we resorted to the use of the “*ent*”-Corey–Noe–Lin ligand (**16**), a diastereomer of the more established Corey–Noe–Lin ligand, which has been shown to exhibit high regioselectivities for sterically less encumbered alkenes.^7^ Gratifyingly, the use of **16** increased the yield of diol **17** to 65–67% yield while maintaining excellent enantioselectivity (93% *ee*). The overoxidation was minimized by discontinuing the reaction shortly before complete consumption of alkene **15**. Notably, **16** was recovered in 99% yield and was used for up to three cycles without any loss of regio- or enantioselectivity.

**Scheme 2:**
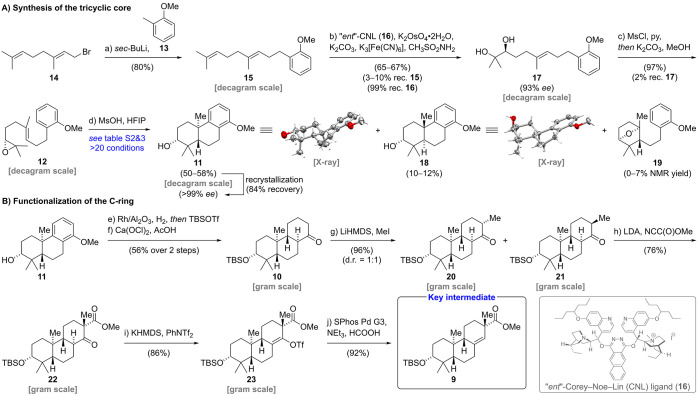
Enantioselective Synthesis of Key Intermediate **9** See the Supporting Information for detailed procedures and characterization data.

With diol **17** in hand, a selective one-pot mono-mesylation of the more accessible secondary alcohol followed by an intramolecular nucleophilic substitution in the presence of potassium carbonate and methanol furnished epoxide **12** in excellent yield (97%).^8^ Our screening of the key bicyclization commenced with established literature conditions for similar systems employing a variety of Lewis acids (i.e., SnCl_4_, Et_2_AlCl, EtAlCl_2_, BF_3_·Et_2_O, Bi(OTf)_3_, InBr_3_, FeCl_3_).^4d,5a,9^ Surprisingly, under these conditions, tricycle **11**(10) was only obtained in low yields (0–36% NMR yield, see the Supporting Information) together with significant amounts of oxabicyclo[2.2.1]heptane **19** and a complex mixture of side products. At this point, conditions recently reported by Qu employing tetraphenylphosphonium tetrafluoroborate (Ph_4_PBF_4_) in combination with 1,1,1,3,3,3-hexafluoroisopropanol (HFIP) attracted our attention.^11^ Notably, the authors hypothesized that hydrofluoric acid, formed via the hydrolysis of Ph_4_PBF_4_, catalyzes the further conversion of oxabicyclo[2.2.1]heptanes such as **19** to the fully cyclized products. Unfortunately, applying these conditions to epoxide **12** only resulted in the formation of equimolar amounts of tricycle **11** and **19** (36–37% NMR yield). Based on this result, we set out to screen alternative Brønsted acids in 1,1,1,3,3,3-hexafluoroisopropanol (HFIP). Following careful optimization, methanesulfonic acid was found to efficiently catalyze the conversion of **12** to the desired bicyclization product **11** in 50–58% yield on a decagram scale. In addition, oxabicyclo[2.2.1]heptane **19** (0–7%) and tricycle **18** (10–12%) featuring an axially oriented secondary alcohol were isolated from this reaction. The relative stereochemistry of **11** and **18** was confirmed by single crystal X-ray analysis. After recrystallization from diethyl ether, tricycle **11** was obtained in enantiopure form (>99% *ee*). We then moved on to investigate reductive dearomatization of the C-ring (Scheme 2B). Initial attempts to employ a Birch reduction protocol using a huge excess of lithium (>600 equiv)^10a,12^ resulted in poor yields (<20%) and left us with considerable safety concerns due to the handling of liquid ammonia at −40 °C, close to its boiling point. Notably, Birch reductions of electron-rich anisoles requiring protonation at a site bearing alkyl substituents have been reported as exceptionally challenging.^13^ Unfortunately, established methodologies such as a modification by Wilds,^14^ an electroreduction method developed by Baran,^15^ as well as an ammonia-free Birch reduction by Koide^16^ failed to deliver the desired products in satisfactory yields. Therefore, we proceeded to investigate alternative reduction protocols. While hydrogenation of structurally related arenes typically requires harsh reaction conditions,^2a,10a,17^ we found that exposure of **11** to Rh on alumina under a hydrogen atmosphere (12 bar) in isopropanol (65 °C) allowed for the formation of the corresponding cyclohexane under relatively mild conditions.^18^ After removal of isopropanol under reduced pressure, the inseparable mixture of diastereomeric alcohols was directly protected using *tert*-butyldimethylsilyl trifluoromethanesulfonate (TBSOTf) in the presence of 2,6-lutidine. Several methods for selective methyl ether oxidation to the corresponding ketone **10** were examined (see the Supporting Information). Extensive investigations revealed a combination of calcium hypochlorite and acetic acid in acetone:water (9:1 v/v) as the ideal oxidation method to yield **10** in 72% NMR yield on a 24 μmol scale.^19^ Unexpectedly, large scale oxidation (18 mmol) suffered from stalling of the reaction after partial conversion. Therefore, unreacted starting material was recovered and resubjected to the reaction conditions. After three cycles, the ketone **10** was obtained in 56% yield over two steps. Deprotonation of **10** using lithium bis(trimethylsilyl)amide (LiHMDS) at cryogenic temperatures (−55 to −38 °C) followed by addition of methyl iodide afforded α-methylated epimers **20** and **21** as an inconsequential 1:1 diastereomeric mixture in excellent combined yield (96%). Interestingly, the use of tetrahydrofuran as solvent was essential, as diethyl ether led to undesired double methylation through enolate equilibration (see the Supporting Information). Next, C-acylation of **20** and **21** was investigated via regioselective deprotonation and subsequent trapping of the enolate with Mander’s reagent. In accordance with Mander’s findings, competitive O-acylation was completely suppressed through the use of diethyl ether instead of tetrahydrofuran and strictly avoiding coordinating agents such as *N*,*N*,*N*′,*N*′-tetramethyl ethylenediamine (TMEDA).^20^ Employing only a slight excess of Mander’s reagent and performing the acylation at −78 °C was found to be essential to prevent the emergence of side products via cyanohydrin formation. Under optimized conditions, we obtained the β-ketoester **22** in 76% yield.^21^

Formation of the potassium enolate of **22** through deprotonation with potassium bis(trimethylsilyl)amide (KHMDS) in tetrahydrofuran (0 °C, 100 min) followed by trapping with phenyl triflimide (PhNTf_2_) at −78 °C furnished triflate **23** in 86% yield. Subsequent reduction of **23** was best performed employing SPhos Pd G3 catalyst (5 mol %), formic acid, and triethylamine to provide the key intermediate **9** in 92% yield (10-step LLS).

With ample amounts of key intermediate **9** in hand, we proceeded to investigate the anticipated diversifications of the A- and C-rings (Scheme 3). With regard to the A-ring, we performed a silyl deprotection of **9** using aqueous hydrofluoric acid, directly followed by oxidation with Dess–Martin periodinane (DMP) to yield ketone **24** in 97% over two steps. For the conversion of **24** to the α-hydroxylated ketone **25**, we opted for a robust Rubottom oxidation protocol that allowed us to obtain **25** as a single diastereomer.^22^ Reduction of α-hydroxy ketone **25** with sodium borohydride provided *trans*-diol **26** as the main product (66%) along with *cis*-diol **28** (19%) and, unexpectedly, also *cis*-diol **27** (3%). We hypothesize that isomerization of **25** via its enediol tautomer and subsequent reduction of the regioisomeric α-hydroxy ketone explains the formation of *cis*-diol **27**. Ester hydrolysis of **26** and **28** with aqueous sodium hydroxide was high yielding (97%) for both substrates and afforded 2,3-dihydroxy-16-nor-*ent*-pimar-8(14)-en-15-oic acid (DHPA, **29**) (17 mg) and norflickinflimiod A (**2**) (5.6 mg).^23^

**Scheme 3:**
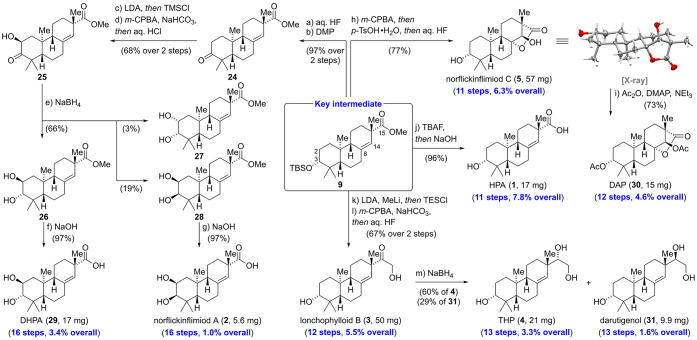
Divergent Synthesis of *ent*-Pimaranes through A- and C-Ring Modifications See the Supporting Information for detailed procedures and characterization data.

Having prepared natural products bearing modifications in the A-ring, we turned our attention toward diversification of the C-ring. According to the biosynthetic hypothesis,^1b^ the γ-lactone of norflickinflimiod C (**5**) is formed via a sequence that involves epoxidation of the C8/C14 alkene and intramolecular cyclization. In practice, exposure of **9** to *meta*-chloroperoxybenzoic acid (*m*-CPBA) followed by the addition of *para*-toluenesulfonic acid (*p*-TsOH) and desilylation using aqueous hydrofluoric acid directly afforded norflickinflimiod C (**5**) in 77% yield (57 mg). Single crystal X-ray analysis validated the depicted relative stereochemistry. Double acetylation with acetic anhydride and catalytic amounts of 4-(dimethylamino)pyridine (DMAP) gave 3,14-diacetoxy-16-nor-*ent*-pimar-15α,8-olide (DAP, **30**) in 73% yield (15 mg). Sequential desilylation with tetrabutylammonium fluoride (TBAF) and ester hydrolysis of **9** using sodium hydroxide furnished the 2-hydroxy-16-nor-*ent*-pimar-8(14)-en-15-oic acid (HPA, **1**) in excellent yield (96%, 17 mg). For the conversion of the ester at C15 into an α-hydroxy ketone, we turned to the Taber modification of the Fehr procedure.^24^ First, ester **9** was treated with lithium diisopropylamide (LDA) and methyl lithium and the resulting lithium enolate was trapped with triethylsilyl chloride (TESCl). The crude silyl enol ether was treated with *m*-CPBA at low temperatures (−30 °C) to prevent oxidation of the C8/C14 alkene. Excess *m*-CPBA was removed by addition of amylene, and silyl deprotection with aqueous hydrofluoric acid yielded lonchophylloid B (**3**) (50 mg). Reduction of lonchophylloid B (**3**) with sodium borohydride gave 3,15,16*-*trihydroxy-*ent*-pimar-8(14)-ene (THP, **4**) (60%, 21 mg) and darutigenol (**31**) (29%, 9.9 mg). The spectroscopic data for the eight synthetic natural products matched the literature reports; however, the sign of the optical rotation values for norflickinflimiod A (**2**) (α_D_^20^ = +65.1 vs α_D_^20^(literature)^1b^ = −48.4), norflickinflimiod C (**5**) (α_D_^20^ = +3.4 vs α_D_^20^(literature)^1b^ = −13.3), and lonchophylloid B (**3**) (α_D_^20^ = +6.2 vs α_D_^25^(literature)^1c^ = −9.93) was inverted. Validation of the absolute stereochemistry was finally possible by comparison of the ECD spectra with the literature and allowed us to confirm the configuration of all three natural products.

In summary, we have accomplished the first enantioselective total synthesis of eight *ent*-pimarane natural products in 11–16 steps (1.0–7.8% overall yield) from commercially available starting materials. The developed strategy enabled rapid access to diverse substitution patterns in the A-ring ((3*R*)-hydroxy, (2*S*,3*S*)-*trans*-diol, and (2*S*,3*R*)-*cis*-diol) and C-ring (γ-lactone, C15 carboxylic acid, α-hydroxy ketone, and C15/C16-diols). Salient features of our synthetic strategy encompass a scalable and robust four-step sequence allowing access to the tricyclic carbon scaffold through Sharpless asymmetric dihydroxylation in combination with a powerful Brønsted acid catalyzed bicyclization. A mild rhodium catalyzed arene hydrogenation served as an entry to the fully saturated 6,6,6-carbocyclic ring systems en route to a late synthetic branching point. Application of the key findings of this study may drive the development of scalable syntheses for other pimaranes and related diterpenoids and are currently underway in our laboratories.

## Supporting Information Available

Experimental details, spectroscopic data, and X-ray data (PDF)
