# Supplementary material for: Short, Divergent, and Enantioselective Total Synthesis of Bioactive ent-Pimaranes
Source: Org Lett. 2022 Sep 28;24(39):7151–6. doi: 10.1021/acs.orglett.2c02843 (PMC7613685; doi:10.1021/acs.orglett.2c02843)
Supplement: Supplementary file 1 — ol2c02843_si_001.pdf [file ol2c02843_si_001.pdf]

Supporting Information

Short, Divergent and Enantioselective Total Synthesis of Bioactive *ent*-Pimaranes

Immanuel Plangger[†], Klaus Wurst[§], and Thomas Magauer[†,*]

[†]Institute of Organic Chemistry and Center for Molecular Biosciences, Leopold-Franzens-University Innsbruck, Innrain 80–82, 6020 Innsbruck, Austria

[§]Institute of General, Inorganic and Theoretical Chemistry, Leopold-Franzens-University Innsbruck, Innrain 80–82, 6020 Innsbruck, Austria

*E-mail: thomas.magauer@uibk.ac.at

Content

1. General Experimental Details 4

2. Experimental Part 7

2.1 Synthetic intermediates 7

2.1.1 Geranyl bromide (**14**) 7

2.1.2 Geranyl arene **15** 7

2.1.3 Diol **17** 8

2.1.4 Epoxide **12** 11

2.1.5 Tricycle **11** 12

2.1.6 Ketone **10** 19

2.1.7 Ketone **20** and **21** 22

2.1.8 β-Ketoester **22** 24

2.1.9 Triflate **23** 25

2.1.10 Alkene **9** 27

2.1.11 Secondary alcohol **S3** 28

2.1.12 Ketone **24** 29

2.1.13 α-Hydroxy ketone **25** 30

2.1.14 Diols **26**, **27** and **28** 32

2.2 Synthesis of "*ent*"-Corey–Noe–Lin ligand (**16**) 35

2.2.1 2,3-dihydrobenzo[*g*]phthalazine-1,4-dione (**S5**) 35

2.2.2 1,4-dichlorobenzo[*g*]phthalazine (**S6**) 35

2.2.3 *O*6'-(4-Heptyl)hydrocupreine (**S8**) 36

2.2.4 Dimer **S10** 38

2.2.5 "*Ent*"-Corey–Noe–Lin ligand (**16**) 39

2.3 Racemic access to epoxide **12** 41

2.3.1 Epoxide ***rac*-12** 41

2.5 Syntheses of *ent*-pimarane natural products 43

2.5.1 (3*R*,5*S*,9*S*,10*S*,13*S*)-2-hydroxy-16-nor-*ent*-pimar-8(14)-en-15-oic acid (HPA, **1**) ... 43

2.5.2 Norflickinflimiod C (**5**) 47

2.5.3 3α,14β-diacetoxy-16-nor-*ent*-pimar-15α,8-olide (DAP, **30**) 51

2.5.4 Lonchophylloid B (**3**) 56

2.5.5 Darutigenol (**31**) and *ent*-3β,15*R*,16-trihydroxypimar-8(14)-ene (THP, **4**) 62

2.5.6 (2*S*,3*S*,5*S*,9*S*,10*S*,13*S*)-2,3-dihydroxy-16-nor-*ent*-pimar-8(14)-en-15-oic acid (DHPA, **29**) 73
2.5.7 Norflickinflimiod A (**2**) 77
2.6 Screenings 81
2.6.1 Screening of dihydroxylation to diol **17** 81
2.6.2 Screening of the cationic bicyclization to tricycle **11** 85
2.6.3 Screening of the methyl ether oxidation to ketone **10** 87
2.6.4 Screening of the α-methylation of ketone **10** 90
2.6.5 Screening of the C-acylation of ketone **20** and **21** 92
2.7 Alternative route of β-ketoester **22** to alkene **9** 94
2.7.1 Secondary alcohol **S17** 94
2.7.2 Alkene **9** 95
2.8 Alternative route of secondary alcohol **S3** to diol **28** 96
2.8.1 Disubstituted alkene **S18** 96
2.8.2 Screening of the dihydroxylation of disubstituted alkene **S18** 97
2.8.3 Synthesis of DHQ-IND 100
2.9 Absolute configuration of tricycle **18** 102
2.9.1 Synthesis of (*R*)-Mosher ester **S19** 102
2.9.2 Synthesis of (*S*)-Mosher ester **S20** 103
2.9.3 Mosher ester analysis of **S19** and **S20** 105
3. X–ray 106
3.1 Tricycle **11** 106
3.2 Tricycle **18** 107
3.3 Norflickinflimiod C (**5**) 109
4. NMR Spectra 110
5. References 176

1. General Experimental Details

All reactions were performed in oven-dried glassware (110 °C oven temperature) with magnetic stirring under argon atmosphere, unless otherwise noted, using standard Schlenk techniques. If necessary, glassware was further dried under high-vacuum with a heat-gun at 650 °C. Temperature control was performed by external bath thermometers. High temperature reactions were either carried out using a reaction flask connected to a reflux condenser or in sealed pressure tubes while heating with a silicon oil bath. Low temperature reactions were either conducted using a distilled water/ice bath (0 °C) or using an acetone bath (Dewar vessel) in combination with an electronically controlled cryostat (–78 °C to 0 °C) or a Dewar vessel filled with dry ice/acetone (–78 °C). Diethyl ether and tetrahydrofuran (THF) were dried over molecular sieves (4Å) prior to use. All other solvents were purchased from Arcos Organics (Fisher Scientific) or Sigma Aldrich as 'extra dry' reagents. If required, solvents were either degassed by five freeze-pump-thaw cycles or by bubbling argon through the solvent under simultaneous sonication for at least 30 min. Solvents for extractions and flash column chromatography (FCC) were purchased in technical grade and purified by distillation prior to use. All reagents were obtained from commercial sources (Sigma Aldrich, Arcos Organics (Fisher Scientific), Alfa Aesar, Tokyo Chemical Industry, BLD Pharmatech, Fluorochem, Abcr, and ChemPUR) with a purity >95% and used without further purification unless otherwise noted. Particularly moisture or air sensitive reagents were handled in a glovebox. Transfer of these sensitive reagents or solutions of these was performed under argon atmosphere via syringes through rubber septa. If not noted otherwise, concentration of reaction mixtures or combined organic layers after extraction was performed on rotary evaporators with a bath temperature of 40 °C.

Flash column chromatography (FCC) was carried out using Merck silica gel 60 (0.040–0.063 mm). Analytical thin layer chromatography (TLC) was carried out using Merck silica gel 60 F254 aluminum foils and visualized under UV light at 254 nm or by staining with either ceric ammonium molybdate (CAM) or an aqueous potassium permanganate ($KMnO_4$) solution and subsequent heating.

High pressure liquid chromatography (HPLC) was conducted on a normal-phase Varian Dynamax column (250 x 41.4 mm Microsorb 60-8 Si column).

NMR spectra (^{1}H NMR, ^{13}C NMR and ^{19}F NMR) were recorded in deuterated chloroform (chloroform-*d*), deuterated methanol (methanol-d_4), deuterated dimethyl sulfoxide (dimethyl sulfoxide-d_6) or deuterated pyridine (pyridine-d_5) on a Bruker Avance Neo 400 MHz spectrometer, a Bruker Avance II 600 MHz spectrometer, or a Bruker Avance 4 Neo 700 MHz spectrometer. For ^{1}H NMR spectra the residual proton peak of the respective solvent (chloroform-*d*: 7.26 ppm, methanol-d_4: 3.31 ppm, dimethyl sulfoxide-d_6: 2.50 ppm, pyridine-d_5: 8.74 ppm, 7.58 ppm, 7.22 ppm) served as internal reference. ^{1}H spectroscopic data is reported as follows: chemical shift δ in ppm (multiplicity, coupling constant *J* in Hz, number of protons). Multiplicities are abbreviated as follows: s = singlet, d = doublet, t = triplet, q = quartet, p = quintet, h = hextet, br = broad, m = multiplet, or combinations thereof. Combined multiplicities are listed in order of their respective coupling constant *J* starting with the highest one. For ^{13}C NMR the central ^{13}C resonance of the respective solvent (chloroform-*d*: 77.16 ppm, methanol-d_4: 49.00 ppm, dimethyl sulfoxide-d_6: 39.52 ppm, pyridine-d_5: 150.35 ppm, 135.91 ppm, 123.87 ppm) served as internal reference and ^{13}C spectroscopic data is reported as follows: chemical shift δ in ppm (number of carbons in parenthesis if >1). NMR spectra were assigned using information ascertained from COSY, HMBC, HSQC and NOESY experiments. ^{19}F NMR spectra were externally referenced ($CFCl_3$).

High resolution mass spectra (HRMS) were recorded on a Thermo Scientific™ LTQ Orbitrap XL™ Hybrid Ion Trap-Orbitrap Mass Spectrometer at the Institute of Organic Chemistry and Center for Molecular Biosciences, University of Innsbruck.

Infrared spectra (IR) were recorded from 4000 cm^{-1} to 450 cm^{-1} on a Bruker™ ALPHA FT-IR spectrometer from Bruker. Samples were measured as a neat film by evaporation of a solution in chloroform-*d*. IR data is reported as follows: frequency of absorption in cm^{-1} (absorption intensity), whereby the absorption intensity is abbreviated as follows: w = weak, m = medium, s = strong, br = broad or combinations thereof.

Melting Points were measured with a SRS MPA120 EZ-Melt Melting Point Apparatus in open glass capillaries and are uncorrected.

Optical rotation values were recorded on a Schmidt+Haensch UniPol L1000 Peltier polarimeter. The specific rotation is calculated as follows: $[\alpha]_{\lambda}^{T} = \frac{\alpha \times 100}{c \times d}$. Thereby, the wavelength λ is reported in nm and the measuring temperature in °C. α represents the recorded optical rotation, *c* the concentration of the analyte in 10 mg/mL and *d* the length of the cuvette in dm. Thus, the specific

rotation is given in 10^{-1} deg cm^2 g^{-1}. Use of the sodium *D* line (λ = 589 nm) is indicated by *D* instead of the wavelength in nm. The sample concentration as well as the solvent is reported within the respective characterization data of each compound later in the experimental section.

ECD spectra were measured with a JASCO J-1500 CD Spectrometer using a JASCO CTU-100 circulating thermostat unit for temperature control.

For **X-ray diffraction analysis**, data collections were performed on a Bruker D8Quest using MoKα-radiation (λ = 0.71073 Å, Incoatec Microfocus). The Bruker Apex III software was applied for the integration, scaling and multi-scan absorption correction of the data. Structures were solved by direct methods with SHELXTL-XT-2014. Structure refinement was performed by least-squares methods against F2 with SHELXL-2014/7. All non-hydrogen atoms were refined anisotropically. The hydrogen atoms were placed in ideal geometry riding on their parent atoms. Relevant details of the data collection and evaluation are listed in tables at the corresponding sections. Supplementary crystallographic data for **11** (CCDC 2194515), **18** (CCDC 2194516) and **5** (CCDC 2194517), can be obtained from the Cambridge Crystallographic Data Centre CCDC deposition service via www.ccdc.cam.ac.uk/structures on quoting the deposition number CCDC 2194515–2194517. Further details are summarized in the tables at the corresponding sections. Plotting of thermal ellipsoids in this document and in the main text was carried out using MERCURY for Windows at 50% probability level.

All yields are isolated, unless otherwise specified.

2. Experimental Part

2.1 Synthetic intermediates

2.1.1 Geranyl bromide (**14**)

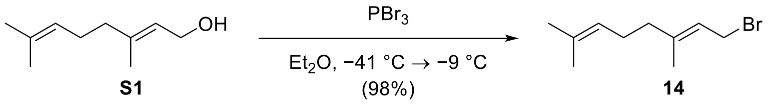

Geranyl bromide (**14**) was prepared according to a modified literature procedure[1]: To a stirred solution of geraniol (**S1**) (76.0 g, 97.0 wt%, 0.478 mol, 1 equiv) in diethyl ether (1.50 L) was added a solution of phosphorous tribromide (64.7 g, 239 mmol, 0.500 equiv) in diethyl ether (28 mL) dropwise at −41 °C via syringe pump (30 mL/h). After complete addition of phosphorous tribromide, the slightly yellowish solution was allowed to warm up to −9 °C over 6 h 30 min. Subsequently, the reaction mixture was poured into ice water (1.40 L) under vigorous stirring. The aqueous layer was separated and extracted with diethyl ether (3 × 600 mL). The combined organic layers were washed consecutively with a saturated aqueous solution of sodium bicarbonate (800 mL) and a saturated aqueous solution of sodium chloride (800 mL). The washed organic layers were dried over magnesium sulfate and the dried solution was filtered. The filtrate was concentrated under reduced pressure to afford geranyl bromide (**14**) (102 g, 470 mmol, 98%) as a colorless liquid.

The obtained analytical data were in accordance with reported literature values.[2]

2.1.2 Geranyl arene **15**

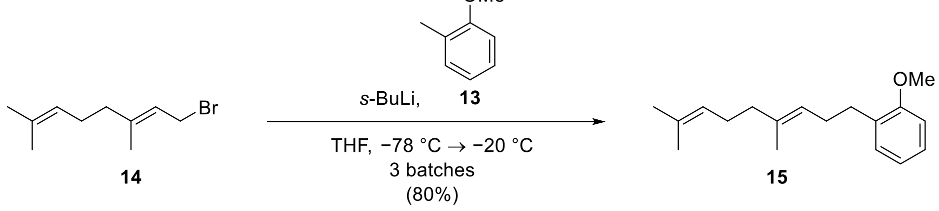

Owing to safety concerns regarding the large amount of *sec*-butyllithium, geranyl bromide (**14**) was divided into three equal batches and sequentially subjected to the reaction conditions:

To a solution of 2-methyl anisole (**13**) (27.1 g, 222 mmol, 1.42 equiv) in tetrahydrofuran (266 mL) was added *sec*-butyllithium (1.40 M in cyclohexane, 200 mL, 279 mmol, 1.79 equiv) at –78 °C over 25 min, during which the colorless solution turned deep yellow. After complete addition, the reaction mixture was allowed to warm up to –20 °C over the range of 3.5 h to 5 h. The deep yellow-orange reaction mixture was cooled again to –78 °C and geranyl bromide (**14**) (33.9 g, 156 mmol, 1 equiv) was added over 5 min resulting in a slight decolorization. After 5 min at –78 °C, water (20 mL) and a saturated aqueous solution of ammonium chloride (350 mL) were added. The cooling bath was removed, the reaction mixture was allowed to warm to 22 °C, and additional water (50 mL) was added. The organic layer was separated and the aqueous layer was extracted with diethyl ether (3 × 300 mL). The combined organic layers were washed with a saturated aqueous solution of sodium chloride (200 mL), dried over sodium sulfate, the dried solution was filtered, and the filtrate was concentrated under reduced pressure. The residues of all three batches were combined and purified by flash column chromatography on silica gel (diethyl ether : dichloromethane : cyclohexane, 0:10:90 grading to 0.5:10:89.5) to yield the title compound **15** (97.1 g, 376 mmol, 80%) as a colorless oil.

The obtained analytical data were in accordance with reported literature values.[3]

2.1.3 Diol **17**

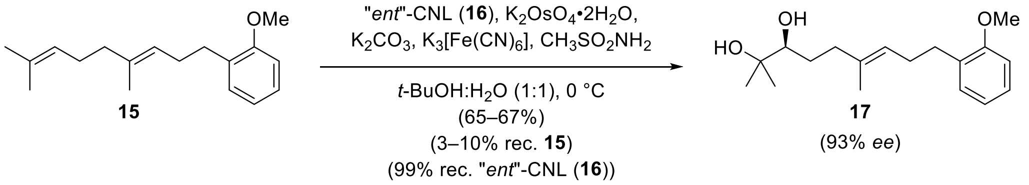

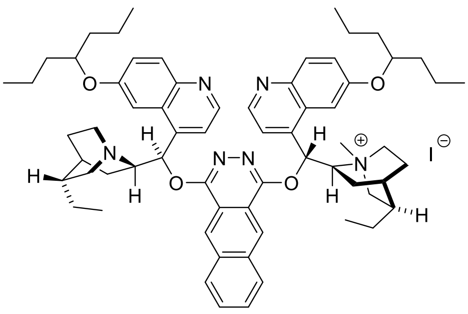

"*ent*"-Corey–Noe–Lin ligand (**16**)

Owing to a limited amount of the "*ent*"-Corey–Noe–Lin ligand (**16**), this step was performed in several batches with recovery and recycling of the "*ent*"-Corey–Noe–Lin ligand (**16**). A representative procedure is given below:

To a suspension of potassium carbonate (28.2 g, 204 mmol, 3.00 equiv), $K_3[Fe(CN)_6]$ (67.1 g, 204 mmol, 3.00 equiv)[1], "*ent*"-Corey–Noe–Lin ligand (**16**)[4] (1.16 g, 1.02 mmol, 1.50 mol%) and $K_2OsO_4{\cdot}2H_2O$ (188 mg, 510 μmol, 0.750 mol%) in water (340 mL) was added methane sulfonamide (6.46 g, 68.0 mmol, 1.00 equiv) and *tert*-butanol (290 mL). The biphasic, red-brownish mixture was sonicated for 15 min at 22 °C and then cooled to 0 °C before a solution of geranyl arene **15** (17.6 g, 68.0 mmol, 1 equiv) in *tert*-butanol (50 mL) was added. The biphasic reaction mixture was stirred vigorously at 0 °C for 26 h, after which sodium sulfite (85.7 g, 680 mmol, 10.0 equiv) was added at 0 °C and the reaction mixture was stirred for 30 min at 22 °C.[2] A 1 M aqueous sodium hydroxide solution (125 mL) was added and the resulting green mixture was extracted with ethyl acetate (5 × 250 mL). The combined organic layers were dried over sodium sulfate, the dried solution was filtered, and the filtrate was concentrated under reduced pressure. The residue was filtered through a silica plug and eluted with four column volumes of acetone, which were concentrated under reduced pressure and kept for isolation of diol **17**. Elution with concentrated aqueous ammonia : methanol : dichloromethane (NH_4OH : MeOH : CH_2Cl_2 = 1:25:74) then yielded recovered "*ent*"-Corey–Noe–Lin ligand (**16**) (1.14 g, 1.01 mmol, 99% recovery) as a beige solid. After concentration of the acetone-fractions under reduced pressure, the residue was purified by flash column chromatography on silica gel (40% ethyl acetate in cyclohexane) to give diol **17** (12.8 g, 43.9 mmol, 65%, 93% *ee*) as a white solid as well as recovered starting material **15** (1.75 g, 6.77 mmol, 10%) as a colorless oil.

The recovered "*ent*"-Corey–Noe–Lin ligand (**16**) did not exhibit any change in regio- and enantioselectivity.

Analytical data of diol **17**:

TLC (40% ethyl acetate in cyclohexane): R_f = 0.28 (UV, CAM).

mp: 47–48°C.

[1] Prior to use, potassium carbonate and $K_3[Fe(CN)_6]$ were ground to a fine powder in a mortar.
[2] To avoid overoxidation, the reaction was stopped before complete consumption of the starting material.

$[\alpha]_D^{20}$ = +18.3 (*c* = 1.14, dichloromethane).

^{1}H NMR (400 MHz, $CDCl_3$): δ 7.17 (td, *J* = 7.8, 1.8 Hz, 1H), 7.12 (dd, *J* = 7.4, 1.8 Hz, 1H), 6.91 – 6.82 (m, 2H), 5.28 (th, *J* = 7.1, 1.3 Hz, 1H), 3.83 (s, 3H), 3.31 (d, *J* = 10.5 Hz, 1H), 2.65 (t, *J* = 7.8 Hz, 2H), 2.30 (q, *J* = 7.7 Hz, 2H), 2.26 – 2.13 (m, 2H), 2.12 – 2.02 (m, 2H), 1.57 (d, *J* = 1.1 Hz, 3H), 1.63 – 1.52 (m, 1H), 1.40 (dddd, *J* = 13.9, 10.5, 8.4, 5.6 Hz, 1H), 1.19 (s, 3H), 1.15 (s, 3H).

^{13}C NMR (101 MHz, $CDCl_3$): δ 157.7, 135.4, 130.7, 130.0, 127.1, 125.1, 120.4, 110.4, 78.3, 73.1, 55.4, 36.9, 30.6, 29.7, 28.3, 26.5, 23.4, 15.9.

IR (ATR, neat): $\tilde{\nu}$ = 3405 (br), 2926 (w), 2855 (w), 2835 (w), 1601 (w), 1587 (w), 1493 (m), 1463 (m), 1439 (m), 1382 (m), 1327 (w), 1289 (w), 1241 (s), 1175 (m), 1160 (m), 1112 (m), 1075 (m), 1052 (m), 1032 (m), 960 (w), 931 (w), 905 (w), 848 (w), 816 (w), 751 (s), 732 (m), 583 (w), 504 (w), 467 (w) cm^{-1}.

HRMS (ESI): calcd for $C_{18}H_{28}NaO_3^+$ $[M+Na]^+$: 315.1931; found: 315.1925.

Chiral high performance liquid chromatography (HPLC) was conducted on a Daicel Chiralpak IB column (Shimadzu LC-2030, 1 mL/min flow rate, isocratic elution, 2% *iso*-propanol in *n*-hexane, λ = 210 nm, t_R (minor) = 27.3 min, t_R (major) = 18.0 min). An approximately 1:1 mixture of diol **17** originating from either dihydroxylation with the "*ent*"-Corey–Noe–Lin ligand (**16**) (leading to (*S*)-**17**) or with the Corey–Noe–Lin ligand (**S14**) (leading to (*R*)-**17**) served as a "racemic" reference.

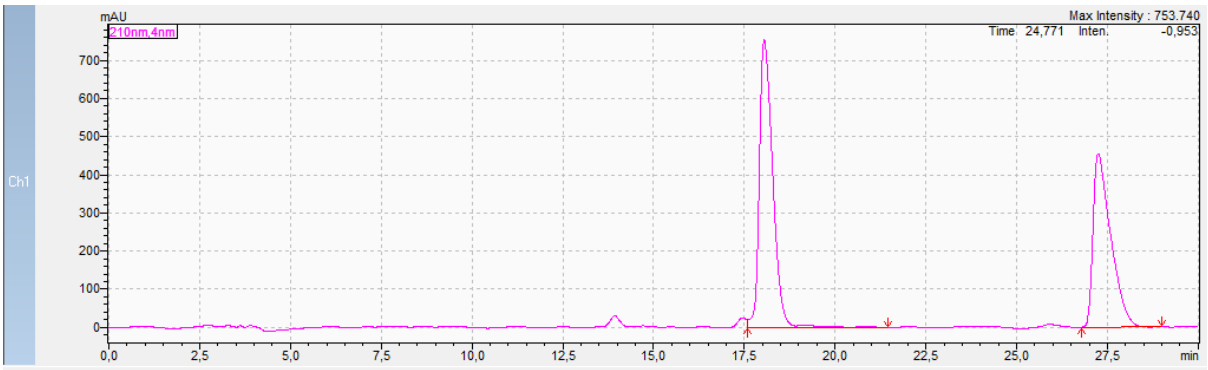

Results View - Peak Table

Peak Table | Compound | Group | Calibration Curve

Peak#	Ret. Time	Area	Height	Mark	Area%
1	18,047	19867072	754775		56,585
2	27,250	15242938	454648	M	43,415
Total		35110011	1209423		100,000

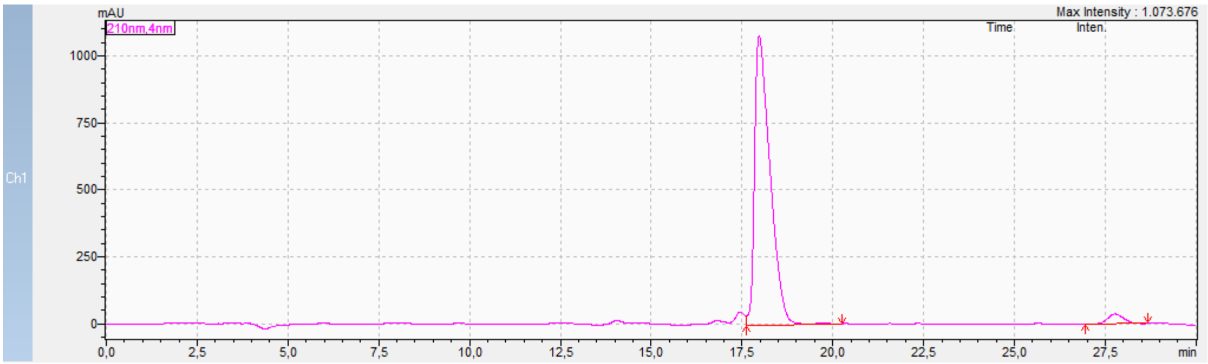

Results View - Peak Table

Peak Table | Compound | Group | Calibration Curve

Peak#	Ret. Time	Area	Height	Mark	Area%
1	17,969	32182218	1079257	M	96,746
2	27,766	1082338	36081	M	3,254
Total		33264556	1115338		100,000

2.1.4 Epoxide **12**

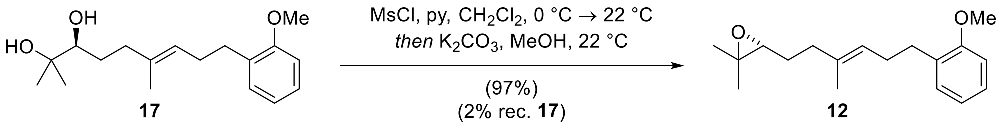

To a solution of diol **17** (32.6 g, 111 mmol, 1 equiv) and dry pyridine (62.8 mL, 779 mmol, 7.00 equiv) in dichloromethane (320 mL) was added methanesulfonyl chloride (13.9 mL, 178 mmol, 1.60 equiv) at 0 °C over 5 min, during which the solution turned yellow-orange.[5] After complete addition, the cooling bath was removed and the reaction mixture was stirred for 4.5 h at 22 °C before more methanesulfonyl chloride (2.60 mL, 33.4 mmol, 0.300 equiv) was added at 22 °C. After stirring for additional 3.5 h at 22 °C, dry methanol (1.54 L) and potassium carbonate (61.5 g, 445 mmol, 4.00 equiv) were added successively at 22 °C. After stirring for 14.5 h, the thick slurry was concentrated to approx. half of its original volume and water (400 mL) was added. The mixture was extracted with diethyl ether (4 × 400 mL), the combined organic layers were dried over sodium

sulfate, and the dried solution was filtered. The filtrate was concentrated under reduced pressure and the residue was purified by flash column chromatography on silica gel (10% diethyl ether in *n*-pentane grading to 40% ethyl acetate in cyclohexane) to yield epoxide **12** (29.5 g, 108 mmol, 97%) as a colorless oil as well as recovered starting material **17** (543 mg, 1.86 mmol, 2%) as a white solid.

<u>Analytical data of epoxide **12**:</u>

TLC (10% diethyl ether in *n*-pentane): R_f = 0.40 (UV, CAM).

$[\alpha]_D^{20}$ = +3.0 (c = 0.93, dichloromethane).

^{1}H NMR (400 MHz, $CDCl_3$): δ 7.17 (td, *J* = 7.8, 1.8 Hz, 1H), 7.12 (dd, *J* = 7.4, 1.8 Hz, 1H), 6.88 (td, *J* = 7.5, 1.2 Hz, 1H), 6.84 (d, *J* = 8.5 Hz, 1H), 5.27 (tq, *J* = 7.2, 1.3 Hz, 1H), 3.83 (s, 3H), 2.70 (t, *J* = 6.3 Hz, 1H), 2.64 (dd, *J* = 9.1, 6.6 Hz, 2H), 2.34 – 2.24 (m, 2H), 2.22 – 2.02 (m, 2H), 1.72 – 1.56 (m, 2H), 1.59 (d, *J* = 1.3 Hz, 3H), 1.31 (s, 3H), 1.27 (s, 3H).

^{13}C NMR (101 MHz, $CDCl_3$): δ 157.6, 134.6, 130.7, 130.0, 127.1, 124.9, 120.4, 110.3, 64.3, 58.5, 55.3, 36.4, 30.6, 28.3, 27.6, 25.0, 18.9, 16.0.

IR (ATR, neat): $\tilde{\nu}$ = 2958 (w), 2924 (w), 2856 (w), 2836 (w), 1601 (w), 1587 (w), 1493 (m), 1462 (m), 1439 (m), 1377 (m), 1324 (w), 1289 (w), 1240 (s), 1177 (w), 1161 (w), 1148 (w), 1112 (m), 1051 (m), 1031 (m), 924 (w), 899 (w), 872 (w), 820 (w), 794 (w), 750 (s), 732 (m), 680 (w), 565 (w), 522 (w), 496 (w), 459 (w), 422 (w), 409 (w) cm^{-1}.

HRMS (ESI): calcd for $C_{18}H_{26}NaO_2^+$ [M+Na]$^+$: 297.1825; found: 297.1813.

2.1.5 Tricycle **11**

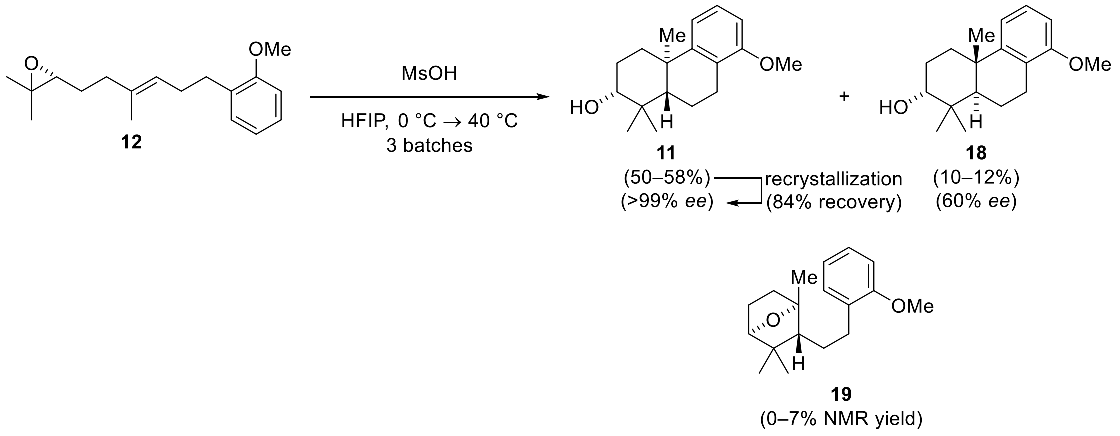

Epoxide **12** was divided into three equal batches and sequentially subjected to the reaction conditions. 1,1,1,3,3,3-hexafluoro-2-propanol (HFIP) was recycled and reused for the subsequent batches.

To a solution of epoxide **12** (10.6 g, 38.6 mmol, 1 equiv) in 1,1,1,3,3,3-hexafluoro-2-propanol (HFIP) (800 mL) was added dropwise methanesulfonic acid (261 µL, 3.88 mmol, 10.0 mol%) at 0 °C over 60 sec, during which the reaction mixture turned wine red. After complete addition, the reaction mixture was stirred for 4 min at 0 °C. Next, the reaction mixture was stirred at 40 °C until thin layer chromatography (TLC) indicated only minor residues of 7-oxabicyclo[2.2.1]heptane **19** (typically around 1 h 25 min to 1 h 40 min; see representative TLC image).[3] Then, potassium carbonate (1.87 g, 13.5 mmol, 0.350 equiv) was added at 40 °C and the reaction mixture was stirred for 15 min at 40 °C. 1,1,1,3,3,3-hexafluoro-2-propanol (HFIP) was removed under reduced pressure (and used for the next batch). The residue was filtered through a silica plug, which was eluted with four column volumes of ethyl acetate. The filtrates of all three batches were combined, concentrated under reduced pressure and the residue was purified by flash column chromatography on silica gel (10% diethyl ether in *n*-pentane grading to 20% ethyl acetate in *n*-pentane) to yield 7-oxabicyclo[2.2.1]heptane **19** (2.29 g, 8.35 mmol, 7% NMR yield[4]) along with an inseparable impurity as a colorless oil, tricycle **18** (3.80 g, 13.8 mmol, 12%) as a white solid and tricycle **11** (16.11 g, 58.7 mmol, 50%) as a white solid. For characterization, a small aliquot of impure 7-oxabicyclo[2.2.1]heptane **19** was purified by semipreparative normal-phase high performance liquid chromatography (HPLC) (3% grading to 6% ethyl acetate in *n*-hexane over 30 min) to obtain pure 7-oxabicyclo[2.2.1]heptane **19** as a colorless oil.

[3] After a certain number of uses recycled HFIP may (presumably due to an increased water content) show only slow conversion of the 7-oxabicyclo[2.2.1]heptane **19**. In these cases, sequential addition of more methanesulfonic acid in 0.10 equiv steps (until conversion is observed via TLC) is advised.
[4] Yield was determined through addition of 1,1,2,2-tetrachloroethane as a NMR standard.

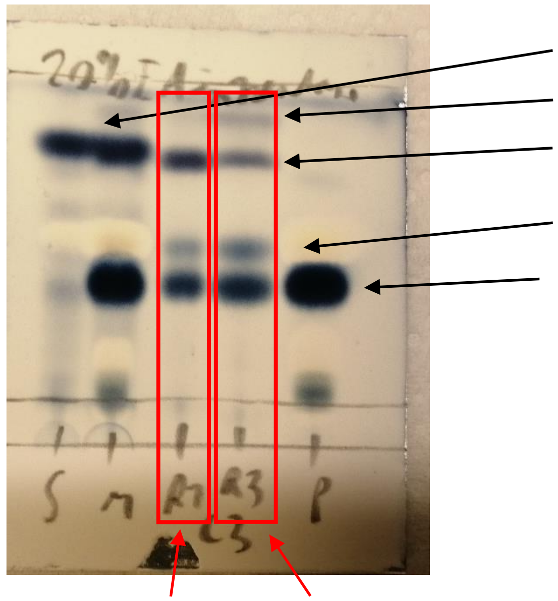

Recrystallization: Tricycle **11** (13.7 g, 50.0 mmol) was dissolved in diethyl ether (300 mL) at 40 °C and the solution was concentrated under reduced pressure (30 °C bath temperature) until approximately 25 mL of a suspension with white needles remain. The resulting white needles (1st recrystallization) were filtered off, washed with diethyl ether (4 × 10 mL) and dried under high vacuum. The filtrate was concentrated under reduced pressure (30 °C bath temperature) until approximately 10 mL of a suspension with white needles remain. The resulting white needles (2nd recrystallization) were filtered off, washed with diethyl ether (3 × 5 mL) and dried under high vacuum. The filtrate was slowly concentrated under atmoshperhic pressure at 22 °C until approximately 4 mL of a suspension with colorless hexagonal crystals remain. The resulting colorless hexagonal crystals (3rd recrystallization) were filtered off, washed successively with *n*-pentane (3 × 1 mL) and diethyl ether:*n*-pentane (1:1 v/v, 2 × 2 mL) and dried under high vacuum. After determination of the enantiomeric excess using chiral high performance liquid chromatography (HPLC), the 1st and 2nd recrystallization fractions were pooled to afford tricycle **11** (11.5 g, 41.9 mmol, 84% recovery, >99% *ee*) as white needles. The 3rd recrystallization afforded tricycle **11** (0.97 g, 3.5 mmol, 7% recovery, 59% *ee*) as colorless hexagonal crystals.

Colorless hexagonal crystals (59% *ee*) of tricycle **11** suitable for single crystal X-ray analysis were obtained by slow evaporation of a solution in diethyl ether.

White crystals of tricycle **18** suitable for single crystal X-ray analysis were obtained by slow evaporation of a solution in methanol.

The same reaction was conducted also on smaller scale: Epoxide **12** (18.4 g, 67.1 mmol, split into three batches of 6.13 g each), 30 mM instead of 50 mM in 1,1,1,3,3,3-hexafluoro-2-propanol (HFIP). Hereby, the reaction was stopped after complete conversion of 7-oxabicyclo[2.2.1]heptane **19** (along with more pronounced decomposition) thus affording tricycle **11** (10.7 g, 39.1 mmol, 58%) as a white solid and tricycle **18** (1.91 g, 6.96 mmol, 10%) as a white solid.

Analytical data of 7-oxabicyclo[2.2.1]heptane **19**:

TLC (3% ethyl acetate in cyclohexane): R_f = 0.14 (UV, CAM).

$[\alpha]_D^{20}$ = –39.0 (c = 0.947, dichloromethane).

^{1}H NMR (400 MHz, $CDCl_3$): δ 7.18 (td, J = 7.8, 1.8 Hz, 1H), 7.14 (dd, J = 7.4, 1.8 Hz, 1H), 6.89 (td, J = 7.4, 1.2 Hz, 1H), 6.85 (dd, J = 8.1, 1.1 Hz, 1H), 3.82 (s, 3H), 3.74 (d, J = 5.4 Hz, 1H), 2.62 (ddd, J = 13.4, 10.6, 6.0 Hz, 1H), 2.52 (ddd, J = 13.4, 10.3, 5.9 Hz, 1H), 1.93 (ddd, J = 12.4, 9.0, 4.7 Hz, 1H), 1.69 (tdd, J = 12.4, 5.5, 4.6 Hz, 1H), 1.62 – 1.49 (m, 3H), 1.45 (td, J = 12.0, 4.7 Hz, 1H), 1.36 (s, 3H), 1.29 (dd, J = 8.3, 6.1 Hz, 1H), 1.10 (s, 3H), 1.09 (s, 3H).

^{13}C NMR (101 MHz, $CDCl_3$): δ 157.5, 131.4, 129.7, 127.1, 120.5, 110.3, 86.9, 86.2, 56.1, 55.3, 45.5, 39.2, 30.9, 28.5, 26.2, 25.9, 23.4, 19.0.

IR (ATR, neat): $\tilde{\nu}$ = 2958 (m), 2869 (w), 2835 (w), 1601 (w), 1587 (w), 1493 (m), 1464 (m), 1439 (m), 1381 (w), 1364 (w), 1316 (w), 1291 (w), 1241 (s), 1191 (w), 1177 (w), 1138 (w), 1111 (w), 1071 (w), 1052 (w), 1033 (m), 993 (m), 929 (w), 872 (w), 833 (w), 806 (w), 750 (s), 643 (w), 600 (w), 545 (w), 496 (w), 472 (w) cm^{-1}.

HRMS (ESI): calcd for $C_{18}H_{27}O_2^+$ $[M+H]^+$: 275.2006; found: 275.1994.

Analytical data of tricycle **11**:

TLC (15% ethyl acetate in *n*-pentane): R_f = 0.31 (UV, CAM).

mp: 164–165 °C (for tricycle **11**, >99% *ee*), 122–123 °C (for tricycle ***rac*-11**).

$[\alpha]_D^{20}$ = –41.8 (c = 0.950, dichloromethane).

^{1}H NMR (400 MHz, $CDCl_3$): δ 7.13 (tt, *J* = 8.1, 0.7 Hz, 1H), 6.89 (dd, *J* = 8.1, 1.0 Hz, 1H), 6.65 (dd, *J* = 8.0, 1.0 Hz, 1H), 3.81 (s, 3H), 3.30 (dd, *J* = 11.2, 5.1 Hz, 1H), 2.92 (dd, *J* = 18.1, 6.5 Hz, 1H), 2.59 (dddd, *J* = 18.1, 11.8, 7.8, 1.0 Hz, 1H), 2.31 (dt, *J* = 13.1, 3.5 Hz, 1H), 1.94 (ddt, *J* = 13.5, 7.9, 1.8 Hz, 1H), 1.85 – 1.74 (m, 2H), 1.74 – 1.63 (m, 1H), 1.54 (td, *J* = 12.7, 5.0 Hz, 1H), 1.39 (s, 1H), 1.31 (dd, *J* = 12.5, 2.1 Hz, 1H), 1.21 (d, *J* = 0.8 Hz, 3H), 1.08 (s, 3H), 0.90 (s, 3H).

^{13}C NMR (101 MHz, $CDCl_3$): δ 157.2, 150.9, 126.3, 124.3, 116.7, 106.6, 78.9, 55.4, 49.5, 39.1, 37.7, 37.3, 28.3, 28.2, 25.0, 24.9, 18.3, 15.5.

IR (ATR, neat): $\tilde{\nu}$ = 3410 (br), 3069 (w), 2964 (m), 2940 (m), 2868 (m), 2835 (m), 1599 (w), 1580 (m), 1458 (s), 1436 (m), 1374 (w), 1348 (w), 1301 (w), 1256 (s), 1198 (w), 1158 (w), 1130 (w), 1097 (m), 1085 (m), 1061 (s), 1034 (s), 1006 (m), 973 (w), 936 (w), 909 (w), 870 (w), 778 (m), 718 (m), 642 (w), 609 (w), 553 (w), 513 (w), 428 (w) cm^{-1}.

HRMS (ESI): calcd for $C_{18}H_{27}O_2^+$ $[M+H]^+$: 275.2006; found: 275.2004.

Chiral high performance liquid chromatography (HPLC) was conducted on a Daicel Chiralpak IB column (Shimadzu LC-2030, 1 mL/min flow rate, isocratic elution, 1% *iso*-propanol in *n*-hexane, *λ* = 210 nm, t_R (minor) = 16.4 min, t_R (major) = 15.4 min). A racemic reference sample was prepared through an analgous cationic bicyclization from racemic epoxide **12** (see preparation in corresponding chapter).

Tricycle ***rac*-11**:

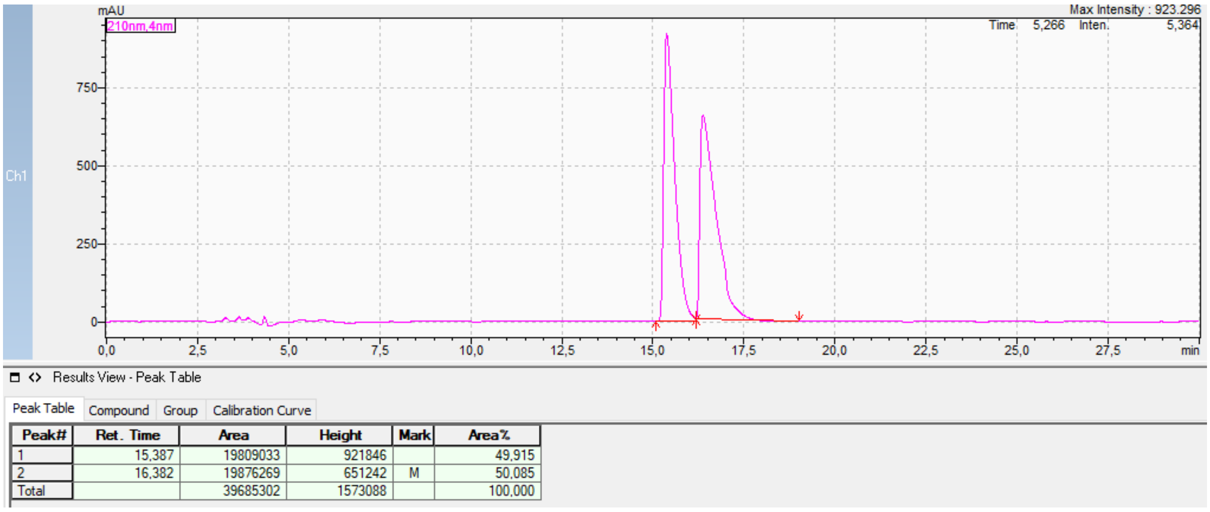

Peak#	Ret. Time	Area	Height	Mark	Area%
1	15,387	19809033	921846		49,915
2	16,382	19876269	651242	M	50,085
Total		39685302	1573088		100,000

1st and 2nd recrystallization of tricycle **11** (white needles):

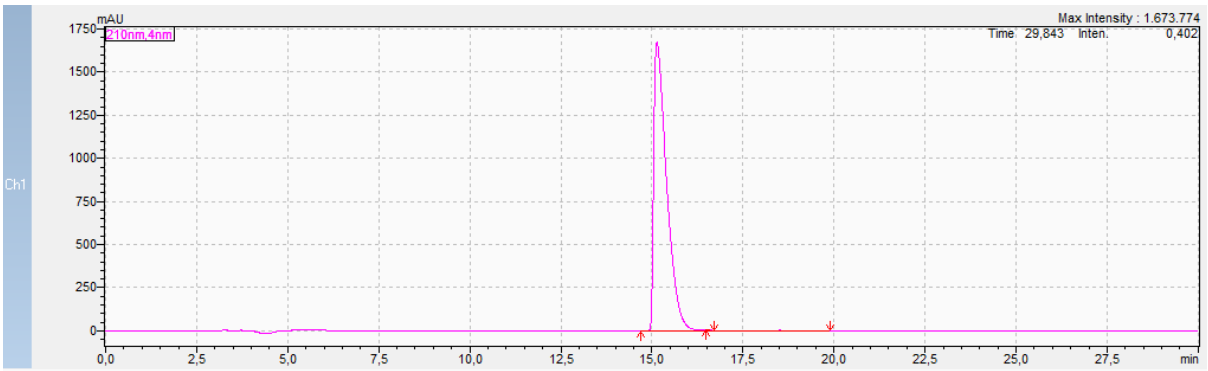

Results View - Peak Table

Peak Table | Compound | Group | Calibration Curve

Peak#	Ret. Time	Area	Height	Mark	Area%
1	15,138	41439300	1673992	S	99,981
2	16,575	7958	1533	T	0,019
Total		41447258	1675525		100,000

3rd recrystallization of tricycle **11** (colorless hexagonal plates):

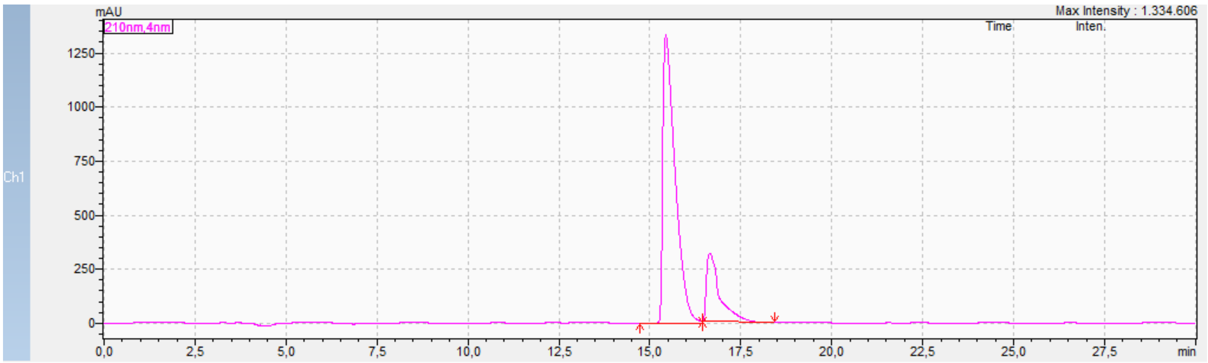

Results View - Peak Table

Peak Table | Compound | Group | Calibration Curve

Peak#	Ret. Time	Area	Height	Mark	Area%
1	15,446	31408506	1333937		79,676
2	16,642	8011998	315617	M	20,324
Total		39420504	1649554		100,000

<u>Analytical data of tricycle **18**:</u>

TLC (15% ethyl acetate in *n*-pentane): R_f = 0.48 (UV, CAM).

mp: 185–186 °C (for tricycle **18**, 60% *ee*), 190–191 °C (for tricycle ***rac*-18**).

$[\alpha]_{\mathbf{D}}^{\mathbf{20}}$ = +13.3 (*c* = 1.10, dichloromethane).

^{1}H NMR (400 MHz, $CDCl_3$): δ 7.12 (tt, *J* = 8.0, 0.9 Hz, 1H), 6.91 (dd, *J* = 8.0, 1.0 Hz, 1H), 6.65 (dd, *J* = 8.0, 1.0 Hz, 1H), 3.80 (s, 3H), 3.51 (q, *J* = 3.0 Hz, 1H), 2.89 (dd, *J* = 17.8, 6.4 Hz, 1H), 2.68 – 2.57 (m, 1H), 2.16 – 2.01 (m, 2H), 1.92 – 1.81 (m, 2H), 1.80 – 1.63 (m, 3H), 1.41 (d, *J* = 4.4 Hz, 1H), 1.21 (d, *J* = 0.8 Hz, 3H), 1.05 (s, 3H), 0.96 (s, 3H).

^{13}C NMR (101 MHz, $CDCl_3$): δ 157.2, 151.3, 126.2, 124.3, 116.6, 106.6, 75.8, 55.4, 43.3, 37.9, 37.6, 31.8, 28.3, 26.1, 24.7, 24.5, 22.3, 18.2.

IR (ATR, neat): $\tilde{\nu}$ = 3403 (br), 3066 (w), 2999 (w), 2959 (m), 2946 (m), 2934 (m), 2868 (m), 2836 (w), 1598 (w), 1577 (m), 1459 (m), 1435 (m), 1385 (w), 1375 (w), 1342 (w), 1298 (w), 1253 (s), 1206 (w), 1193 (w), 1152 (w), 1125 (w), 1095 (w), 1067 (s), 1055 (s), 1042 (m), 1015 (w), 986 (m), 963 (w), 953 (w), 942 (w), 925 (w), 911 (w), 854 (w), 780 (m), 735 (w), 720 (m), 665 (w), 631 (w), 608 (w), 505 (w) cm^{-1}.

HRMS (ESI): calcd for $C_{18}H_{27}O_2^+$ $[M+H]^+$: 275.2006; found: 275.2006.

Chiral high performance liquid chromatography (HPLC) was conducted on a Daicel Chiralpak IB column (Shimadzu LC-2030, 1 mL/min flow rate, isocratic elution, 5% *iso*-propanol in *n*-hexane, λ = 210 nm, t_R (minor) = 8.9 min, t_R (major) = 12.6 min). A racemic reference sample was prepared through an analogous cationic bicyclization from racemic epoxide **12** (see preparation in corresponding chapter).

Tricycle ***rac*-18**:

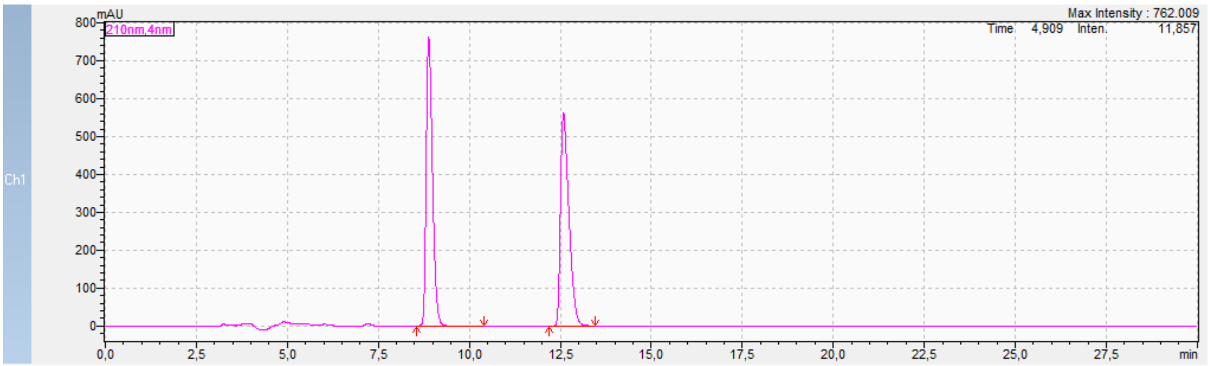

Results View - Peak Table

Peak Table | Compound | Group | Calibration Curve

Peak#	Ret. Time	Area	Height	Mark	Area%
1	8,885	9040490	762292		49,469
2	12,586	9234666	562902	M	50,531
Total		18275156	1325193		100,000

Tricycle **18**:

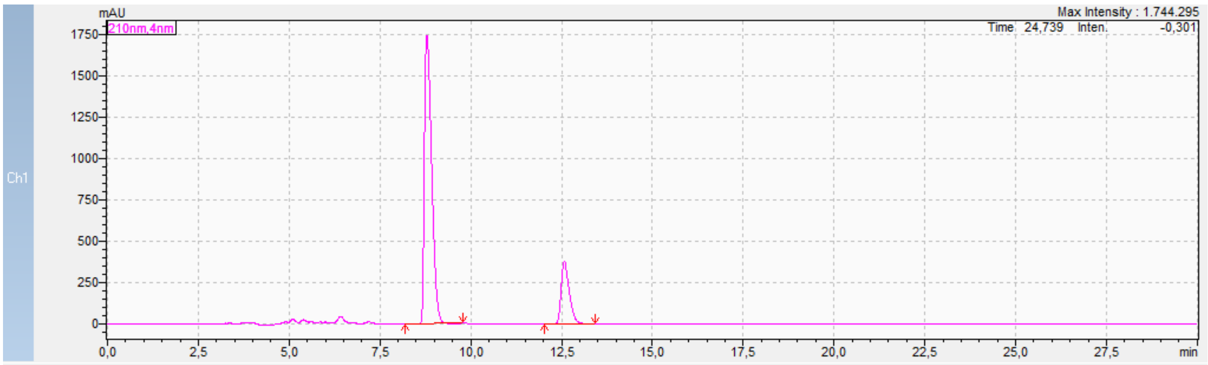

Results View - Peak Table

Peak Table | Compound | Group | Calibration Curve

Peak#	Ret. Time	Area	Height	Mark	Area%
1	8,794	23921925	1742601	M	80,024
2	12,575	5971406	378682	M	19,976
Total		29893331	2121283		100,000

2.1.6 Ketone **10**

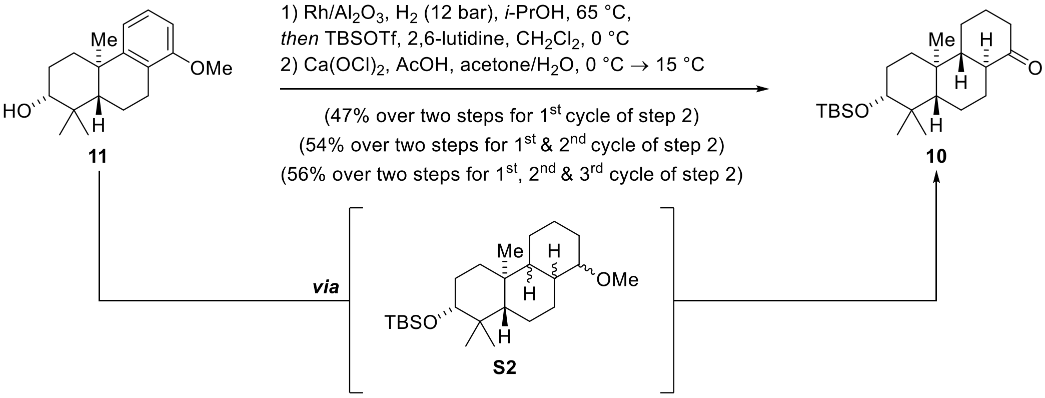

A suspension of tricycle **11** (5.00 g, 18.2 mmol, 1 equiv) and rhodium on activated alumina (2.8 g, 5.0 wt%, 1.4 mmol, 7.5 mol%) in isopropanol (40 mL) was stirred in an autoclave at 65 °C under a hydrogen pressure of 12 bar for 22 h.[6] After release of the hydrogen pressure and cooling to 22 °C, the reaction mixture was concentrated under reduced pressure. Chloroform (100 mL) was added, and the reaction mixture was concentrated under reduced pressure. Then, dichloromethane (100 mL) was added and the reaction mixture was concentrated under reduced pressure. To the residue was added dry dichloromethane (100 mL) and the blackish suspension was cooled to 0 °C. Next, 2,6-lutidine (5.29 mL, 45.5 mmol, 2.50 equiv) and *tert*-butyldimethylsilyl trifluoromethanesulfonate (5.22 mL, 22.8 mmol, 1.25 equiv) were added consecutively at 0 °C. After 35 min at 0 °C, the reaction mixture was filtered through celite and the celite filter was washed with ethyl acetate (350 mL). The resulting organic layer was washed in succession with a saturated aqueous solution of ammonium chloride (50 mL), 1 M aqueous hydrochloric acid (3 × 50 mL), a saturated aqueous solution of sodium bicarbonate (50 mL), and a saturated aqueous solution of sodium chloride (50 mL). The washed organic layer was dried over sodium sulfate, the

dried solution was filtered, and the filtrate was concentrated under reduced pressure to afford an inseparable diastereomeric mixture of cyclohexanes **S2** bearing a methyl ether substitutent.

1st cycle of step 2:[7] To a stirred solution of the aforementioned crude reaction mixture in acetone (630 mL) and water (70 mL) was added acetic acid (4.22 mL, 73.8 mmol, 4.05 equiv) at 22 °C. After cooling to 0 °C, calcium hypochlorite (6.8 g, 67 wt%, 32 mmol, 1.8 equiv) was added and the turbid, white suspension was stirred at 0 °C for 46.5 h before additional acetic acid (445 µL, 7.77 mmol, 0.427 equiv) and calcium hypochlorite (0.72 g, 67 wt%, 3.4 mmol, 0.18 equiv) were added in succession at 0 °C. Stirring was continued for 24 h at 0 °C, after which additional acetic acid (889 µL, 15.5 mmol, 0.852 equiv) and calcium hypochlorite (1.4 g, 67 wt%, 6.7 mmol, 0.37 equiv) were added consecutively at 0 °C. After 20.5 h at 0 °C, acetone (70 mL) was added, and the reaction mixture was stirred for 24 h at 5 °C followed by 12 h at 8 °C. As thin layer chromatography indicated no further conversion of the starting material to the desired ketone **10**, a saturated aqueous solution of sodium thiosulfate (100 mL) was added and stirring was continued for 10 min at 8 °C. Water (200 mL) and a saturated aqueous solution of sodium bicarbonate (100 mL) were added and the mixture was extracted with ethyl acetate (4 × 200 mL). The combined organic layers were washed with a saturated aqueous solution of sodium chloride (200 mL), the washed solution was dried over sodium sulfate, the dried organic layer was filtered, and the filtrate was concentrated under reduced pressure. The residue was purified by flash column chromatography on silica gel (10% diethyl ether in *n*-pentane) to yield title compound **10** (3.23 g, 8.53 mmol, 47% over two steps) as a white solid and a recovered diastereomeric mixture of methyl ethers **S2** (1.61 g, 4.08 mmol, 22% over two steps) as a white solid.

2nd cycle of step 2: To a stirred solution of the aforementioned mixture of methyl ethers **S2** (1.61 g, 4.08 mmol, 1 equiv) in acetone (170 mL) and water (17.0 mL) was added acetic acid (1.03 mL, 17.9 mmol, 4.40 equiv) at 22 °C. After cooling to 0 °C, calcium hypochlorite (1.7 g, 67 wt%, 7.8 mmol, 1.9 equiv) was added and the turbid, white suspension was stirred at 0 °C for 2 h before keeping the reaction mixture at 4 °C for 5 days without stirring. Then, stirring at 0 °C was continued for 21 h, after which additional acetic acid (216 µL, 3.78 mmol, 0.926 equiv) and a suspension of calcium hypochlorite (0.35 g, 67 wt%, 1.6 mmol, 0.40 equiv) in water (3 mL) were added in succession at 0 °C. After stirring for 17 h at 0 °C, the reaction mixture was allowed to warm up to 15 °C over 10 h, during which thin layer chromatography indicated no further conversion of the starting material **S2** and rather decomposition of product and starting material was observed. A

saturated aqueous solution of sodium thiosulfate (50 mL), water (40 mL) and a saturated aqueous solution of sodium bicarbonate (50 mL) were added and the mixture was extracted with diethyl ether (4 × 100 mL). The combined organic layers were washed with a saturated aqueous solution of sodium chloride (2 × 100 mL), the washed solution was dried over sodium sulfate, the dried organic layer was filtered, and the filtrate was concentrated under reduced pressure. The residue was purified by flash column chromatography on silica gel (10% diethyl ether in *n*-pentane) to yield title compound **10** (468 mg, 1.24 mmol, 30% for 2nd cycle, additional 7% over two steps overall) as a white solid and a recovered diastereomeric mixture of methyl ethers **S2** (547 mg, 1.39 mmol, 34% for 2nd cycle) as a white solid.

<u>3rd cycle of step 2:</u> To a stirred solution of the aforementioned mixture of methyl ethers **S2** (547 mg, 1.39 mmol, 1 equiv) in acetone (52 mL) and water (5.8 mL) was added acetic acid (349 µL, 6.10 mmol, 4.40 equiv) at 22 °C. After cooling to 0 °C, calcium hypochlorite (0.56 g, 67 wt%, 2.6 mmol, 1.9 equiv) was added and the turbid, white suspension was stirred at 0 °C for 3.5 h before keeping the reaction mixture at 4 °C for 2 days 14 h without stirring. Then, additional acetic acid (349 µL, 6.10 mmol, 4.40 equiv) and calcium hypochlorite (0.56 g, 67 wt%, 2.6 mmol, 1.9 equiv) were added and stirring was continued at 0 °C for 7 h. The reaction mixture was kept at 4 °C without stirring for 2 days 20 h, then sonicated at 0 °C for 1 h and again kept at 4 °C without stirring for 3 days 18 h. As thin layer chromatography indicated no further conversion of the starting material **S2** to the desired ketone **10**, a saturated aqueous solution of sodium thiosulfate (40 mL) and a saturated aqueous solution of sodium bicarbonate (40 mL) were added and the mixture was extracted with diethyl ether (4 × 100 mL).The combined organic layers were washed with a saturated aqueous solution of sodium chloride (1 × 80 mL, 2 × 50 mL), the washed solution was dried over sodium sulfate, the dried organic layer was filtered, and the filtrate was concentrated under reduced pressure. The residue was purified by flash column chromatography on silica gel (10% diethyl ether in *n*-pentane) to yield title compound **10** (154 mg, 406 µmol, 29% for 3rd cycle, additional 2% over two steps overall) as a white solid and a recovered diastereomeric mixture of methyl ethers **S2** (222 mg, 562 µmol, 41% for 3rd cycle) as a white solid.

<u>Analytical data of ketone **10**:</u>

TLC (10% diethyl ether in *n*-pentane): R_f = 0.32 (CAM).

mp: 119–120°C.

$[\alpha]_D^{20}$ = +3.22 (c = 1.10, dichloromethane).

^{1}H NMR (400 MHz, $CDCl_3$): δ 3.16 (dd, J = 11.2, 4.7 Hz, 1H), 2.34 (dddd, J = 13.5, 4.3, 2.6, 1.6 Hz, 1H), 2.28 – 2.15 (m, 2H), 2.06 (ddq, J = 11.8, 5.8, 3.0 Hz, 1H), 2.01 – 1.94 (m, 1H), 1.86 – 1.78 (m, 1H), 1.74 – 1.64 (m, 2H), 1.63 – 1.45 (m, 3H), 1.43 – 1.21 (m, 3H), 1.09 (td, J = 12.0, 3.5 Hz, 1H), 1.01 (td, J = 13.2, 3.9 Hz, 1H), 0.93 (d, J = 0.8 Hz, 3H), 0.88 (s, 12H), 0.78 – 0.73 (m, 4H), 0.03 (s, 3H), 0.02 (s, 3H).

^{13}C NMR (101 MHz, $CDCl_3$): δ 213.8, 79.4, 57.2, 53.9, 49.6, 42.0, 39.6, 37.6, 37.3, 28.8, 28.1, 26.6, 26.3, 26.0 (3C), 24.5, 20.6, 18.3, 16.2, 14.0, –3.7, –4.8.

IR (ATR, neat): $\tilde{\nu}$ = 2946 (s), 2855 (s), 1705 (s), 1471 (m), 1462 (w), 1439 (w), 1387 (w), 1370 (w), 1360 (w), 1314 (w), 1250 (m), 1219 (w), 1190 (w), 1152 (w), 1111 (m), 1068 (s), 1044 (w), 1007 (w), 991 (w), 970 (w), 940 (m), 887 (m), 835 (s), 801 (m), 774 (s), 679 (w), 666 (w), 578 (w) cm^{-1}.

HRMS (ESI): calcd for $C_{23}H_{42}NaO_2Si^+$ [M+Na]$^+$: 401.2846; found: 401.2838.

2.1.7 Ketone **20** and **21**

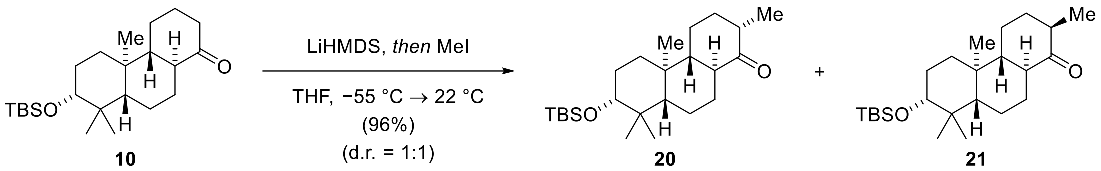

To a solution of ketone **10** (3.10 g, 8.20 mmol, 1 equiv) in tetrahydrofuran (185 mL) was added a solution of lithium bis(trimethylsilyl)amide in tetrahydrofuran (1.00 M, 9.43 mL, 9.43 mmol, 1.15 equiv) at –55 °C, whereupon the colorless solution turned yellow. The solution was allowed to warm-up over 2 h from –55 °C to –38 °C, after which the reaction mixture was cooled to –50 °C and methyl iodide (2.56 mL, 41.0 mmol, 5.00 equiv) was added. The reaction mixture was allowed to warm up to 22 °C through removal of the cooling bath. After stirring for 22 h, excess of methyl iodide was removed through addition of triethylamine (5.71 mL, 41.0 mmol, 5.00 equiv) and stirring for 15 min at 22 °C, during which a white precipitate formed. A saturated aqueous solution of ammonium chloride (150 mL) was added, and the mixture was extracted with diethyl ether (4 × 200 mL). The combined organic layers were washed with a saturated aqueous solution of sodium chloride (150 mL), the washed solution was dried over sodium sulfate, the dried organic layer was filtered, and the filtrate was concentrated under reduced pressure. The residue was purified by flash

column chromatography on silica gel (7% diethyl ether in *n*-pentane) to yield a diastereomeric mixture of ketone **20** and ketone **21** (3.08 g, 7.84 mmol, 96%, d.r. = 1:1) as a white solid. For characterization of the diastereomeric ketones, a small reference sample was purified by flash column chromatography on silica gel (2% grading to 5% diethyl ether in *n*-pentane) to yield ketone **20** as a white solid and ketone **21** as a white solid.

Analytical data of ketone **20**:

TLC (10% diethyl ether in *n*-pentane): R_f = 0.40 (CAM).

mp: 107–108 °C.

$[\alpha]_D^{20}$ = +29 (*c* = 0.62, dichloromethane).

^{1}H NMR (400 MHz, $CDCl_3$): δ 3.17 (dd, *J* = 11.2, 4.7 Hz, 1H), 2.54 – 2.43 (m, 1H), 2.37 (td, *J* = 11.9, 4.0 Hz, 1H), 2.00 – 1.92 (m, 1H), 1.79 – 1.64 (m, 4H), 1.63 – 1.49 (m, 4H), 1.34 – 1.24 (m, 2H), 1.15 (d, *J* = 7.3 Hz, 3H), 1.12 – 0.98 (m, 2H), 0.94 (d, *J* = 0.8 Hz, 3H), 0.88 (s, 12H), 0.77 – 0.72 (m, 4H), 0.03 (s, 3H), 0.03 (s, 3H).

^{13}C NMR (101 MHz, $CDCl_3$): δ 217.7, 79.4, 56.5, 53.9, 44.9, 44.0, 39.6, 37.4, 37.3, 31.7, 28.7, 28.1, 26.9, 26.0 (3C), 20.8, 18.9, 18.3, 17.2, 16.2, 14.0, –3.7, –4.8.

IR (ATR, neat): $\tilde{\nu}$ = 2935 (s), 2855 (m), 1705 (s), 1471 (m), 1462 (m), 1387 (w), 1361 (w), 1292 (w), 1252 (m), 1198 (w), 1137 (w), 1111 (m), 1083 (s), 1061 (m), 1006 (w), 993 (w), 970 (w), 939 (w), 885 (m), 860 (m), 834 (s), 773 (s), 670 (w), 553 (w), 487 (w) cm^{-1}.

HRMS (ESI): calcd for $C_{24}H_{44}NaO_2Si^+$ $[M+Na]^+$: 415.3003; found: 415.2996.

Analytical data of ketone **21**:

TLC (10% diethyl ether in *n*-pentane): R_f = 0.56 (CAM).

mp: 118–119 °C.

$[\alpha]_D^{20}$ = +4.13 (*c* = 1.44, dichloromethane).

^{1}H NMR (400 MHz, $CDCl_3$): δ 3.16 (dd, *J* = 11.2, 4.7 Hz, 1H), 2.38 – 2.23 (m, 1H), 2.19 (tdd, *J* = 11.9, 4.1, 1.4 Hz, 1H), 2.06 (ddt, *J* = 12.6, 5.6, 3.3 Hz, 1H), 1.92 (dq, *J* = 13.1, 3.4 Hz, 1H), 1.78 (dq, *J* = 13.2, 3.3 Hz, 1H), 1.74 – 1.62 (m, 2H), 1.62 – 1.30 (m, 4H), 1.29 – 1.16 (m, 2H), 1.09 (td, *J* = 12.0,

3.2 Hz, 1H), 1.03 (dd, *J* = 9.2, 4.3 Hz, 1H), 0.98 (d, *J* = 6.5 Hz, 3H), 0.93 (d, *J* = 0.7 Hz, 3H), 0.88 (s, 12H), 0.78 – 0.70 (m, 4H), 0.03 (s, 3H), 0.02 (s, 3H).

^{13}C NMR (101 MHz, $CDCl_3$): δ 214.8, 79.4, 58.0, 53.9, 49.4, 45.1, 39.6, 37.5, 37.4, 35.5, 28.8, 28.1, 26.7, 26.0 (3C), 24.6, 20.6, 18.2, 16.2, 14.6, 14.1, –3.7, –4.8.

IR (ATR, neat): $\tilde{\nu}$ = 2946 (m), 2924 (m), 2853 (m), 1704 (s), 1471 (w), 1455 (m), 1387 (w), 1369 (m), 1360 (m), 1325 (w), 1295 (w), 1249 (m), 1207 (w), 1190 (w), 1117 (m), 1088 (s), 1070 (s), 1034 (m), 1006 (m), 981 (m), 929 (m), 911 (w), 886 (s), 863 (m), 834 (s), 815 (m), 772 (s), 734 (m), 669 (m), 618 (w), 596 (w), 546 (w), 493 (w), 442 (w), 420 (w) cm^{-1}.

HRMS (ESI): calcd for $C_{24}H_{44}NaO_2Si^+$ $[M+Na]^+$: 415.3003; found: 415.2997.

2.1.8 β-Ketoester **22**

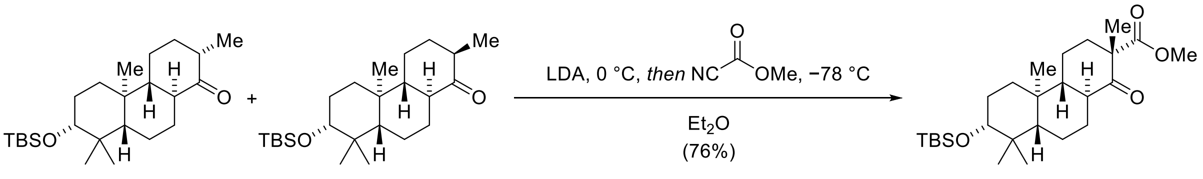

To a solution of diisopropylamine (1.43 mL, 10.2 mmol, 1.30 equiv) in diethyl ether (31.5 mL) was added a solution of *n*-butyllithium in hexanes (2.50 M, 3.76 mL, 9.41 mmol, 1.20 equiv) dropwise at –78 °C. After complete addition, the reaction mixture warmed to 0 °C and stirred for 45 min. Next, the thusly generated colorless lithium diispropylamide solution was added dropwise to a solution of ketone **20** and ketone **21** (3.08 g, 7.84 mmol, d.r. = 1:1, 1 equiv) in diethyl ether (187 mL) at 0 °C, whereupon the colorless solution turned first yellow, then pinkish. After stirring for 3 h at 0 °C, the reaction mixture was cooled to –78 °C and methyl cyanoformate (Mander's reagent) (778 μL, 9.80 mmol, 1.25 equiv) was added dropwise over 60 sec, during which the pinkish solution turned yellow/orange.[8] After stirring for 30 min at –78 °C, water (200 mL) was added at –78 °C and the mixture was allowed to warm up to 22 °C. The organic layer was separated, and the aqueous layer was extracted with diethyl ether (3 × 120 mL). The combined organic layers were washed with a saturated aqueous solution of sodium chloride (120 mL), the washed solution was dried over sodium sulfate, the dried organic layer was filtered, and the filtrate was concentrated under reduced

pressure. The residue was purified by flash column chromatography on silica gel (2.5% diethyl ether in *n*-pentane) to yield β-ketoester **22** (2.68 g, 5.95 mmol, 76%) as a white solid.

Analytical data of β-ketoester **22**:

TLC (10% diethyl ether in *n*-pentane): R_f = 0.40 (CAM).

mp: 127–128 °C.

$[\alpha]_D^{20}$ = +49.5 (*c* = 1.04, dichloromethane).

^{1}H NMR (400 MHz, $CDCl_3$): δ 3.71 (s, 3H), 3.15 (dd, *J* = 11.0, 4.7 Hz, 1H), 2.50 (dt, *J* = 13.6, 3.3 Hz, 1H), 2.32 (td, *J* = 11.9, 4.0 Hz, 1H), 1.97 (dq, *J* = 13.5, 3.4 Hz, 1H), 1.71 – 1.63 (m, 3H), 1.61 – 1.55 (m, 1H), 1.54 – 1.37 (m, 3H), 1.34 – 1.25 (m, 2H), 1.25 (s, 3H), 1.06 (td, *J* = 11.9, 3.4 Hz, 1H), 0.99 (td, *J* = 13.4, 12.9, 4.0 Hz, 1H), 0.89 – 0.87 (m, 12H), 0.87 (s, 3H), 0.75 (s, 3H), 0.74 (dd, *J* = 12.2, 2.4 Hz, 1H), 0.03 (s, 3H), 0.02 (s, 3H).

^{13}C NMR (101 MHz, $CDCl_3$): δ 209.6, 173.9, 79.4, 57.4, 56.5, 53.8, 52.6, 48.2, 39.6, 37.4, 37.4, 37.2, 28.8, 28.1, 26.9, 26.0 (3C), 21.9, 21.6, 20.5, 18.2, 16.2, 14.0, –3.7, –4.8.

IR (ATR, neat): $\tilde{\nu}$ = 2934 (m), 2854 (m), 1743 (m), 1713 (s), 1472 (w), 1452 (m), 1387 (w), 1375 (w), 1361 (w), 1314 (w), 1289 (w), 1250 (m), 1214 (m), 1198 (w), 1165 (m), 1153 (m), 1136 (w), 1112 (s), 1081 (s), 1029 (w), 1007 (w), 981 (w), 960 (w), 940 (m), 886 (m), 861 (m), 835 (s), 773 (s), 707 (w), 670 (w), 485 (w), 444 (w) cm^{-1}.

HRMS (ESI): calcd for $C_{26}H_{46}NaO_4Si^+$ $[M+Na]^+$: 473.3058; found: 473.3054.

2.1.9 Triflate **23**

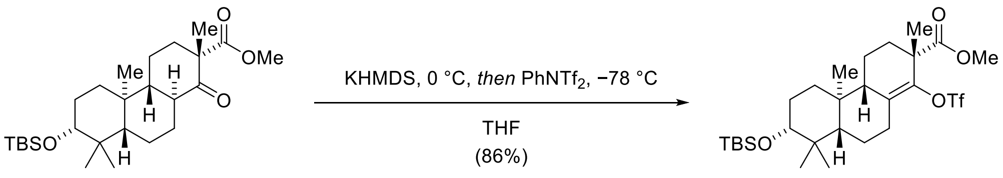

22 **23**

To a solution of β-ketoester **22** (2.50 g, 5.55 mmol, 1 equiv) in tetrahydrofuran (130 mL) was added a solution of potassium bis(trimethylsilyl)amide in tetrahydrofuran (1.00 M, 6.10 mL, 6.10 mmol, 1.10 equiv) at 0 °C, whereupon the colorless solution turned yellow. After stirring for 1 h 40 min at 0 °C, the reaction mixture was cooled to –78 °C. A solution of phenyl triflimide (2.18 g, 6.10 mmol,

1.10 equiv) in tetrahydrofuran (15 mL) was added at –78 °C causing the reaction mixture to adopt a deep yellow color. After stirring for 15 min at –78 °C, water (80 mL) and a saturated aqueous solution of ammonium chloride (40 mL) were added at –78 °C and the mixture was allowed to warm up to 22 °C. The organic layer was separated, and the aqueous layer was extracted with diethyl ether (3 × 100 mL). The combined organic layers were washed with a saturated aqueous solution of sodium chloride (50 mL), the washed solution was dried over sodium sulfate, the dried organic layer was filtered, and the filtrate was concentrated under reduced pressure. The residue was purified by flash column chromatography on silica gel (40% dichloromethane in cyclohexane) to yield triflate **23** (2.78 g, 4.78 mmol, 86%) as a white solid.

Analytical data of triflate **23**:

TLC (3% diethyl ether in *n*-pentane): R_f = 0.27 (CAM).

mp: 100–102 °C.

$[\alpha]_D^{20}$ = –6.16 (c = 0.824, dichloromethane).

^{1}H NMR (400 MHz, $CDCl_3$): δ 3.69 (s, 3H), 3.21 (dd, J = 10.6, 4.9 Hz, 1H), 2.95 – 2.88 (m, 1H), 2.14 (dt, J = 12.0, 2.6 Hz, 1H), 1.92 (dd, J = 9.8, 6.8 Hz, 1H), 1.81 – 1.70 (m, 2H), 1.64 – 1.50 (m, 5H), 1.51 – 1.33 (m, 2H), 1.38 (s, 3H), 1.13 (td, J = 12.9, 4.3 Hz, 1H), 1.02 (dd, J = 12.5, 2.5 Hz, 1H), 0.94 (s, 3H), 0.89 (s, 9H), 0.83 (s, 3H), 0.81 (s, 3H), 0.04 (s, 6H).

^{13}C NMR (101 MHz, $CDCl_3$): δ 174.6, 142.0, 133.5, 118.8 (q, J = 319.8 Hz), 79.5, 54.1, 52.5, 52.1, 47.7, 39.7, 39.3, 36.9, 36.8, 29.0, 28.6, 27.9, 26.0 (3C), 22.9, 21.5, 19.3, 18.3, 16.3, 14.0, –3.6, –4.8.

^{19}F NMR (377 MHz, $CDCl_3$): δ –74.3.

IR (ATR, neat): $\tilde{\nu}$ = 2951 (m), 2893 (w), 2855 (w), 1739 (m), 1674 (w), 1462 (w), 1400 (m), 1362 (w), 1272 (w), 1250 (m), 1207 (s), 1168 (w), 1139 (m), 1111 (m), 1099 (m), 1070 (w), 1056 (w), 1025 (w), 1002 (w), 961 (w), 939 (w), 920 (w), 882 (s), 837 (m), 806 (w), 774 (m), 734 (w), 694 (w), 678 (w), 637 (w), 608 (m), 577 (w), 541 (w), 511 (w) cm^{-1}.

HRMS (ESI): calcd for $C_{27}H_{45}F_3NaO_6SSi^+$ $[M+Na]^+$: 605.2550; found: 605.2540.

2.1.10 Alkene 9

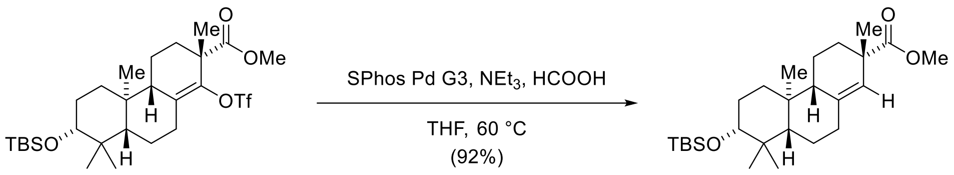

To a solution of triflate **23** (1.00 g, 1.72 mmol, 1 equiv) in degassed tetrahydrofuran[5] (17.0 mL) was added successively (2-dicyclohexylphosphino-2′,6′-dimethoxybiphenyl) [2-(2′-amino-1,1′-biphenyl)]palladium(II) methanesulfonate (SPhos Pd G3) (75.1 mg, 85.8 µmol, 4.99 mol%) and a degassed solution[6] of triethylamine (1.91 mL, 13.7 mmol, 8.00 equiv) and formic acid (327 µL, 8.58 mmol, 5.00 equiv) in degassed tetrahydrofuran[7] (17.0 mL) at 22 °C. The resulting mixture was heated to 60 °C for 4 h, during which the initially colorless solution turned yellow, then orange, then red-brownish and finally to a blackish suspension. The reaction mixture was cooled to 22 °C, a saturated aqueous solution of ammonium chloride (40 mL) was added, the organic layer was separated, and the aqueous layer was extracted with diethyl ether (3 × 40 mL). The combined organic layers were washed with a saturated aqueous solution of sodium chloride (50 mL), the washed solution was dried over sodium sulfate, the dried organic layer was filtered, and the filtrate was concentrated under reduced pressure. The residue was purified by flash column chromatography on silica gel (20% grading to 35% dichloromethane in cyclohexane) to yield alkene **9** (687 mg, 1.58 mmol, 92%) as a white solid.

Analytical data of alkene **9**:

TLC (3% diethyl ether in *n*-pentane): R_f = 0.32 (CAM).

mp: 108–109 °C.

$[\alpha]_{\mathrm{D}}^{20}$ = –46.2 (c = 1.11, dichloromethane).

^{1}H NMR (400 MHz, $CDCl_3$): δ 5.36 (q, J = 1.8, 1.4 Hz, 1H), 3.64 (s, 3H), 3.21 (dd, J = 10.5, 5.1 Hz, 1H), 2.31 (ddd, J = 14.2, 4.6, 2.0 Hz, 1H), 2.15 (dtd, J = 12.8, 3.6, 1.4 Hz, 1H), 2.00 (td, J = 13.7,

[5] Degassed by argon bubbling for at least 30 min under simultaneous sonication.
[6] Solution of triethylamine and formic acid in degassed tetrahydrofuran was additionally degassed by argon bubbling for 10 min under simultaneous sonication.
[7] Degassed by argon bubbling for at least 30 min under simultaneous sonication.

5.9 Hz, 1H), 1.70 – 1.43 (m, 6H), 1.43 – 1.31 (m, 2H), 1.18 (s, 3H), 1.15 – 1.06 (m, 2H), 1.00 (dd, *J* = 12.4, 2.7 Hz, 1H), 0.91 (s, 3H), 0.88 (s, 9H), 0.77 (s, 3H), 0.67 (s, 3H), 0.03 (s, 6H).

^{13}C NMR (101 MHz, $CDCl_3$): δ 177.7, 139.3, 125.4, 79.7, 54.3, 51.8, 50.7, 43.2, 39.7, 38.3, 37.1, 35.8, 33.6, 29.0, 28.1, 27.7, 26.1 (3C), 22.5, 20.3, 18.3, 16.3, 14.4, –3.6, –4.8.

IR (ATR, neat): $\tilde{\nu}$ = 2935 (m), 2853 (m), 1732 (s), 1472 (w), 1460 (w), 1433 (w), 1387 (w), 1361 (w), 1248 (m), 1200 (m), 1159 (m), 1136 (m), 1119 (m), 1099 (s), 1065 (m), 1054 (m), 1035 (w), 1004 (m), 968 (w), 938 (m), 914 (w), 883 (m), 859 (m), 833 (s), 772 (s), 698 (w), 667 (w), 594 (w), 540 (w), 488 (w) cm^{-1}.

HRMS (ESI): calcd for $C_{26}H_{46}NaO_3Si^+$ [M+Na]$^+$: 457.3108; found: 457.3100.

2.1.11 Secondary alcohol **S3**

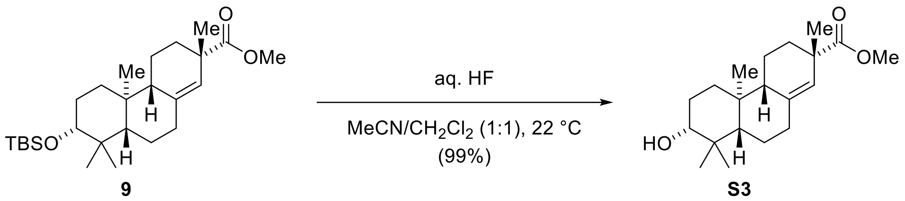

To a solution of alkene **9** (213.7 mg, 491.6 µmol, 1 equiv) in dichloromethane (6.00 mL) and acetonitrile (6.00 mL) was added an aqueous solution of hydrogen fluoride (1.78 mL, 48.0 wt%, 49.2 mmol, 100 equiv) at 22 °C. The biphasic colorless solution was stirred for 14 min at 22 °C, after which the reaction mixture was poured into a mixture of ethyl acetate (100 mL) and a 2 M aqueous solution of sodium hydroxide (60 mL). The organic layer was separated, and the aqueous layer was extracted with ethyl acetate (1 × 40 mL). The combined organic layers were successively washed with a saturated aqueous solution of ammonium chloride (40 mL) and a saturated aqueous solution of sodium chloride (40 mL). The washed solution was dried over sodium sulfate, the dried organic layer was filtered, and the filtrate was concentrated under reduced pressure. The residue was purified by flash column chromatography on silica gel (40% diethyl ether in *n*-pentane) to yield secondary alcohol **S3** (156.5 mg, 488.3 µmol, 99%) as a white solid.

Analytical data of secondary alcohol **S3**:

TLC (40% diethyl ether in *n*-pentane): R_f = 0.34 (CAM).

mp: 106–107 °C.

$[\alpha]_D^{20}$ = –50.9 (c = 1.03, dichloromethane).

^{1}H NMR (400 MHz, $CDCl_3$): δ 5.36 (q, J = 1.9 Hz, 1H), 3.63 (s, 3H), 3.24 (dd, J = 11.6, 4.0 Hz, 1H), 2.32 (ddd, J = 14.2, 4.6, 2.1 Hz, 1H), 2.15 (dtd, J = 12.8, 3.6, 1.5 Hz, 1H), 2.00 (tdt, J = 13.5, 5.8, 1.9, 1.4 Hz, 1H), 1.70 – 1.46 (m, 6H), 1.46 – 1.31 (m, 3H), 1.18 (s, 3H), 1.19 – 1.06 (m, 2H), 1.03 (dd, J = 12.4, 2.7 Hz, 1H), 1.00 (s, 3H), 0.80 (s, 3H), 0.67 (s, 3H).

^{13}C NMR (101 MHz, $CDCl_3$): δ 177.7, 139.0, 125.6, 79.2, 54.2, 51.8, 50.6, 43.2, 39.1, 38.4, 37.1, 35.7, 33.5, 28.6, 27.7 (2C), 22.2, 20.2, 15.8, 14.3.

IR (ATR, neat): $\tilde{\nu}$ = 3444 (br), 2939 (s), 2871 (m), 2849 (m), 1728 (s), 1515 (w), 1454 (m), 1434 (w), 1384 (m), 1364 (w), 1266 (m), 1245 (m), 1201 (s), 1159 (m), 1132 (s), 1119 (s), 1090 (s), 1057 (w), 1033 (s), 999 (m), 970 (m), 931 (m), 884 (w), 865 (m), 847 (m), 808 (w), 770 (w), 752 (w), 704 (w), 644 (w), 608 (w), 532 (w) cm^{-1}.

HRMS (ESI): calcd for $C_{20}H_{32}NaO_3^+$ $[M+Na]^+$: 343.2244; found: 343.2238.

2.1.12 Ketone **24**

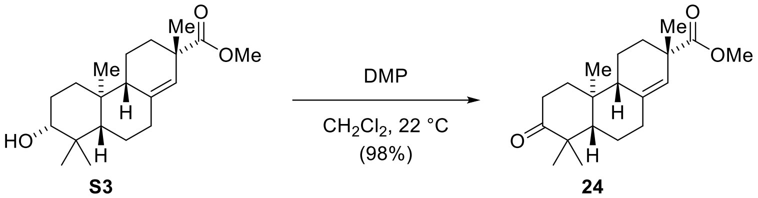

To a solution of secondary alcohol **S3** (71.7 mg, 224 µmol, 1 equiv) in dichloromethane (5.00 mL) was added Dess–Martin periodinane (DMP) (243 mg, 573 µmol, 2.56 equiv) at 22 °C. The resulting white suspension was stirred for 7 h at 22 °C, after which additional Dess–Martin periodinane (DMP) (49.9 mg, 118 µmol, 0.526 equiv) was added to the reaction mixture. After stirring for another 13 h at 22 °C, the reaction mixture was directly subjected to purification by flash column chromatography on silica gel (10% grading to 20% diethyl ether in *n*-pentane) to yield ketone **24** (69.7 mg, 219 µmol, 98%) as a white solid.

<u>Analytical data of ketone **24**:</u>

TLC (20% diethyl ether in *n*-pentane): R_f = 0.24 (CAM).

mp: 95–96 °C.

$[\alpha]_D^{20}$ = –22.4 (*c* = 1.12, dichloromethane).

¹H NMR (400 MHz, $CDCl_3$): δ 5.43 (q, *J* = 1.9 Hz, 1H), 3.66 (s, 3H), 2.63 (td, *J* = 14.7, 5.7 Hz, 1H), 2.37 (dt, *J* = 14.3, 3.1 Hz, 1H), 2.27 (ddd, *J* = 14.9, 4.4, 3.1 Hz, 1H), 2.18 (dtd, *J* = 12.9, 3.7, 1.4 Hz, 1H), 2.10 – 1.99 (m, 1H), 1.94 (ddd, *J* = 13.3, 5.7, 3.1 Hz, 1H), 1.76 (t, *J* = 8.3 Hz, 1H), 1.65 (ddt, *J* = 14.1, 7.1, 3.7 Hz, 1H), 1.59 – 1.36 (m, 5H), 1.20 (s, 3H), 1.14 (td, *J* = 13.3, 3.4 Hz, 1H), 1.08 (s, 3H), 1.05 (s, 3H), 0.90 (s, 3H).

¹³C NMR (101 MHz, $CDCl_3$): δ 216.8, 177.5, 138.2, 126.5, 55.5, 51.9, 49.8, 48.0, 43.2, 38.2, 37.7, 35.4, 34.9, 33.4, 27.6, 25.7, 23.2, 22.5, 20.3, 14.0.

IR (ATR, neat): $\tilde{\nu}$ = 2944 (m), 2872 (w), 2854 (w), 1729 (s), 1706 (s), 1456 (m), 1433 (m), 1385 (w), 1367 (w), 1266 (w), 1245 (w), 1199 (m), 1160 (m), 1133 (m), 1122 (m), 1087 (w), 1036 (w), 993 (w), 971 (w), 928 (w), 885 (w), 859 (w), 844 (w), 769 (w), 582 (w) cm^{-1}.

HRMS (ESI): calcd for $C_{20}H_{30}NaO_3^+$ $[M+Na]^+$: 341.2087; found: 341.2076.

2.1.13 α-Hydroxy ketone **25**

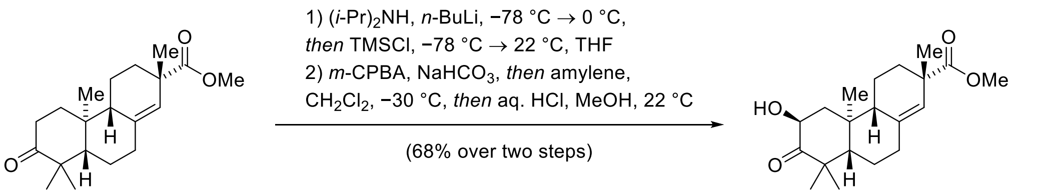

24 **25**

To a solution of diisopropylamine (173 µL, 1.23 mmol, 6.00 equiv) in tetrahydrofuran (5.00 mL) was added a solution of *n*-butyllithium in hexanes (639 µL, 1.60 M, 1.02 mmol, 5.00 equiv) at –78 °C. After 10 min at –78 °C, a solution of ketone **24** (65.1 mg, 204 µmol, 1 equiv) in tetrahydrofuran (7.00 mL) was added at –78 °C and the reaction mixture was warmed to 0 °C by exchange of the cooling bath. The yellow solution was stirred at 0 °C for 45 min. Next, the reaction mixture was cooled to –78 °C and chlorotrimethylsilane (182 µL, 1.43 mmol, 7.00 equiv) was added at –78 °C. After complete addition, the reaction mixture was allowed to warm to 22 °C through removal of

the cooling bath and stirring was continued for 20 min. Next, the reaction mixture was poured into a saturated aqueous solution of sodium hydrogencarbonate (30 mL), and the biphasic mixture was extracted with a 1:1 mixture of diethyl ether (70 mL) and *n*-pentane (70 mL). The organic layer was washed in succession with a saturated aqueous solution of sodium hydrogencarbonate (30 mL) and a saturated aqueous solution of sodium chloride (30 mL). The washed organic layer was dried over sodium sulfate, the dried organic layer was filtered, and the filtrate was concentrated under reduced pressure to give a yellow oil (113.9 mg), which was used for the next step without further purification.

To a solution of crude enol ether (113.9 mg, in theory: 79.7 mg, 204 µmol, 1 equiv) in dichloromethane (6.00 mL) was added successively sodium hydrogencarbonate (235 mg, 2.80 mmol, 13.7 equiv,) and *meta*-chloroperoxybenzoic acid (*m*-CPBA) (140 mg, 77.0 wt%, 623 µmol, 3.06 equiv) at –30 °C.[9] After stirring for 1 h 40 min at –30 °C, amylene (2-methyl-2-butene) (528 µL, 5.10 mmol, 25.0 equiv) was added at –30 °C and the reaction mixture was allowed to warm to 22 °C through removal of the cooling bath. After 10 min, methanol (6.00 mL) and an aqueous solution of hydrogen chloride (2.04 mL, 2.00 M, 4.08 mmol, 20.0 equiv) were added at 22 °C successively. After stirring at 22 °C for 15 min, the reaction was diluted with ethyl acetate (140 mL) and successively washed with a 1 M aqueous solution of sodium hydroxide (2 × 30 mL), with a saturated aqueous solution of ammonium chloride (30 mL) and with a saturated aqueous solution of sodium chloride (30 mL). The washed solution was dried over sodium sulfate, the dried organic layer was filtered, and the filtrate was concentrated under reduced pressure. The residue was purified by flash column chromatography on silica gel (20% grading to 30% ethyl acetate in cyclohexane) to yield α-hydroxy ketone **25** (46.6 mg, 139 µmol, 68% over two steps) as a viscous colorless oil.

Analytical data of α-hydroxy ketone **25**:

TLC (30% ethyl acetate in cyclohexane): R_f = 0.44 (CAM).

$[\alpha]_D^{20}$ = –49.9 (*c* = 0.718, dichloromethane).

^{1}**H NMR** (400 MHz, $CDCl_3$): δ 5.46 (q, *J* = 1.9 Hz, 1H), 4.51 (ddd, *J* = 13.1, 6.1, 3.9 Hz, 1H), 3.67 (s, 3H), 3.63 (d, *J* = 3.9 Hz, 1H), 2.40 (dt, *J* = 14.4, 3.1 Hz, 1H), 2.34 (dd, *J* = 12.6, 6.2 Hz, 1H), 2.19 (dtd, *J* = 13.1, 3.6, 1.5 Hz, 1H), 2.11 – 2.01 (m, 1H), 1.77 (t, *J* = 8.6 Hz, 1H), 1.67 (ddt, *J* = 13.9, 6.9, 3.6 Hz,

1H), 1.62 – 1.54 (m, 2H), 1.49 – 1.38 (m, 2H), 1.30 (t, *J* = 12.8 Hz, 1H), 1.21 (s, 3H), 1.19 – 1.13 (m, 1H), 1.16 (s, 3H), 1.12 (s, 3H), 1.02 (s, 3H).

^{13}C NMR (101 MHz, $CDCl_3$): δ 216.3, 177.4, 137.5, 127.3, 69.3, 56.5, 51.9, 50.1, 47.6, 47.6, 43.3, 38.5, 35.2, 33.3, 27.5, 25.2, 22.8, 22.4, 20.5, 15.2.

IR (ATR, neat): $\tilde{\nu}$ = 3483 (br), 2944 (m), 2873 (w), 2853 (w), 1727 (s), 1706 (s), 1456 (m), 1434 (m), 1389 (m), 1264 (m), 1193 (s), 1160 (m), 1135 (m), 1122 (s), 1096 (m), 1066 (w), 1051 (m), 1001 (m), 983 (w), 968 (m), 935 (w), 885 (w), 855 (w), 792 (w), 769 (w), 739 (w), 685 (w), 657 (w), 636 (w), 584 (w), 555 (w) cm^{-1}.

HRMS (ESI): calcd for $C_{20}H_{30}NaO_4^+$ $[M+Na]^+$: 357.2036; found: 357.2027.

2.1.14 Diols **26**, **27** and **28**

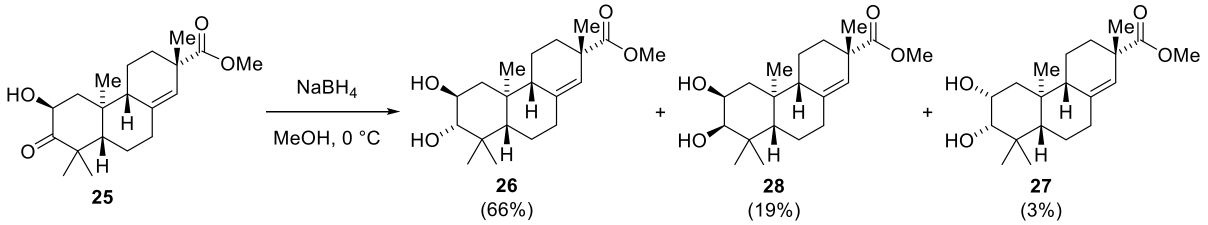

To a solution of α-hydroxy ketone **25** (32.6 mg, 97.5 μmol, 1 equiv) in methanol (4.00 mL) was added sodium borohydride (17.9 mg, 473 μmol, 4.85 equiv) at 0 °C. The colorless solution was stirred at 0 °C for 50 min. Next, the reaction mixture was diluted with dichloromethane (40 mL). Water (5 mL) and a saturated aqueous solution of ammonium chloride (10 mL) were added cautiously in succession at 0 °C. The organic layer was separated, and the aqueous layer was extracted with dichloromethane (3 × 10 mL). The combined organic layers were dried over sodium sulfate, the dried organic layer was filtered, and the filtrate was concentrated under reduced pressure. The residue was purified by semipreparative normal-phase high performance liquid chromatography (HPLC) (35% grading to 50% ethyl acetate in *n*-hexane over 30 min) to yield *trans*-diol **26** (21.7 mg, 64.5 μmol, 66%) as a viscous colorless oil, *cis*-diol **28** (6.2 mg, 18 μmol, 19%) as a viscous colorless oil, and *cis*-diol **27** (1.1 mg, 3.3 μmol, 3%) as an amorphous white solid.

<u>Analytical data of *trans*-diol **26**:</u>

TLC (50% ethyl acetate in cyclohexane): R_f = 0.21 (CAM).

$[\alpha]_D^{20}$ = –52.3 (*c* = 1.09, dichloromethane).

^{1}H NMR (400 MHz, $CDCl_3$): δ 5.40 (q, *J* = 1.8 Hz, 1H), 3.69 – 3.58 (m, 4H), 3.03 (d, *J* = 9.5 Hz, 1H), 2.34 (ddd, *J* = 14.3, 4.6, 2.1 Hz, 1H), 2.27 (s, 2H), 2.17 (dtd, *J* = 12.8, 3.6, 1.4 Hz, 1H), 2.02 (tdt, *J* = 13.7, 5.3, 1.7 Hz, 1H), 1.95 (dd, *J* = 12.4, 4.2 Hz, 1H), 1.76 (t, *J* = 8.5 Hz, 1H), 1.62 (dddt, *J* = 11.6, 8.2, 5.4, 3.0 Hz, 2H), 1.49 – 1.32 (m, 2H), 1.19 (s, 3H), 1.19 – 1.07 (m, 3H), 1.03 (s, 3H), 0.84 (s, 3H), 0.75 (s, 3H).

^{13}C NMR (101 MHz, $CDCl_3$): δ 177.6, 138.4, 126.3, 83.8, 68.8, 54.2, 51.9, 50.6, 45.1, 43.2, 39.4, 39.2, 35.5, 33.4, 29.1, 27.6, 22.2, 20.3, 17.0, 15.3.

IR (ATR, neat): $\tilde{\nu}$ = 3381 (br), 2943 (s), 2873 (m), 1730 (s), 1455 (m), 1433 (m), 1386 (w), 1367 (w), 1267 (m), 1196 (s), 1160 (m), 1141 (m), 1121 (s), 1093 (m), 1054 (s), 994 (m), 966 (m), 937 (w), 917 (w), 888 (w), 866 (w), 849 (w), 796 (w), 769 (w), 733 (m), 651 (w), 594 (w), 472 (w) cm^{-1}.

HRMS (ESI): calcd for $C_{20}H_{32}NaO_4^+$ $[M+Na]^+$: 359.2193; found: 359.2188.

<u>Analytical data of *cis*-diol **28**:</u>

TLC (50% ethyl acetate in cyclohexane): R_f = 0.28 (CAM).

$[\alpha]_D^{20}$ = –58 (*c* = 0.36, dichloromethane).

^{1}H NMR (400 MHz, $CDCl_3$): δ 5.39 (q, *J* = 1.9 Hz, 1H), 4.02 – 3.92 (m, 1H), 3.64 (s, 3H), 3.44 (s, 1H), 2.33 (ddd, *J* = 14.1, 4.4, 2.0 Hz, 1H), 2.17 (dtd, *J* = 12.8, 3.6, 1.4 Hz, 1H), 2.11 – 1.98 (m, 2H), 1.93 – 1.86 (br, 1H), 1.84 (t, *J* = 8.6 Hz, 1H), 1.71 – 1.56 (m, 2H), 1.58 – 1.31 (m, 5H), 1.19 (s, 3H), 1.13 (td, *J* = 13.4, 3.4 Hz, 1H), 1.03 (s, 3H), 0.88 (s, 3H), 0.73 (s, 3H).

^{13}C NMR (101 MHz, $CDCl_3$): δ 177.7, 138.9, 126.1, 79.1, 66.8, 51.9, 50.5, 47.4, 43.2, 40.2, 39.4, 38.5, 35.6, 33.5, 28.7, 27.7, 22.2, 21.9, 20.2, 15.2.

IR (ATR, neat): $\tilde{\nu}$ = 3403 (br), 2942 (s), 2872 (m), 1731 (s), 1454 (m), 1433 (m), 1387 (m), 1266 (m), 1197 (m), 1160 (m), 1127 (s), 1089 (w), 1042 (m), 987 (w), 968 (w), 948 (w), 925 (w), 885 (w), 854 (w), 802 (w), 768 (w), 734 (w), 698 (w), 674 (w), 617 (w), 587 (w), 511 (w) cm^{-1}.

HRMS (ESI): calcd for $C_{20}H_{32}NaO_4^+$ $[M+Na]^+$: 359.2193; found: 359.2189.

Analytical data of *cis*-diol **27**:

TLC (50% ethyl acetate in cyclohexane): R_f = 034 (CAM).

$[\alpha]_D^{20}$ = –39 (c = 0.081, dichloromethane).

^{1}H NMR (400 MHz, $CDCl_3$): δ 5.41 (q, *J* = 1.7 Hz, 1H), 4.11 (p, *J* = 2.9 Hz, 1H), 3.64 (s, 3H), 3.21 (dd, *J* = 7.3, 3.7 Hz, 1H), 2.36 (ddd, *J* = 14.2, 4.6, 2.1 Hz, 1H), 2.18 (dtd, *J* = 12.8, 3.9, 2.7, 1.1 Hz, 1H), 2.11 – 1.97 (m, 3H), 1.85 (d, *J* = 2.7 Hz, 1H), 1.70 – 1.59 (m, 3H), 1.59 – 1.39 (m, 2H), 1.35 (dd, *J* = 14.6, 3.4 Hz, 1H), 1.19 (s, 3H), 1.16 – 1.08 (m, 2H), 1.03 (s, 3H), 1.02 (s, 3H), 0.92 (s, 3H).

^{13}C NMR (101 MHz, $CDCl_3$): δ 177.7, 138.7, 126.3, 78.5, 71.4, 54.2, 51.8, 51.5, 43.2, 42.9, 38.5, 37.5, 35.7, 33.5, 30.3, 27.7, 22.1, 20.2, 17.5, 16.0.

IR (ATR, neat): $\tilde{\nu}$ = 3468 (br), 2933 (s), 2871 (m), 1729 (s), 1456 (m), 1433 (m), 1364 (m), 1252 (m), 1203 (m), 1159 (s), 1140 (s), 1123 (s), 1069 (m), 1030 (m), 992 (m), 969 (w), 936 (w), 851 (m), 775 (w), 752 (w), 701 (w), 620 (w) cm^{-1}.

HRMS (ESI): calcd for $C_{20}H_{32}NaO_4^+$ $[M+Na]^+$: 359.2193; found: 359.2188.

2.2 Synthesis of "*ent*"-Corey–Noe–Lin ligand (**16**)

2.2.1 2,3-dihydrobenzo[*g*]phthalazine-1,4-dione (**S5**)

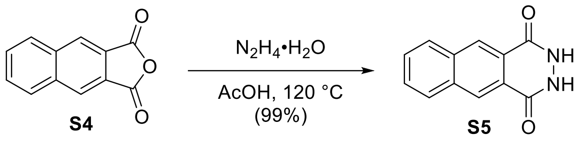

2,3-dihydrobenzo[*g*]phthalazine-1,4-dione (**S5**) was prepared according to a modified literature procedure[10]: To a suspension of naphtho[2,3-*c*]furan-1,3-dione (**S4**) (7.5 g, 95 wt%, 36 mmol, 1 equiv) in acetic acid (38 mL) was added hydrazine hydrate (5.8 mL, 72 mmol, 2.0 equiv) at 22 °C. The reaction mixture was heated to 120 °C for 4 h, during which a beige colored precipitate formed. Next, the reaction mixture was filtered, and the filter cake was washed consecutively with methanol (15 mL), water (45 mL) and methanol (15 mL). The filter cake was dried in a desiccator over potassium hydroxide under high vacuum for three days to yield 2,3-dihydrobenzo[*g*]phthalazine-1,4-dione (**S5**) (7.54 g, 35.5 mmol, 99%) as a beige solid.

The obtained analytical data were in accordance with reported literature values.[11]

2.2.2 1,4-dichlorobenzo[*g*]phthalazine (**S6**)

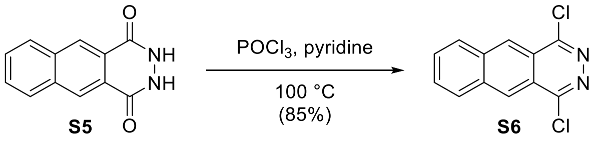

1,4-dichlorobenzo[*g*]phthalazine (**S6**) was prepared according to a modified literature procedure[12]:To a suspension of 2,3-dihydrobenzo[*g*]phthalazine-1,4-dione (**S5**) (5.07 g, 23.9 mmol, 1 equiv) in phosphoryl chloride (44.4 mL, 478 mmol, 20.0 equiv) was added pyridine (3.9 mL, 48 mmol, 2.0 equiv) at 22 °C. Heating to 100 °C resulted in the formation of a yellow solution, followed shortly thereafter by precipitation of a yellow solid. After stirring for 2.5 h at 100 °C, the reaction mixture was cooled to 65 °C and remaining phosphoryl chloride was removed by distillation. The residue was suspended in diethyl ether (50 mL) and filtered. The filter cake was washed with diethyl ether (250 mL) and then vigorously stirred in a 1:1 mixture of ice-cold water (400 mL) and ethyl acetate (400 mL) for 10 min. The suspension was filtered and the solid was washed successively with water (25 mL) and diethyl ether (25 mL). Drying of the solid in a

desiccator over phosphorus pentoxide under high vacuum for three days yielded 1,4-dichlorobenzo[*g*]phthalazine (**S6**) (5.94 g, 20.3 mmol, 85%) as a yellow solid.

The obtained analytical data were in accordance with reported literature values.[12]

2.2.3 *O*6'-(4-Heptyl)hydrocupreine (**S8**)

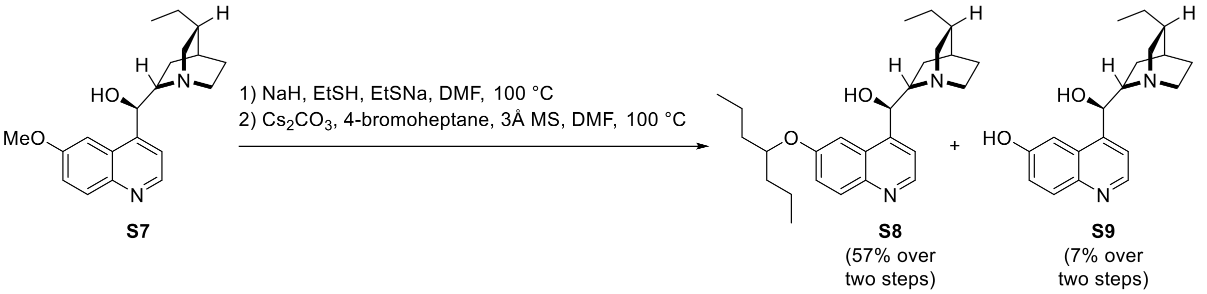

*O*6'-(4-heptyl)hydrocupreidine (**S8**) was prepared according to modified literature procedures[4c,13]: To a suspension of sodium hydride (14 g, 60 wt% in mineral oil, 0.16 mol, 8.0 equiv) in *N,N*-dimethylformamide (220 mL) was added dropwise ethanethiol (25.7 mL, 356 mmol, 8.00 equiv) at 22 °C. After ceasing of the gas evolution, hydroquinine (**S7**) (15.0 g, 97 wt%, 44.5 mmol, 1 equiv) was added and the reaction mixture was heated at 100 °C for 16 h, before it was cooled down to 22 °C and sodium ethanethiolate (4.3 g, 90 wt%, 46 mmol, 1.0 equiv) was added to the dark red solution. After stirring for 6 h at 100 °C, the reaction mixture was cooled to 22 °C and additional sodium ethanethiolate (2.2 g, 90 wt%, 23 mmol, 0.52 equiv) was added. After stirring for additional 18 h at 100 °C, the reaction mixture was cooled to 22 °C and additional sodium ethanethiolate (2.3 g, 90 wt%, 24 mmol, 0.55 equiv) was added to the reaction mixture followed by stirring at 100 °C for 28 h. After cooling to 22 °C, a saturated aqueous solution of ammonium chloride (300 mL) and ethyl acetate (700 mL) were added to the deep red reaction mixture. The organic layer was separated, and the aqueous layer was extracted with ethyl acetate (3 × 300 mL). The combined organic layers were dried over magnesium sulfate, the dried solution was filtered, and the filtrate was concentrated under reduced pressure (bath temperature: 80 °C). To the residue was added *n*-heptane (70 mL) and the mixture was concentrated under reduced pressure (bath temperature: 80 °C). To the remaining yellow-orange solid was added successively cesium carbonate (36.3 g, 111 mmol, 2.50 equiv), freshly activated 3Å molecular sieves (14.2 g), *N,N*-dimethylformamide (417 mL) and 4-bromoheptane (21.0 mL, 134 mmol, 3.00 equiv). The orange-brownish reaction mixture was stirred at 100 °C for 70 h, during which the color changed to deep red. The deep red suspension was filtered through celite and concentrated under reduced pressure (bath temperature:

80 °C). To remove remaining *N,N*-dimethylformamide, *n*-heptane (60 mL) was added to the residue and the mixture was concentrated under reduced pressure (bath temperature: 80 °C). This drying procedure was performed twice. The remaining solid was dissolved in ethyl acetate (400 mL) and the organic layer was washed with a 1 M aqueous solution of potassium hydroxide (5 × 200 mL), which were pooled and kept for isolation of hydrocupreine (**S9**). The organic layer was washed with a saturated aqueous solution of sodium chloride (100 mL), the washed organic layer was dried over magnesium sulfate, the dried solution was filtered, and the filtrate was concentrated under reduced pressure. The residue was purified by flash column chromatography on silica gel (concentrated aqueous ammonia : methanol : chloroform (NH_4OH : MeOH : $CHCl_3$), 1:3:96 grading to 1:9:90) to yield title compound **S8** (10.38 g, 25.28 mmol, 57%) as a beige solid.

Recovered hydrocupreine (**S9**): The pooled 1 M potassium hydroxide washing solutions were neutralized with ammonium chloride and extracted with ethyl acetate (4 × 175 mL). The combined organic layers were dried over magnesium sulfate, the dried solution was filtered, and the filtrate was concentrated under reduced pressure to give hydrocupreine (**S9**) (0.95 g, 3.1 mmol, 7%) as a yellowish-brown foam.

The obtained analytical data for **S9** were in accordance with reported literature values.[13]

Analytical data of *O*6'-(4-heptyl)hydrocupreidine (**S8**):

TLC (1% concentrated aqueous ammonia and 9% methanol in dichloromethane): R_f = 0.31 (UV).

mp: 55–56 °C.

$[\alpha]_D^{20}$ = –61.7 (*c* = 1.03, dichloromethane).

^{1}H NMR (400 MHz, $CDCl_3$): δ 8.32 (d, *J* = 4.6 Hz, 1H), 7.78 (d, *J* = 9.2 Hz, 1H), 7.39 (d, *J* = 4.5 Hz, 1H), 7.32 (d, *J* = 2.7 Hz, 1H), 7.19 (dd, *J* = 9.2, 2.6 Hz, 1H), 5.94 (s, 1H), 5.42 (d, *J* = 4.0 Hz, 1H), 4.43 (p, *J* = 5.6 Hz, 1H), 3.43 (t, *J* = 13.2 Hz, 1H), 3.01 (dd, *J* = 11.9, 8.2 Hz, 1H), 2.95 (dd, *J* = 13.5, 9.9 Hz, 1H), 2.52 (ddd, *J* = 14.5, 10.6, 4.5 Hz, 1H), 2.27 (ddd, *J* = 13.5, 4.7, 2.3 Hz, 1H), 1.74 – 1.53 (m, 7H), 1.52 – 1.40 (m, 2H), 1.40 – 1.27 (m, 5H), 1.23 – 1.09 (m, 2H), 0.92 – 0.82 (m, 6H), 0.74 (t, *J* = 7.3 Hz, 3H).

^{13}C NMR (101 MHz, $CDCl_3$): δ 156.7, 148.7, 147.1, 143.7, 131.1, 126.8, 122.4, 118.6, 104.0, 77.7, 72.1, 60.0, 58.8, 43.4, 37.6, 36.4, 36.1, 28.4, 27.8, 25.6, 21.5, 18.8, 18.5, 14.3, 14.3, 12.1.

IR (ATR, neat): $\tilde{\nu}$ = 3074 (w), 2957 (s), 2931 (s), 2871 (m), 1618 (s), 1589 (m), 1506 (s), 1456 (s), 1379 (m), 1360 (m), 1329 (m), 1306 (w), 1239 (s), 1223 (s), 1204 (m), 1193 (m), 1169 (w), 1117 (s), 1086 (m), 1058 (m), 1043 (m), 991 (s), 936 (m) 882 (m), 853 (s), 826 (s), 760 (m), 643 (s), 621 (m), 574 (w), 522 (w), 495 (w), 461 (w), 436 (w) cm^{-1}.

HRMS (ESI): calcd for $C_{26}H_{39}N_2O_2^+$ $[M+H]^+$: 411.3006; found: 411.2997.

2.2.4 Dimer **S10**

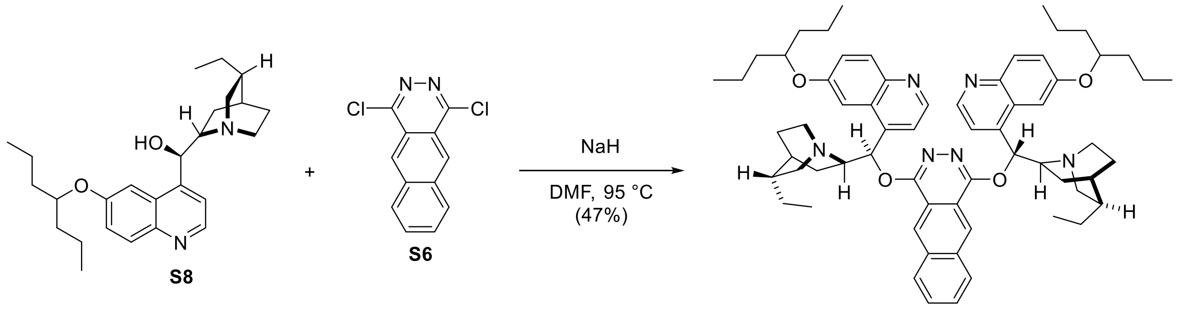

Dimer **S10** was prepared according to a modified literature procedure[14]: To a solution of *O*6'-(4-heptyl)hydrocupreine (**S8**) (10.02 g, 24.40 mmol, 1 equiv) in *N,N*-dimethylformamide (91 mL) was added sodium hydride (1.4 g, 60 wt% in mineral oil, 35 mmol, 1.5 equiv) at 22 °C. The brown suspension was stirred for 15 min at 22 °C, then 1,4-dichlorobenzo[*g*]phthalazine (**S6**) (4.26 g, 17.1 mmol, 0.701 equiv) was added at 22 °C, whereupon the reaction mixture turned deep red. The reaction mixture was then heated to 70 °C for 4 h, before additional sodium hydride (0.52 g, 60 wt% in mineral oil, 13 mmol, 0.53 equiv) was added and stirring was continued at 95 °C for 3 h until TLC indicated complete consumption of 1,4-dichlorobenzo[*g*]phthalazine (**S6**). Next, the reaction mixture was allowed to cool to 22 °C, water (300 mL) was added, the organic layer was separated, and the aqueous layer was extracted with ethyl acetate (6 × 300 mL). The combined organic layers were dried over sodium sulfate, the dried organic layer was filtered, and the filtrate was concentrated under reduced pressure. The residue was purified by flash column chromatography on silica gel (concentrated aqueous ammonia : methanol : dichloromethane (NH_4OH : MeOH : CH_2Cl_2) 1:4:95 grading to 1:9:90) to yield title compound **S10** (5.78 g, 5.80 mmol, 47%) as a beige solid.

<u>Analytical data of dimer **S10**:</u>

TLC (1% concentrated aqueous ammonia and 9% methanol in dichloromethane): R_f = 0.48 (UV).

mp: 126–127 °C.

$[\alpha]_D^{20}$ = +341 (c = 1.07, dichloromethane).

^{1}H NMR (400 MHz, $CDCl_3$): δ 8.90 (s, 2H), 8.63 (d, J = 4.5 Hz, 2H), 8.22 (dd, J = 6.4, 3.3 Hz, 2H), 7.98 (d, J = 9.2 Hz, 2H), 7.74 (dd, J = 6.5, 3.3 Hz, 2H), 7.65 (d, J = 2.7 Hz, 2H), 7.48 (d, J = 4.5 Hz, 2H), 7.32 (dd, J = 9.2, 2.6 Hz, 2H), 7.01 (d, J = 5.7 Hz, 2H), 4.49 (p, J = 5.8 Hz, 2H), 3.58 (q, J = 7.7, 7.1 Hz, 2H), 3.25 – 3.15 (m, 2H), 3.03 (dd, J = 13.6, 9.5 Hz, 2H), 2.56 (ddd, J = 14.7, 10.8, 4.7 Hz, 2H), 2.33 (d, J = 14.0 Hz, 2H), 1.93 – 1.60 (m, 16H), 1.58 – 1.25 (m, 16H), 0.90 (t, J = 7.3 Hz, 6H), 0.87 – 0.80 (m, 12H).

^{13}C NMR (101 MHz, $CDCl_3$): δ 156.8, 156.3, 147.3, 144.7, 144.3, 134.7, 131.8, 129.3, 128.5, 127.1, 123.2, 122.8, 119.9, 119.2, 104.7, 78.0, 77.8, 60.0, 58.9, 42.8, 37.6, 36.3, 36.1, 28.8, 27.9, 25.7, 23.6, 18.9, 18.6, 14.3, 14.3, 12.2.

IR (ATR, neat): $\tilde{\nu}$ = 3070 (w), 2956 (m), 2931 (m), 2871 (m), 1664 (w), 1618 (m), 1592 (w), 1571 (w), 1542 (w), 1505 (m), 1459 (s), 1433 (s), 1375 (s), 1346 (s), 1306 (m), 1239 (s), 1223 (s), 1215 (s), 1202 (s), 1145 (m), 1131 (m), 1119 (m), 1079 (m), 1059 (m), 986 (m), 948 (m), 927 (m), 850 (s), 831 (s), 792 (w), 673 (m), 644 (s), 613 (m), 592 (w), 533 (w), 512 (w), 474 (m), 438 (w) cm^{-1}.

HRMS (ESI): calcd for $C_{64}H_{81}N_6O_4^+$ $[M+H]^+$: 997.6314; found: 997.6299.

2.2.5 "*Ent*"-Corey–Noe–Lin ligand (**16**)

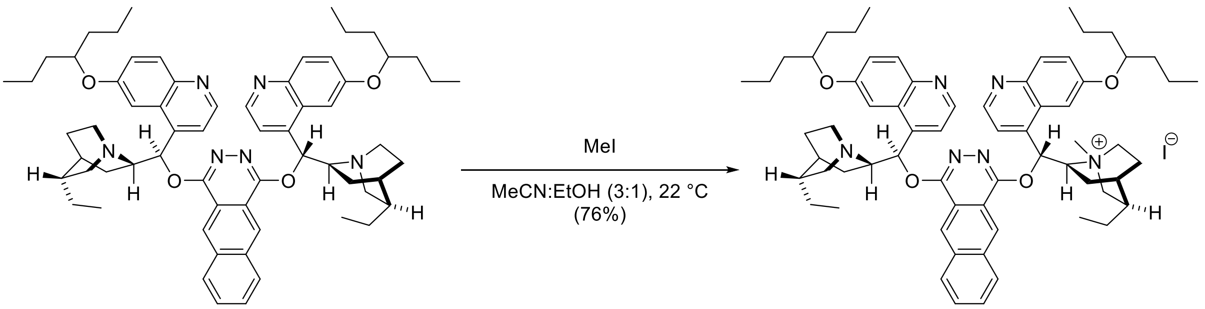

S10 "*ent*"-Corey–Noe–Lin ligand (**16**)

"*Ent*"-Corey–Noe–Lin ligand (**16**) was prepared according to a modified literature procedure[4c]: To a solution of dimer **S10** (3.75 g, 3.76 mmol, 2.99 equiv) in a mixture of acetonitrile (18.8 mL) and ethanol (6.3 mL) was added methyl iodide (178.6 mg, 1.258 mmol, 1 equiv) at 22 °C. After stirring

for 14 h at 22 °C, the reaction mixture was concentrated under reduced pressure. The residue was purified by flash column chromatography on silica gel (concentrated aqueous ammonia : methanol : dichloromethane (NH_4OH : MeOH : CH_2Cl_2), 1:4:95 grading to 1:6:93) to yield the "*ent*"-Corey–Noe–Lin ligand (**16**) (1.09 g, 958 μmol, 76%) as a beige solid and recovered dimer **S10** (2.50 g, 2.51 mmol, 67% recovery).

Analytical data of "*ent*"-Corey–Noe–Lin ligand (**16**):

TLC (1% concentrated aqueous ammonia and 9% methanol in dichloromethane): R_f = 0.30 (UV).

mp: Decomposition at 224 °C before melting.

$[\alpha]_D^{20}$ = +243 (*c* = 0.976, dichloromethane).

^{1}H NMR (700 MHz, $CDCl_3$, 45 °C): δ 9.02 (s, 1H), 8.90 (s, 1H), 8.68 (d, *J* = 4.6 Hz, 1H), 8.63 (d, *J* = 4.5 Hz, 1H), 8.37 (d, *J* = 8.4 Hz, 1H), 8.28 (d, *J* = 8.2 Hz, 1H), 8.09 (d, *J* = 9.2 Hz, 1H), 7.98 (d, *J* = 9.2 Hz, 1H), 7.85 – 7.80 (m, 3H), 7.63 (d, *J* = 4.6 Hz, 1H), 7.53 (s, 1H), 7.45 – 7.42 (m, 2H), 7.32 (dd, *J* = 9.2, 2.6 Hz, 1H), 7.13 (d, *J* = 2.6 Hz, 1H), 6.95 (br, 1H), 4.97 (dd, *J* = 12.6, 10.6 Hz, 1H), 4.56 (td, *J* = 12.0, 11.5, 4.7 Hz, 1H), 4.51 – 4.40 (m, 1H), 4.42 (p, *J* = 5.8 Hz, 1H), 3.82 – 3.74 (m, 2H), 3.54 (br, 1H), 3.49 (s, 3H), 3.18 (br, 1H), 3.03 (t, *J* = 9.2 Hz, 1H), 2.93 (d, *J* = 12.6 Hz, 1H), 2.66 (dd, *J* = 14.1, 8.2 Hz, 1H), 2.56 (br, 1H), 2.40 – 2.32 (m, 3H), 2.31 – 2.25 (m, 2H), 1.95 (br, 1H), 1.86 (br, 1H), 1.83 – 1.59 (m, 11H), 1.51 – 1.40 (m, 6H), 1.40 – 1.21 (m, 8H), 0.90 (t, *J* = 7.4 Hz, 3H), 0.88 (t, *J* = 7.5, 7.1 Hz, 3H), 0.86 – 0.81 (m, 12H).

^{13}C NMR (176 MHz, $CDCl_3$, 45 °C): δ 158.0, 157.7, 157.1, 154.8, 147.5 (2C), 144.9, 144.7, 143.8, 138.0, 135.4, 135.2, 133.1, 132.1, 129.6, 129.5, 129.5, 129.4, 126.9, 125.8, 124.3, 122.8, 122.4, 122.2, 119.8, 119.2, 118.9, 118.6, 104.7, 103.6, 78.7 (2C), 78.2, 69.1, 69.0, 68.3, 60.1, 59.1, 55.1, 50.0, 43.2, 37.8, 36.4, 36.2 (3C), 35.9, 29.1, 27.9, 27.0, 26.2, 25.9, 24.0, 23.2, 22.2, 18.9, 18.9, 18.7, 18.6, 14.3, 14.3, 14.3 (2C), 12.1, 11.4.

IR (ATR, neat): $\tilde{\nu}$ = 2955 (s), 2931 (m), 2871 (m), 1667 (w), 1618 (m), 1592 (w), 1569 (w), 1543 (w), 1505 (m), 1460 (s), 1434 (s), 1377 (s), 1346 (s), 1306 (m), 1239 (m), 1220 (s), 1199 (m), 1145 (m), 1130 (m), 1120 (m), 1082 (m), 1067 (m), 991 (m), 947 (m), 930 (w), 898 (w), 852 (m), 827 (m), 754 (m), 733 (w), 673 (w), 642 (w), 614 (w), 595 (w), 476 (w), 436 (w) cm^{-1}.

HRMS (ESI): calcd for $C_{65}H_{83}N_6O_4^+$ $[M–I^-]^+$: 1011.6470; found: 1011.6451.

2.3 Racemic access to epoxide **12**

2.3.1 Epoxide ***rac*-12**

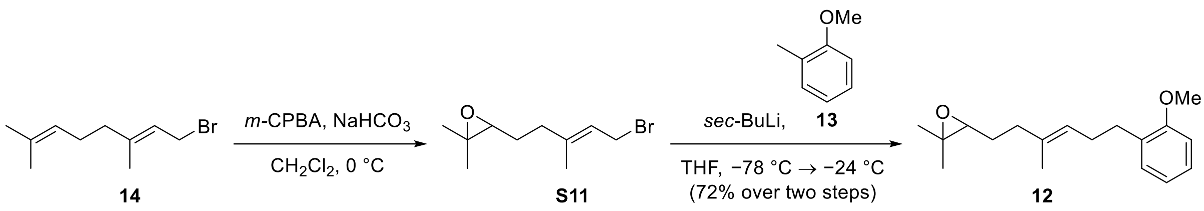

Epoxygeranyl bromide (**S11**) was prepared in analogy to known literature procedure[15]: To a solution of geranyl bromide (**14**) (39.6 g, 183 mmol, 1 equiv) in dichloromethane (910 mL) was added sequentially sodium bicarbonate (19.9 g, 237 mmol, 1.30 equiv) and *meta*-chloroperoxybenzoic acid (*m*-CPBA) (45 g, 77 wt%, 0.20 mol, 1.1 equiv) portionwise at 0 °C over 5 min. After stirring for 40 min at 0 °C, water (600 mL) was added to the white suspension, the organic layer was separated and the aqueous layer was extracted with diethylether:*n*-pentane (1:1 v/v) (1 × 400 mL, 1 × 300 mL). The combined organic layers were washed with a saturated aqueous sodium bicarbonate solution (400 mL) followed by washing with a saturated aqueous sodium chloride solution (300 mL). The washed organic layer was dried over magnesium sulfate, the dried solution was filtered, and the filtrate was concentrated under reduced pressure. The crude product (42.8 g of a colorless liquid) was used in the next step without further purification.

To a solution of 2-methyl anisole (**13**) (18.6 g, 152 mmol, 1.56 equiv) in tetrahydrofuran (564 mL) was added *sec*-butyllithium (1.40 M in cyclohexane, 138 mL, 193 mmol, 1.98 equiv) at –78 °C over 20 min, during which the colorless solution turned deep yellow. After complete addition, the reaction mixture was allowed to warm up to –24 °C over 4 h 45 min. The deep yellow-orange reaction mixture was cooled again to –78 °C and crude epoxygeranyl bromide (**S11**) (22.7 g of crude product, assumed 97.3 mmol, 1 equiv) was added over 5 min resulting in partial decolorization. After 5 min at –78 °C, a saturated aqueous solution of ammonium chloride (300 mL) was added. The cooling bath was removed, the reaction mixture allowed to warm to 22 °C and additional water (150 mL) was added. The organic layer was separated, and the aqueous layer was extracted with diethyl ether (3 × 250 mL). The combined organic layers were washed with a saturated aqueous solution of sodium chloride (250 mL), dried over magnesium sulfate, the dried solution was filtered, and the filtrate was concentrated under reduced pressure. The residue was purified by flash column chromatography on silica gel (10% diethyl ether in *n*-pentane) to yield the title compound ***rac*-12** (19.2 g, 69.9 mmol, 72% over two steps) as a colorless oil.

See analytical data in the section for enantioselective preparation of epoxide **12**.

2.5 Syntheses of *ent*-pimarane natural products

2.5.1 (3*R*,5*S*,9*S*,10*S*,13*S*)-2-hydroxy-16-nor-*ent*-pimar-8(14)-en-15-oic acid (HPA, 1)

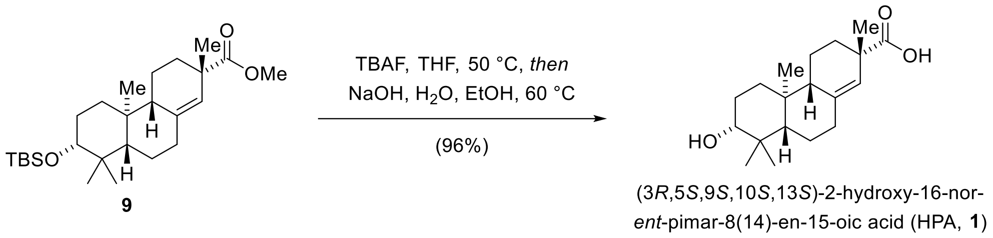

To a solution of alkene **9** (25.7 mg, 59.1 µmol, 1 equiv) in tetrahydrofuran (400 µL) was added a solution of tetrabutylammonium fluoride (TBAF) in tetrahydrofuran (1.00 M, 177 µL, 177 µmol, 3.00 equiv) at 22 °C. The reaction mixture was stirred at 50 °C for 27 h, after which the reaction mixture was cooled to 22 °C and water (300 µL), ethanol (300 µL) and sodium hydroxide (33.1 mg, 828 µmol, 14.0 equiv) were added successively at 22 °C. Stirring was continued at 60 °C. After 4 h, a 1 M aqueous solution of hydrogen chloride (5 mL) was added, and the mixture was extracted with dichloromethane (4 × 10 mL). The combined organic layers were washed with a 1 M aqueous solution of hydrogen chloride (3 × 5 mL), the washed solution was dried over sodium sulfate, the dried organic layer was filtered, and the filtrate was concentrated under reduced pressure. The residue was purified by flash column chromatography on silica gel (5% methanol in dichloromethane) to yield (3*R*,5*S*,9*S*,10*S*,13*S*)-2-hydroxy-16-nor-*ent*-pimar-8(14)-en-15-oic acid (HPA, **1**) (17.4 mg, 56.8 µmol, 96%) as a white solid.

<u>Analytical data of (3*R*,5*S*,9*S*,10*S*,13*S*)-2-hydroxy-16-nor-*ent*-pimar-8(14)-en-15-oic acid (HPA, **1**):</u>

TLC (5% methanol in dichloromethane): R_f = 0.28 (CAM).

mp: 120–121 °C.

$[\alpha]_D^{20}$ = –37.5 (*c* = 1.02, methanol).

$[\alpha]_D^{25}$ (literature)[16] = –14.6 (*c* = 0.1, methanol).

^{1}H NMR (400 MHz, CD_3OD): δ 5.39 (q, *J* = 1.8 Hz, 1H), 3.23 – 3.16 (m, 1H), 2.33 (ddd, *J* = 14.2, 4.6, 2.0 Hz, 1H), 2.14 (dt, *J* = 12.4, 3.3 Hz, 1H), 2.05 (td, *J* = 13.6, 5.6 Hz, 1H), 1.76 – 1.54 (m, 6H), 1.53 – 1.36 (m, 2H), 1.24 – 1.15 (m, 1H), 1.18 (s, 3H), 1.11 (td, *J* = 13.3, 3.5 Hz, 1H), 1.08 (dd, *J* = 12.4, 2.5 Hz, 1H), 0.99 (s, 3H), 0.81 (s, 3H), 0.73 (s, 3H).

^{13}C NMR (101 MHz, CD_3OD): δ 180.9, 140.0, 127.1, 79.7, 55.5, 51.9, 43.9, 40.1, 39.3, 38.4, 36.7, 34.3, 29.0, 28.2 (2C), 23.4, 21.3, 16.4, 14.9.

IR (ATR, neat): $\tilde{\nu}$ = 3398 (br), 2938 (s), 2872 (m), 2851 (m), 1698 (s), 1455 (m), 1385 (w), 1366 (w), 1282 (m), 1244 (m), 1202 (m), 1161 (m), 1136 (m), 1087 (m), 1030 (m), 1000 (w), 971 (w), 932 (w), 865 (w), 848 (w), 746 (w), 635 (w), 605 (w) cm^{-1}.

HRMS (ESI): calcd for $C_{19}H_{30}NaO_3^+$ $[M+Na]^+$: 329.2087; found: 329.2079.

NMR comparison for (3*R*,5*S*,9*S*,10*S*,13*S*)-2-hydroxy-16-nor-*ent*-pimar-8(14)-en-15-oic acid (HPA, **1**):

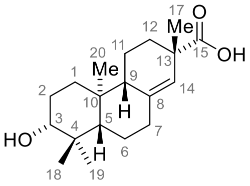

Atom number	Natural **1**[16] (400 MHz, CD_3OD) δ_H [ppm]	Synthetic **1** (400 MHz, CD_3OD) δ_H [ppm]	$\lvert\Delta\delta_H\rvert$ [ppm]
1α	1.69 (m, 1H)	1.68 (m, 1H)	0.01
1β	1.19 (m, 1H)	1.20 (m, 1H)	–0.01
2α	1.61 (m, 1H)	1.63 – 1.54 (m, 2H)	—
2β	1.59 (m, 1H)		—
3	3.20 (dd, *J* = 10.2, 6.2 Hz, 1H)	3.20 (m, 1H)	0.00
5	1.08 (dd, *J* = 12.2, 2.2 Hz, 1H)	1.08 (dd, *J* = 12.4, 2.5 Hz, 1H)	0.00
6α	1.42 (dd, *J* = 12.8, 4.2 Hz, 1H)	1.43 (m, 1H)	–0.01
6β	1.64 (m, 1H)	1.64 (m, 1H)	0.00
7α	2.34 (dd, *J* = 14.1, 2.4 Hz, 1H)	2.33 (ddd, *J* = 14.2, 4.6, 2.0 Hz, 1H)	0.01
7β	2.05 (td, *J* = 13.4, 5.1 Hz, 1H)	2.05 (td, *J* = 13.6, 5.6 Hz, 1H)	0.00
9	1.71 (m, 1H)	1.72 (m, 1H)	–0.01
11α	1.48 (m, 1H)	1.47 (m, 1H)	0.01
11β	1.62 (m, 1H)	1.62 (m, 1H)	0.00
12α	1.08 (dd, *J* = 12.2, 2.2 Hz, 1H)	1.08 (td, *J* = 13.1, 12.5, 2.7 Hz, 1H)	0.00
12β	2.16 (d, *J* = 11.2 Hz, 1H)	2.14 (dt, *J* = 12.4, 3.3 Hz, 1H)	0.02
14	5.43 (s, 1H)	5.39 (q, *J* = 1.8 Hz, 1H)	0.04
17	1.17 (s, 3H)	1.18 (s, 3H)	–0.01
18	1.00 (s, 3H)	0.99 (s, 3H)	0.01
19	0.81 (s, 3H)	0.81 (s, 3H)	0.00
20	0.75 (s, 3H)	0.73 (s, 3H)	0.02

Atom number	Natural **1**[16] (101 MHz, CD_3OD) δ_C [ppm]	Synthetic **1** (101 MHz, CD_3OD) δ_C [ppm]	$\lvert\Delta\delta_C\rvert$ [ppm]
1	38.7	38.4	0.3
2	28.4	28.2	0.2
3	79.9	79.7	0.2
4	40.2	40.1	0.1
5	55.7	55.5	0.2
6	23.5	23.4	0.1
7	36.9	36.7	0.2
8	139.1	140.0	–0.9
9	52.3	51.9	0.4
10	39.5	39.3	0.2
11	21.7	21.3	0.4
12	34.9	34.3	0.6
13	44.8	43.9	0.9
14	128.4	127.1	1.3
15	181.3	180.9	0.4
17	28.7	28.2	0.5
18	29.2	29.0	0.2
19	16.6	16.4	0.2
20	15.3	14.9	0.4

A systematic shift of approximately 0.2 ppm between natural **1** and synthetic **1** is observed in the ^{13}C NMR spectrum, which might be due to a different CD_3OD resonance reference in the literature. Larger deviations might originate from a pH- and thus also concentration-dependence of the ^{13}C NMR spectrum. The ^{1}H NMR data are in good agreement with the literature.

2.5.2 Norflickinflimiod C (**5**)

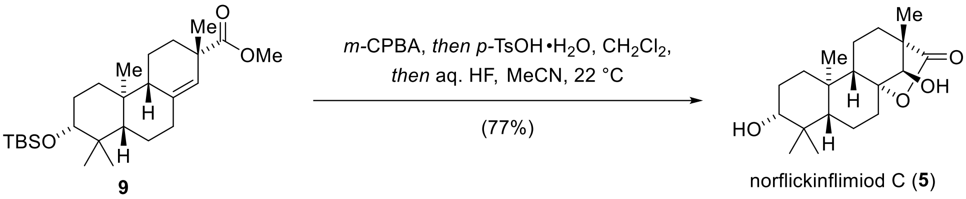

To a solution of alkene **9** (100.1 mg, 230.3 µmol, 1 equiv) in dichloromethane (5.00 mL) was added *meta*-chloroperoxybenzoic acid (*m*-CPBA) (108 mg, 77.0 wt%, 480 µmol, 2.08 equiv) at 22 °C. After stirring for 1 h 40 min at 22 °C, *para*-toluenesulfonic acid monohydrate (40.2 mg, 211 µmol, 0.918 equiv) was added at 22 °C. After 30 min, acetonitrile (5.00 mL) and an aqueous solution of hydrogen fluoride (834 µL, 48.0 wt%, 23.0 mmol, 100 equiv) were added at 22 °C successively. After stirring for additional 10 min at 22 °C, the reaction mixture was diluted with ethyl acetate (70 mL) and successively washed with a 1 M aqueous solution of sodium hydroxide (1 × 40 mL, 2 × 20 mL), a saturated aqueous solution of ammonium chloride (20 mL) and a saturated aqueous solution of sodium chloride (20 mL). The washed solution was dried over sodium sulfate, the dried organic layer was filtered, and the filtrate was concentrated under reduced pressure. The residue was purified by flash column chromatography on silica gel (3% methanol in dichloromethane) to yield norflickinflimiod C (**5**) (57.0 mg, 117 µmol, 77%) as a white solid.

Analytical data of norflickinflimiod C (**5**):

TLC (50% ethyl acetate in cyclohexane): R_f = 0.24 (CAM).

mp: 213–214 °C.

$[\alpha]_D^{20}$ = +3.4 (c = 0.22, chloroform).

$[\alpha]_D^{20}$ (literature)[17] = −13.3 (c = 0.1, chloroform).

^{1}H NMR (400 MHz, CD_3OD): δ 3.39 (s, 1H), 3.18 (dd, *J* = 10.3, 5.8 Hz, 1H), 1.92 – 1.80 (m, 2H), 1.76 (ddd, *J* = 14.8, 4.1, 2.5 Hz, 1H), 1.69 (dt, *J* = 12.9, 3.4 Hz, 1H), 1.66 – 1.53 (m, 6H), 1.34 – 1.24 (m, 2H), 1.09 (td, *J* = 12.6, 5.4 Hz, 1H), 1.06 (s, 3H), 1.01 (s, 3H), 0.97 (s, 3H), 0.89 (dd, *J* = 11.0, 3.3 Hz, 1H), 0.84 (s, 3H).

^{13}C NMR (101 MHz, CD_3OD): δ 181.1, 86.8, 79.5, 79.5, 55.1, 47.1, 46.1, 39.9, 39.1, 37.2, 35.0, 29.1, 27.7, 26.8, 19.6, 19.4, 18.7, 16.6, 15.6.

IR (ATR, neat): $\tilde{\nu}$ = 3376 (br), 2929 (m), 2872 (m), 1752 (s), 1649 (w), 1455 (m), 1378 (w), 1362 (m), 1333 (w), 1287 (w), 1255 (w), 1240 (w), 1215 (w), 1184 (w), 1156 (m), 1137 (m), 1117 (s), 1082 (m), 1030 (m), 1007 (w), 993 (m), 972 (m), 946 (s), 924 (m), 901 (w), 862 (w), 831 (w), 752 (w), 631 (w), 596 (w), 571 (w), 540 (w) cm^{-1}.

HRMS (ESI): calcd for $C_{19}H_{30}NaO_4^+$ [M+Na]$^+$: 345.2036; found: 345.2033.

ECD (MeCN, 25 °C, 400 μM): λ_{max} ($\Delta\varepsilon$): 221 (–1.51).

ECD (MeCN, 930 μM, literature)[17]: λ_{max} ($\Delta\varepsilon$): 221 (–0.31).

Comparison of the measured ECD spectrum with the ECD spectrum in ref 17 indicates that the same enantiomer has been prepared.

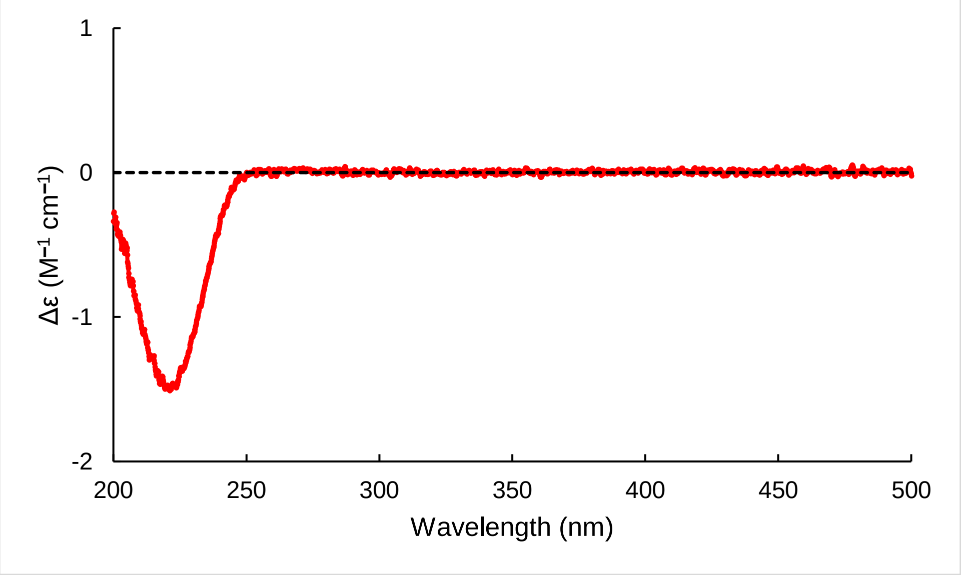

Figure 1. Experimental ECD spectrum of norflickinflimiod C (**5**).

NMR comparison for norflickinflimiod C (**5**):

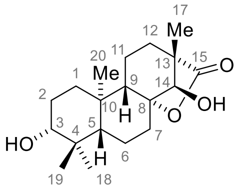

Atom number	Natural **5**[17] (400 MHz, CD_3OD) δ_H [ppm]	Synthetic **5** (400 MHz, CD_3OD) δ_H [ppm]	$\lvert\Delta\delta_H\rvert$ [ppm]
1α	1.69 (td, *J* = 13.0, 2.8 Hz, 1H)	1.69 (dt, *J* = 12.9, 3.4 Hz, 1H)	0.00
1β	1.10 (dd, *J* = 13.0, 5.6 Hz, 1H)	1.09 (td, *J* = 12.6, 5.4 Hz, 1H)	0.01
2α	1.62 (m, 1H)	1.64–1.54 (m, 2H)	—
2β	1.58 (m, 1H)		—
3	3.18 (dd, *J* = 10.4, 6.0 Hz, 1H)	3.18 (dd, *J* = 10.3, 5.8 Hz, 1H)	0.00
5	0.89 (m, 1H)	0.89 (dd, *J* = 11.0, 3.3 Hz, 1H)	0.00
6α	1.66 (m, 1H)	1.67–1.55 (m, 2H)	—
6β	1.62 (m, 1H)		—
7α	1.86 (m, 1H)	1.85 (m, 1H)	0.01
7β	1.78 (t, *J* = 2.4 Hz, 1H)	1.76 (ddd, *J* = 14.8, 4.1, 2.5 Hz, 1H)	0.02
9	1.58 (m, 1H)	1.58 (m, 1H)	0.00
11α	1.28 (m, 1H)	1.28 (m, 1H)	0.00
11β	**1.27 (m, 1H)***	1.67–1.55 (m, 1H)	—
12α	1.29 (m, 1H)	1.28 (m, 1H)	0.01
12β	**1.77 (m, 1H)****	1.85 (m, 1H)	–0.08
14	3.39 (s, br, 1H)	3.39 (s, 1H)	0.00
17	1.06 (s, 3H)	1.06 (s, 3H)	0.00
18	0.84 (s, 3H)	0.84 (s, 3H)	0.00
19	1.00 (s, 3H)	1.01 (s, 3H)	–0.01
20	0.97 (s, 3H)	0.97 (s, 3H)	0.00

*Presumably an assignment error

**Appears to be an error in writing in ref 17 upon comparison with the literature spectrum in the supporting information of ref 17

Atom number	Natural **5**[17] (101 MHz, CD_3OD) δ_C [ppm]	Synthetic **5** (101 MHz, CD_3OD) δ_C [ppm]	$\lvert\Delta\delta_C\rvert$ [ppm]
1	39.1	39.1	0.0
2	27.7	27.7	0.0
3	79.6	79.5	0.1
4	40.0	39.9	0.1
5	55.1	55.1	0.0
6	19.6	19.6	0.0
7	35.0	35.0	0.0
8	86.9	86.8	0.1
9	46.1	46.1	0.0
10	37.2	37.2	0.0
11	19.4	19.4	0.0
12	26.8	26.8	0.0
13	47.1	47.1	0.0
14	79.5	79.5	0.0
15	181.2	181.1	0.1
17	18.7	18.7	0.0
18	16.6	16.6	0.0
19	29.1	29.1	0.0
20	15.6	15.6	0.0

The ^1H NMR and ^{13}C NMR data are in good agreement with the literature.

2.5.3 3α,14β-diacetoxy-16-nor-*ent*-pimar-15α,8-olide (DAP, **30**)

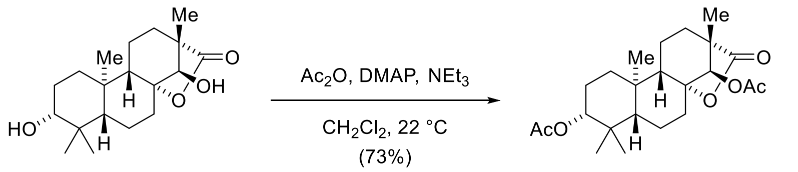

norflickinflimiod C (**5**) → 3α,14β-diacetoxy-16-nor-*ent*-pimar-15α,8-olide (DAP, **30**)

To a solution of norflickinflimiod C (**5**) (16.0 mg, 49.6 μmol, 1 equiv) and triethylamine (104 μL, 744 μmol, 15.0 equiv) in dichloromethane (3.50 mL) was added consecutively *N*,*N*-dimethylpyridin-4-amine (DMAP) (0.6 mg, 5 μmol, 0.1 equiv) and acetic anhydride (46.9 μL, 496 μmol, 10.0 equiv) at 22 °C. The resulting colorless solution was stirred at 22 °C for 15 h, after which the reaction mixture was diluted with diethyl ether (35 mL) and washed successively with a 1 M aqueous solution of hydrogen chloride (2 × 10 mL), a saturated aqueous solution of sodium hydrogencarbonate (10 mL) and a saturated aqueous solution of sodium chloride (10 mL). The washed solution was dried over sodium sulfate, the dried organic layer was filtered, and the filtrate was concentrated under reduced pressure. The residue was purified by flash column chromatography on silica gel (40% grading to 50% diethyl ether in *n*-pentane) to yield 3α,14β-diacetoxy-16-nor-*ent*-pimar-15α,8-olide (DAP, **30**) (14.8 mg, 36.4 μmol, 73%) as a white solid.

Analytical data of 3α,14β-diacetoxy-16-nor-*ent*-pimar-15α,8-olide (DAP, **30**):

TLC (40% diethyl ether in *n*-pentane): R_f = 0.40 (CAM).

mp: 147–148 °C.

$[\alpha]_D^{20}$ = –12.8 (c = 0.871, methanol).

$[\alpha]_D^{25}$ (literature)[16] = –8.5 (c = 0.16, methanol).

^{1}H NMR (400 MHz, CD3OD): δ 4.85 (s, 1H), 4.54 – 4.46 (m, 1H), 2.18 (s, 3H), 2.04 (s, 3H), 1.91 – 1.56 (m, 10H), 1.48 – 1.36 (m, 2H), 1.23 – 1.15 (m, 1H), 1.07 (s, 3H), 1.05 – 1.00 (m, 1H), 1.02 (s, 3H), 0.95 (s, 3H), 0.92 (s, 3H).

^{13}C NMR (101 MHz, CD3OD): δ 178.6, 172.8, 171.4, 85.0, 82.1, 78.6, 54.8, 47.2, 46.8, 38.8, 38.3, 37.4, 35.0, 28.8, 27.8, 24.4, 21.1, 20.7, 19.3, 19.2, 18.8, 17.4, 15.5.

IR (ATR, neat): $\tilde{\nu}$ = 2950 (w), 2876 (w), 1782 (s), 1733 (s), 1455 (w), 1366 (m), 1287 (w), 1245 (s), 1224 (s), 1155 (w), 1138 (m), 1116 (m), 1069 (m), 1029 (m), 1011 (m), 977 (m), 950 (m), 901 (w),

854 (w), 835 (w), 755 (w), 719 (w), 651 (w), 629 (w), 610 (w), 588 (w), 572 (w), 535 (w), 519 (w), 477 (w) cm^{-1}.

HRMS (ESI): calcd for $C_{23}H_{34}NaO_6^+$ $[M+Na]^+$: 429.2248; found: 429.2241.

NMR comparison for 3α,14β-diacetoxy-16-nor-*ent*-pimar-15α,8-olide (DAP, **30**):

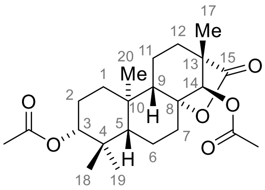

Atom number	Natural **30**[16] (400 MHz, CD_3OD) δ_H [ppm]	Synthetic **30** (400 MHz, CD_3OD) δ_H [ppm]	$\lvert\Delta\delta_H\rvert$ [ppm]
1α	1.72 (m, 1H)	1.72 (m, 1H)	0.00
1β	1.18 (m, 1H)	1.19 (m, 1H)	–0.01
2α	1.64 (m, 1H)	1.68–1.60 (m, 2H)	—
2β	1.62 (m, 1H)		—
3	4.50 (dd, *J* = 10.2, 6.2 Hz, 1H)	4.50 (m, 1H)	0.00
5	1.02 (m, 1H)	1.03 (m, 1H)	–0.01
6α	1.64 (m, 1H)	1.67–1.59 (m, 2H)	—
6β	1.61 (m, 1H)		—
7α	1.86 (m, 1H)	1.87 (m, 1H)	–0.01
7β	1.68 (m, 1H)	1.69 (m, 1H)	–0.01
9	1.58 (m, 1H)	1.59 (m, 1H)	–0.01
11α	1.42 (m, 1H)	1.41 (m, 1H)	0.01
11β	**1.37 (m, 1H)***	1.73 (m, 1H)	–0.36
12α	1.43 (m, 1H)	1.43 (m, 1H)	0.00
12β	1.80 (m, 1H)	1.82 (m, 1H)	–0.02
14	4.85 (br, 1H)	4.85 (s, 1H)	0.00
17	1.07 (s, 3H)	1.07 (s, 3H)	0.00
18 (20)**	1.02 (s, 3H)	1.02 (s, 3H)	0.00
19	0.95 (s, 3H)	0.95 (s, 3H)	0.00
20 (18)**	0.92 (s, 3H)	0.92 (s, 3H)	0.00
3-$OCCH_3$	2.03 (s, 3H)	2.04 (s, 3H)	–0.01
14-$OCCH_3$	2.18 (s, 3H)	2.18 (s, 3H)	0.00

*Appears to be an error in writing in ref 16 upon comparison with the literature spectrum in the supporting information of ref 16

**Corrected assignment in parenthesis

Atom number	Natural **30**[16] (101 MHz, CD_3OD) δ_C [ppm]	Synthetic **30** (101 MHz, CD_3OD) δ_C [ppm]	$\lvert\Delta\delta_C\rvert$ [ppm]
1	38.5	38.3	0.2
2	24.5	24.4	0.1
3	82.2	82.1	0.1
4	38.9	38.8	0.1
5	54.9	54.8	0.1
6	19.4	19.2	0.2
7	35.1	35.0	0.1
8	85.1	85.0	0.1
9	47.3	47.2	0.1
10	37.6	37.4	0.2
11	19.5	19.3	0.2
12	**28.6***	27.8	0.8
13	47.0	46.8	0.2
14	78.7	78.6	0.1
15	178.7	178.6	0.1
17	19.0	18.8	0.2
18	29.0	28.8	0.2
19	17.6	17.4	0.2
20	15.6	15.5	0.1
3-OC$\underline{C}H_3$	21.2	21.1	0.1
3-O$\underline{C}CH_3$	172.9	172.8	0.1
14-OC$\underline{C}H_3$	20.9	20.7	0.2
14-O$\underline{C}CH_3$	171.6	171.4	0.2

*Appears to be an error in writing in ref 16 upon comparison with the literature spectrum in the supporting information of ref 16

A systematic shift of 0.1–0.2 ppm between natural **30** and synthetic **30** is observed, which might be due to a different CD_3OD resonance reference in the literature.

The ^{1}H NMR and ^{13}C NMR data are in good agreement with the literature.

2.5.4 Lonchophylloid B (3)

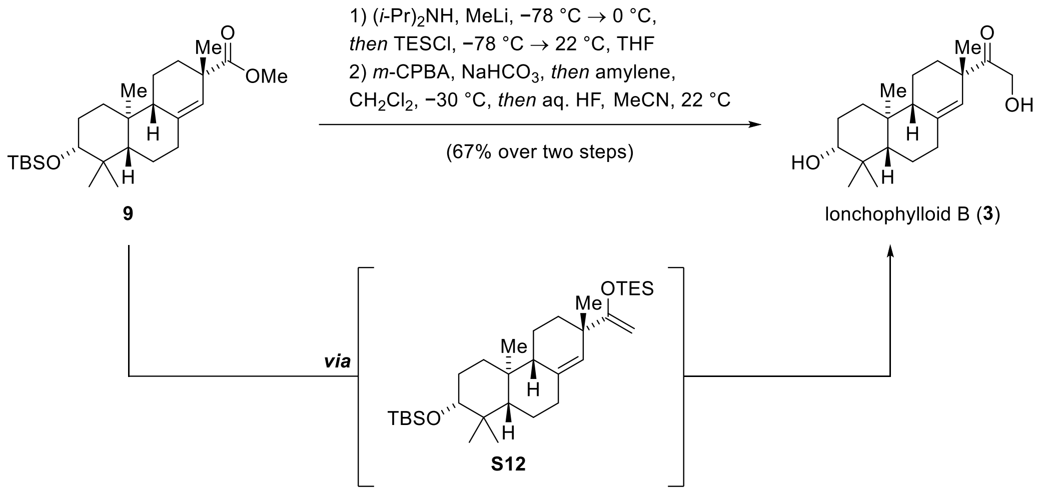

Enol ether **S12** was prepared according to a modified Fehr procedure[18]: To a solution of diisopropylamine (326 µL, 2.31 mmol, 10.0 equiv) in tetrahydrofuran (3.00 mL) was added a solution of methyl lithium in diethyl ether (361 µL, 1.60 M, 578 µmol; 2.50 equiv) at –78 °C. After 10 min, a solution of alkene **9** (100.5 mg, 231.2 µmol, 1 equiv) in tetrahydrofuran (3.00 mL) was added at –78 °C, the reaction mixture was warmed to 0 °C by exchange of the cooling bath, and a solution of methyl lithium in diethyl ether (3.03 mL, 1.60 M, 4.86 mmol; 21.0 equiv) was added at 0 °C. After stirring for 1 h at 0 °C, the yellow reaction mixture was cooled to –78 °C and chlorotriethylsilane (1.05 mL, 6.24 mmol, 27.0 equiv) was added. After stirring for 10 min at –78 °C, the reaction mixture was allowed to warm to 22 °C through removal of the cooling bath and stirring was continued for 1 h 10 min, during which the solution turned deep yellow. The reaction mixture was poured into a saturated aqueous solution of sodium hydrogencarbonate (100 mL) and the biphasic mixture was extracted with diethyl ether (1 × 100 mL, 1 × 50 mL). The combined organic layers were dried over sodium sulfate, the dried organic layer was filtered, and the filtrate was concentrated under reduced pressure to give a yellow oil (506.2 mg), which was used for the next step without further purification.

To a solution of crude enol ether **S12** (506.2 mg, theoretically: 123.2 mg, 231.2 µmol, 1 equiv) in dichloromethane (6.00 mL) was added successively sodium hydrogencarbonate (390 mg, 4.64 mmol, 20.1 equiv,) and *meta*-chloroperoxybenzoic acid (*m*-CPBA) (155 mg, 77.0 wt%, 694 µmol, 3.00 equiv) at –30 °C. After stirring for 1 h 50 min at –30 °C, amylene (2-methyl-2-butene) (599 µL, 5.78 mmol, 25.0 equiv) was added at –30 °C and the reaction mixture was allowed to warm to 22 °C through removal of the cooling bath. After 10 min, acetonitrile (6.00 mL) and an

aqueous solution of hydrogen fluoride (837 µL, 48.0 wt%, 23.1 mmol, 100 equiv) were added successively. After stirring for 20 min, further aqueous solution of hydrogen fluoride (837 µL, 48.0 wt%, 23.1 mmol, 100 equiv) was added. Stirring was continued at 22 °C for 50 min, after which the reaction was diluted with ethyl acetate (120 mL) and successively washed with a 1 M aqueous solution of sodium hydroxide (2 × 60 mL), a saturated aqueous solution of ammonium chloride (30 mL) and a saturated aqueous solution of sodium chloride (30 mL). The washed solution was dried over sodium sulfate, the dried organic layer was filtered, and the filtrate was concentrated under reduced pressure. The residue was purified by flash column chromatography on silica gel (20% grading to 30% ethyl acetate in cyclohexane) to yield lonchophylloid B (**3**) (49.9 mg, 156 µmol, 67% over two steps) as a white solid.

Analytical data of lonchophylloid B (**3**):

TLC (30% ethyl acetate in cyclohexane): R_f = 0.24 (CAM).

mp: 132–133 °C.

mp (literature)[16,19]: 132–133 °C.

$[\alpha]_D^{20}$ = +6.2 (c = 0.43, methanol).

$[\alpha]_D^{25}$ (literature)[16] = –10.2 (c = 0.13, methanol).

$[\alpha]_D^{25}$ (literature)[19] = –9.93 (c = 1.0, ethanol).

^{1}H NMR (400 MHz, $CDCl_3$): δ 5.39 (q, J = 1.9 Hz, 1H), 4.34 (d, J = 4.6 Hz, 2H), 3.25 (dd, J = 11.4, 3.9 Hz, 1H), 3.15 (t, J = 4.7 Hz, 1H), 2.36 (ddd, J = 14.4, 4.7, 2.0 Hz, 1H), 2.32 – 2.27 (m, 1H), 2.07 (tdt, J = 13.5, 5.5, 1.8, 1.3 Hz, 1H), 1.72 (t, J = 7.9 Hz, 1H), 1.69 – 1.55 (m, 4H), 1.54 – 1.45 (m, 1H), 1.39 (td, J = 12.9, 4.6 Hz, 1H), 1.32 (br, 1H), 1.20 – 1.10 (m, 3H), 1.12 (s, 3H), 1.05 (dd, J = 12.4, 2.7 Hz, 1H), 1.01 (s, 3H), 0.81 (s, 3H), 0.65 (s, 3H).

^{13}C NMR (101 MHz, $CDCl_3$): δ 214.8, 142.5, 123.4, 79.1, 66.0, 54.1, 50.9, 47.0, 39.1, 38.4, 36.9, 35.8, 32.8, 28.6, 27.6, 27.5, 22.2, 20.3, 15.8, 14.6.

IR (ATR, neat): $\tilde{\nu}$ = 3421 (br), 2936 (s), 2870 (m), 2851 (m), 1707 (s), 1453 (m), 1383 (m), 1364 (m), 1267 (m), 1088 (m), 1034 (s), 1020 (s), 1000 (m), 970 (m), 932 (m), 874 (m), 732 (w), 704 (w), 645 (w), 607 (w), 585 (w), 551 (w) cm^{-1}.

HRMS (ESI): calcd for $C_{20}H_{32}NaO_3^+$ [M+Na]$^+$: 343.2244; found: 343.2239.

ECD (MeCN, 25 °C, 500 μM): λ_{max} ($\Delta\varepsilon$): 221 (–3.30), 292 (+0.71).

ECD (MeCN, 94 μM, literature)[16]: λ_{max} ($\Delta\varepsilon$): 216 (–1.19), 291 (+0.31).

Comparison of the measured ECD spectrum with the ECD spectrum in ref 16 indicates that the same enantiomer has been prepared.

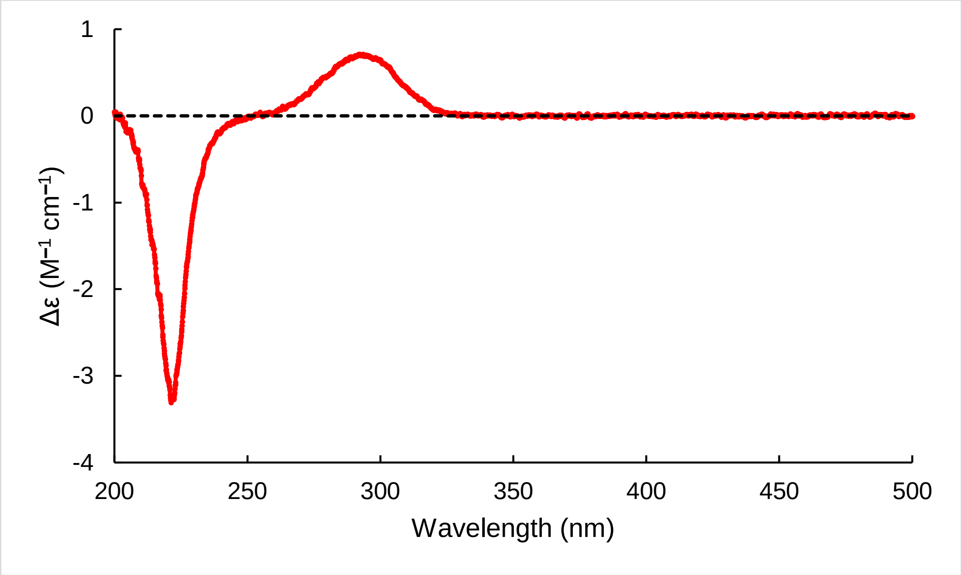

Figure 2. Experimental ECD spectrum of lonchophylloid B (**3**).

NMR comparison for lonchophylloid B (**3**):

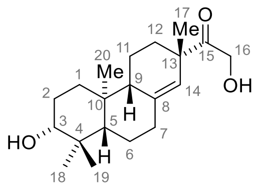

Atom number	Natural **3**[19] (300 MHz, $CDCl_3$) δ_H [ppm]	Synthetic **3** (400 MHz, $CDCl_3$) δ_H [ppm]	$\lvert\Delta\delta_H\rvert$ [ppm]
1α	1.61 (m, 1H)	1.62 (m, 1H)	–0.01
1β	1.14 (m, 1H)	1.14 (m, 1H)	0.00
2α	1.54 (m, 1H)	1.52 (m, 1H)	0.02
2β	**1.18 (m, 1H)***	1.63 (m, 1H)	–0.45
3	3.25 (dd, *J* = 11.5, 4.1 Hz, 1H)	3.25 (dd, *J* = 11.4, 3.9 Hz, 1H)	0.00
5	1.03 (dd, *J* = 12.6, 2.7 Hz, 1H)	1.05 (dd, *J* = 12.4, 2.7 Hz, 1H)	–0.02
6α	1.64 (m, 1H)	1.65 (m, 1H)	–0.01
6β	1.37 (ddd, *J* = 14.1, 12.6, 5.7 Hz, 1H)	1.39 (td, *J* = 12.9, 4.6 Hz, 1H)	–0.02
7α	2.03 (td, *J* = 13.8, 5.4 Hz, 1H)	2.07 (tdt, *J* = 13.5, 5.5, 1.8, 1.3 Hz, 1H)	–0.04
7β	2.38 (dt, *J* = 13.8, 5.4 Hz, 1H)	2.36 (ddd, *J* = 14.4, 4.7, 2.0 Hz, 1H)	0.02
9	1.68 (t, *J* = 8.4 Hz, 1H)	1.72 (t, *J* = 7.9 Hz, 1H)	–0.04
11α	1.19 (m, 1H)	1.14 (m, 1H)	0.05
11β	**1.48 (ddd, *J* = 14.1, 12.6, 3.3 Hz, 1H)***	1.59 (m, 1H)	–0.11
12α	2.33 (dt, *J* = 12.6, 5.3 Hz, 1H)	2.30 (m, 1H)	0.03
12β	1.07 (m, 1H)	1.11 (m, 1H)	–0.04
14	**5.53 (d, *J* = 1.6 Hz, 1H)****	5.39 (q, *J* = 1.9 Hz, 1H)	–0.14
16	4.35 (s, 2H)	4.34 (d, *J* = 4.6 Hz, 2H)	0.01
17	1.12 (s, 3H)	1.12 (s, 3H)	0.00
18	0.80 (s, 3H)	0.81 (s, 3H)	–0.01
19	1.01 (s, 3H)	1.01 (s, 3H)	0.00

20	0.64 (s, 3H)	0.65 (s, 3H)	–0.01

*Presumably an assignment error

**For ref 16 (compare literature spectrum in the supporting information), this ^{1}H-signal appears at 5.38 ppm, which is in good agreement with our data.

Atom number	Natural **3**[19] (75 MHz, $CDCl_3$) δ_C [ppm]	Synthetic **3** (101 MHz, $CDCl_3$) δ_C [ppm]	$\lvert\Delta\delta_C\rvert$ [ppm]
1	36.8	36.9	–0.1
2	27.4	27.6	–0.2
3	78.9	79.1	–0.2
4	39.0	39.1	–0.1
5	54.0	54.1	–0.1
6	22.0	22.2	–0.2
7	35.6	35.8	–0.2
8	142.3	142.5	–0.2
9	50.7	50.9	–0.2
10	38.3	38.4	–0.1
11	20.1	20.3	–0.2
12	32.6	32.8	–0.2
13	46.8	47.0	–0.2
14	123.2	123.4	–0.2
15	214.7	214.8	–0.1
16	65.8	66.0	–0.2
17 (19)*	28.4	28.6	–0.2
18	15.7	15.8	–0.2
19 (17)*	27.4	27.5	–0.1
20	14.5	14.6	–0.1

*Corrected assignment in parenthesis

A systematic shift of –0.1 to –0.2 ppm between natural **3** and synthetic **3** is observed, which might be due to a different resonance reference in the literature.

The ^{13}C NMR data are in good agreement with the literature, whereas there are some discrepancies within the ^{1}H NMR data. Unfortunately, no literature spectra were included in ref 19 for further comparison. However, ref 16 included ^{13}C and ^{1}H NMR spectra, in which at least a clear agreement was found for the olefinic proton (H-14) with our data.

2.5.5 Darutigenol (**31**) and *ent*-3β,15*R*,16-trihydroxypimar-8(14)-ene (THP, **4**)

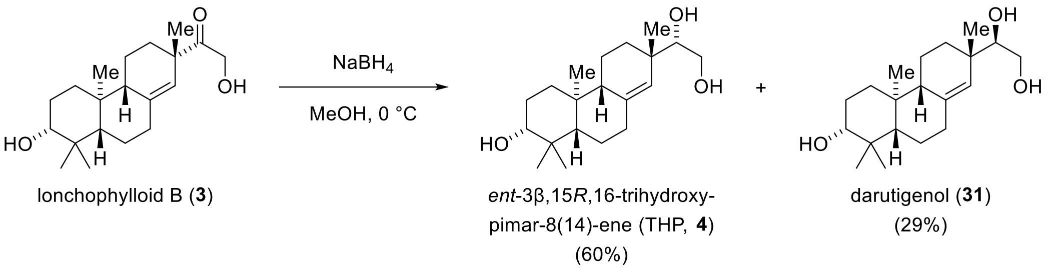

To a solution of lonchophylloid B (**3**) (34.5 mg, 108 µmol, 1 equiv) in methanol (3.50 mL) was added sodium borohydride (20.4 mg, 538 µmol, 5.00 equiv) at 0 °C. After stirring for 55 min at 0 °C, the colorless solution was diluted with ethyl acetate (30 mL). Water (5 mL) and a saturated aqueous solution of sodium hydrogencarbonate (10 mL) were added cautiously in succession at 0 °C. The organic layer was separated, and the aqueous layer was extracted with ethyl acetate (3 × 10 mL). The combined organic layers were washed with a saturated aqueous solution of sodium chloride (10 mL), the washed solution was dried over sodium sulfate, the dried organic layer was filtered, and the filtrate was concentrated under reduced pressure. The residue was purified by flash column chromatography on silica gel (3% methanol in dichloromethane) to yield *ent*-3β,15*R*,16-trihydroxypimar-8(14)-ene (THP, **4**) (20.9 mg, 64.8 µmol, 60%) as a white solid and darutigenol (**31**) (9.9 mg, 31 µmol, 29%) as a white solid.

Analytical data of *ent*-3β,15*R*,16-trihydroxypimar-8(14)-ene (THP, **4**):

TLC (6% methanol in dichloromethane): R_f = 0.29 (CAM).

mp: 158–159 °C.

$[\alpha]_D^{20}$ = –15.8 (c = 0.994, methanol).

$[\alpha]_D^{25}$ (literature)[20] = –48 (c = 0.1, methanol).

^{1}H NMR (400 MHz, CD_3OD): δ 5.36 (t, J = 2.2 Hz, 1H), 4.58 (br, 1H), 3.69 (dd, J = 11.0, 2.5 Hz, 1H), 3.53 (dd, J = 8.8, 2.5 Hz, 1H), 3.42 (dd, J = 11.0, 8.8 Hz, 1H), 3.20 (dd, J = 9.1, 6.9 Hz, 1H), 2.31 (ddd, J = 14.1, 4.5, 2.2 Hz, 1H), 2.05 (td, J = 13.1, 5.0 Hz, 1H), 1.76 (dt, J = 13.1, 3.5 Hz, 1H), 1.72 – 1.53 (m, 7H), 1.40 (qd, J = 12.9, 4.5 Hz, 1H), 1.23 – 1.11 (m, 2H), 1.07 (dd, J = 12.4, 2.5 Hz, 1H), 0.99 (s, 3H), 0.88 (s, 3H), 0.84 (s, 3H), 0.81 (s, 3H).

^{13}C NMR (101 MHz, CD_3OD): δ 138.0, 130.4, 80.1, 79.8, 64.4, 55.7, 51.5, 40.1, 39.5, 38.6, 38.4, 37.3, 32.3, 29.1, 28.3, 24.0, 23.6, 19.9, 16.5, 15.8.

^{1}H NMR (400 MHz, C_6D_5N): δ 6.03 (d, *J* = 4.3 Hz, 1H), 5.98 (s, 1H), 5.80 (s, 1H), 5.79 (d, *J* = 5.3 Hz, 1H), 4.17 (d, *J* = 10.0 Hz, 1H), 4.09 (d, *J* = 8.7 Hz, 1H), 4.02 (t, *J* = 9.1 Hz, 1H), 3.50 (dt, *J* = 11.3, 4.8 Hz, 1H), 2.36 (ddd, *J* = 13.9, 4.4, 2.1 Hz, 1H), 2.14 (td, *J* = 13.2, 4.5 Hz, 1H), 1.99 – 1.92 (m, 1H), 1.91 – 1.76 (m, 2H), 1.73 – 1.52 (m, 5H), 1.39 – 1.28 (m, 2H), 1.23 (s, 6H), 1.15 (td, *J* = 13.1, 3.9 Hz, 1H), 1.10 (dd, *J* = 12.4, 2.5 Hz, 1H), 1.05 (s, 3H), 0.79 (s, 3H).

^{13}C NMR (101 MHz, C_6D_5N): δ 136.8, 131.3, 79.5, 78.7, 64.4, 55.1, 50.8, 40.1, 39.2, 38.4, 38.1, 37.1, 32.4, 29.6, 29.0, 24.5, 23.3, 19.8, 17.1, 15.8.

IR (ATR, neat): $\tilde{\nu}$ = 3367 (br), 2936 (s), 2869 (s), 1658 (w), 1548 (w), 1455 (m), 1383 (m), 1366 (m), 1330 (m), 1305 (m), 1235 (w), 1183 (w), 1122 (w), 1074 (s), 1030 (s), 969 (m), 937 (m), 882 (m), 866 (m), 826 (w), 750 (w), 635 (m), 590 (m), 533 (m) cm^{-1}.

HRMS (ESI): calcd for $C_{20}H_{34}NaO_3^+$ $[M+Na]^+$: 345.2400; found: 345.2401.

NMR comparison for *ent*-3β,15*R*,16-trihydroxypimar-8(14)-ene (THP, **4**):

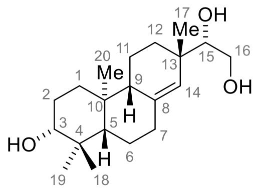

Atom number	Natural **4**[20] (400 MHz, C_6D_5N) δ_H [ppm]	Synthetic **4** (400 MHz, C_6D_5N) δ_H [ppm]	$\lvert\Delta\delta_H\rvert$ [ppm]
1α	1.60 (m, 1H)	1.68 (m, 1H)	–0.08
1β	1.10 (m, 1H)	1.15 (td, *J* = 13.1, 3.9 Hz, 1H)	–0.05
2α	1.80 (m, 1H)	1.87 (m, 1H)	–0.07
2β	1.80 (m, 1H)	1.87 (m, 1H)	–0.07
3	3.45 (m, 1H)	3.50 (dt, *J* = 11.3, 4.8 Hz, 1H)	–0.05
5	1.05 (m, 1H)	1.10 (dd, *J* = 12.4, 2.5 Hz, 1H)	–0.05
6α	1.58 (m, 1H)	1.63 (m, 1H)	–0.05
6β	1.32 (m, 1H)	1.35 (m, 1H)	–0.03
7α	2.30 (m, 1H)	2.36 (ddd, *J* = 13.9, 4.4, 2.1 Hz, 1H)	–0.06
7β	2.10 (m, 1H)	2.14 (td, *J* = 13.2, 4.5 Hz, 1H)	–0.04
9	1.64 (m, 1H)	1.70 (m, 1H)	–0.06
11α	1.54 (m, 1H)	1.69–1.56 (m, 2H)	—
11β	1.54 (m, 1H)		—
12α	1.92 (m, 1H)	1.96 (m, 1H)	–0.04
12β	1.29 (m, 1H)	1.33 (m, 1H)	–0.04
14	5.8 (s, 1H)	5.80 (s, 1H)	0.0
15	4.06 (brd, *J* = 9.8 Hz, 1H)	4.09 (d, *J* = 8.7 Hz, 1H)	–0.03
16α	4.13 (brd, *J* = 10.5 Hz, 1H)	4.17 (d, *J* = 10.0 Hz, 1H)	–0.04
16β	3.98 (brdd, *J* = 10.5, 9.8 Hz, 1H)	4.02 (t, *J* = 9.1 Hz, 1H)	–0.04
17	1.19 (s, 3H)	1.23 (s, 3H)	–0.04
18	1.19 (s, 3H)	1.23 (s, 3H)	–0.04
19	1.00 (s, 3H)	1.05 (s, 3H)	–0.05

20	0.73 (s, 3H)	0.76 (s, 3H)	–0.03
3-OH	—	5.98 (s, 1H)	—
15-OH	—	6.03 (d, *J* = 4.3 Hz, 1H)	—
16-OH	—	5.79 (d, *J* = 5.3 Hz, 1H)	—

A systematic shift of approximately –0.05 ppm between natural **4** and synthetic **4** is observed, which might be due to a different resonance reference in the literature.

Atom number	Natural **4**[20] (100 MHz, C_6D_5N) δ_C [ppm]	Synthetic **4** (101 MHz, C_6D_5N) δ_C [ppm]	$\lvert\Delta\delta_C\rvert$ [ppm]
1	37.6	38.1	–0.5
2	28.5	29.0	–0.5
3	78.1	78.7	–0.6
4	39.5	40.1	–0.6
5	54.6	55.1	–0.5
6	22.7	23.3	–0.6
7	36.6	37.1	–0.5
8	136.2	136.8	–0.6
9	50.3	50.8	–0.5
10 (13)*	37.9	38.4	–0.5
11	19.2	19.8	–0.6
12	31.8	32.4	–0.6
13 (10)*	38.7	39.2	–0.5
14	130.9	131.3	–0.4
15	79.0	79.5	–0.5
16	63.9	64.4	–0.5
17	23.9	24.5	–0.6
18	29.1	29.6	–0.5
19	16.6	17.1	–0.5
20	15.3	15.8	–0.5

*Corrected assignment in parenthesis

A systematic shift of –0.5 to –0.6 ppm between natural **4** and synthetic **4** is observed, which might be due to a different resonance reference in the literature.

The ^{1}H NMR and ^{13}C NMR data are in good agreement with the literature upon considering systematic shifts due to different resonance references.

NMR spectroscopic data for *ent*-3β,15*R*,16-trihydroxypimar-8(14)-ene (THP, **4**) in CD_3OD:

Atom number	^{1}H NMR (400 MHz, CD_3OD) δ_H [ppm]	^{13}C NMR (101 MHz, CD_3OD) δ_C [ppm]
1	1.76 (dt, *J* = 13.1, 3.5 Hz, 1H)<br>1.19 (m, 1H)	38.6
2	1.61 (m, 2H)	28.3
3	3.20 (dd, *J* = 9.1, 6.9 Hz, 1H)	79.8
4	—	40.1
5	1.07 (dd, *J* = 12.4, 2.5 Hz, 1H)	55.7
6	1.64 (m, 1H)<br>1.40 (qd, *J* = 12.9, 4.5 Hz, 1H)	23.6
7	2.31 (ddd, *J* = 14.1, 4.5, 2.2 Hz, 1H)<br>2.05 (td, *J* = 13.1, 5.0 Hz, 1H)	37.3
8	—	138.0
9	1.68 (m, 1H)	51.5
10	—	39.5
11	1.70–1.54 (m, 2H)	19.9
12	1.69 (m, 1H)<br>1.15 (m, 1H)	32.3
13	—	38.4
14	5.36 (t, *J* = 2.2 Hz, 1H)	130.4
15	3.53 (dd, *J* = 8.8, 2.5 Hz, 1H)	80.1
16	3.69 (dd, *J* = 11.0, 2.5 Hz, 1H)<br>3.42 (dd, *J* = 11.0, 8.8 Hz, 1H)	64.4
17	0.88 (s, 3H)	24.0
18	0.99 (s, 3H)	29.1

19	0.81 (s, 3H)	16.5
20	0.84 (s, 3H)	15.8

Analytical data of darutigenol (**31**):

TLC (6% methanol in dichloromethane): R_f = 0.26 (CAM).

mp: 165–167 °C.

mp (literature): 163 °C[21], 168 °C[22], 168–170 °C[23].

$[\alpha]_D^{20}$ = –5.0 (*c* = 0.45, methanol).

$[\alpha]_D$ (literature)[21] = –10 (*c* = 1.0, methanol).

^{1}H NMR (400 MHz, CD_3OD): δ 5.17 (q, *J* = 1.8 Hz, 1H), 3.69 (dd, *J* = 11.0, 2.3 Hz, 1H), 3.57 (dd, *J* = 9.0, 2.3 Hz, 1H), 3.46 (dd, *J* = 11.0, 9.0 Hz, 1H), 3.20 (dd, *J* = 8.6, 7.4 Hz, 1H), 2.28 (ddd, *J* = 14.2, 4.5, 2.1 Hz, 1H), 2.05 (td, *J* = 13.9, 13.5, 5.5 Hz, 1H), 1.98 (dtd, *J* = 13.2, 4.0, 1.2 Hz, 1H), 1.77 – 1.68 (m, 2H), 1.67 – 1.51 (m, 5H), 1.38 (qd, *J* = 13.1, 4.6 Hz, 1H), 1.25 – 1.14 (m, 1H), 1.07 (dd, *J* = 12.4, 2.6 Hz, 1H), 0.99 (s, 3H), 0.96 – 0.87 (m, 1H), 0.83 (s, 3H), 0.82 (s, 3H), 0.81 (s, 3H).

^{13}C NMR (101 MHz, CD_3OD): δ 139.8, 129.6, 79.8, 77.5, 64.3, 55.7, 52.1, 40.1, 39.1, 38.5, 38.4, 37.1, 33.3, 29.1, 28.3, 23.4, 23.0, 19.3, 16.5, 15.3.

^{1}H NMR (400 MHz, C_6D_5N): δ 6.06 (t, *J* = 5.6 Hz, 1H), 6.02 (d, *J* = 4.3 Hz, 1H), 5.79 (d, *J* = 5.3 Hz, 1H), 5.47 (q, *J* = 1.7 Hz, 1H), 4.23 (ddd, *J* = 10.4, 6.3, 2.0 Hz, 1H), 4.13 (ddd, *J* = 9.1, 4.2, 2.0 Hz, 1H), 4.06 (ddd, *J* = 10.3, 8.8, 4.6 Hz, 1H), 3.49 (dt, *J* = 11.3, 4.8 Hz, 1H), 2.48 (dt, *J* = 13.1, 3.5 Hz, 1H), 2.36 (ddd, *J* = 14.0, 4.5, 2.1 Hz, 1H), 2.14 (td, *J* = 13.3, 4.9 Hz, 1H), 1.90 – 1.48 (m, 7H), 1.35 (qd, *J* = 12.9, 4.5 Hz, 1H), 1.24 (s, 3H), 1.21 (s, 3H), 1.19 – 1.06 (m, 3H), 1.05 (s, 3H), 0.76 (s, 3H).

^{13}C NMR (101 MHz, C_6D_5N): δ 138.8, 130.0, 78.7, 77.2, 64.5, 55.1, 51.4, 40.0, 38.8, 38.5, 38.0, 36.9, 33.4, 29.5, 28.9, 23.8, 23.2, 19.2, 17.1, 15.4.

IR (ATR, neat): $\tilde{\nu}$ = 3370 (br), 2940 (s), 2870 (m), 1456 (m), 1383 (w), 1366 (w), 1308 (w), 1084 (m), 1033 (m), 1014 (m), 966 (w), 939 (w), 918 (w), 881 (w), 851 (w), 802 (w), 680 (w), 640 (w), 564 (w) cm^{-1}.

HRMS (ESI): calcd for $C_{20}H_{34}NaO_3^+$ [M+Na]$^+$: 345.2400; found: 345.2393.

NMR comparison for darutigenol (**31**):

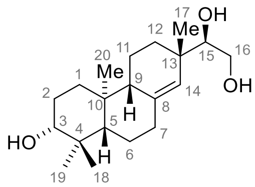

Atom number	Natural **31**[24] (500 MHz, CD_3OD) δ_H [ppm]	Synthetic **31** (400 MHz, CD_3OD) δ_H [ppm]	$\lvert\Delta\delta_H\rvert$ [ppm]
1α	1.61 (m, 1H)	1.73 (m, 1H)	–0.12
1β	1.15 (td, *J* = 12.9, 4.8 Hz, 1H)	1.22 (m, 1H)	–0.07
2α	1.50 (m, 1H)	1.61 (m, 1H)	–0.11
2β	1.50 (m, 1H)	1.61 (m, 1H)	–0.11
3	3.10 (dd, *J* = 11.0, 4.8 Hz, 1H)	3.20 (dd, *J* = 8.6, 7.4 Hz, 1H)	–0.10
5	0.97 (dd, *J* = 10.3, 1.8 Hz, 1H)	1.07 (dd, *J* = 12.4, 2.6 Hz, 1H)	–0.10
6α	1.53 (m, 1H)	1.63 (m, 1H)	–0.10
6β	1.28 (qd, *J* = 12.9, 4.4 Hz, 1H)	1.38 (qd, *J* = 13.1, 4.6 Hz, 1H)	–0.10
7α	2.18 (dt, *J* = 13.4, 2.1 Hz, 1H)	2.28 (ddd, *J* = 14.2, 4.5, 2.1 Hz, 1H)	–0.10
7β	1.95 (m, 1H)	2.05 (td, *J* = 13.9, 13.5, 5.5 Hz, 1H)	–0.10
9	1.61 (m, 1H)	1.71 (m, 1H)	–0.10
11α	1.44 (m, 1H)	1.54 (m, 1H)	–0.10
11β	1.44 (m, 1H)	1.54 (m, 1H)	–0.10
12α	1.88 (brd, *J* = 13.1 Hz, 1H)	1.98 (dtd, *J* = 13.2, 4.0, 1.2 Hz, 1H)	–0.10
12β	0.82 (m, 1H)	0.91 (m, 1H)	–0.09
14	5.07 (s, 1H)	5.17 (q, *J* = 1.8 Hz, 1H)	–0.10
15	3.46 (brd, *J* = 8.9 Hz, 1H)	3.57 (dd, *J* = 9.0, 2.3 Hz, 1H)	–0.09
16α	3.36 (dd, *J* = 10.8, 8.9 Hz, 1H)	3.46 (dd, *J* = 11.0, 9.0 Hz, 1H)	–0.10
16β	3.58 (brd, *J* = 10.8 Hz, 1H)	3.69 (dd, *J* = 11.0, 2.3 Hz, 1H)	–0.11
17	0.74 (s, 3H)	0.83 (s, 3H)	–0.09
18	0.89 (s, 3H)	0.99 (s, 3H)	–0.10

19	0.72 (s, 3H)	0.82 (s, 3H)	–0.10
20	0.71 (s, 3H)	0.81 (s, 3H)	–0.10

A systematic shift of approximately –0.10 ppm between natural **31** and synthetic **31** is observed, which might be due to a different resonance reference in the literature.

Atom number	Synthetic **31** (101 MHz, CD_3OD) δ_C [ppm]
1	38.4
2	28.3
3	79.8
4	40.1
5	55.7
6	23.4
7	37.1
8	139.8
9	52.1
10	39.1
11	19.3
12	33.3
13	38.5
14	129.6
15	77.5
16	64.3
17	23.0
18	29.1
19	16.5
20	15.3

Atom number	Natural **31**[20] (400 MHz, C_6D_5N) δ_H [ppm]	Synthetic **31** (400 MHz, C_6D_5N) δ_H [ppm]	$\lvert\Delta\delta_H\rvert$ [ppm]
1α	1.51 (m, 1H)	1.61 (m, 1H)	–0.10
1β	**1.51 (m, 1H)***	1.16 (m, 1H)	0.35
2α	1.80 (m, 1H)	1.83 (m, 1H)	–0.03
2β	1.75 (m, 1H)	1.79 (m, 1H)	–0.04
3	3.45 (dd, *J* = 11.2, 4.3 Hz, 1H)	3.49 (dt, *J* = 11.3, 4.8 Hz, 1H)	–0.04
5	1.09 (m, 1H)	1.10 (m, 1H)	–0.01
6α	1.58 (m, 1H)	1.62 (m, 1H)	–0.04
6β	1.32 (m, 1H)	1.35 (qd, *J* = 12.9, 4.5 Hz, 1H)	–0.03
7α	2.30 (dd, *J* = 10.2, 2.4 Hz, 1H)	2.36 (ddd, *J* = 14.0, 4.5, 2.1 Hz, 1H)	–0.06
7β	2.09 (d, *J* = 5.0 Hz, 1H)	2.14 (td, *J* = 13.3, 4.9 Hz, 1H)	–0.05
9	1.67 (m, 1H)	1.72 (m, 1H)	–0.05
11α	1.62 (m, 1H)	1.67 (m, 1H)	–0.05
11β	1.54 (m, 1H)	1.55 (m, 1H)	–0.01
12α	2.44 (m, 1H)	2.48 (dt, *J* = 13.1, 3.5 Hz, 1H)	–0.04
12β	1.13 (m, 1H)	1.14 (m, 1H)	–0.01
14	5.40 (s, 1H)	5.47 (q, *J* = 1.7 Hz, 1H)	–0.07
15	4.09 (dd, *J* = 10.2, 1.7 Hz, 1H)	4.13 (ddd, *J* = 9.1, 4.2, 2.0 Hz, 1H)	–0.04
16α	4.20 (d, *J* = 10.2 Hz, 1H)	4.23 (ddd, *J* = 10.4, 6.3, 2.0 Hz, 1H)	–0.03
16β	4.02 (m, 1H)	4.06 (ddd, *J* = 10.3, 8.8, 4.6 Hz, 1H)	–0.04
17 (18)**	1.22 (s, 3H)	1.24 (s, 3H)	–0.02
18 (17)**	1.17 (s, 3H)	1.21 (s, 3H)	–0.04
19	1.01 (s, 3H)	1.05 (s, 3H)	–0.04
20	0.72 (s, 3H)	0.76 (s, 3H)	–0.04
3-OH	—	5.79 (d, *J* = 5.3 Hz, 1H)	—
15-OH	—	6.02 (d, *J* = 4.3 Hz, 1H)	—

16-OH	—	6.06 (t, *J* = 5.6 Hz, 1H)	—

*Presumably an assignment error

**Corrected assignment in parenthesis

A systematic shift of approximately –0.04 ppm between natural **31** and synthetic **31** is observed, which might be due to a different resonance reference in the literature.

Atom number	Natural **31**[21] (25.2 MHz, C_6D_5N) δ_C [ppm]	Natural **31**[20] (101 MHz, C_6D_5N) δ_C [ppm]	Synthetic **31** (101 MHz, C_6D_5N) δ_C [ppm]	$\lvert\Delta\delta_C\rvert$* [ppm]
1	37.5	37.6	38.0	–0.4
2	28.5	28.5	28.9	–0.4
3	78.2	78.2	78.7	–0.5
4	39.5	39.6	40.0	–0.4
5	54.7	54.7	55.1	–0.4
6	22.8	22.7	23.2	–0.5
7	36.5	36.5	36.9	–0.4
8	138.5	138.3	138.8	–0.5
9	51.0	51.0	51.4	–0.4
10	38.3	38.3 (C-13)**	38.8	–0.5
11	18.8	18.8	19.2	–0.4
12	32.9	32.9	33.4	–0.5
13	37.6	38.1 (C-10)**	38.5	–0.4
14	129.6	129.5	130.0	–0.5
15	76.7	76.8	77.2	–0.4
16	64.0	64.0	64.5	–0.5
17	23.3	23.3	23.8	–0.5
18	29.0	29.1	29.5	–0.4
19	14.9 (C-20)*	16.6	17.1	–0.5
20	16.6 (C-19)*	15.0	15.4	–0.4

*Compared to ref 20

**Originally proposed assignment

A systematic shift of –0.4 to –0.5 ppm between natural **31** and synthetic **31** is observed, which might be due to a different resonance reference in the literature.

The ^{1}H NMR and ^{13}C NMR data are in good agreement with the literature upon considering systematic shifts due to different resonance references.

2.5.6 (2*S*,3*S*,5*S*,9*S*,10*S*,13*S*)-2,3-dihydroxy-16-nor-*ent*-pimar-8(14)-en-15-oic acid (DHPA, **29**)

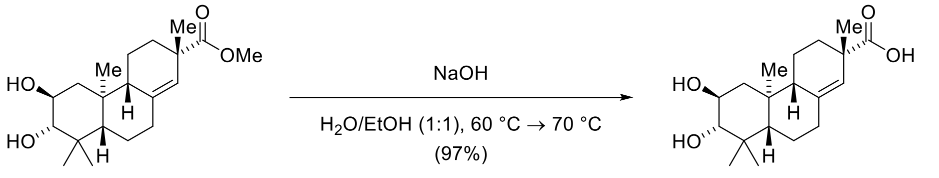

To a solution of diol **26** (18.3 mg, 54.4 µmol, 1 equiv) in ethanol (1.00 mL) was added an aqueous solution of sodium hydroxide (1.00 mL, 2.00 M, 2.00 mmol, 36.8 equiv) at 22 °C. The colorless solution was stirred at 60 °C for 5 h and then at 70 °C for 1.5 h. The reaction mixture was cooled to 22 °C, a 1 M aqueous solution of hydrochloric acid (5 mL) was added, and the mixture was extracted with dichloromethane/ethyl acetate (2:1 v/v, 4 × 10 mL). The combined organic layers were dried over sodium sulfate, the dried organic layer was filtered, and the filtrate was concentrated under reduced pressure to yield (2*S*,3*S*,5*S*,9*S*,10*S*,13*S*)-2,3-dihydroxy-16-nor-*ent*-pimar-8(14)-en-15-oic acid (DHPA, **29**) (17.0 mg, 52.7 µmol, 97%) as a white solid.

Analytical data of (2*S*,3*S*,5*S*,9*S*,10*S*,13*S*)-2,3-dihydroxy-16-nor-*ent*-pimar-8(14)-en-15-oic acid (DHPA, **29**):

TLC (8% methanol in dichloromethane): R_f = 0.31 (CAM).

mp: Decomposition at 207 °C before melting.

$[\alpha]_D^{20}$ = –47.7 (c = 1.00, methanol).

$[\alpha]_D^{25}$ (literature)[16] = –21.0 (c = 0.13, methanol).

^{1}H NMR (400 MHz, CD_3OD): 5.42 (q, J = 1.5 Hz, 1H), 3.57 (ddd, J = 11.7, 9.6, 4.2 Hz, 1H), 2.96 (d, J = 9.6 Hz, 1H), 2.35 (ddd, J = 14.2, 4.6, 2.1 Hz, 1H), 2.16 (dt, J = 12.8, 3.5 Hz, 1H), 2.07 (td, J = 13.6, 5.4 Hz, 1H), 1.91 (dd, J = 12.5, 4.2 Hz, 1H), 1.79 (t, J = 8.5 Hz, 1H), 1.69 – 1.61 (m, 2H), 1.54 – 1.37 (m, 2H), 1.19 (s, 3H), 1.20 – 1.09 (m, 3H), 1.02 (s, 3H), 0.83 (s, 3H), 0.78 (s, 3H).

^{13}C NMR (101 MHz, CD_3OD): δ 180.9, 139.5, 127.6, 84.2, 69.2, 55.3, 52.0, 46.6, 44.0, 40.6, 40.1, 36.6, 34.3, 29.5, 28.2, 23.3, 21.4, 17.6, 15.8.

IR (ATR, neat): $\tilde{\nu}$ = 3329 (br), 2942 (m), 1704 (m), 1459 (m), 1395 (m), 1265 (m), 1205 (m), 1162 (m), 1125 (m), 1092 (m), 1054 (s), 1038 (s), 996 (m), 968 (s), 895 (m), 849 (m), 835 (m), 783 (m), 648 (m), 575 (m) cm^{-1}.

HRMS (ESI): calcd for $C_{19}H_{29}O_4^-$ [M–H]$^-$: 321.2071; found: 321.2071.

NMR comparison for (2*S*,3*S*,5*S*,9*S*,10*S*,13*S*)-2,3-dihydroxy-16-nor-*ent*-pimar-8(14)-en-15-oic acid (DHPA, **29**):

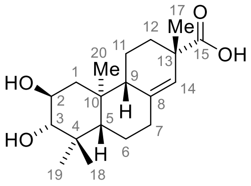

Atom number	Natural **29**[16] (400 MHz, CD3OD) δH [ppm]	Synthetic **29** (400 MHz, CD3OD) δH [ppm]	|ΔδH| [ppm]
1α	1.91 (dd, *J* = 12.2, 4.2 Hz, 1H)	1.91 (dd, *J* = 12.5, 4.2 Hz, 1H)	0.00
1β	1.13 (d, *J* = 3.2 Hz, 1H)	1.13 (m, 1H)	0.00
2	3.56 (ddd, *J* = 12.2, 9.6, 4.2 Hz, 1H)	3.57 (ddd, *J* = 11.7, 9.6, 4.2 Hz, 1H)	–0.01
3	2.95 (d, *J* = 9.6 Hz, 1H)	2.96 (d, *J* = 9.6 Hz, 1H)	–0.01
5	1.15 (m, 1H)	1.16 (m, 1H)	–0.01
6α	1.42 (dd, *J* = 12.7, 4.4 Hz, 1H)	1.42 (m, 1H)	0.00
6β	1.63 (m, 1H)	1.64 (m, 1H)	–0.01
7α	2.33 (ddd, *J* = 13.5, 4.4, 1.8 Hz, 1H)	2.35 (ddd, *J* = 14.2, 4.6, 2.1 Hz, 1H)	–0.02
7β	2.06 (td, *J* = 13.5, 5.4 Hz, 1H)	2.07 (td, *J* = 13.6, 5.4 Hz, 1H)	–0.01
9	1.79 (t, *J* = 8.6 Hz, 1H)	1.79 (t, *J* = 8.5 Hz, 1H)	0.00
11α	1.48 (td, *J* = 13.8, 3.3 Hz, 1H)	1.49 (m, 1H)	–0.01
11β	1.65 (m, 1H)	1.65 (m, 1H)	0.00
12α	1.13 (d, *J* = 3.2 Hz, 1H)	1.13 (m, 1H)	0.00
12β	2.16 (dt, *J* = 12.9, 3.5 Hz, 1H)	2.16 (dt, *J* = 12.8, 3.5 Hz, 1H)	0.00
14	5.42 (brs, 1H)	5.42 (q, *J* = 1.5 Hz, 1H)	0.00
17	1.19 (s, 3H)	1.19 (s, 3H)	0.00
18	1.02 (s, 3H)	1.02 (s, 3H)	0.00
19	0.83 (s, 3H)	0.83 (s, 3H)	0.00
20	0.78 (s, 3H)	0.78 (s, 3H)	0.00

Atom number	Natural **29**[16] (101 MHz, CD_3OD) δ_C [ppm]	Synthetic **29** (101 MHz, CD_3OD) δ_C [ppm]	$\lvert\Delta\delta_C\rvert$ [ppm]
1	46.8	46.6	0.2
2	69.4	69.2	0.2
3	84.3	84.2	0.1
4	40.8	40.6	0.2
5	55.5	55.3	0.2
6	23.5	23.3	0.2
7	36.7	36.6	0.1
8	139.6	139.5	0.1
9	52.1	52.0	0.1
10	40.2	40.1	0.1
11	21.7	21.4	0.3
12	34.4	34.3	0.1
13	44.2	44.0	0.2
14	127.8	127.6	0.2
15	181.1	180.9	0.2
17	28.3	28.2	0.1
18	29.7	29.5	0.2
19	17.7	17.6	0.1
20	15.9	15.8	0.1

A systematic shift of approximately 0.1 to 0.2 ppm between natural **29** and synthetic **29** is observed in the ^{13}C NMR spectrum, which might be due to a different CD_3OD resonance reference in the literature. The ^{1}H NMR and ^{13}C NMR data are in good agreement with the literature.

2.5.7 Norflickinflimiod A (2)

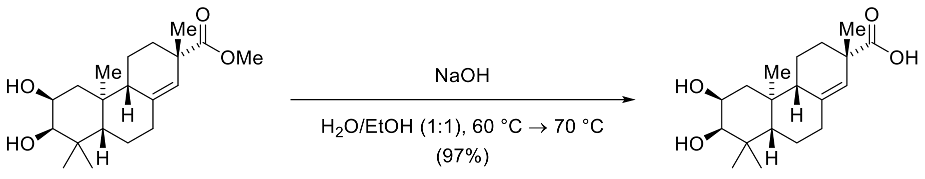

To a solution of diol **28** (6.0 mg, 18 µmol, 1 equiv) in ethanol (0.40 mL) was added an aqueous solution of sodium hydroxide (0.40 mL, 2.0 M, 0.80 mmol, 45 equiv) at 22 °C. The reaction mixture was heated to 60 °C. After 4.5 h, additional sodium hydroxide (22 mg, 0.55 mmol, 31 equiv) was added and the reaction mixture was heated to 70 °C. After 7.5 h, the colorless reaction mixture was cooled to 22 °C, a 1 M aqueous solution of hydrochloric acid (5 mL) was added, and the mixture was extracted with dichloromethane/ethyl acetate (2:1 v/v, 4 × 10 mL). The combined organic layers were dried over sodium sulfate, the dried organic layer was filtered, and the filtrate was concentrated under reduced pressure. The residue was purified by flash column chromatography on silica gel (8% methanol in dichloromethane) to yield norflickinflimiod A (**2**) (5.6 mg, 17 µmol, 97%) as a white solid.

Analytical data of norflickinflimiod A (**2**):

TLC (8% methanol in dichloromethane): R_f = 0.35 (CAM).

mp: 202–203 °C.

mp (literature)[17]: 201.5–202.5 °C.

$[\alpha]_D^{20}$ = –48.4 (c = 0.32, methanol[8]).

$[\alpha]_D^{20}$ (literature)[17] = +65.1 (c = 0.24, chloroform).

^{1}H NMR (400 MHz, CD_3OD): δ 5.41 (q, J = 1.9 Hz, 1H), 3.89 (ddd, J = 10.7, 6.1, 2.7 Hz, 1H), 3.34 (d, J = 2.6 Hz, 1H), 2.34 (ddd, J = 14.2, 4.5, 2.0 Hz, 1H), 2.16 (dtd, J = 12.9, 3.6, 1.4 Hz, 1H), 2.07 (td, J = 13.5, 5.8 Hz, 1H), 1.87 (t, J = 8.2 Hz, 1H), 1.67 (ddt, J = 14.0, 7.0, 3.6 Hz, 1H), 1.60 – 1.43 (m, 5H), 1.37 (qd, J = 12.5, 4.5 Hz, 1H), 1.19 (s, 3H), 1.13 (td, J = 13.3, 3.4 Hz, 1H), 1.00 (s, 3H), 0.89 (s, 3H), 0.78 (s, 3H).

[8] Norflickinflimiod A (**2**) was insoluble at this concentration in chloroform.

^{13}C NMR (101 MHz, CD_3OD): δ 181.1, 139.9, 127.5, 80.1, 67.3, 51.8, 48.6, 44.0, 40.9, 40.2, 39.6, 36.7, 34.3, 29.3, 28.2, 22.9, 22.7, 21.4, 15.9.

IR (ATR, neat): $\tilde{\nu}$ = 3423 (br), 2937 (s), 2872 (s), 1698 (s), 1454 (m), 1388 (m), 1271 (m), 1198 (s), 1131 (s), 1081 (m), 1040 (s), 999 (m), 985 (m), 968 (m), 948 (m), 927 (m), 884 (m), 854 (m), 837 (m), 786 (m), 751 (m), 735 (m), 672 (m), 624 (m), 547 (m) cm^{-1}.

HRMS (ESI): calcd for $C_{19}H_{29}O_4^-$ $[M–H]^-$: 321.2071; found: 321.2071.

ECD (MeCN,17 °C, 2.8 mM): λ_{max} (Δε): 235 (–0.92).

ECD (MeCN, 5.6 mM, literature)[17]: λ_{max} (Δε): 220 (–0.6), 301 (+0.01).

Comparison of the measured ECD spectrum with the ECD spectrum in ref 17 indicates that the same enantiomer has been prepared.

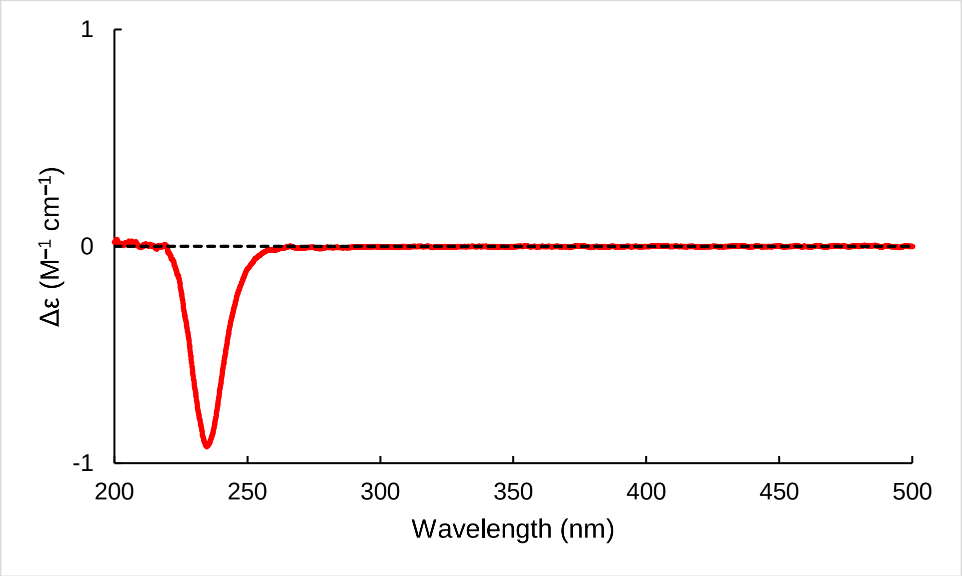

Figure 3. Experimental ECD spectrum of norflickinflimiod A (**2**).

NMR comparison for norflickinflimiod A (**2**):

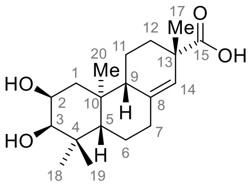

Atom number	Natural **2**[17] (400 MHz, CD_3OD) δ_H [ppm]	Synthetic **2** (400 MHz, CD_3OD) δ_H [ppm]	$\lvert\Delta\delta_H\rvert$ [ppm]
1α	1.56 (m, 1H)	1.57–1.53 (m, 2H)	—
1β	1.54 (m, 1H)		—
2	3.90 (ddd, *J* = 8.8, 6.4, 2.6 Hz, 1H)	3.89 (ddd, *J* = 10.7, 6.1, 2.7 Hz, 1H)	0.01
3	3.35 (d, *J* = 2.6 Hz, 1H)	3.34 (d, *J* = 2.6 Hz, 1H)	0.01
5	1.48 (brs, 1H)	1.47 (m, 1H)	0.01
6α (11α)*	1.45 (d, *J* = 2.0 Hz, 1H)	1.48 (m, 1H)	–0.03
6β (11β)*	1.67 (dddd, *J* = 13.6, 10.0, 6.8, 2.2 Hz, 1H)	1.67 (ddt, *J* = 14.0, 7.0, 3.6 Hz, 1H)	0.00
7α	2.33 (dd, *J* = 13.8, 2.0 Hz, 1H)	2.34 (ddd, *J* = 14.2, 4.5, 2.0 Hz, 1H)	–0.01
7β	2.07 (td, *J* = 13.8, 2.2 Hz, 1H)	2.07 (td, *J* = 13.5, 5.8 Hz, 1H)	0.00
9	1.85 (d, *J* = 6.2 Hz, 1H)	1.87 (t, *J* = 8.2 Hz, 1H)	–0.02
11α (6α)*	1.38 (td, *J* = 13.8, 2.2 Hz, 1H)	1.37 (qd, *J* = 12.5, 4.5 Hz, 1H)	0.01
11β (6β)*	1.55 (d, *J* = 6.2 Hz, 1H)	1.55 (m, 1H)	0.00
12α	1.13 (d, *J* = 3.6 Hz, 1H)	1.13 (td, *J* = 13.3, 3.4 Hz, 1H)	0.00
12β	2.16 (d, *J* = 12.6 Hz, 1H)	2.16 (dtd, *J* = 12.9, 3.6, 1.4 Hz, 1H)	0.00
14	5.41 (s, 1H)	5.41 (q, *J* = 1.9 Hz, 1H)	0.00
17	1.19 (s, 3H)	1.19 (s, 3H)	0.00
18	0.89 (s, 3H)	0.89 (s, 3H)	0.00
19	1.00 (s, 3H)	1.00 (s, 3H)	0.00
20	0.77 (s, 3H)	0.78 (s, 3H)	–0.01

*Corrected assignment in parenthesis

Atom number	Natural **2**[17] (101 MHz, CD_3OD) δ_C [ppm]	Synthetic **2** (101 MHz, CD_3OD) δ_C [ppm]	$\lvert\Delta\delta_C\rvert$ [ppm]
1	40.9	40.9	0.0
2	67.3	67.3	0.0
3	80.0	80.1	–0.1
4	39.5	39.6	–0.1
5	48.5	48.6	–0.1
6 (11)*	21.3	21.4	–0.1
7	36.6	36.7	–0.1
8	139.9	139.9	0.0
9	51.7	51.8	–0.1
10	40.2	40.2	0.0
11 (6)*	22.9	22.9	0.0
12	34.3	34.3	0.0
13	43.9	44.0	–0.1
14	127.4	127.5	–0.1
15	181.0	181.1	–0.1
17	28.2	28.2	0.0
18	22.8	22.7	0.1
19	29.3	29.3	0.0
20	15.9	15.9	0.0

*Corrected assignment in parenthesis

The ^1H NMR and ^{13}C NMR data are in good agreement with the literature.

2.6 Screenings

2.6.1 Screening of dihydroxylation to diol 17

Table S1. Screening of the dihydroxylation of geranyl arene **15**.

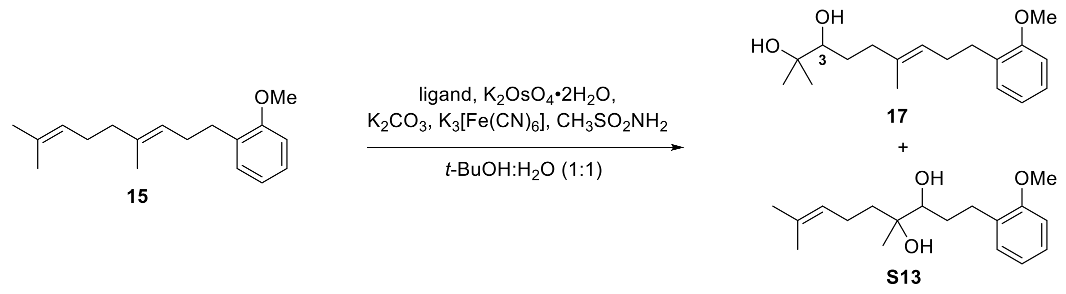

entry	scale	ligand	conc.	temp.	time	NMR yield 15	17	S13	*ee*(**17**)
1	0.38 mmol	$(DHQ)_2$PHAL (1.5 mol%)	50 mM	0 °C	61 h	21%	25%	11%	92% (3*S*)
2	0.38 mmol	$(DHQ)_2$AQN (1.5 mol%)	50 mM	0 °C	61 h	11%	20%	28%	93% (3*S*)
3[A]	0.19 mmol	$(DHQ)_2$PHAL (0.80 mol%)	150 mM	22 °C	24 h	27%	27%	13%	—
4	0.38 mmol	CNL-ligand (**S14**) (2.0 mol%)	50 mM	0 °C	61 h	14%	65%	0%	96% (3*R*)
5	68 mmol	"*ent*"-CNL-ligand (**16**) (1.5 mol%)	100 mM	0 °C	26 h	10%[B]	65%[B]	0%	93% (3*S*)

[A]Only 2.4 equiv K_2CO_3 and 2.4 equiv $K_3[Fe(CN)_6]$ used
[B]Isolated yield

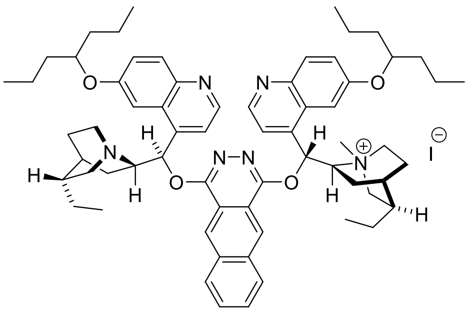

"*ent*"-Corey–Noe–Lin ligand (**16**)

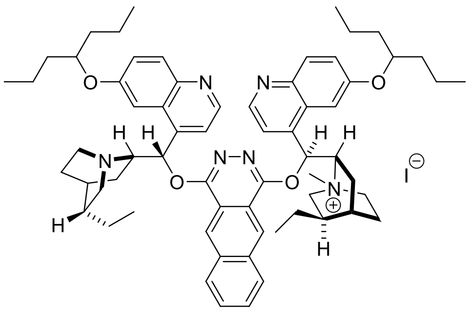

Corey–Noe–Lin ligand (**S14**)

General procedure: To a suspension of potassium carbonate (3.00 equiv), $K_3[Fe(CN)_6]$ (3.00 equiv)[9], ligand[4] (2.0–0.80 mol%, as indicated) and $K_2OsO_4{\bullet}2H_2O$ (0.5 × mol% of ligand) in water/*tert*-butanol (1:1 v/v) was added methane sulfonamide (1.00 equiv). The biphasic, red-brownish mixture was sonicated for 15 min at 22 °C, before geranyl arene **15** (1 equiv) was added at the indicated temperature. The biphasic reaction mixture was stirred vigorously at the indicated temperature until thin layer chromatography indicated a conversion >70%[10], after which sodium sulfite (10.0 equiv) was added and the reaction mixture was stirred for 30 min at 22 °C. A 1 M aqueous sodium hydroxide solution was added until all solids were dissolved, and the resulting green

[9] Prior to use, potassium carbonate and $K_3[Fe(CN)_6]$ were ground to a fine powder in a mortar.
[10] To avoid overoxidation, the reaction was discontinued before complete consumption of the starting material.

mixture was extracted with ethyl acetate (5 × same volume as reaction volume). The combined organic layers were dried over sodium sulfate, the dried solution was filtered, and the filtrate was concentrated under reduced pressure. To the residue was added 1,1,2,2-tetrachloroethane as a NMR standard and the yield was determined through NMR analysis. For characterization, diol **S13** was purified by semipreparative normal-phase high performance liquid chromatography (HPLC) (20% grading to 30% ethyl acetate in *n*-hexane over 30 min) to obtain diol **S13** as a colorless oil.

Analytical data of diol **S13**:

TLC (40% ethyl acetate in cyclohexane): R_f = 0.41 (UV, CAM).

^{1}H NMR (400 MHz, $CDCl_3$): δ 7.22 – 7.14 (m, 2H), 6.91 (td, *J* = 7.4, 1.1 Hz, 1H), 6.86 (dd, *J* = 8.1, 1.1 Hz, 1H), 5.08 (thept, *J* = 7.1, 1.4 Hz, 1H), 3.84 (s, 3H), 3.37 (ddd, *J* = 10.7, 4.0, 2.2 Hz, 1H), 2.87 – 2.73 (m, 2H), 2.63 (d, *J* = 4.0 Hz, 1H), 2.16 (s, 1H), 2.08 – 1.89 (m, 2H), 1.78 – 1.69 (m, 1H), 1.66 (d, *J* = 1.4 Hz, 3H), 1.68 – 1.59 (m, 1H), 1.57 (d, *J* = 1.4 Hz, 3H), 1.52 – 1.36 (m, 2H), 1.10 (s, 3H).

^{13}C NMR (101 MHz, $CDCl_3$): δ 157.4, 132.0, 130.4, 130.2, 127.4, 124.6, 121.0, 110.6, 75.9, 74.8, 55.6, 39.0, 32.1, 26.8, 25.8, 22.0, 21.3, 17.8.

IR (ATR, neat): $\tilde{\nu}$ = 3422 (br), 2966 (m), 2926 (m), 2856 (w), 1601 (w), 1587 (w), 1493 (s), 1463 (m), 1439 (m), 1376 (m), 1326 (w), 1290 (w), 1241 (s), 1179 (m), 1119 (m), 1073 (m), 1046 (m), 1031 (m), 933 (w), 831 (w), 787 (w), 752 (s), 574 (w), 509 (w), 465 (w) cm^{-1}.

HRMS (ESI): calcd for $C_{18}H_{28}NaO_3^+$ $[M+Na]^+$: 315.1931; found: 315.1925.

Determination of the enantiomeric excess of diol **17**:

Chiral high performance liquid chromatography (HPLC) was conducted on a Daicel Chiralpak IB column (Shimadzu LC-2030, 1 mL/min flow rate, isocratic elution, 2% *iso*-propanol in *n*-hexane, λ = 210 nm, t_R (minor) = 27.3 min, t_R (major) = 18.0 min). An approximately 1:1 mixture of diol **17** originating from either dihydroxylation with the "*ent*"-Corey–Noe–Lin ligand (**16**) (leading to (*S*)-**17**) or with the Corey–Noe–Lin ligand (**S14**) (leading to (*R*)-**17**) served as a "racemic" reference.

Diol "*rac*"-**17**:

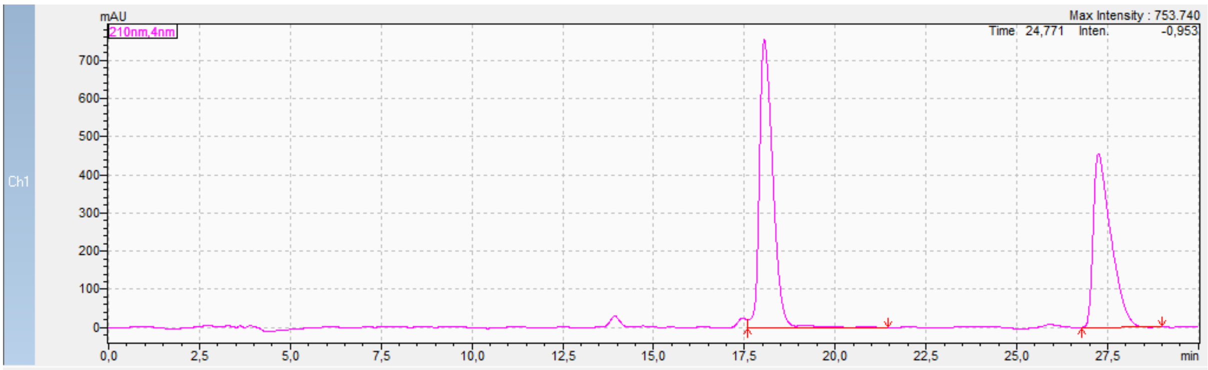

Results View - Peak Table

Peak Table | Compound | Group | Calibration Curve

Peak#	Ret. Time	Area	Height	Mark	Area%
1	18,047	19867072	754775		56,585
2	27,250	15242938	454648	M	43,415
Total		35110011	1209423		100,000

With "*ent*"-Corey–Noe–Lin ligand (**16**):

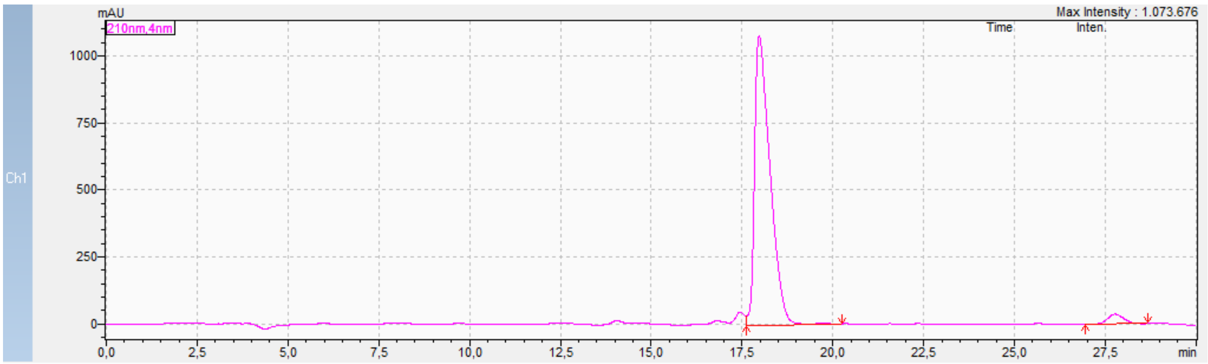

Results View - Peak Table

Peak Table | Compound | Group | Calibration Curve

Peak#	Ret. Time	Area	Height	Mark	Area%
1	17,969	32182218	1079257	M	96,746
2	27,766	1082338	36081	M	3,254
Total		33264556	1115338		100,000

With Corey–Noe–Lin ligand (**S14**):

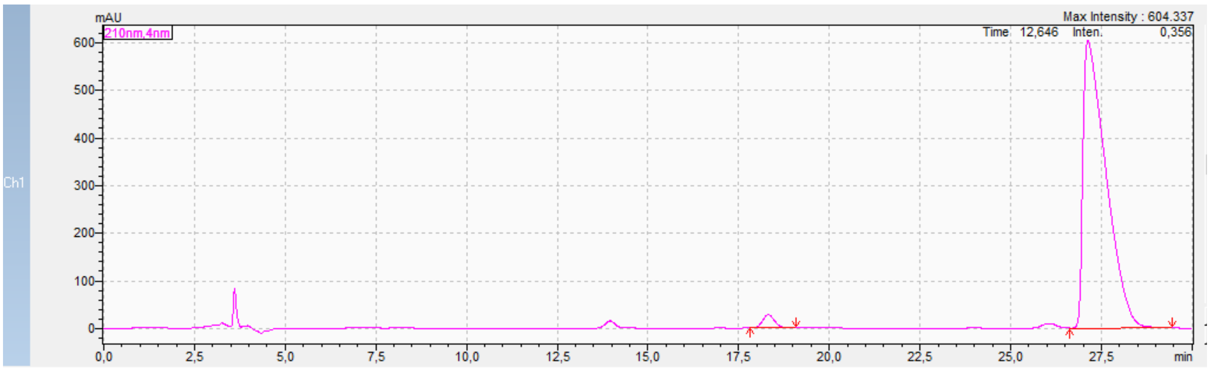

Results View - Peak Table

Peak Table | Compound | Group | Calibration Curve

Peak#	Ret. Time	Area	Height	Mark	Area%
1	18,319	590223	27162	M	2,177
2	27,136	26520232	603459	M	97,823
Total		27110454	630621		100,000

With (DHQ)2PHAL:

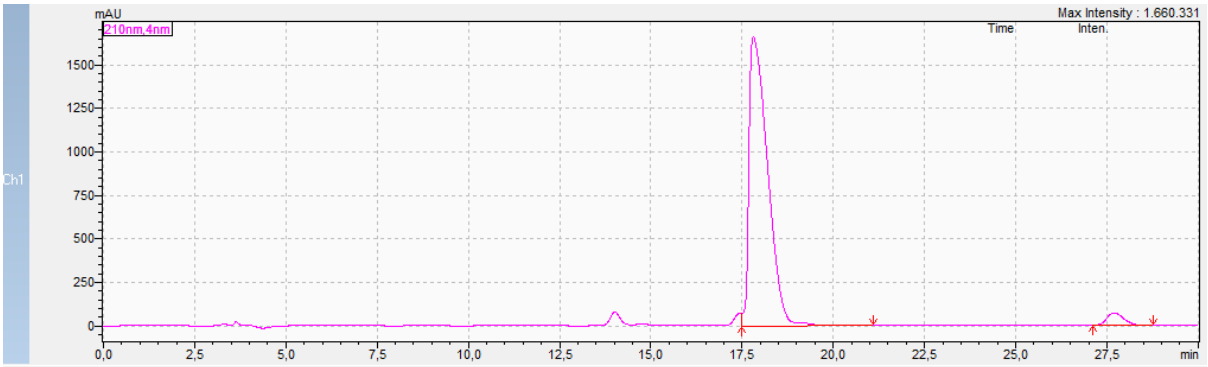

Results View - Peak Table

Peak Table | Compound | Group | Calibration Curve

Peak#	Ret. Time	Area	Height	Mark	Area%
1	17,812	58899826	1659131		96,124
2	27,696	2374771	72463		3,876
Total		61274597	1731594		100,000

With (DHQ)2AQN:

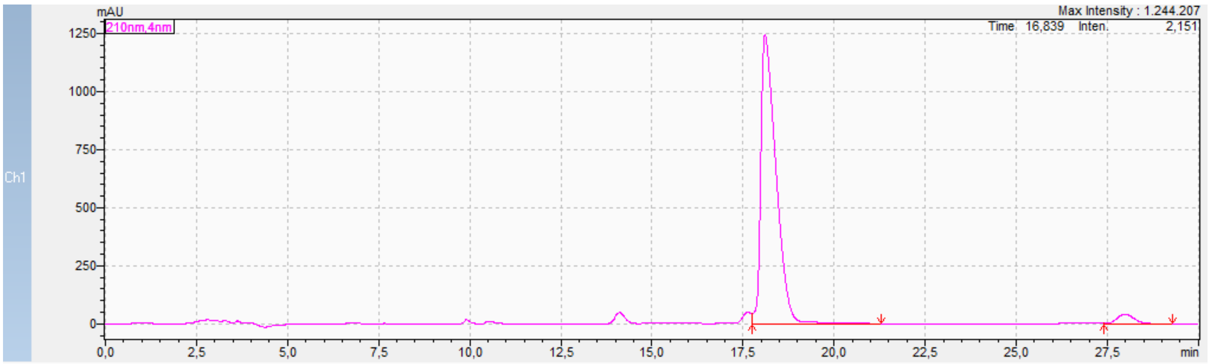

Results View - Peak Table

Peak Table | Compound | Group | Calibration Curve

Peak#	Ret. Time	Area	Height	Mark	Area%
1	18,107	36570396	1245941		96,486
2	27,980	1331766	41141		3,514
Total		37902162	1287081		100,000

2.6.2 Screening of the cationic bicyclization to tricycle **11**

Part A: Screening of literature conditions (mostly Lewis acids)[25]

Table S2. Screening of the cationic bicyclization to tricycle **11** using literature conditions (mostly Lewis acids).

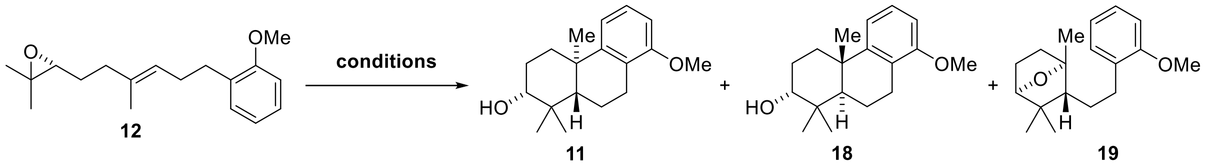

entry	scale	reagent	solvent	temp.	time	NMR yield 11	18	19
1	74 µmol	$SnCl_4$ (1.5 equiv)	CH_2Cl_2 (8 mM)	–78 °C	26 min	36%	n.d.	36%
2	77 µmol	$SnCl_4$ (1.5 equiv)	CH_2Cl_2 (8 mM)	0 °C	25 min	28%	n.d.	54%
3	78 µmol	$SnCl_4$ (1.5 equiv)	5% HFIP in CH_2Cl_2 (8 mM)	–10 °C	23 min	33%	n.d.	44%
4[A]	92 µmol	Et_2AlCl (1.5 equiv)	CH_2Cl_2 (8 mM)	–78 °C	65 min	10%	n.d.	12%
5	77 µmol	Et_2AlCl (1.5 equiv)	CH_2Cl_2 (8 mM)	0 °C	28 min	7%	n.d.	24%
6	78 µmol	$EtAlCl_2$ (1.5 equiv)	CH_2Cl_2 (8 mM)	–78 °C	60 min	25%	n.d.	37%
7	74 µmol	$EtAlCl_2$ (1.5 equiv)	CH_2Cl_2 (8 mM)	0 °C	25 min	10%	n.d.	45%
8	84 µmol	BF_3•Et_2O (2.0 equiv)	CH_2Cl_2 (55 mM)	–78 °C	58 min	25%	n.d.	28%
9	79 µmol	BF_3•Et_2O (2.0 equiv)	CH_2Cl_2 (55 mM)	0 °C	23 min	6%	n.d.	42%
10	83 µmol	$Bi(OTf)_3$ (0.10 equiv)	CH_2Cl_2 (16 mM)	22 °C	14 h	11%	n.d.	6%
11	87 µmol	$FeCl_3$ (2.0 equiv)	CH_2Cl_2 (14 mM)	22 °C	105 min	0%[B]	n.d.	0%[B]
12	73 µmol	$InBr_3$ (3.9 equiv)	CH_2Cl_2 (50 mM)	22 °C	140 min	32%	n.d.	53%
13	113 µmol	Ph_4PBF_4 (10 equiv)	HFIP (10 mM)	0 °C → 60 °C	12 min	37%	n.d.	36%

[A]63% recovered starting material **12**
[B]decomposition
n.d. = not detected

General procedure: To a solution of epoxide **12** (1 equiv) in the indicated solvent at the indicated temperature was added the indicated reagent. Reactions were monitored by thin layer chromatography and stopped after consumption of the starting material (see also indicated reaction time). For entry 1–7, the reaction was stopped through addition of triethylamine (4.5 equiv) followed by addition of a saturated aqueous solution of sodium bicarbonate (10 mL). For entry 8–9, the reaction was stopped through addition of a saturated aqueous solution of sodium bicarbonate (10 mL). For entry 10–12, the reaction was stopped through addition of water (10 mL). For entry 1–12: The organic layer was separated and the aqueous layer was extracted with dichloromethane (4 × 10 mL). The combined organic layers were dried over sodium sulfate, the dried solution was filtered, and the filtrate was concentrated under reduced pressure. To the residue was added 1,1,2,2-tetrachloroethane as a NMR standard and the yield was determined through NMR analysis. For entry 13, the reaction mixture was stirred for 12 min at 0 °C and then concentrated under atmospheric pressure (1 bar) at 60 °C until a solid precipitated. The reaction mixture was suspended in ethyl acetate (3 mL) and filtered through a silica plug, which was washed with 4 × column volumes ethyl acetate. The eluate was concentrated under reduced pressure. To the residue was

added 1,1,2,2-tetrachloroethane as a NMR standard and the yield was determined through NMR analysis.

Part B: Screening of Brønsted acids

Table S3. Screening of the cationic bicyclization to tricycle **11** using Brønsted acids.

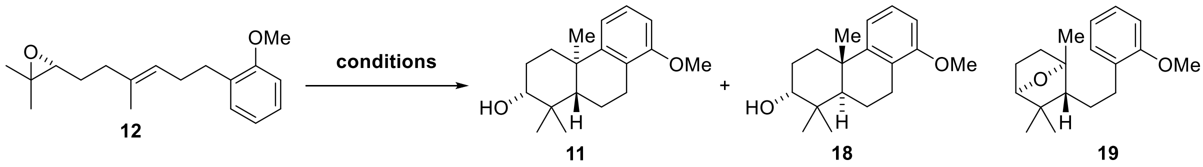

entry	scale	reagent	solvent	temp.	time	NMR yield 11	18	19
1	75 µmol	MsOH (10 mol%)	$MeNO_2$ (25 mM)	40 °C	17 h	12%	n.d.	19%
2[A]	74 µmol	MsOH (10 mol%)	MeCN (25 mM)	40 °C	17 h	1%	n.d.	2%
3	82 µmol	MsOH (10 mol%)	CH_2Cl_2 (25 mM)	40 °C	17 h	9%	n.d.	10%
4	90 µmol	TfOH (10 mol%)	CH_2Cl_2 (25 mM)	40 °C	17 h	7%	n.d.	4%
5	76 µmol	TFA (40 mol%)	HFIP (15 mM)	50 °C	15 h	34%	n.d.	47%
6	78 µmol	(+)-CSA (10 mol%)	HFIP (15 mM)	50 °C	15 h	48%	7%	25%
7	85 µmol	H_2SO_4 (10 mol%)	HFIP (15 mM)	40 °C	32 min	53%	4%	n.d.
8	89 µmol	H_2SO_4 (10 mol%)	HFIP (15 mM)	22 °C	90 min	55%	4%	n.d.
9	75 µmol	H_2SO_4 (10 mol%)	HFIP (15 mM)	0 °C	6 h	59%	7%	n.d.
10	84 µmol	TfOH (10 mol%)	HFIP (15 mM)	22 °C	28 min	59%	8%	n.d.
11	92 µmol	*p*-TsOH•H_2O (10 mol%)	HFIP (15 mM)	40 °C	90 min	64%	8%	n.d.
12	78 µmol	MsOH (10 mol%)	HFIP (15 mM)	40 °C	75 min	65%	10%	n.d.

[A]58% recovered starting material **12** n.d. = not detected

General procedure: To a solution of epoxide **12** (1 equiv) in the indicated solvent at 0 °C was added the indicated reagent. After complete addition, the reaction was stirred at the indicated temperature. Reactions were monitored by thin layer chromatography and stopped either after complete conversion of 7-oxabicyclo[2.2.1]heptane **19** (see also representative TLC image in chapter 2.1.5) or after no further conversion was observed. Reactions were stopped through addition of potassium carbonate (2 equiv) and subsequently stirred at 22 °C for at least 15 min. The reaction mixture was concentrated under reduced pressure, the residue was suspended in ethyl acetate (3 mL) and filtered through a silica plug, which was washed with 4 × column volumes ethyl acetate. To the residue was added 1,1,2,2-tetrachloroethane as a NMR standard and the yield was determined through NMR analysis.

2.6.3 Screening of the methyl ether oxidation to ketone **10**

Table S4. Screening of the methyl ether oxidation to ketone **10**.

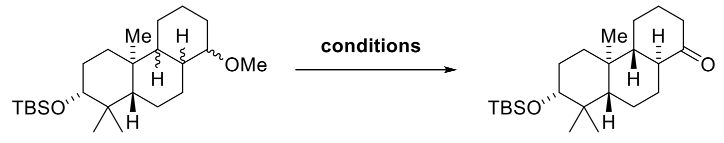

entry	scale	reagent	solvent	temp.	time	result
1	23 µmol	oxone	acetone:sat. aq. $NaHCO_3$ (1:1, 52 mM)	22 °C	5 d	traces of **10** & overoxidation
2	22 µmol	*m*-CPBA	MeCN:Cl_3CCN (1:1, 55 mM)	22 °C	24 h	26% of **10** & overoxidation
3	25 µmol	KBr, oxone	MeCN (83 mM)	22 °C	24 h	61% of **10** & overoxidation
4	22 µmol	$Ca(OCl)_2$, AcOH	acetone:H_2O (7:1, 22 mM)	22 °C	3 h	43% of **10** & overoxidation
5	24 µmol	$Ca(OCl)_2$, AcOH	acetone:H_2O (9:1, 24 mM)	22 °C	3 d	72% of **10**

Only NMR yields are given.

Entry 1 was performed according to a modified literature procedure[26] generating DMDO in situ:

To a solution of diastereomeric methyl ethers **S2** (9.2 mg, 23 µmol, 1 equiv) in acetone (300 µL) and a saturated aqueous solution of sodium bicarbonate (150 µL) was added oxone ($KHSO_5$ • 0.5 $KHSO_4$ • 0.5 K_2SO_4) (33 mg, 0.11 mmol, 4.6 equiv) at 0 °C. After 30 min at 0 °C, the reaction mixture was stirred for 17 h at 22 °C, before additional oxone ($KHSO_5$ • 0.5 $KHSO_4$ • 0.5 K_2SO_4) (15 mg, 49 µmol, 2.1 equiv) was added at 22 °C. After 25 h at 22 °C, additional oxone ($KHSO_5$ • 0.5 $KHSO_4$ • 0.5 K_2SO_4) (17 mg, 56 µmol, 2.4 equiv) was added at 22 °C and stirring was continued for 72 h at 22 °C. Next, a saturated aqueous solution of sodium sulfite (1.5 mL) was added, and the resulting mixture was extracted with ethyl acetate (4 × 2 mL). The combined organic layers were dried over sodium sulfate, the dried solution was filtered, and the filtrate was concentrated under reduced pressure. To the residue was added 1,1,2,2-tetrachloroethane as a NMR standard and the yield was determined through NMR analysis.

Entry 2 was performed according to a modified literature procedure[27]:

To a solution of diastereomeric methyl ethers **S2** (8.7 mg, 22 µmol, 1 equiv) in acetonitrile (200 µL) and trichloroacetonitrile (200 µL) was added *meta*-chloroperoxybenzoic acid (*m*-CPBA) (13 mg, 77 wt%, 59 µmol, 2.7 equiv) at 0 °C. After 30 min at 0 °C, the reaction mixture was stirred for 17 h at 22 °C, before additional *meta*-chloroperoxybenzoic acid (*m*-CPBA) (7.0 mg, 77 wt%, 31 µmol, 1.4 equiv) was added at 22 °C and stirring was continued for 7 h at 22 °C. Next, a saturated aqueous solution of sodium sulfite (1.5 mL) was added, and the resulting mixture was extracted with ethyl acetate (4 × 2 mL). The combined organic layers were dried over sodium sulfate, the dried solution

was filtered, and the filtrate was concentrated under reduced pressure. To the residue was added 1,1,2,2-tetrachloroethane as a NMR standard and the yield was determined through NMR analysis.

Entry 3 was performed according to a modified literature procedure[28]:

To a solution of diastereomeric methyl ethers **S2** (9.8 mg, 25 µmol, 1 equiv) in acetonitrile (300 µL) was added in succession potassium bromide (3.0 mg, 25 µmol, 1.0 equiv) and oxone ($KHSO_5$ • 0.5 $KHSO_4$ • 0.5 K_2SO_4) (12 mg, 39 µmol, 1.6 equiv) at 0 °C.[11] After 30 min at 0 °C, the reaction mixture was stirred for 17 h at 22 °C, before additional oxone ($KHSO_5$ • 0.5 $KHSO_4$ • 0.5 K_2SO_4) (7.4 mg, 24 µmol, 1.0 equiv) was added at 22 °C and stirring was continued for 7 h at 22 °C. Next, a saturated aqueous solution of sodium sulfite (1.5 mL) was added, and the resulting mixture was extracted with ethyl acetate (4 × 2 mL). The combined organic layers were dried over sodium sulfate, the dried solution was filtered, and the filtrate was concentrated under reduced pressure. To the residue was added 1,1,2,2-tetrachloroethane as a NMR standard and the yield was determined through NMR analysis.

Entry 4 & 5 were performed according to a modified literature procedure[7]:

Entry 4: To a solution of diastereomeric methyl ethers **S2** (8.7 mg, 22 µmol, 1 equiv) in acetone (875 µL) and water (125 µL) was added successively acetic acid (11 µL, 0.20 µmol, 8.9 equiv) and calcium hypochlorite (28 mg, 67 wt%, 0.13 mmol, 6.0 equiv) at 22 °C. After stirring for 3 h at 22 °C, a saturated aqueous solution of sodium sulfite (1.5 mL) was added, and the resulting mixture was extracted with ethyl acetate (4 × 2 mL). The combined organic layers were dried over sodium sulfate, the dried solution was filtered, and the filtrate was concentrated under reduced pressure. To the residue was added 1,1,2,2-tetrachloroethane as a NMR standard and the yield was determined through NMR analysis.

Entry 5: To a solution of diastereomeric methyl ethers **S2** (9.4 mg, 24 µmol, 1 equiv) in acetone (900 µL) and water (100 µL) was added successively acetic acid (6.0 µL, 0.11 µmol, 4.4 equiv) and calcium hypochlorite (9.9 mg, 67 wt%, 46 µmol, 1.9 equiv) at 0 °C. The turbid white suspension was stirred for 8 h at 0 °C and then kept for 3 d at 0 °C without stirring. Next, a saturated aqueous solution of sodium sulfite (1.5 mL) was added, and the resulting mixture was extracted with ethyl acetate (4 × 2 mL). The combined organic layers were dried over sodium sulfate, the dried solution

[11] The reaction vessel must not be protected from light.

was filtered, and the filtrate was concentrated under reduced pressure. To the residue was added 1,1,2,2-tetrachloroethane as a NMR standard and the yield was determined through NMR analysis.

2.6.4 Screening of the α-methylation of ketone 10

Table S5. Screening of the α-methylation of ketone **10**.

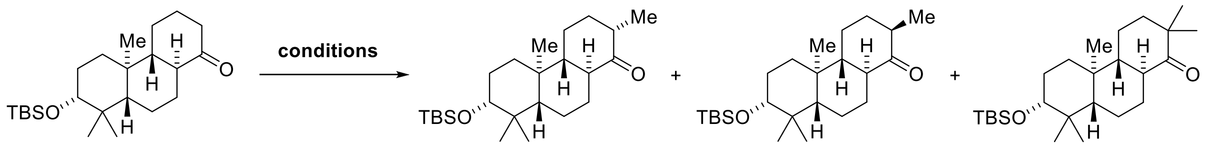

10 **20** **21** **S15**

entry	scale	reagents	solvent	temp.	time	yield 10	yield 20	yield 21	yield S15
1*	132 µmol	LiHMDS, *then* MeI	Et_2O (44 mM)	−55 °C → 22 °C	20 h	11%	30%	35%	12%
2	1.32 mmol	LiHMDS, *then* MeI	THF (44 mM)	−55 °C → 22 °C	14 h	3%	48%	48%	n.d.

*NMR yield. n.d. = not detected

General procedure: To a solution of ketone **10** (1 equiv) in the indicated solvent was added a solution of lithium bis(trimethylsilyl)amide in tetrahydrofuran (1.00 M, 1.15 equiv) at –55 °C, whereupon the colorless solution turned yellow. The solution was warmed up over 1 h 50 min from –55 °C to –38 °C, after which the reaction mixture was cooled to –50 °C and methyl iodide (5.00 equiv) was added. The reaction mixture was allowed to warm up to 22 °C through removal of the cooling bath. After stirring for the indicated time, excess of methyl iodide was removed through addition of triethylamine (5.00 equiv) and stirring for 15 min at 22 °C, during which a white precipitate formed. A saturated aqueous solution of ammonium chloride (1.5-fold solvent volume) was added, and the mixture was extracted with diethyl ether (4 × 1.5-fold solvent volume). The combined organic layers were washed with a saturated aqueous solution of sodium chloride (1.5-fold solvent volume) the washed solution was dried over sodium sulfate, the dried organic layer was filtered, and the filtrate was concentrated under reduced pressure. Entry 1: To the residue was added 1,1,2,2-tetrachloroethane as a NMR standard and the yield was determined through NMR analysis. Entry 2: The residue was purified by flash column chromatography on silica gel (7% diethyl ether in *n*-pentane) to yield recovered starting material **10** and a diastereomeric mixture of ketone **20** and ketone **21** (d.r. = 1:1). For characterization of the dimethylated ketone **S15**, a reference sample was purified by flash column chromatography on silica gel (0.5% diethyl ether and 20% dichloromethane in cyclohexane) to yield ketone **S15** as a white solid.

<u>Analytical data of ketone **S15**:</u>

TLC (10% diethyl ether in *n*-pentane): R_f = 0.68 (CAM).

mp: 105–106 °C.

$[\alpha]_D^{20}$ = +26.4 (*c* = 1.11, dichloromethane).

^{1}**H NMR** (400 MHz, $CDCl_3$): δ 3.16 (dd, *J* = 11.1, 4.6 Hz, 1H), 2.45 (td, *J* = 11.6, 4.1 Hz, 1H), 1.98 – 1.90 (m, 1H), 1.78 – 1.64 (m, 3H), 1.63 – 1.47 (m, 4H), 1.47 – 1.39 (m, 1H), 1.34 – 1.22 (m, 2H), 1.13 (s, 3H), 1.09 – 0.98 (m, 2H), 1.01 (s, 3H), 0.95 (s, 3H), 0.88 (s, 12H), 0.76 (s, 3H), 0.77 – 0.71 (m, 1H), 0.03 (s, 3H), 0.03 (s, 3H).

^{13}**C NMR** (101 MHz, $CDCl_3$): δ 217.7, 79.4, 57.6, 53.9, 44.8, 44.5, 40.2, 39.6, 37.4, 37.4, 28.8, 28.1, 27.2, 26.1, 25.6, 25.2, 20.8, 20.3, 18.3, 16.2, 14.1, –3.7, –4.8.

IR (ATR, neat): $\tilde{\nu}$ = 2934 (m), 2853 (m), 1704 (s), 1472 (m), 1461 (m), 1386 (m), 1363 (m), 1296 (w), 1252 (m), 1206 (w), 1118 (m), 1085 (s), 1071 (s), 1028 (w), 1007 (w), 992 (w), 969 (w), 940 (w), 885 (m), 862 (m), 834 (s), 773 (s), 670 (w), 588 (w), 546 (w), 490 (w) cm^{-1}.

HRMS (ESI): calcd for $C_{25}H_{46}NaO_2Si^+$ $[M+Na]^+$: 429.3159; found: 429.3188.

2.6.5 Screening of the C-acylation of ketone **20** and **21**

Table S6. Screening of the C-acylation of ketone **20** and **21**.

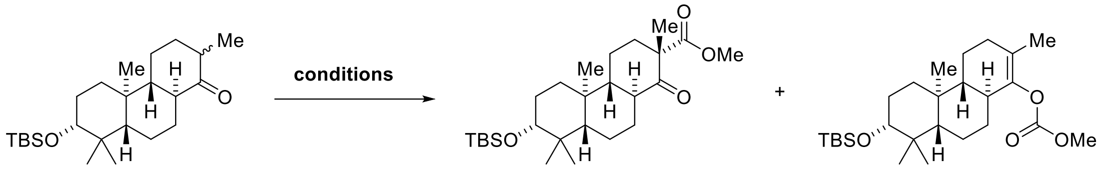

20 & **21** (d.r. = 1:1) → **22** + **S16**

entry	scale	deprotonation	C-acylation	solvent	NMR yield 22	NMR yield S16
1	64 µmol	LDA, 0 °C, 3 h	Mander's reagent (6 equiv), −78 °C → 0 °C, 80 min	THF	27%	6%
2	64 µmol	LDA, TMEDA, 0 °C, 3 h	Mander's reagent (6 equiv), −78 °C → 0 °C, 80 min	THF	44%	28%
3	64 µmol	LDA, TMEDA, 0 °C, 3 h	Mander's reagent (6 equiv), −78 °C → 0 °C, 80 min	Et_2O	50%	23%
4	64 µmol	LDA, 0 °C, 3 h	Mander's reagent (6 equiv), −78 °C → 0 °C, 80 min	Et_2O	45%	n.d.
5	64 µmol	LDA, 0 °C, 3 h	Mander's reagent (1.5 equiv), −78 °C, 80 min	Et_2O	66%	n.d.
6	64 µmol	LDA, 0 °C, 2 h	Mander's reagent (1.5 equiv), −78 °C, 80 min	Et_2O	55%	n.d.
7	64 µmol	LDA, −15 °C, 3 h	Mander's reagent (1.5 equiv), −78 °C, 80 min	Et_2O	62%	n.d.

n.d. = not detected

General procedure: To a solution of diisopropylamine (1.50 equiv for entry 1–4, 1.30 equiv for entry 5–7) in the indicated solvent (0.300 M) with or without *N*,*N*,*N*,*N*-tetramethyl ethylenediamine (TMEDA) (2.8 equiv) was added a solution of *n*-butyllithium in hexanes (2.50 M,1.40 equiv for entry 1–4, 1.20 equiv for entry 5–7) dropwise at –78 °C. After complete addition, the reaction mixture was warmed to 0 °C and stirred for 45 min. Next, the thusly generated lithium diispropylamide solution was added dropwise to a solution of ketone **20** and ketone **21** (d.r. = 1:1, 1 equiv) in the indicated solvent (1.5 mL, 0.043 M) at the indicated temperature, whereupon the colorless solution turned bright yellow. After stirring for the indicated time at the indicated temperature, the reaction mixture was cooled to –78 °C and methyl cyanoformate (Mander's reagent) (6.0 equiv for entry 1–4, 1.50 equiv for entry 5–7) was added dropwise over 60 sec.[8] After stirring for the indicated time with the indicated temperature profile, water (2 mL) was added at the indicated temperature and the mixture was allowed to warm up to 22 °C. The mixture was extracted with diethyl ether (4 × 2 mL). The combined organic layers were dried over sodium sulfate, the dried organic layer was filtered, and the filtrate was concentrated under reduced pressure. To the residue was added 1,1,2,2-tetrachloroethane as a NMR standard and the yield was determined through NMR analysis. For characterization of the O-acylation product **S16**, a reference sample was purified by flash column chromatography on silica gel (3% grading 4.5% diethyl ether in *n*-pentane) to yield carbonate **S16** as a white solid.

Analytical data of carbonate **S16**:

TLC (10% diethyl ether in *n*-pentane): R_f = 0.54 (CAM).

mp: 89–90 °C.

$[\alpha]_D^{20}$ = +25 (*c* = 0.43, dichloromethane).

^{1}H NMR (400 MHz, $CDCl_3$): δ 3.82 (s, 3H), 3.17 (dd, *J* = 11.1, 4.7 Hz, 1H), 2.35 (t, *J* = 11.4 Hz, 1H), 2.12 – 1.97 (m, 2H), 1.94 (dq, *J* = 12.6, 3.6 Hz, 1H), 1.72 – 1.59 (m, 4H), 1.57 – 1.49 (m, 4H), 1.36 – 1.18 (m, 2H), 1.07 – 0.94 (m, 3H), 0.89 (s, 9H), 0.87 (s, 3H), 0.84 – 0.79 (m, 4H), 0.74 (s, 3H), 0.04 (s, 3H), 0.03 (s, 3H).

^{13}C NMR (101 MHz, $CDCl_3$): δ 154.1, 145.0, 120.7, 79.6, 55.2, 54.5, 54.1, 39.6, 37.1, 36.9, 36.4, 30.8, 29.9, 28.6, 28.1, 26.1 (3C), 21.4 (2C), 18.3, 16.0 (2C), 14.4, –3.6, –4.8.

IR (ATR, neat): $\tilde{\nu}$ = 2934 (m), 2855 (w), 1757 (s), 1702 (w), 1472 (w), 1461 (w), 1441 (m), 1387 (w), 1361 (w), 1263 (s), 1238 (s), 1203 (w), 1156 (w), 1126 (w), 1097 (m), 1069 (m), 1051 (m), 1001 (w), 973 (w), 936 (m), 887 (m), 873 (w), 836 (s), 815 (w), 773 (m), 733 (w), 669 (w) cm^{-1}.

HRMS (ESI): calcd for $C_{26}H_{46}NaO_4Si^+$ $[M+Na]^+$: 473.3058; found: 473.3044.

2.7 Alternative route of β-ketoester **22** to alkene **9**

2.7.1 Secondary alcohol **S17**

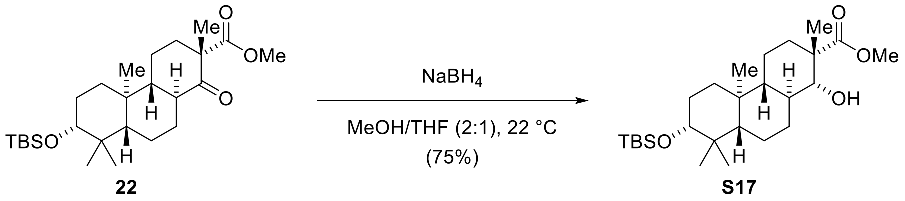

To a solution of β-ketoester **22** (37.6 mg, 83.4 µmol, 1 equiv) in methanol (4.00 mL) and tetrahydrofuran (2.00 mL) was added sodium borohydride (12.0 mg, 317 µmol, 3.80 equiv) at 22 °C. The slightly turbid reaction mixture was stirred for 20 min at 22 °C, after which water (1 mL) and a saturated aqueous solution of ammonium chloride (4 mL) were added consecutively (caution: strong gas development!). The mixture was extracted with dichloromethane (4 × 10 mL), the combined organic layers were dried over sodium sulfate, the dried organic layer was filtered, and the filtrate was concentrated under reduced pressure. The residue was purified by flash column chromatography on silica gel (18% diethyl ether in *n*-pentane) to yield secondary alcohol **S17** (28.2 mg, 62.3 µmol, 75%) as a white solid.

Analytical data of secondary alcohol **S17**:

TLC (20% diethyl ether in *n*-pentane): R_f = 0.31 (CAM).

mp: 146–147 °C.

$[\alpha]_D^{20}$ = +27.2 (*c* = 1.07, dichloromethane).

^{1}H NMR (400 MHz, $CDCl_3$): δ 3.71 (s, 3H), 3.16 (dd, *J* = 10.9, 4.8 Hz, 1H), 3.14 (d, *J* = 11.6 Hz, 1H), 2.77 (dd, *J* = 11.6, 10.1 Hz, 1H), 2.38 (dq, *J* = 13.2, 3.7 Hz, 1H), 2.20 (dt, *J* = 13.5, 3.2 Hz, 1H), 1.69 – 1.54 (m, 3H), 1.53 – 1.40 (m, 3H), 1.35 – 1.23 (m, 4H), 1.07 (td, *J* = 13.4, 3.1 Hz, 1H), 1.01 – 0.83 (m, 15H), 0.79 (dd, *J* = 12.4, 2.5 Hz, 1H), 0.76 (s, 3H), 0.74 (s, 3H), 0.70 (td, *J* = 11.5, 3.1 Hz, 1H), 0.02 (s, 6H).

^{13}C NMR (101 MHz, $CDCl_3$): δ 178.4, 82.7, 79.6, 54.8, 54.5, 51.9, 48.1, 40.9, 39.6, 37.4, 36.5, 35.6, 31.6, 28.8, 28.1, 26.1 (3C), 25.2, 22.0, 21.3, 18.3, 16.2, 14.5, –3.6, –4.8.

IR (ATR, neat): $\tilde{\nu}$ = 2934 (m), 2855 (w), 1757 (s), 1702 (w), 1472 (w), 1461 (w), 1441 (m), 1387 (w), 1361 (w), 1263 (s), 1238 (s), 1203 (w), 1156 (w), 1126 (w), 1097 (m), 1069 (m), 1051 (m), 1001 (w), 973 (w), 936 (m), 887 (m), 873 (w), 836 (s), 815 (w), 773 (m), 733 (w), 669 (w) cm^{-1}.

HRMS (ESI): calcd for $C_{26}H_{48}NaO_4Si^+$ $[M+Na]^+$: 475.3214; found: 475.3213.

2.7.2 Alkene **9**

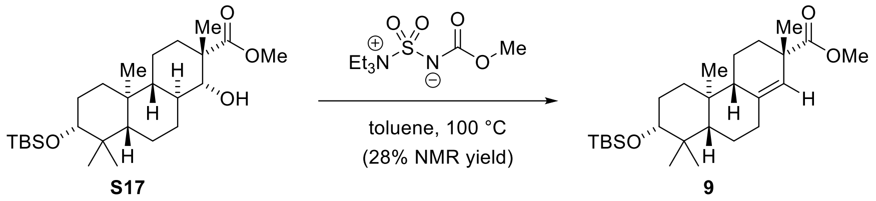

To a solution of secondary alcohol **S17** (9.3 mg, 21 µmol, 1 equiv) in toluene (4.00 mL) was added Burgess reagent (9.8 mg, 41 µmol, 2.0 equiv) at 22 °C. The colorless solution was stirred at 100 °C for 8 h, after which additional Burgess reagent (7.7 mg, 32 µmol, 1.6 equiv) was added. After stirring for further 14 h at 100 °C, the now yellow reaction mixture was concentrated under reduced pressure. The residue was purified by flash column chromatography on silica gel (3% grading to 5% diethyl ether in *n*-pentane) to yield alkene **9** (2.5 mg, 5.7 µmol, 28% NMR yield)[12] along with inseparable side products.[13]

[12] NMR yield was determined through addition of 1,1,2,2-tetrachloroethane as a NMR standard.
[13] Alternative elimination with Martin's sulfurane gave lower yields.

2.8 Alternative route of secondary alcohol **S3** to diol **28**

2.8.1 Disubstituted alkene **S18**

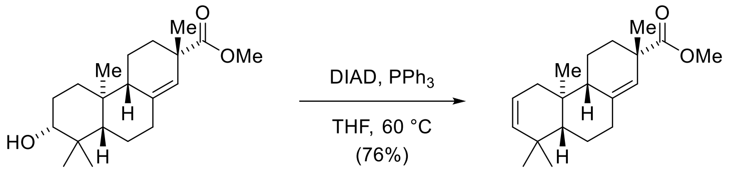

S3 **S18**

To a solution secondary alcohol **S3** (50.1 mg, 156 µmol, 1 equiv) and triphenylphosphine (205 mg, 782 µmol, 5.00 equiv) in tetrahydrofuran (2.50 mL) was added diisopropyl azodicarboxylate (DIAD) (154 µL, 782 µmol, 5.00 equiv) at 22 °C. The reaction mixture was heated to 60 °C for 5 h 15 min, during which the initially yellow solution turned to a yellow suspension and eventually to a red solution. After cooling to 22 °C, the reaction mixture was partitioned between water (20 mL) and diethyl ether (60 mL). The organic layer was separated and successively washed with an aqueous hydrogen peroxide solution (10 wt%, 2 × 20 mL) and a saturated aqueous solution of sodium chloride (20 mL). The washed solution was dried over sodium sulfate, the dried organic layer was filtered, and the filtrate was concentrated under reduced pressure. The residue was purified by flash column chromatography on silica gel (0% grading to 3% diethyl ether in *n*-pentane) to yield disubstituted alkene **S18** (35.8 mg, 118 µmol, 76%) as a white solid.

Analytical data of disubstituted alkene **S18**:

TLC (3% diethyl ether in *n*-pentane): R_f = 0.37 (CAM).

mp: 36–38 °C.

$[\alpha]_D^{20}$ = –37.5 (*c* = 1.19, dichloromethane).

^{1}H NMR (400 MHz, $CDCl_3$): δ 5.47 (ddd, *J* = 10.0, 6.0, 2.0 Hz, 1H), 5.41 – 5.32 (m, 2H), 3.64 (s, 3H), 2.34 (ddd, *J* = 13.8, 4.2, 2.2 Hz, 1H), 2.16 (dtd, *J* = 12.8, 3.9, 1.4 Hz, 1H), 2.03 (tdt, *J* = 13.3, 5.2, 1.6 Hz, 1H), 1.90 (dd, *J* = 16.9, 6.1 Hz, 1H), 1.81 – 1.71 (m, 2H), 1.67 – 1.56 (m, 2H), 1.55 – 1.41 (m, 2H), 1.38 (dd, *J* = 12.2, 2.9 Hz, 1H), 1.20 (s, 3H), 1.16 (td, *J* = 13.0, 3.8 Hz, 1H), 0.97 (s, 3H), 0.88 (s, 3H), 0.70 (s, 3H).

^{13}C NMR (101 MHz, $CDCl_3$): δ 177.8, 139.3, 138.0, 125.1, 121.4, 51.8, 51.3, 49.1, 43.1, 38.4, 38.0, 35.8, 34.7, 33.5, 32.2, 27.5, 24.0, 23.8, 19.9, 13.6.

IR (ATR, neat): $\tilde{\nu}$ = 3009 (w), 2954 (m), 2932 (m), 2870 (w), 2831 (w), 1731 (s), 1664 (w), 1453 (m), 1433 (m), 1396 (w), 1378 (w), 1361 (w), 1340 (w), 1264 (m), 1246 (m), 1199 (s), 1162 (m), 1130 (m), 1114 (m), 1089 (w), 1065 (w), 1037 (w), 1019 (w), 996 (w), 968 (w), 941 (w), 885 (w), 855 (w), 843 (w), 803 (w), 769 (w), 729 (m), 709 (w), 664 (w), 638 (w), 582 (w), 511 (w), 456 (w) cm^{-1}.

HRMS (ESI): calcd for $C_{20}H_{30}NaO_2^+$ $[M+Na]^+$: 303.2319; found: 303.2307.

2.8.2 Screening of the dihydroxylation of disubstituted alkene **S18**

Table S7. Screening of the dihydroxylation of disubstituted alkene **S18**.

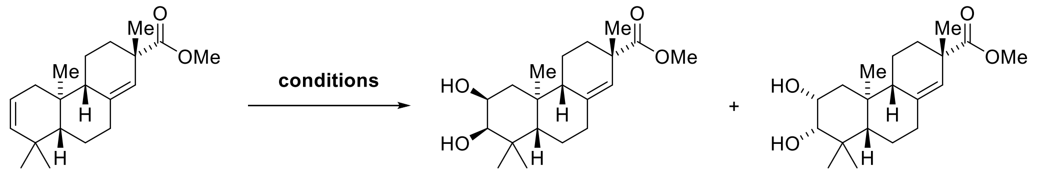

entry	scale	reagents	ligand	temp.	time	NMR yield S18	NMR yield 28	NMR yield 27
1	6.6 µmol	K_2OsO_4•$2H_2O$, NMO	citric acid	22 °C	11 d	n.d.	8%	18%
2	6.6 µmol	OsO_4, NMO	—	22 °C	11 d	77%	4%	8%
3	6.6 µmol	OsO_4, NMO	$(DHQD)_2$PHAL	22 °C	11 d	34%	n.d.	49%
4	6.6 µmol	OsO_4, NMO	$(DHQ)_2$PHAL	22 °C	11 d	71%	n.d.	6%
5*	6.6 µmol	OsO_4	$(DHQ)_2$PHAL	22 °C	30 h	n.d.	11%	12%
6*	5.6 µmol	OsO_4	DHQ-IND	22 °C	30 h	n.d.	8%	10%
7	15 µmol	a) AgOAc, I_2, *then* H_2O<br>b) KOH, MeOH	—<br>—	22 °C → 95 °C<br>22 °C	6.5 h<br>15 h	—<br>—	—<br>n.d.	—<br>n.d.

*Solvents were degassed through Ar bubbling under sonication for at least 30 min   n.d. = not detected

Sharpless dihydroxylation conditions in the presence of citric acid[29] (entry 1) and the Woodward dihydroxylation[30] (entry 7) suffered from low yields due to insufficient regioselectivity between the disubstituted and the trisubstituted alkenes. In contrast — without degassed solvents (entry 2–4) — osmium-catalyzed dihydroxylation in the presence of *N*-methylmorpholine *N*-oxide and an optional ligand exhibited slow conversion rates. Although full conversion of disubstituted olefine **S18** was observed in degassed solvents, low yields of the desired diol **28** were obtained. Interestingly, substrate-control favors the undesired diol **27** and employing (DHQD)2PHAL (entry 3) as a ligand represents a matched case of diastereoselection affording exclusively diol **27**. Unfortunately, the mismatched case with (DHQ)2PHAL (entry 4 & 5) gave at best approx. a 1:1 mixture of diol **28** and diol **27**. Also, indolinylcarbamoyl ligands such as DHQD-IND and DHQ-

IND[31] — especially designed for *cis*-disubstituted alkenes — did not result in a significant improvement (entry 6).

<u>Experimental procedures:</u>

Entry 1 was performed according to a modified literature procedure[29]: To a solution of disubstituted olefine **S18** (2.0 mg, 6.6 µmol, 1 equiv), citric acid monohydrate (4.4 mg, 21 µmol, 3.2 equiv) and *N*-methylmorpholine *N*-oxide (NMO) (1.1 mg, 9.4 µmol, 1.4 equiv) in acetonitrile (1.00 mL) and water (250 µL) was added $K_2OsO_4{\cdot}2H_2O$ (0.2 mg, 0.5 µmol, 8 mol%) at 22 °C. The yellow-greenish solution was stirred for 4 days at 22 °C, after which additional *N*-methylmorpholine *N*-oxide (NMO) (0.9 mg, 8 µmol, 1.2 equiv) was added and stirring was continued for 7 days at 22 °C. Next, a saturated aqueous solution of sodium sulfite (2.0 mL) was added, the resulting mixture was stirred for 10 min at 22 °C and then extracted with dichloromethane (3 × 10 mL). The combined organic layers were dried over sodium sulfate, the dried solution was filtered, and the filtrate was concentrated under reduced pressure. To the residue was added 1,1,2,2-tetrachloroethane as a NMR standard and the yield was determined through NMR analysis.

Entry 2–4: To a solution of disubstituted olefine **S18** (2.0 mg, 6.6 µmol, 1 equiv) and the indicated ligand (1.2 equiv) in acetone (1.00 mL) and water (250 µL) was added a solution of osmium tetroxide in water (4.0 wt%, 50 µL, 7.9 µmol, 1.2 equiv) at 22 °C. The initially colorless solution was stirred for 4 days at 22 °C, after which *N*-methylmorpholine *N*-oxide (NMO) (0.9 mg, 8 µmol, 1.2 equiv) was added to the blackish suspension and stirring was continued for 7 days at 22 °C. Next, a saturated aqueous solution of sodium sulfite (2.0 mL) was added, the resulting mixture was stirred for 10 min at 22 °C and then extracted with dichloromethane (3 × 10 mL). The combined organic layers were dried over sodium sulfate, the dried solution was filtered, and the filtrate was concentrated under reduced pressure. To the residue was added 1,1,2,2-tetrachloroethane as a NMR standard and the yield was determined through NMR analysis.

Entry 5–6: To a solution of disubstituted olefine **S18** (2.0 mg, 6.6 µmol, 1 equiv) and the indicated ligand[14] (1.2 equiv) in acetone (200 µL) and water (40 µL)[15] was added a solution of osmium tetroxide in *tert*-butanol (2.5 wt%, 0.11 mL, 8.6 µmol, 1.3 equiv) at 22 °C. The yellow/orange solution was stirred for 30 h at 22 °C, during which the reaction mixture turned to a blackish suspension. Next, a saturated aqueous solution of sodium sulfite (2.0 mL) was added, the resulting mixture was stirred for 10 min at 22 °C and then extracted with ethyl acetate (4 × 2 mL). The combined organic layers were dried over sodium sulfate, the dried solution was filtered, and the filtrate was concentrated under reduced pressure. To the residue was added 1,1,2,2-tetrachloroethane as a NMR standard and the yield was determined through NMR analysis.

Entry 7 was performed according to a literature procedure[30]: To a solution of disubstituted olefine **S18** (4.4 mg, 15 µmol, 1 equiv) in acetic acid (260 µL) was added silver acetate (6.1 mg, 37 µmol, 2.5 equiv) and iodine (4.3 mg, 17 µmol, 1.2 equiv) sequentially at 22 °C. The yellow suspension was stirred for 2.5 h at 22 °C, after which a solution of water in acetic acid (1.0 M, 15 µL, 15 µmol, 1.0 equiv) was added. After stirring was continued for 4 h at 95 °C, the reaction mixture was cooled to 22 °C and sodium chloride (10 mg, 0.17 mmol, 12 equiv) was added. The reaction mixture was filtered through celite, and the filter cake was washed with ethyl acetate (3 × 5 mL). The filtrate was washed with a saturated aqueous solution of sodium hydrogencarbonate (2 × 10 mL), the washed solution was dried over sodium sulfate, the dried solution was filtered, and the filtrate was concentrated under reduced pressure. The residue was dissolved in methanol (500 µL) and potassium hydroxide (11 mg, 0.20 mmol, 13 equiv) was added at 22 °C. After stirring for 15 h at 22 °C, a 1 M aqueous solution of hydrochloric acid (1.5 mL) was added, and the mixture was extracted with ethyl acetate (4 × 2 mL). The combined organic layers were dried over sodium sulfate, the dried solution was filtered, and the filtrate was concentrated under reduced pressure. To the residue was added 1,1,2,2-tetrachloroethane as a NMR standard and the yield was determined through NMR analysis.

[14] DHQD-IND has been reported to give high enantioselectivities for the dihydroxylation of *cis*-disubstituted olefins.[31]
[15] Both solvents were degassed by argon bubbling for at least 30 min under simultaneous sonication.

2.8.3 Synthesis of DHQ-IND

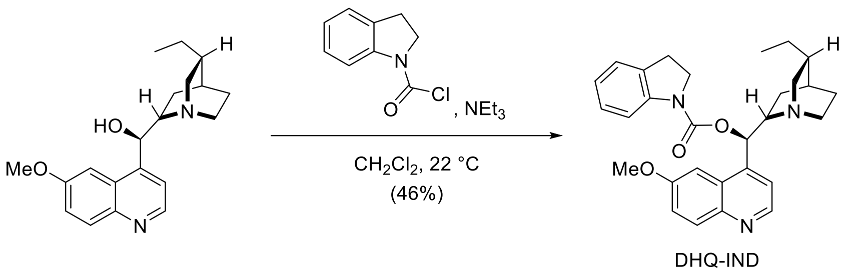

DHQ-IND was prepared according to a modified literature procedure[31]: To a solution of indolinyl-carbamylchloride (2.24 g, 12.3 mmol, 2.04 equiv) (freshly prepared according to a reported literature procedure[31]) in dry dichloromethane (40 mL) was added in succession triethylamine (3.36 mL, 24.1 mmol, 4.00 equiv) and hydroquinine (**S7**) (2.24 g, 97 wt%, 6.03 mmol, 1 equiv) at 22 °C. The brown solution was stirred at 22 °C for 3 days, after which the reaction mixture was concentrated under reduced pressure. The residue was purified by flash column chromatography on silica gel (concentrated aqueous ammonia : methanol : dichloromethane (NH_4OH : MeOH : CH_2Cl_2), 1:5:94 grading to 1:6:93) to yield DHQ-IND (1.32 g, 2.80 mmol, 46%) as an off-white solid.

Analytical data of DHQ-IND:

TLC (1% concentrated aqueous ammonia and 5% methanol in dichloromethane): R_f = 0.29 (UV).

mp: 79–80 °C.

$[\alpha]_D^{20}$ = +99.5 (*c* = 0.971, dichloromethane).

^{1}H NMR (600 MHz, DMSO-*d*$_6$, 75 °C): δ 8.71 (d, *J* = 4.5 Hz, 1H), 7.96 (d, *J* = 9.2 Hz, 1H), 7.63 (d, *J* = 8.0 Hz, 1H), 7.58 (d, *J* = 2.8 Hz, 1H), 7.54 (d, *J* = 4.5 Hz, 1H), 7.43 (dd, *J* = 9.2, 2.8 Hz, 1H), 7.21 (dd, *J* = 7.4, 1.4 Hz, 1H), 7.13 (t, *J* = 7.8 Hz, 1H), 6.95 (td, *J* = 7.4, 1.1 Hz, 1H), 6.39 (d, *J* = 8.2 Hz, 1H), 4.12 (s, 2H), 3.94 (s, 3H), 3.46 – 3.39 (m, 1H), 3.14 (s, 2H), 2.87 (dd, *J* = 13.5, 8.7 Hz, 1H), 2.55 – 2.48 (m, *J* = 2.6 Hz, 1H), 2.21 (d, *J* = 13.5 Hz, 1H), 1.93 (ddd, *J* = 12.0, 8.6, 4.3 Hz, 1H), 1.77 (qd, *J* = 4.7, 2.8 Hz, 1H), 1.72 (ddt, *J* = 11.9, 10.2, 4.2 Hz, 1H), 1.55 (dd, *J* = 13.6, 7.7 Hz, 1H), 1.45 – 1.29 (m, 5H), 0.84 (t, *J* = 7.1 Hz, 3H).

^{13}C NMR (151 MHz, DMSO-*d*$_6$, 75 °C): δ 157.0, 151.4, 147.2, 144.3, 143.8, 141.5, 131.2, 131.0, 126.8, 126.6, 124.7, 122.4, 120.9, 118.9, 113.7, 102.2, 74.6, 59.2, 57.3, 55.3, 47.1, 41.5, 36.9, 27.9, 26.8, 26.6, 24.7, 24.3, 11.6.

IR (ATR, neat): $\tilde{\nu}$ = 2930 (m), 2863 (w), 1702 (s), 1620 (m), 1594 (w), 1508 (m), 1486 (s), 1463 (m), 1432 (m), 1403 (s), 1360 (m), 1334 (m), 1316 (m), 1296 (m), 1263 (m), 1226 (s), 1171 (m), 1136 (s), 1085 (m), 1032 (s), 1014 (s), 911 (m), 851 (m), 754 (s), 729 (s), 684 (w), 644 (m), 620 (w), 598 (w), 529 (w), 494 (w), 468 (w), 423 (w) cm^{-1}.

HRMS (ESI): calcd for $C_{29}H_{34}N_3O_3^+$ $[M–I^-]^+$: 472.2595; found: 472.2585.

2.9 Absolute configuration of tricycle 18

2.9.1 Synthesis of (*R*)-Mosher ester S19

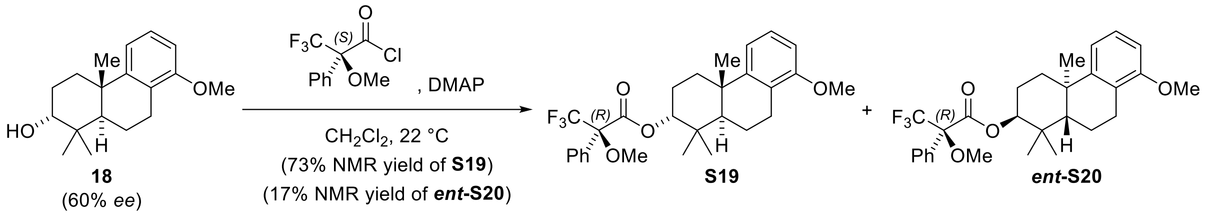

(*R*)-Mosher ester **S19** was prepared according to a modified literature procedure[32]: To a solution of tricycle **18** (20.0 mg, 72.9 µmol, 1 equiv) and *N*,*N*-dimethylpyridin-4-amine (DMAP) (51.1 mg, 418 µmol, 5.74 equiv) in dry dichloromethane (1.30 mL) was added (*S*)-(+)-α-methoxy-α-(trifluoromethyl)phenylacetyl chloride (36.8 mg, 146 µmol, 2.00 equiv) at 22 °C. The slightly yellowish solution was stirred at 22 °C for 5 h 30 min. The reaction mixture was filtered through a silica plug, which was washed with 4 × column volumes of 30% ethyl acetate in *n*-pentane. The filtrate was concentrated under reduced pressure, to the residue was added 1,1,2,2-tetrachloroethane as a NMR standard and the yield was determined through NMR analysis. For characterization, (*R*)-Mosher ester **S19** was purified by semipreparative normal-phase high performance liquid chromatography (HPLC) (2% grading to 5% ethyl acetate in *n*-hexane over 30 min) to obtain (*R*)-Mosher ester **S19** as an amorphous white solid. (*R*)-Mosher ester ***ent*-S20** was identified through NMR comparison with its enantiomer (*S*)-Mosher ester **S20**.

Analytical data of (*R*)-Mosher ester **S19**:

TLC (3% ethyl acetate in cyclohexane): R_f = 0.28 (UV, CAM).

$[\alpha]_D^{20}$ = +4.4 (*c* = 0.34, dichloromethane).

^{1}H NMR (400 MHz, $CDCl_3$): δ 7.49 – 7.45 (m, 2H), 7.34 – 7.28 (m, 3H), 7.09 (t, J = 8.0 Hz, 1H), 6.77 (dd, J = 8.1, 1.0 Hz, 1H), 6.64 (dd, J = 8.1, 1.0 Hz, 1H), 4.93 (t, J = 2.8 Hz, 1H), 3.81 (s, 3H), 3.47 (q, J = 1.3 Hz, 3H), 2.87 (dd, J = 17.5, 5.8 Hz, 1H), 2.59 – 2.47 (m, 1H), 2.09 (tdd, J = 14.4, 3.9, 2.3 Hz, 1H), 1.96 (dt, J = 13.2, 3.5 Hz, 1H), 1.88 (dq, J = 15.2, 3.4 Hz, 1H), 1.80 (dd, J = 11.5, 7.3 Hz, 1H), 1.71 – 1.57 (m, 2H), 1.48 (td, J = 13.7, 3.8 Hz, 1H), 1.18 (s, 3H), 1.03 (s, 3H), 0.94 (s, 3H).

^{13}C NMR (101 MHz, $CDCl_3$): δ 166.4, 157.1, 150.6, 132.4, 129.6, 128.4 (2C), 127.6 (q, J = 1.4 Hz, 2C), 126.2, 123.9, 123.6* (d, J = 288.4 Hz), 116.4, 106.6, 84.9* (d, J = 27.8 Hz), 81.6, 55.6 (q, J = 1.5 Hz),

55.3, 44.4, 37.5, 37.1, 32.0, 28.3, 24.7, 24.3, 23.5, 22.0, 18.0. (The signals marked with * should be quartets, but the outer resonance signals are missing due to their low intensity.)

IR (ATR, neat): $\tilde{\nu}$ = 3067 (w), 2946 (m), 2839 (w), 1741 (s), 1580 (w), 1459 (m), 1437 (m), 1392 (w), 1370 (w), 1343 (w), 1254 (s), 1169 (s), 1123 (m), 1082 (w), 1067 (m), 1046 (m), 1016 (m), 993 (m), 949 (w), 926 (w), 899 (w), 881 (w), 854 (w), 827 (w), 779 (w), 765 (w), 719 (m), 698 (w), 666 (w), 639 (w), 605 (w), 523 (w) cm^{-1}.

HRMS (ESI): calcd for $C_{28}H_{33}F_3NaO_4^+$ [M+Na]$^+$: 513.2223; found: 513.2224.

2.9.2 Synthesis of (*S*)-Mosher ester **S20**

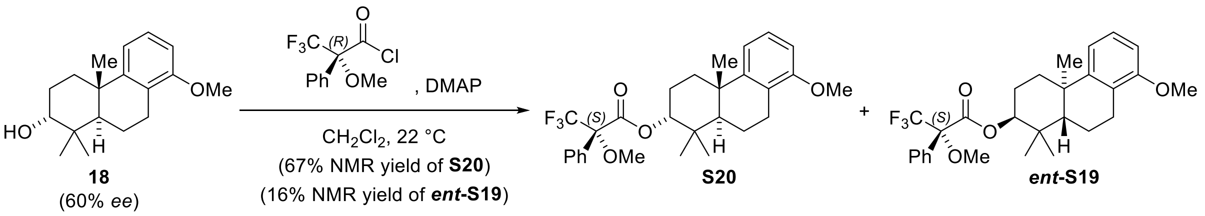

(*S*)-Mosher ester **S20** was prepared according to a modified literature procedure[32]: To a solution of tricycle **18** (20.0 mg, 72.9 µmol, 1 equiv) and *N*,*N*-dimethylpyridin-4-amine (DMAP) (51.1 mg, 418 µmol, 5.74 equiv) in dry dichloromethane (1.30 mL) was added (*R*)-(–)-α-methoxy-α-(trifluoromethyl)phenylacetyl chloride (36.8 mg, 146 µmol, 2.00 equiv) at 22 °C. The slightly yellowish solution was stirred at 22 °C for 5 h 30 min. The reaction mixture was filtered through a silica plug, which was washed with 4 × column volumes of 30% ethyl acetate in *n*-pentane. The filtrate was concentrated under reduced pressure, to the residue was added 1,1,2,2-tetrachloroethane as a NMR standard and the yield was determined through NMR analysis. For characterization, (*S*)-Mosher ester **S20** was purified by semipreparative normal-phase high performance liquid chromatography (HPLC) (1% grading to 5% ethyl acetate in *n*-hexane over 30 min) to obtain (*S*)-Mosher ester **S20** as a white solid. (*S*)-Mosher ester ***ent*-S19** was identified through NMR comparison with its enantiomer (*R*)-Mosher ester **S19**.

<u>Analytical data of (*S*)-Mosher ester **S20**:</u>

TLC (3% ethyl acetate in cyclohexane): R_f = 0.29 (UV, CAM).

mp: 188–189 °C.

$[\alpha]_D^{20}$ = –33.8 (c = 0.794, dichloromethane).

^{1}H NMR (400 MHz, $CDCl_3$): δ 7.53 – 7.46 (m, 2H), 7.34 – 7.28 (m, 3H), 7.12 (t, J = 8.0 Hz, 1H), 6.84 (dd, J = 8.1, 1.1 Hz, 1H), 6.65 (dd, J = 8.1, 1.0 Hz, 1H), 4.99 (t, J = 2.7 Hz, 1H), 3.81 (s, 3H), 3.51 (q, J = 1.4 Hz, 3H), 2.86 (dd, J = 18.3, 5.3 Hz, 1H), 2.59 – 2.46 (m, 1H), 2.20 – 2.05 (m, 2H), 1.91 (dq, J = 13.9, 2.9, 2.4 Hz, 1H), 1.80 – 1.72 (m, 1H), 1.71 – 1.57 (m, 3H), 1.20 (s, 3H), 1.01 (s, 3H), 0.77 (s, 3H).

^{13}C NMR (101 MHz, $CDCl_3$): δ 166.3, 157.1, 150.6, 132.7, 129.5, 128.4 (2C), 127.4 (q, J = 1.4 Hz, 2C), 126.2, 124.1, 123.6* (d, J = 288.9 Hz), 116.5, 106.6, 84.5 (q, J = 27.6 Hz), 81.1, 55.6 (q, J = 1.8 Hz), 55.3, 44.4, 37.5, 37.3, 32.4, 27.8, 24.8, 24.3, 23.8, 22.0, 18.0. (The signal marked with * should be a quartet, but the outer resonance signals are missing due to their low intensity.)

IR (ATR, neat): $\tilde{\nu}$ = 3068 (w), 2947 (m), 2838 (w), 1741 (s), 1580 (w), 1458 (m), 1437 (m), 1393 (w), 1370 (w), 1343 (w), 1254 (s), 1167 (s), 1124 (m), 1082 (w), 1067 (m), 1046 (m), 1017 (m), 993 (m), 949 (w), 900 (w), 882 (w), 853 (w), 827 (w), 779 (w), 766 (w), 719 (m), 697 (w), 644 (w), 606 (w), 504 (w), 457 (w) cm^{-1}.

HRMS (ESI): calcd for $C_{28}H_{33}F_3NaO_4^+$ $[M+Na]^+$: 513.2223; found: 513.2225.

2.9.3 Mosher ester analysis of **S19** and **S20**

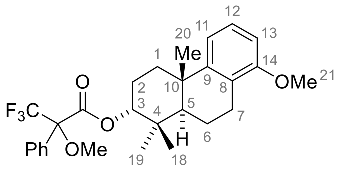

Atom number	(*S*)-Mosher ester **S20** (400 MHz, $CDCl_3$) δ_H [ppm]	(*R*)-Mosher ester **S19** (400 MHz, $CDCl_3$) δ_H [ppm]	$\lvert\Delta\delta^{S-R}\rvert$ [ppm]
1β	1.66 (m, 1H)	1.48 (td, J = 13.7, 3.8 Hz, 1H)	+0.18
1α	2.09 (m, 1H)	1.96 (dt, J = 13.2, 3.5 Hz, 1H)	+0.13
11	6.84 (dd, J = 8.1, 1.1 Hz, 1H)	6.77 (dd, J = 8.1, 1.0 Hz, 1H)	+0.07
2α	2.15 (m, 1H)	2.09 (tdd, J = 14.4, 3.9, 2.3 Hz, 1H)	+0.06
2β	1.91 (dq, J = 13.9, 2.9, 2.4 Hz, 1H)	1.88 (dq, J = 15.2, 3.4 Hz, 1H)	+0.03
12	7.12 (t, J = 8.0 Hz, 1H)	7.09 (t, J = 8.0 Hz, 1H)	+0.03
20	1.20 (s, 3H)	1.18 (s, 3H)	+0.02
13	6.65 (dd, J = 8.1, 1.0 Hz, 1H)	6.64 (dd, J = 8.1, 1.0 Hz, 1H)	+0.01
21	3.81 (s, 3H)	3.81 (s, 3H)	0.00
3	4.99 (t, J = 2.7 Hz, 1H)	4.93 (t, J = 2.8 Hz, 1H)	—
5	1.61 (m, 1H)	1.62 (m, 1H)	–0.01
7α	2.86 (dd, J = 18.3, 5.3 Hz, 1H)	2.87 (dd, J = 17.5, 5.8 Hz, 1H)	–0.01
7β	2.53 (m, 1H)	2.54 (m, 1H)	–0.01
18	1.01 (s, 3H)	1.03 (s, 3H)	–0.02
6β	1.61 (m, 1H)	1.64 (m, 1H)	–0.03
6α	1.76 (m, 1H)	1.80 (dd, J = 11.5, 7.3 Hz, 1H)	–0.04
19	0.77 (s, 3H)	0.94 (s, 3H)	–0.17

Based on the shift differences $\Delta\delta^{S-R}$, the absolute stereochemistry of tricycle **18** was assigned to be (3*R*).[32]

3. X–ray

3.1 Tricycle **11**

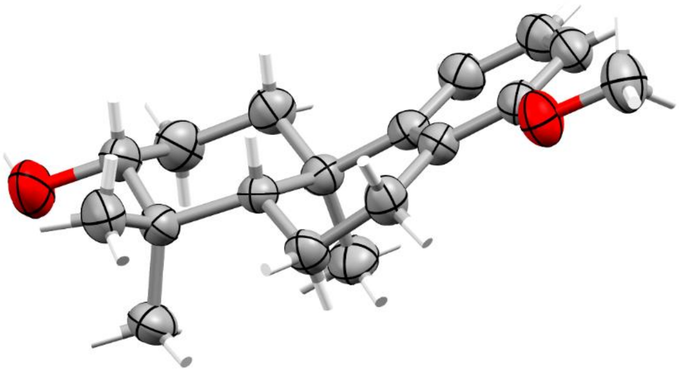

Thermal ellipsoids are shown at the 50% probability level.

Identification code	Tricycle **11**
Empirical formula	$C_{18}H_{26}O_2$
Formula weight	274.39
Temperature	300.00 K
Wavelength	0.71073 Å
Crystal system	Monoclinic
Space group	C2 (no. 5)
Unit cell dimensions	a = 20.3155(10) Å α = 90°.<br>b = 7.6380(4) Å β = 115.225(2)°.<br>c = 11.0166(6) Å γ = 90°.
Volume	1546.43(14) Å^3
Z	4
Density (calculated)	1.179 Mg/m^3
Absorption coefficient	0.075 mm^{-1}
F(000)	600
Crystal size	0.21 x 0.18 x 0.12 mm^3
Theta range for data collection	2.216 to 25.988°.
Index ranges	-24<=h<=24, -9<=k<=9, -13<=l<=13
Reflections collected	21409
Independent reflections	3037 [R(int) = 0.0278]
Completeness to theta = 25.242°	99.6 %
Absorption correction	Semi-empirical from equivalents
Max. and min. transmission	0.9588 and 0.9468
Refinement method	Full-matrix least-squares on F^2
Data / restraints / parameters	3037 / 1 / 187
Goodness-of-fit on F^2	1.057
Final R indices [I>2sigma(I)]	R1 = 0.0308, wR2 = 0.0851
R indices (all data)	R1 = 0.0322, wR2 = 0.0866

Absolute structure parameter	-0.1(3)
Extinction coefficient	0.010(3)
Largest diff. peak and hole	0.147 and -0.102 e.Å^{-3}

3.2 Tricycle **18**

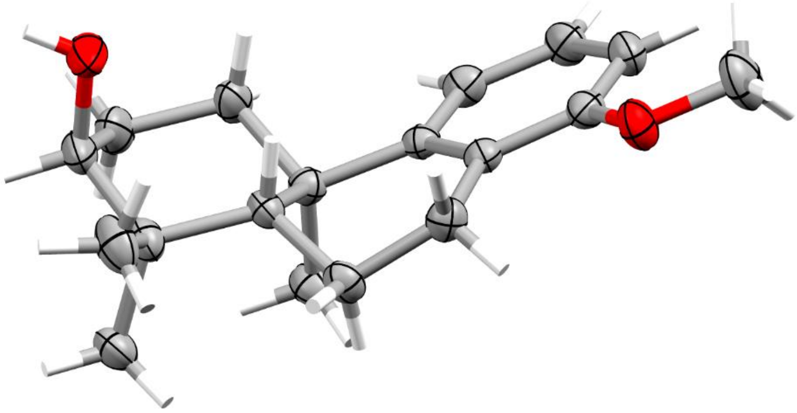

Thermal ellipsoids are shown at the 50% probability level.

Identification code	Tricycle **18**
Empirical formula	$C_{18}H_{26}O_2$
Formula weight	274.39
Temperature	183.00 K
Wavelength	0.71073 Å
Crystal system	Tetragonal
Space group	I-4 (no. 88)
Unit cell dimensions	a = 20.4174(5) Å α = 90°.<br>b = 20.4174(5) Å β = 90°.<br>c = 7.2316(2) Å γ = 90°.
Volume	3014.64(17) Å^3
Z	8
Density (calculated)	1.209 Mg/m^3
Absorption coefficient	0.076 mm^{-1}
F(000)	1200
Crystal size	0.21 x 0.18 x 0.06 mm^3
Theta range for data collection	2.822 to 25.992°.
Index ranges	-25<=h<=25, -25<=k<=24, -8<=l<=8
Reflections collected	20520
Independent reflections	2961 [R(int) = 0.0416]
Completeness to theta = 25.242°	99.8 %
Absorption correction	Semi-empirical from equivalents
Max. and min. transmission	0.9583 and 0.9045

Refinement method	Full-matrix least-squares on F^2
Data / restraints / parameters	2961 / 0 / 186
Goodness-of-fit on F^2	1.025
Final R indices [I>2sigma(I)]	R1 = 0.0344, wR2 = 0.0859
R indices (all data)	R1 = 0.0359, wR2 = 0.0875
Absolute structure parameter	0.6(5)
Extinction coefficient	n/a
Largest diff. peak and hole	0.137 and -0.143 e.$Å^{-3}$

3.3 Norflickinflimiod C (**5**)

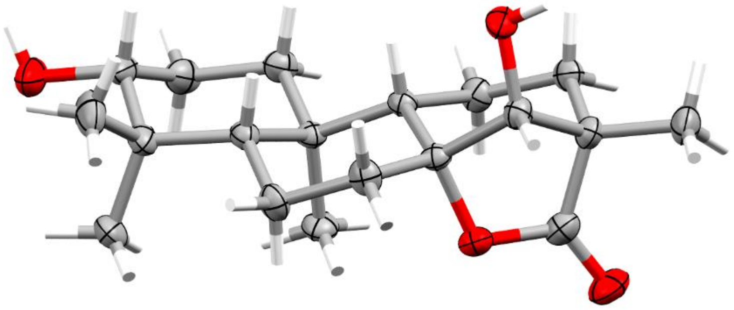

Thermal ellipsoids are shown at the 50% probability level.

Identification code	Norflickinflimiod C (**5**)
Empirical formula	$C_{19}H_{30}O_4 \times 0.5\ H_2O$
Formula weight	331.44
Temperature	173.00 K
Wavelength	0.71073 Å
Crystal system	Orthorhombic
Space group	$P2_12_12_1$ (no. 19)
Unit cell dimensions	a = 12.8340(3) Å α = 90°.<br>b = 13.6532(4) Å β = 90°.<br>c = 20.3029(5) Å γ = 90°.
Volume	3557.58(16) $Å^3$
Z	8
Density (calculated)	1.238 Mg/m^3
Absorption coefficient	0.086 mm^{-1}
F(000)	1448
Crystal size	0.31 x 0.19 x 0.17 mm^3
Theta range for data collection	2.006 to 27.509°.
Index ranges	-16<=h<=16, -17<=k<=17, -25<=l<=26
Reflections collected	80775
Independent reflections	8163 [R(int) = 0.0414]
Completeness to theta = 25.242°	99.9 %
Absorption correction	Semi-empirical from equivalents
Max. and min. transmission	0.9590 and 0.9364
Refinement method	Full-matrix least-squares on F^2
Data / restraints / parameters	8163 / 6 / 456
Goodness-of-fit on F^2	1.027
Final R indices [I>2sigma(I)]	R1 = 0.0304, wR2 = 0.0792
R indices (all data)	R1 = 0.0319, wR2 = 0.0806
Absolute structure parameter	-0.15(16)
Extinction coefficient	n/a
Largest diff. peak and hole	0.243 and -0.199 e.$Å^{-3}$

4. NMR Spectra

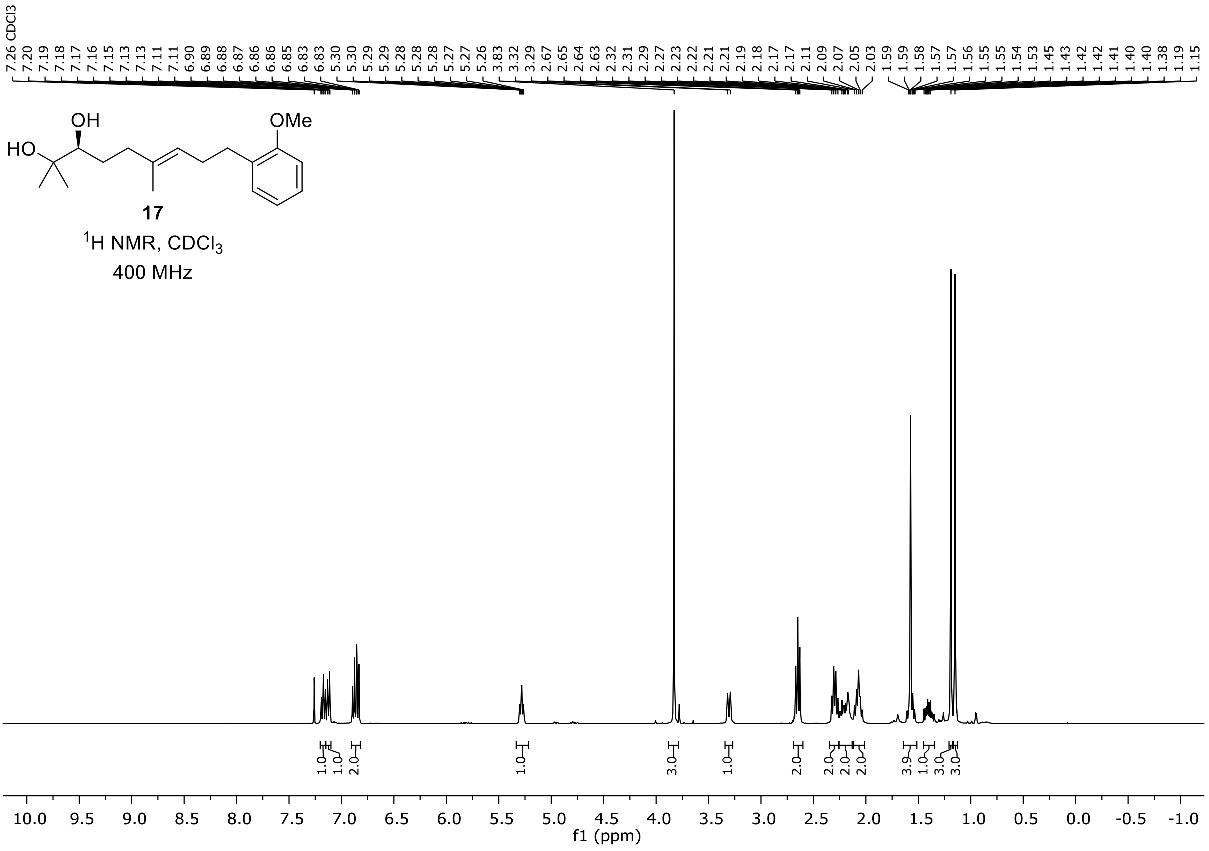

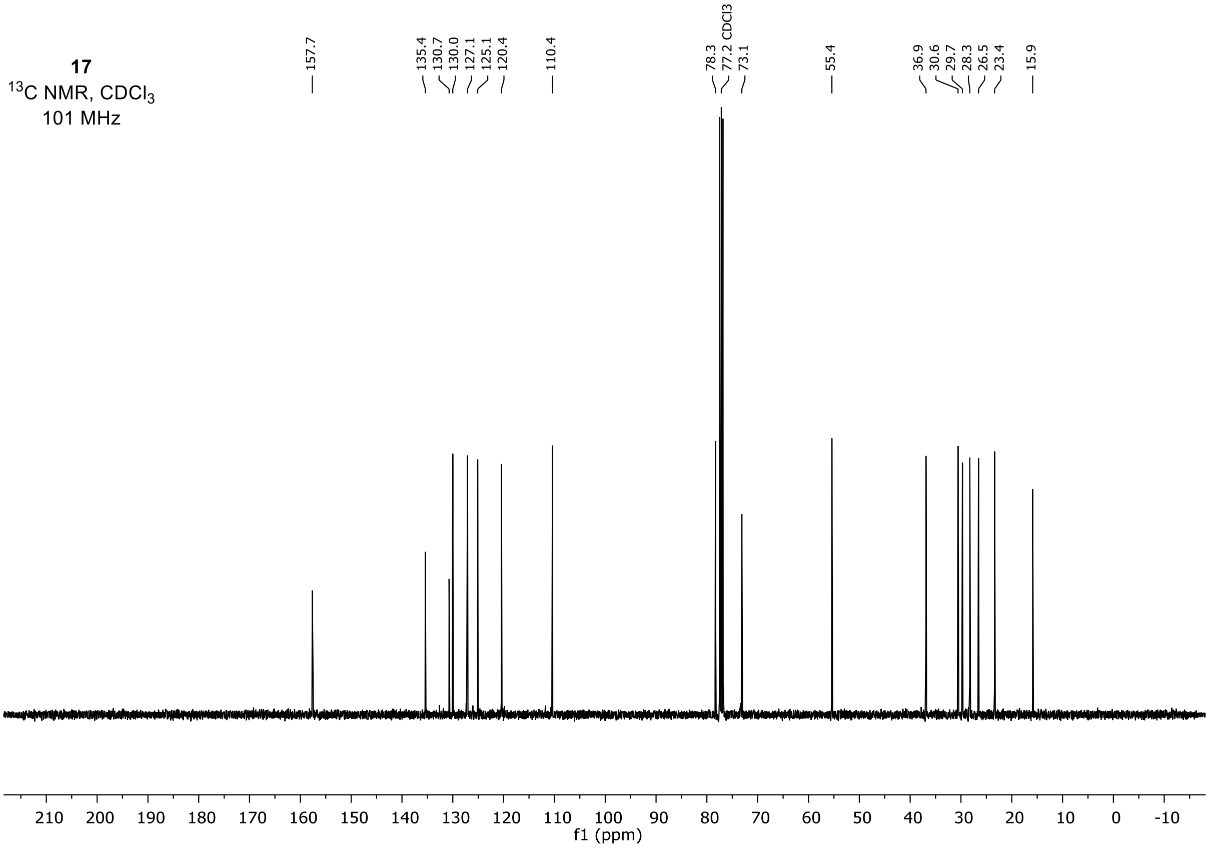

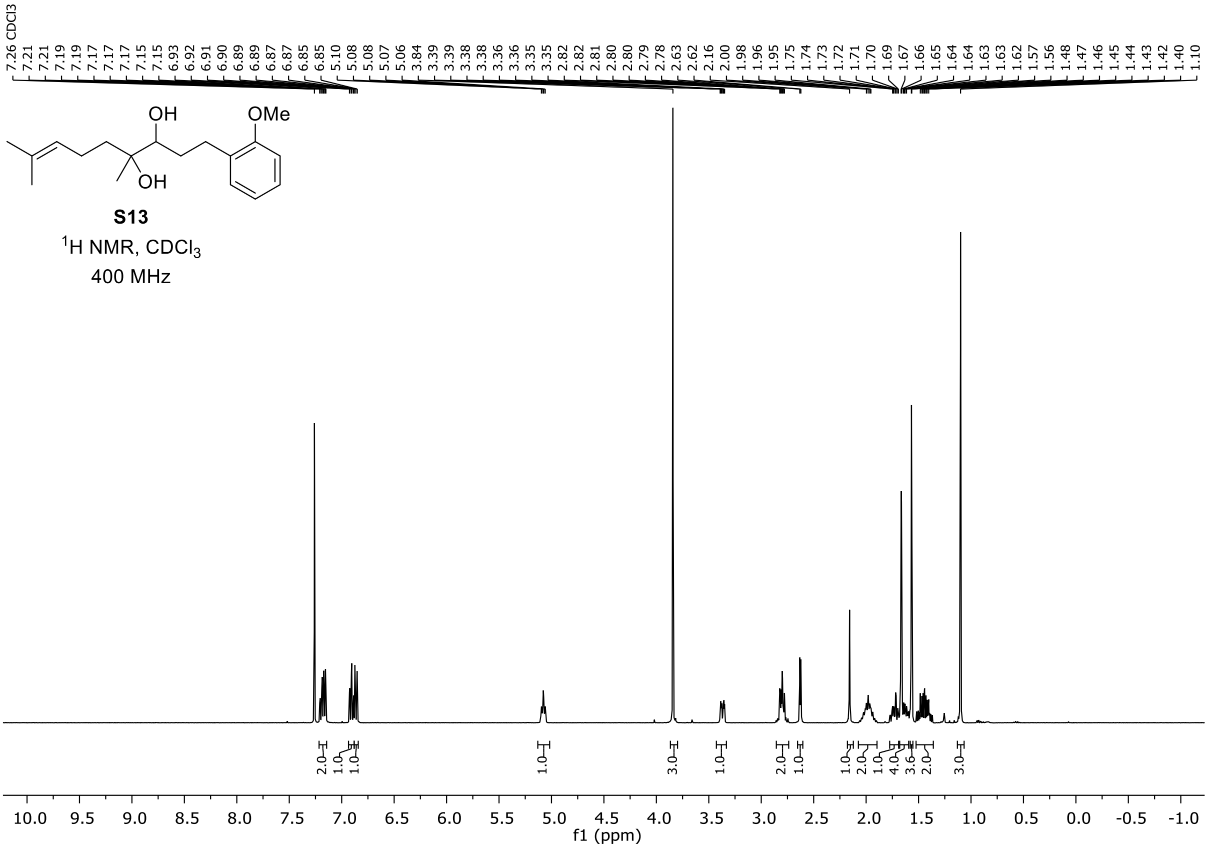
OH
OMe
OH
S13
1H NMR, CDCl3
400 MHz
f1 (ppm)

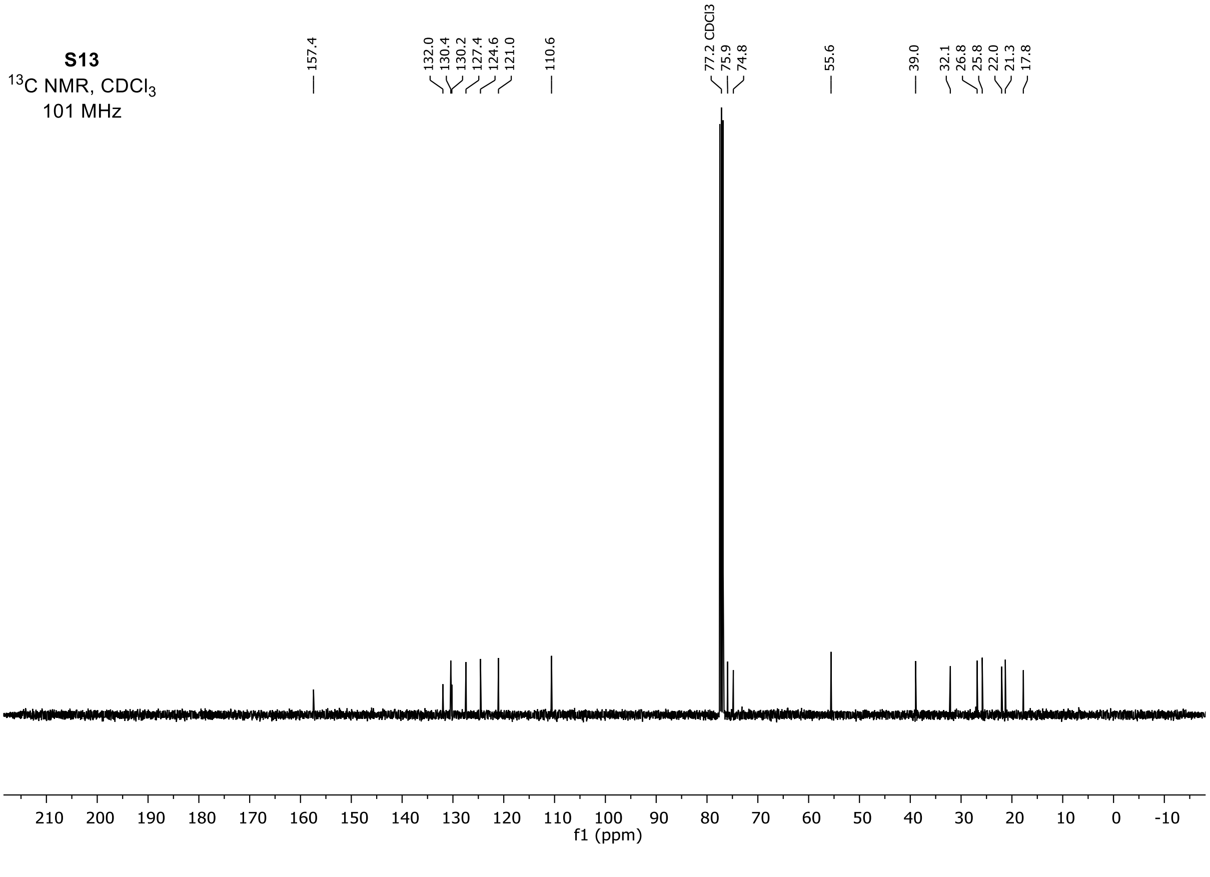
S13
13C NMR, CDCl3
101 MHz
157.4
132.0
130.4
130.2
127.4
124.6
121.0
110.6
77.2 CDCl3
75.9
74.8
55.6
39.0
32.1
26.8
25.8
22.0
21.3
17.8
f1 (ppm)

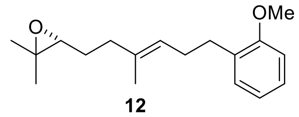

12

^{1}H NMR, $CDCl_3$

400 MHz

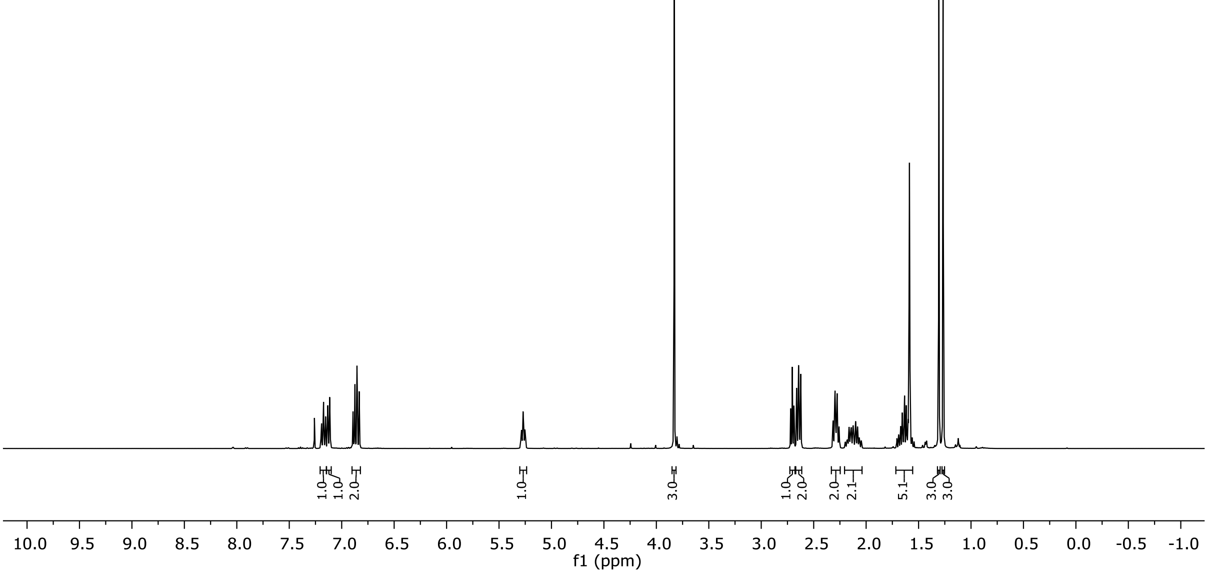

12

^{13}C NMR, $CDCl_3$

101 MHz

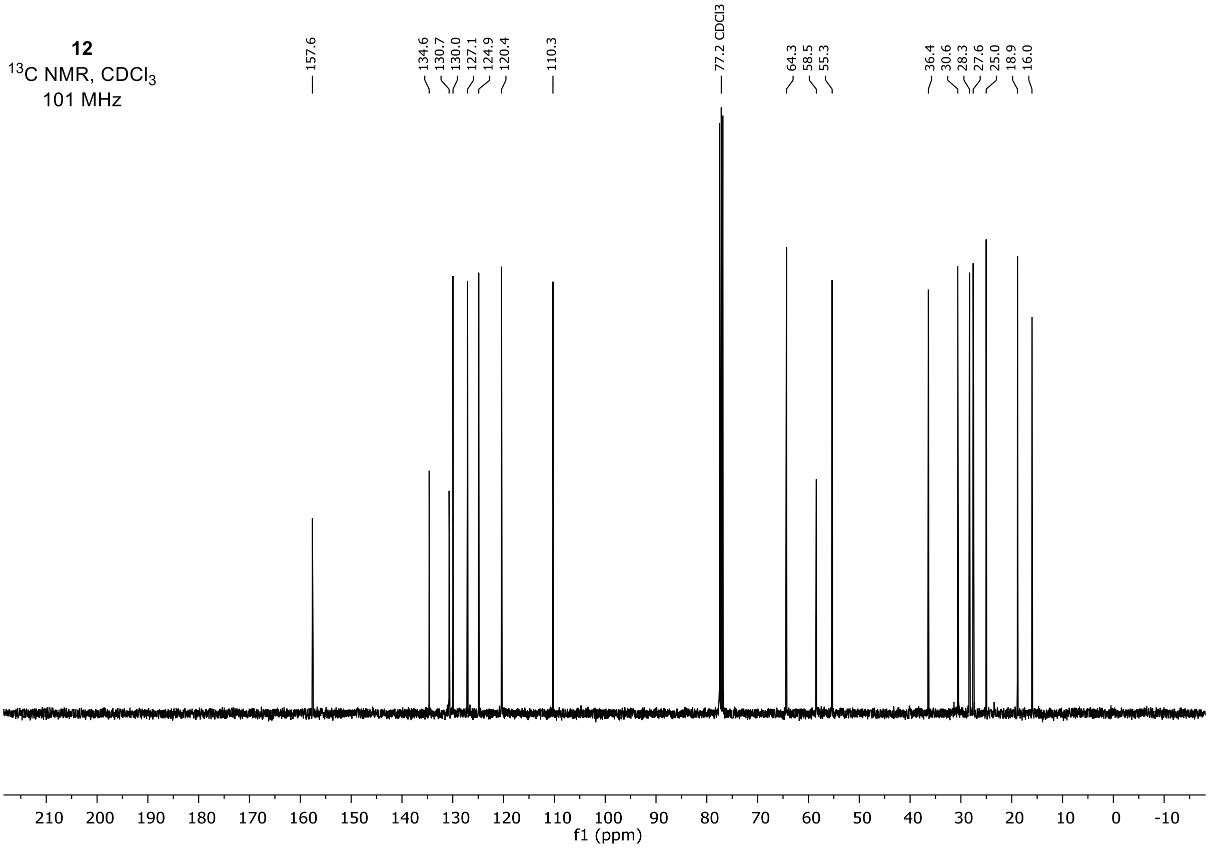

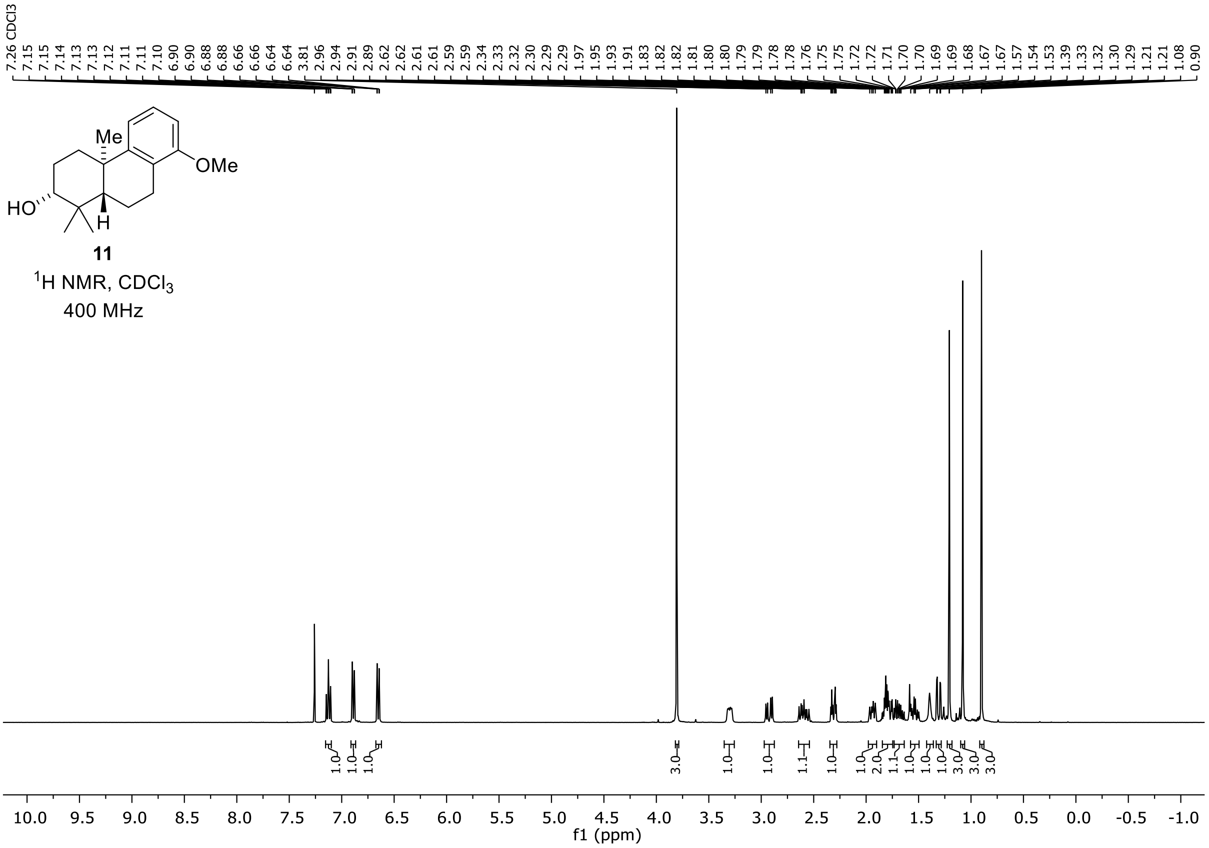
7.26 CDCl3
7.15
7.14
7.13
7.13
7.12
7.11
7.11
7.10
6.90
6.90
6.88
6.88
6.66
6.66
6.64
6.64
3.81
2.96
2.94
2.91
2.89
2.62
2.62
2.61
2.61
2.59
2.59
2.34
2.33
2.32
2.30
2.29
2.29
1.97
1.95
1.93
1.91
1.83
1.82
1.82
1.81
1.80
1.80
1.79
1.79
1.78
1.78
1.76
1.75
1.75
1.72
1.72
1.71
1.70
1.70
1.69
1.69
1.68
1.67
1.67
1.57
1.54
1.53
1.39
1.33
1.32
1.30
1.29
1.21
1.08
0.90
Me
OMe
HO
H
11
^{1}H NMR, CDCl$_3$
400 MHz
f1 (ppm)

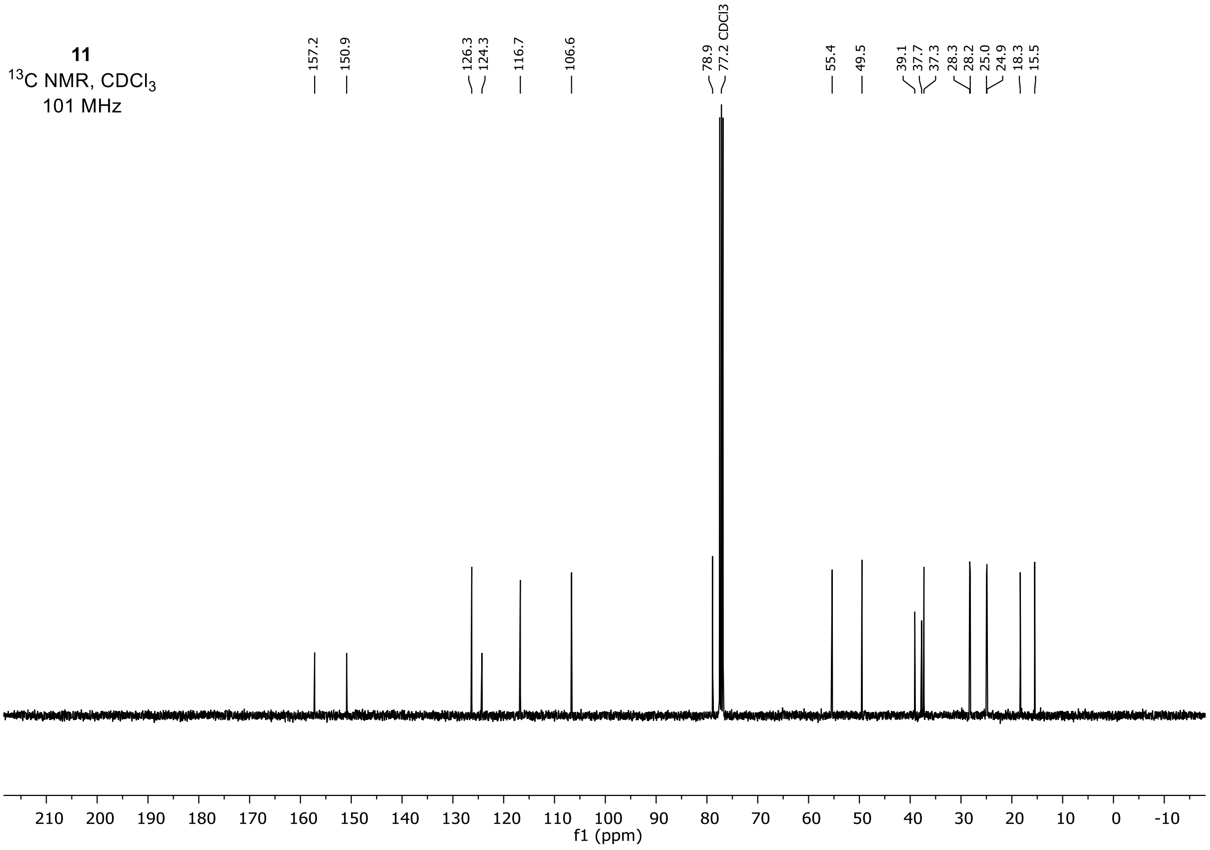
11
^{13}C NMR, CDCl$_3$
101 MHz
157.2
150.9
126.3
124.3
116.7
106.6
78.9
77.2 CDCl3
55.4
49.5
39.1
37.7
37.3
28.3
28.2
25.0
24.9
18.3
15.5
f1 (ppm)

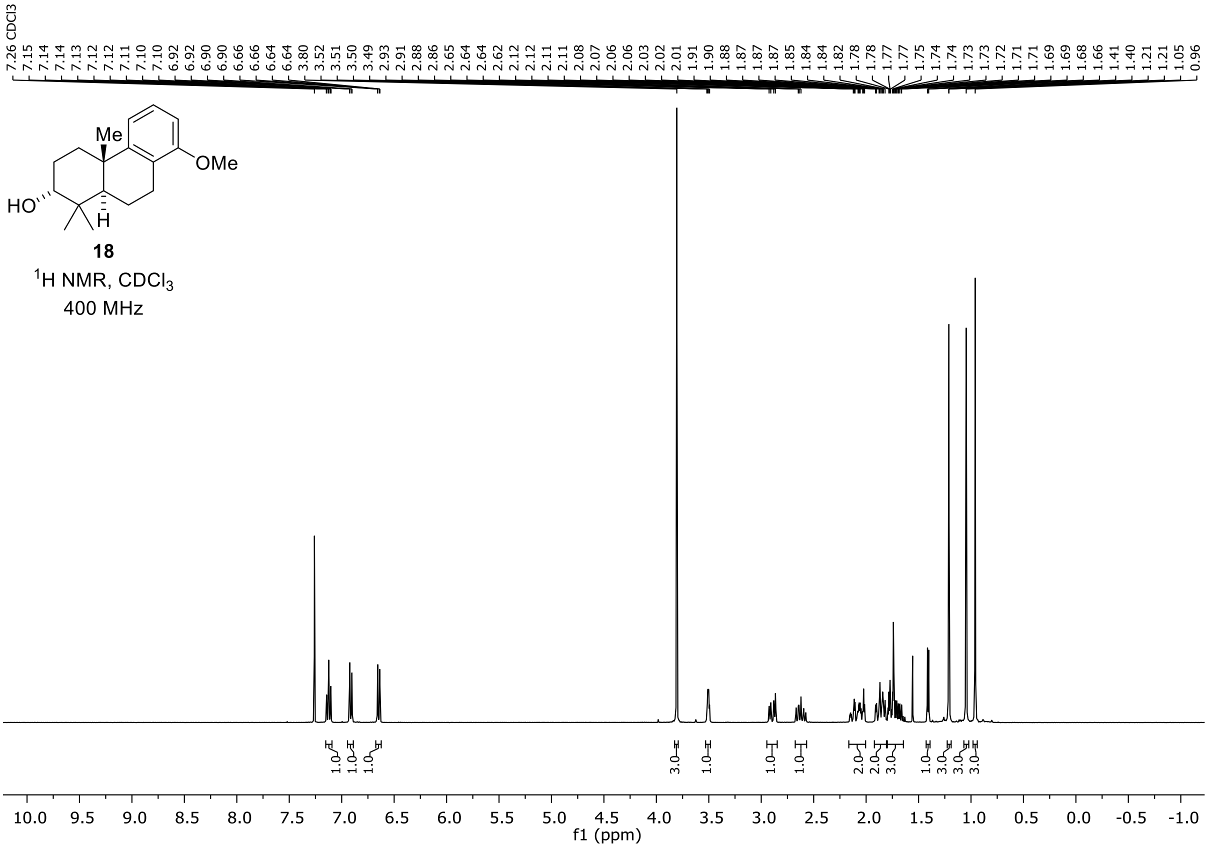

Me
OMe
HO
H
18
1H NMR, CDCl3
400 MHz
f1 (ppm)

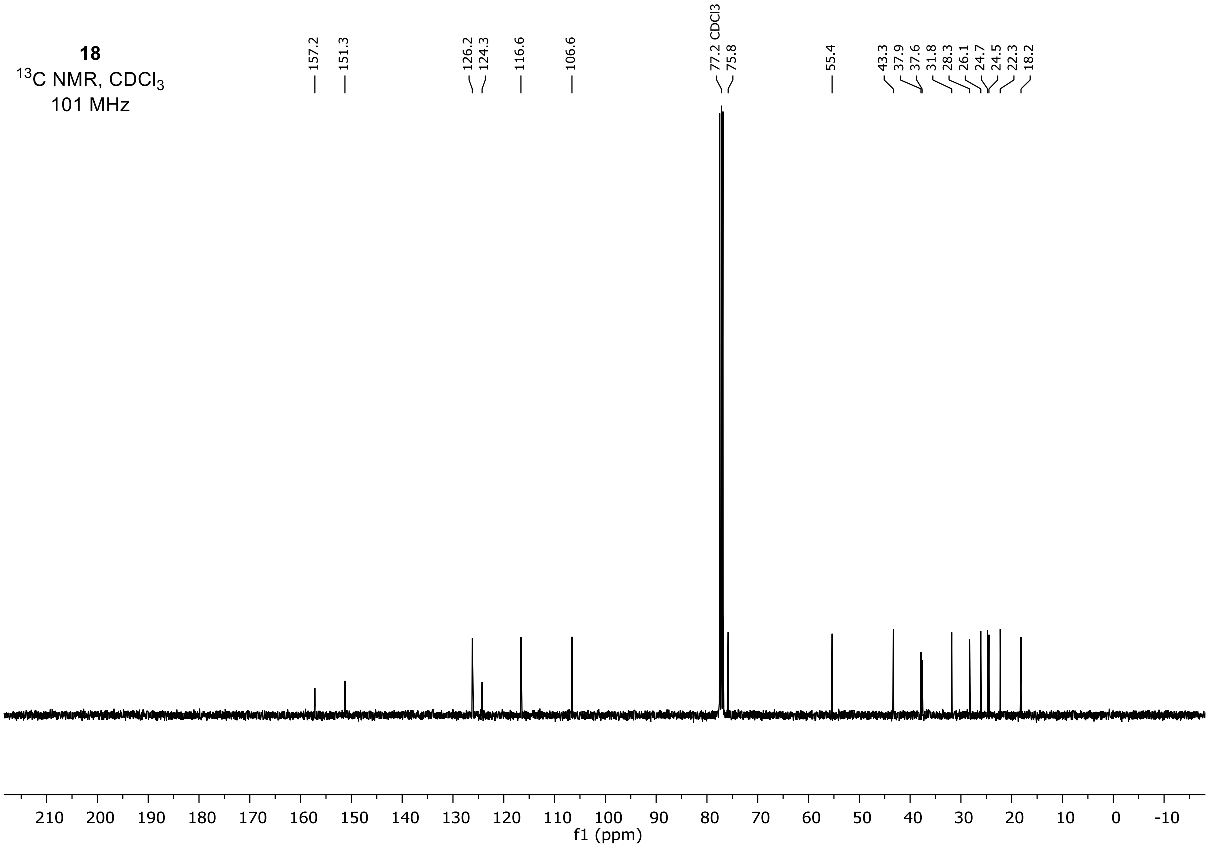

18
13C NMR, CDCl3
101 MHz
157.2
151.3
126.2
124.3
116.6
106.6
77.2 CDCl3
75.8
55.4
43.3
37.9
37.6
31.8
28.3
26.1
24.7
24.5
22.3
18.2
f1 (ppm)

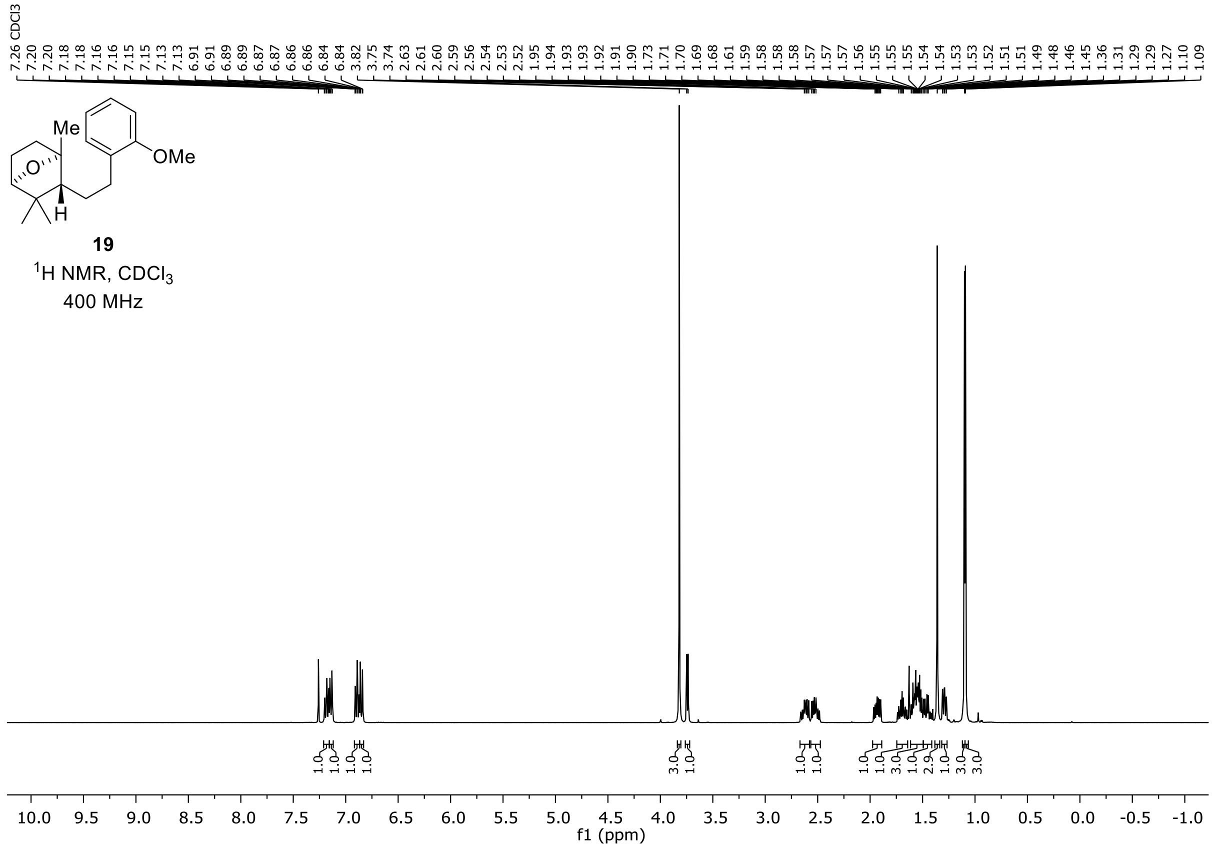
Me
O
OMe
H
19
1H NMR, CDCl3
400 MHz
f1 (ppm)

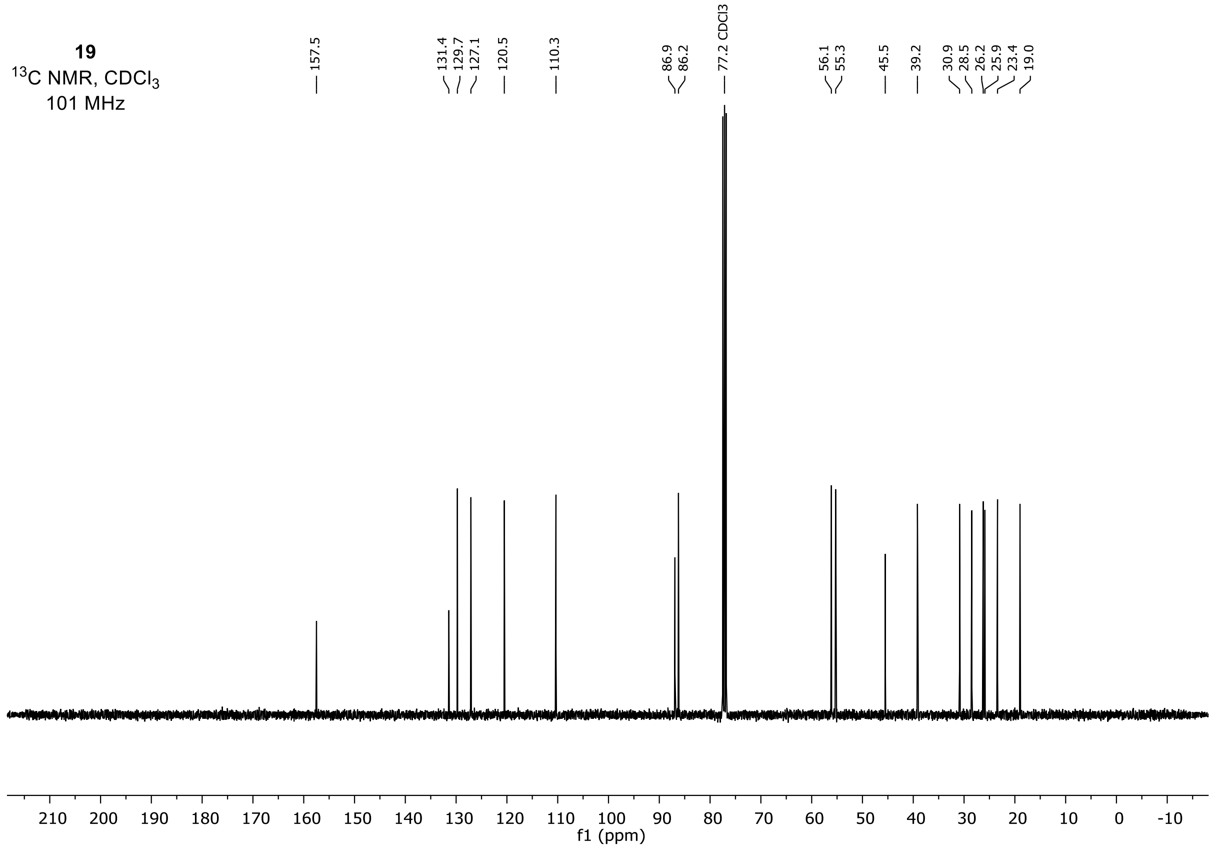
19
13C NMR, CDCl3
101 MHz
157.5
131.4
129.7
127.1
120.5
110.3
86.9
86.2
77.2 CDCl3
56.1
55.3
45.5
39.2
30.9
28.5
26.2
25.9
23.4
19.0
f1 (ppm)

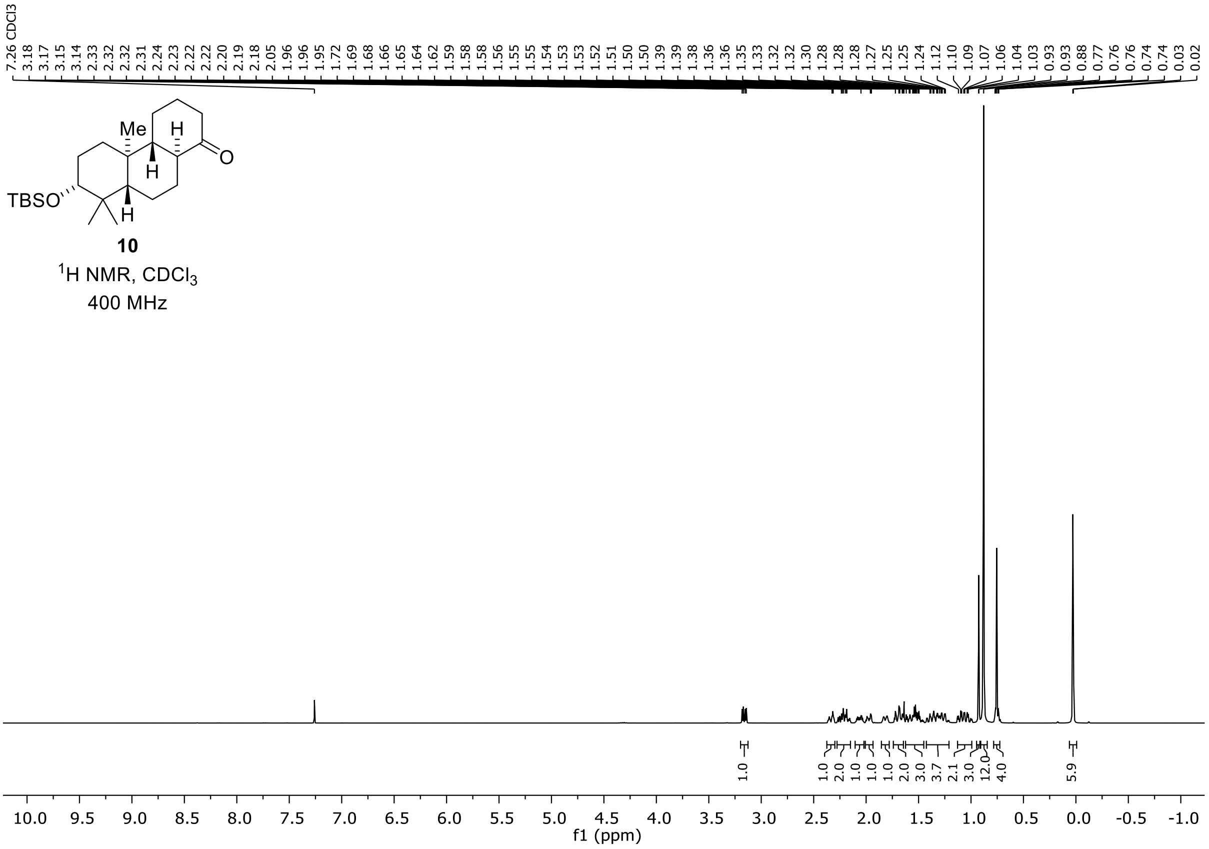
7.26 CDCl3
3.18
3.17
3.15
3.14
2.33
2.32
2.32
2.31
2.24
2.23
2.22
2.22
2.20
2.19
2.18
2.05
1.96
1.96
1.95
1.72
1.69
1.68
1.66
1.65
1.64
1.62
1.59
1.58
1.58
1.56
1.55
1.55
1.54
1.53
1.53
1.52
1.51
1.50
1.50
1.39
1.39
1.38
1.36
1.36
1.35
1.33
1.32
1.32
1.30
1.28
1.28
1.28
1.27
1.25
1.25
1.24
1.12
1.10
1.09
1.07
1.06
1.04
1.03
0.93
0.88
0.77
0.76
0.76
0.74
0.03
0.02
Me
H
H
O
TBSO
H
10
^{1}H NMR, CDCl$_3$
400 MHz
1.0
1.0
2.0
1.0
1.0
1.0
2.0
3.0
3.7
2.1
3.0
12.0
4.0
5.9
10.0 9.5 9.0 8.5 8.0 7.5 7.0 6.5 6.0 5.5 5.0 4.5 4.0 3.5 3.0 2.5 2.0 1.5 1.0 0.5 0.0 -0.5 -1.0
f1 (ppm)

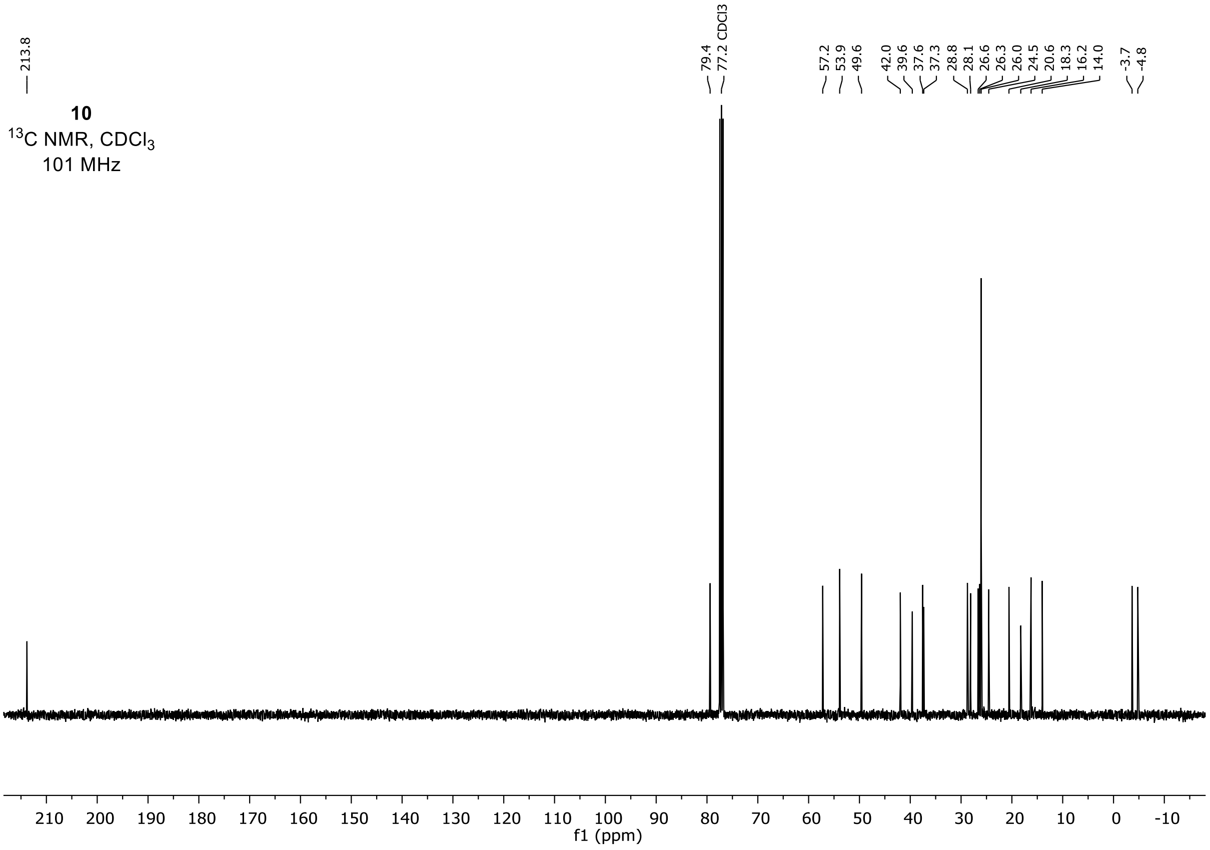
213.8
79.4
77.2 CDCl3
57.2
53.9
49.6
42.0
39.6
37.6
37.3
28.8
28.1
26.6
26.3
26.0
24.5
20.6
18.3
16.2
14.0
-3.7
-4.8
10
^{13}C NMR, CDCl$_3$
101 MHz
210 200 190 180 170 160 150 140 130 120 110 100 90 80 70 60 50 40 30 20 10 0 -10
f1 (ppm)

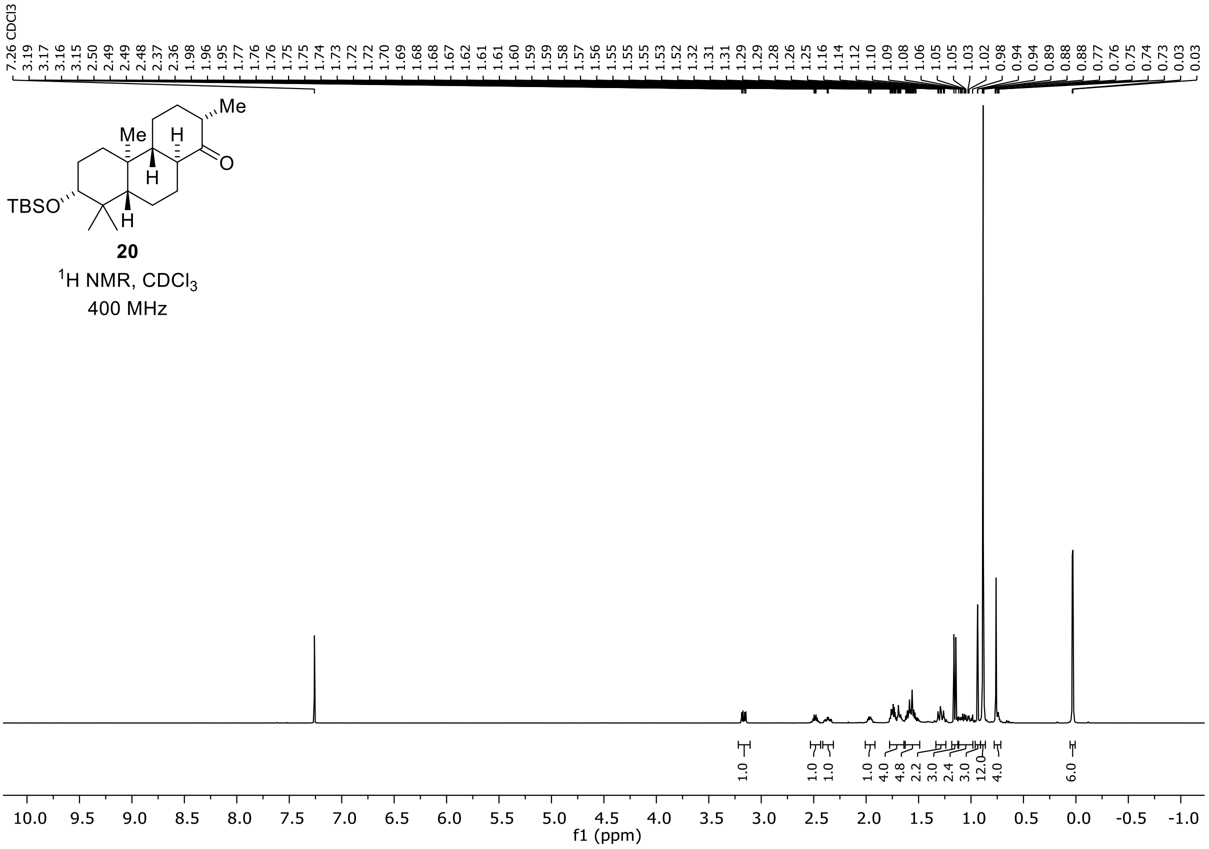
7.26 CDCl3
3.19 3.17 3.16 3.15 2.50 2.49 2.49 2.48 2.37 2.36 1.98 1.96 1.95 1.77 1.76 1.76 1.75 1.75 1.74 1.73 1.72 1.72 1.70 1.69 1.68 1.68 1.67 1.62 1.61 1.61 1.60 1.59 1.59 1.58 1.57 1.56 1.55 1.55 1.55 1.53 1.52 1.32 1.31 1.31 1.29 1.29 1.28 1.26 1.25 1.16 1.14 1.12 1.10 1.09 1.08 1.06 1.05 1.05 1.03 1.02 0.98 0.94 0.94 0.89 0.88 0.88 0.77 0.76 0.75 0.74 0.73 0.03 0.03
Me
Me
H
H
O
TBSO
H
20
1H NMR, CDCl3
400 MHz
1.0 1.0 1.0 1.0 4.0 4.8 2.2 3.0 2.4 3.0 12.0 4.0 6.0
10.0 9.5 9.0 8.5 8.0 7.5 7.0 6.5 6.0 5.5 5.0 4.5 4.0 3.5 3.0 2.5 2.0 1.5 1.0 0.5 0.0 -0.5 -1.0
f1 (ppm)

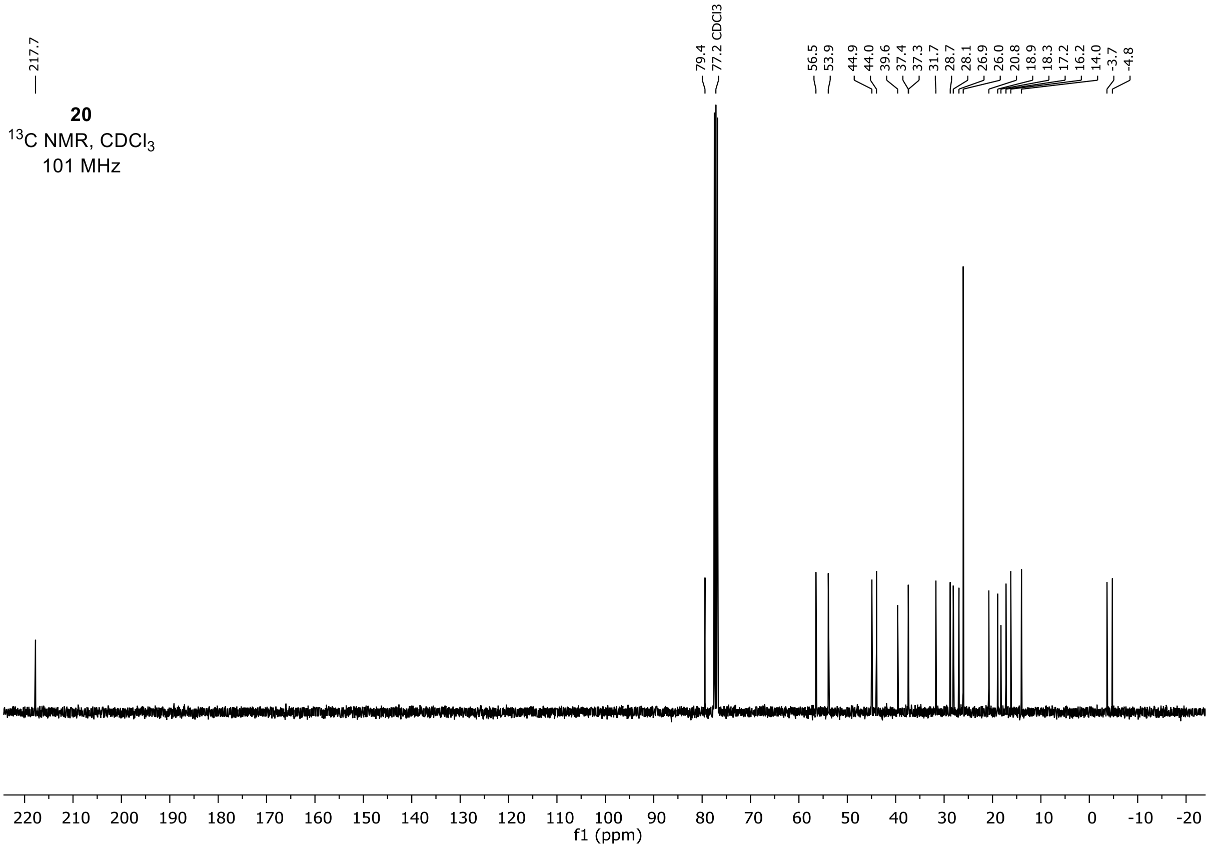
217.7
79.4
77.2 CDCl3
56.5 53.9 44.9 44.0 39.6 37.4 37.3 31.7 28.7 28.1 26.9 26.0 20.8 18.9 18.3 17.2 16.2 14.0 -3.7 -4.8
20
13C NMR, CDCl3
101 MHz
220 210 200 190 180 170 160 150 140 130 120 110 100 90 80 70 60 50 40 30 20 10 0 -10 -20
f1 (ppm)

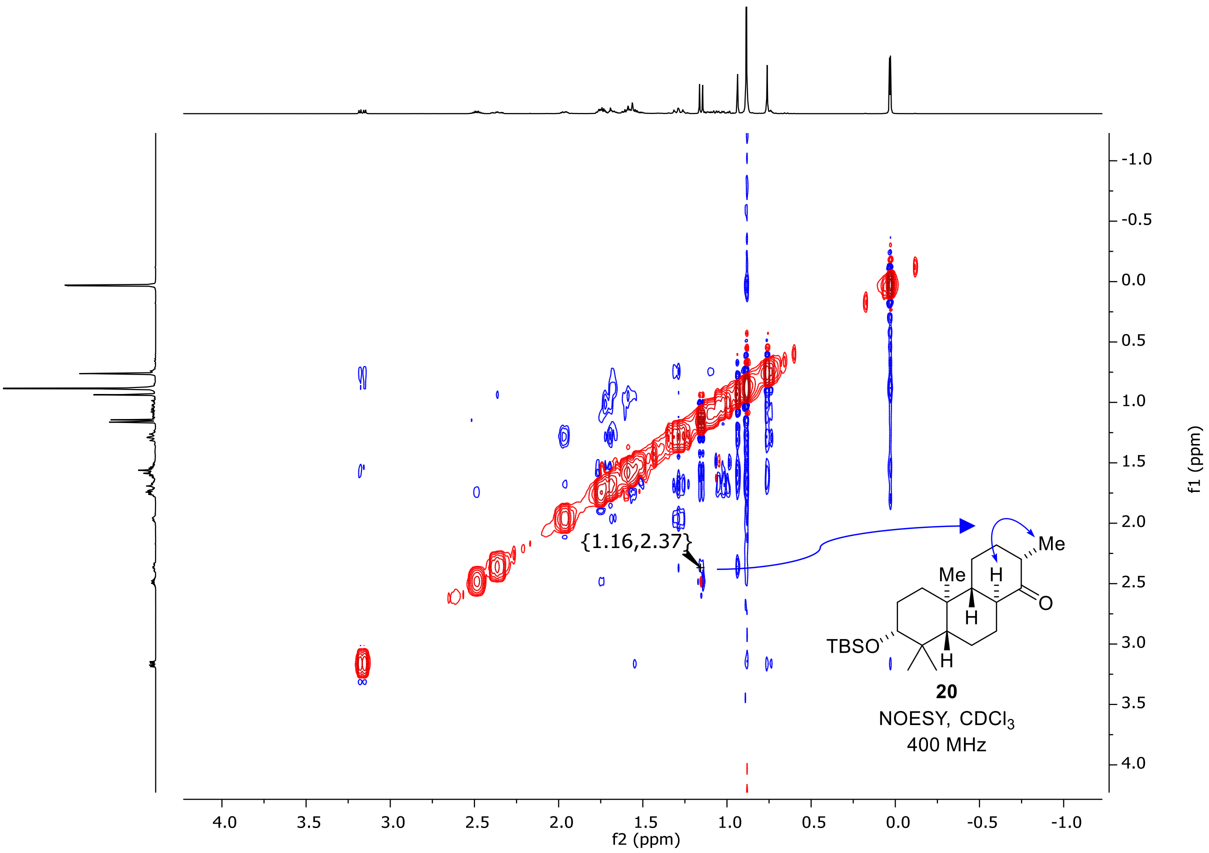

{1.16,2.37}
Me
Me
H
H
O
TBSO
H
20
NOESY, CDCl3
400 MHz
f1 (ppm)
f2 (ppm)

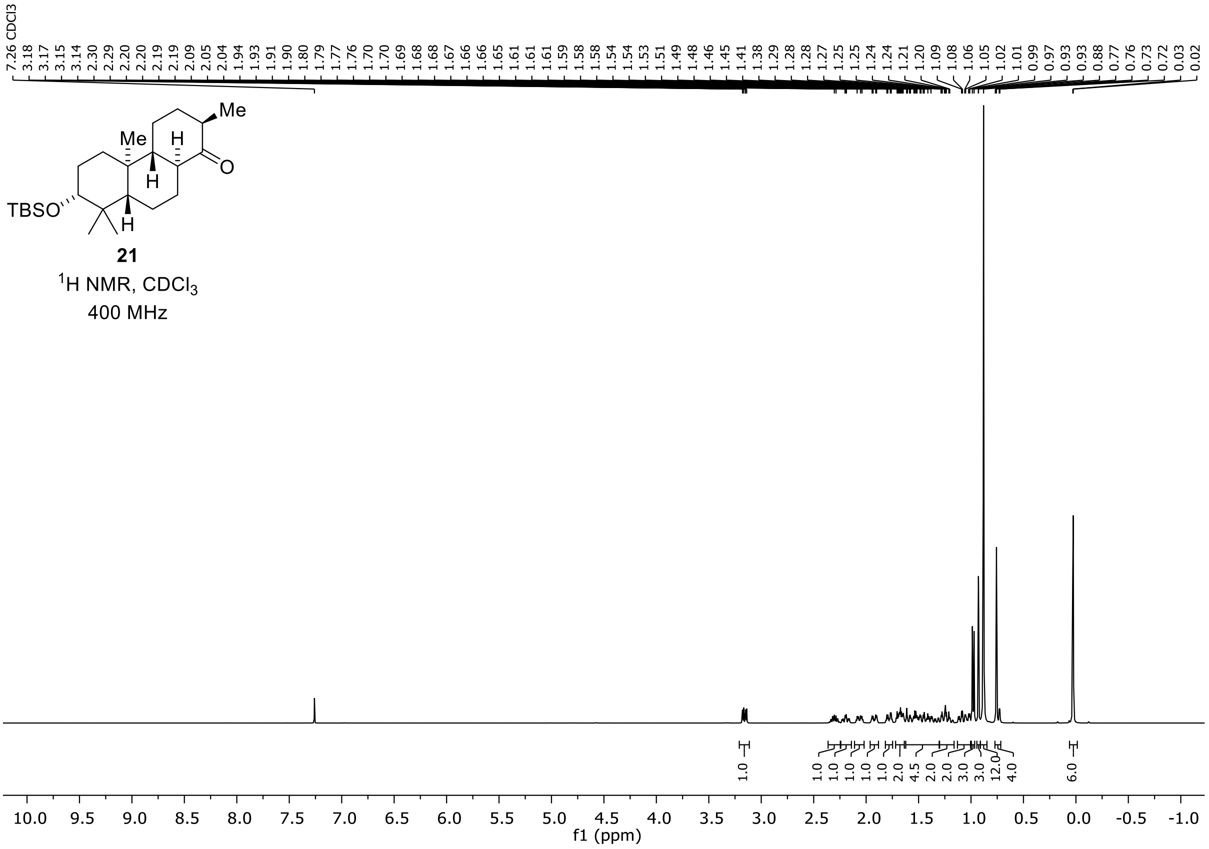
7.26 CDCl3
3.18 3.17 3.15 3.14 2.30 2.29 2.20 2.20 2.19 2.19 2.09 2.05 2.04 1.94 1.93 1.91 1.90 1.80 1.79 1.77 1.76 1.70 1.70 1.69 1.68 1.67 1.66 1.66 1.65 1.61 1.61 1.61 1.59 1.58 1.58 1.54 1.54 1.53 1.51 1.49 1.48 1.46 1.45 1.41 1.38 1.29 1.28 1.28 1.27 1.25 1.25 1.24 1.24 1.21 1.20 1.09 1.08 1.06 1.05 1.02 1.01 0.99 0.97 0.93 0.93 0.88 0.77 0.76 0.73 0.72 0.03 0.02
Me
Me
H
H
O
TBSO
H
21
^{1}H NMR, $CDCl_3$
400 MHz
1.0
1.0 1.0 1.0 1.0 2.0 4.5 2.0 2.0 3.0 3.0 12.0 4.0
6.0
10.0 9.5 9.0 8.5 8.0 7.5 7.0 6.5 6.0 5.5 5.0 4.5 4.0 3.5 3.0 2.5 2.0 1.5 1.0 0.5 0.0 -0.5 -1.0
f1 (ppm)

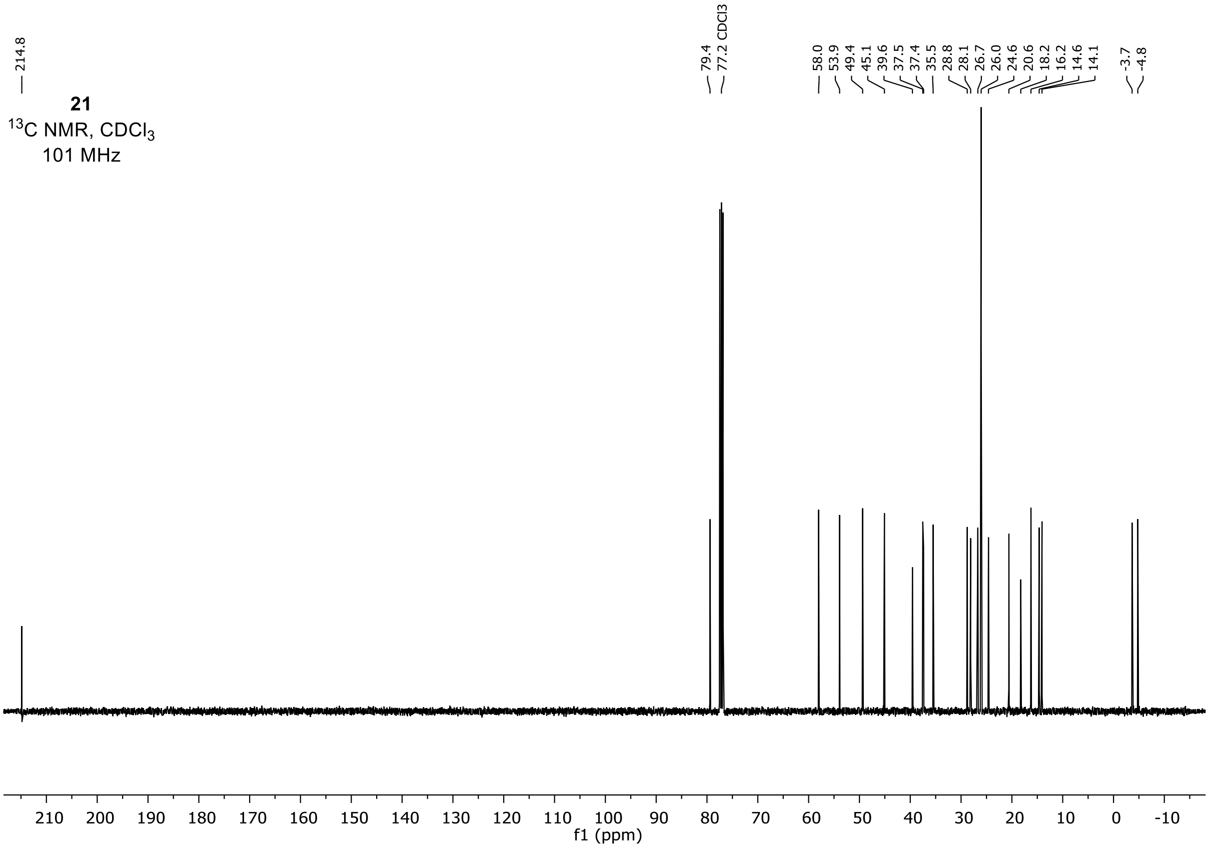
214.8
79.4
77.2 CDCl3
58.0 53.9 49.4 45.1 39.6 37.5 37.4 35.5 28.8 28.1 26.7 26.0 24.6 20.6 18.2 16.2 14.6 14.1
-3.7 -4.8
21
^{13}C NMR, $CDCl_3$
101 MHz
210 200 190 180 170 160 150 140 130 120 110 100 90 80 70 60 50 40 30 20 10 0 -10
f1 (ppm)

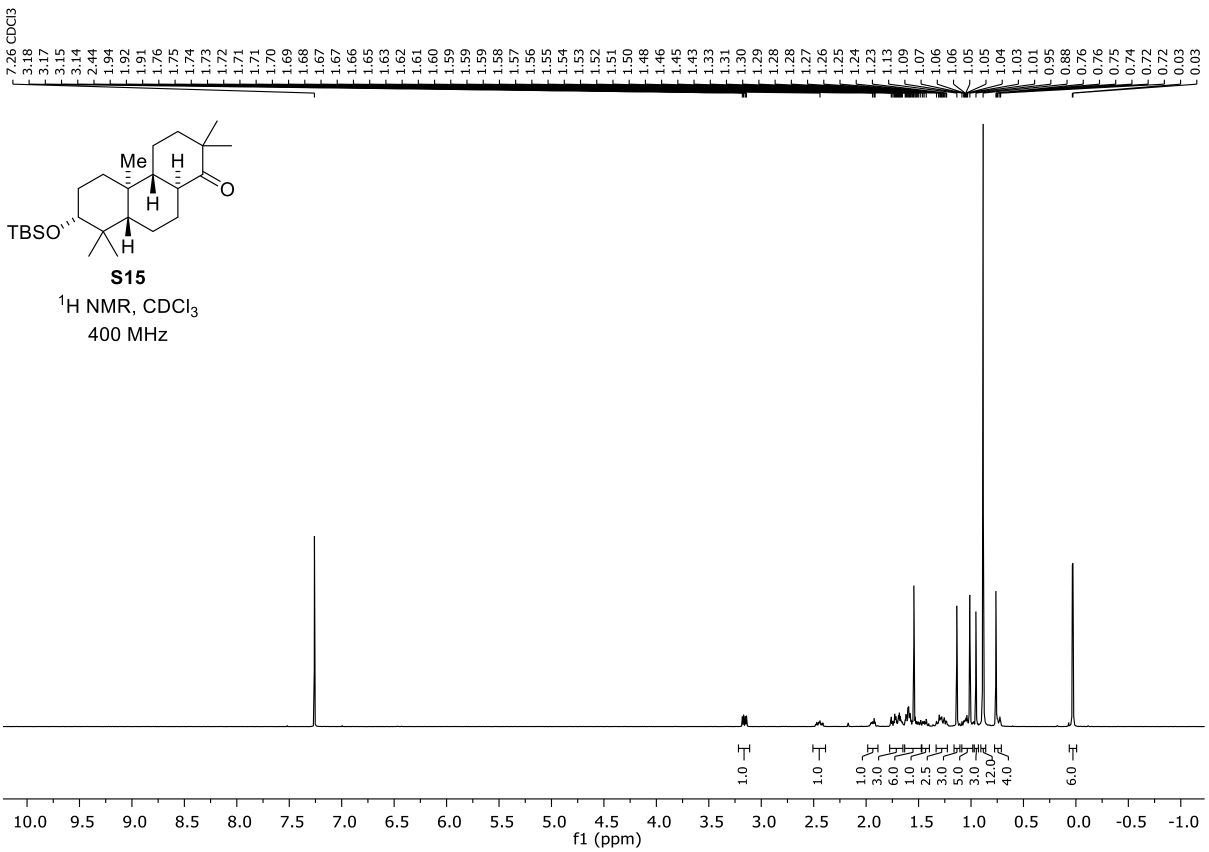
7.26 CDCl3
3.18
3.17
3.15
3.14
2.44
1.94
1.92
1.91
1.76
1.75
1.74
1.73
1.72
1.71
1.71
1.70
1.69
1.68
1.67
1.67
1.66
1.65
1.63
1.62
1.61
1.60
1.59
1.59
1.59
1.58
1.57
1.56
1.55
1.54
1.53
1.52
1.51
1.50
1.48
1.46
1.45
1.43
1.33
1.31
1.30
1.29
1.28
1.27
1.26
1.25
1.24
1.23
1.13
1.09
1.07
1.06
1.06
1.05
1.05
1.04
1.03
1.01
0.95
0.88
0.76
0.76
0.75
0.74
0.72
0.72
0.03
0.03
Me
H
H
O
TBSO
H
S15
^{1}H NMR, CDCl$_3$
400 MHz
1.0
1.0
1.0
3.0
6.0
1.0
2.5
3.0
5.0
3.0
12.0
4.0
6.0
10.0 9.5 9.0 8.5 8.0 7.5 7.0 6.5 6.0 5.5 5.0 4.5 4.0 3.5 3.0 2.5 2.0 1.5 1.0 0.5 0.0 -0.5 -1.0
f1 (ppm)

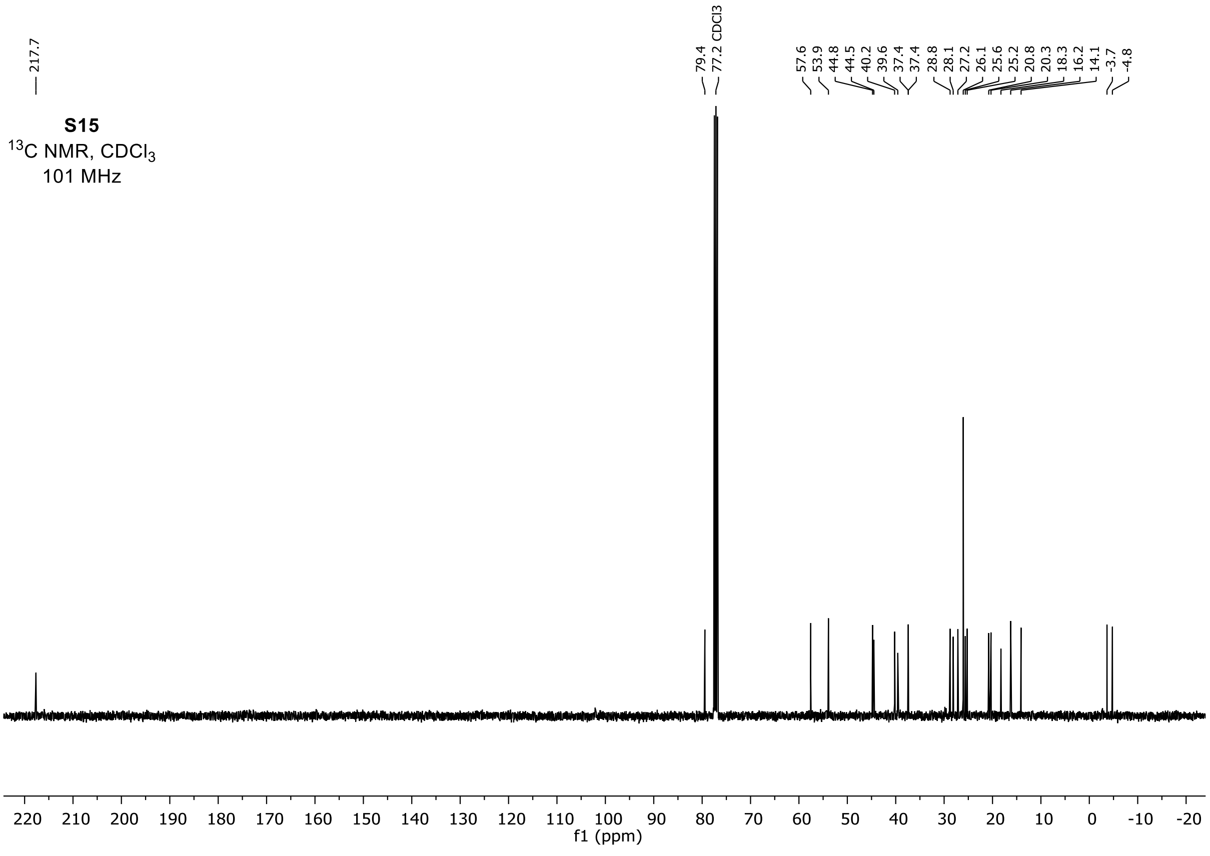
217.7
79.4
77.2 CDCl3
57.6
53.9
44.8
44.5
40.2
39.6
37.4
37.4
28.8
28.1
27.2
26.1
25.6
25.2
20.8
20.3
18.3
16.2
14.1
-3.7
-4.8
S15
^{13}C NMR, CDCl$_3$
101 MHz
220 210 200 190 180 170 160 150 140 130 120 110 100 90 80 70 60 50 40 30 20 10 0 -10 -20
f1 (ppm)

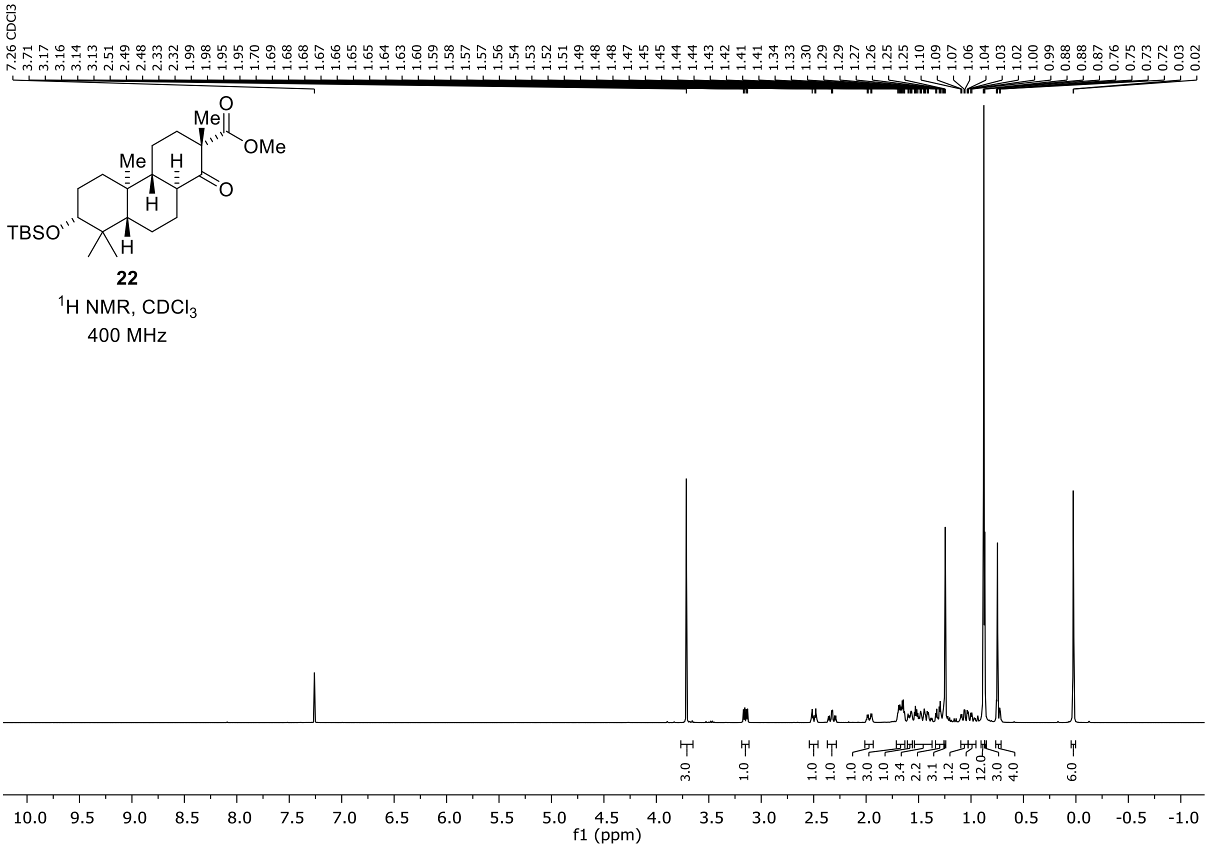
7.26 CDCl3
3.71 3.17 3.16 3.14 3.13 2.51 2.49 2.48 2.33 2.32 1.99 1.98 1.95 1.95 1.70 1.69 1.68 1.68 1.67 1.66 1.65 1.65 1.64 1.63 1.60 1.59 1.58 1.57 1.57 1.56 1.54 1.53 1.52 1.51 1.49 1.48 1.48 1.47 1.45 1.45 1.44 1.44 1.43 1.42 1.41 1.41 1.34 1.33 1.30 1.29 1.29 1.27 1.26 1.25 1.25 1.10 1.09 1.07 1.06 1.04 1.03 1.02 1.00 0.99 0.88 0.87 0.76 0.75 0.73 0.72 0.03 0.02
Me
O
OMe
Me
H
H
O
TBSO
H
22
1H NMR, CDCl3
400 MHz
3.0 1.0 1.0 1.0 1.0 3.0 1.0 3.4 2.2 3.1 1.2 1.0 1.0 12.0 3.0 4.0 6.0
10.0 9.5 9.0 8.5 8.0 7.5 7.0 6.5 6.0 5.5 5.0 4.5 4.0 3.5 3.0 2.5 2.0 1.5 1.0 0.5 0.0 -0.5 -1.0
f1 (ppm)

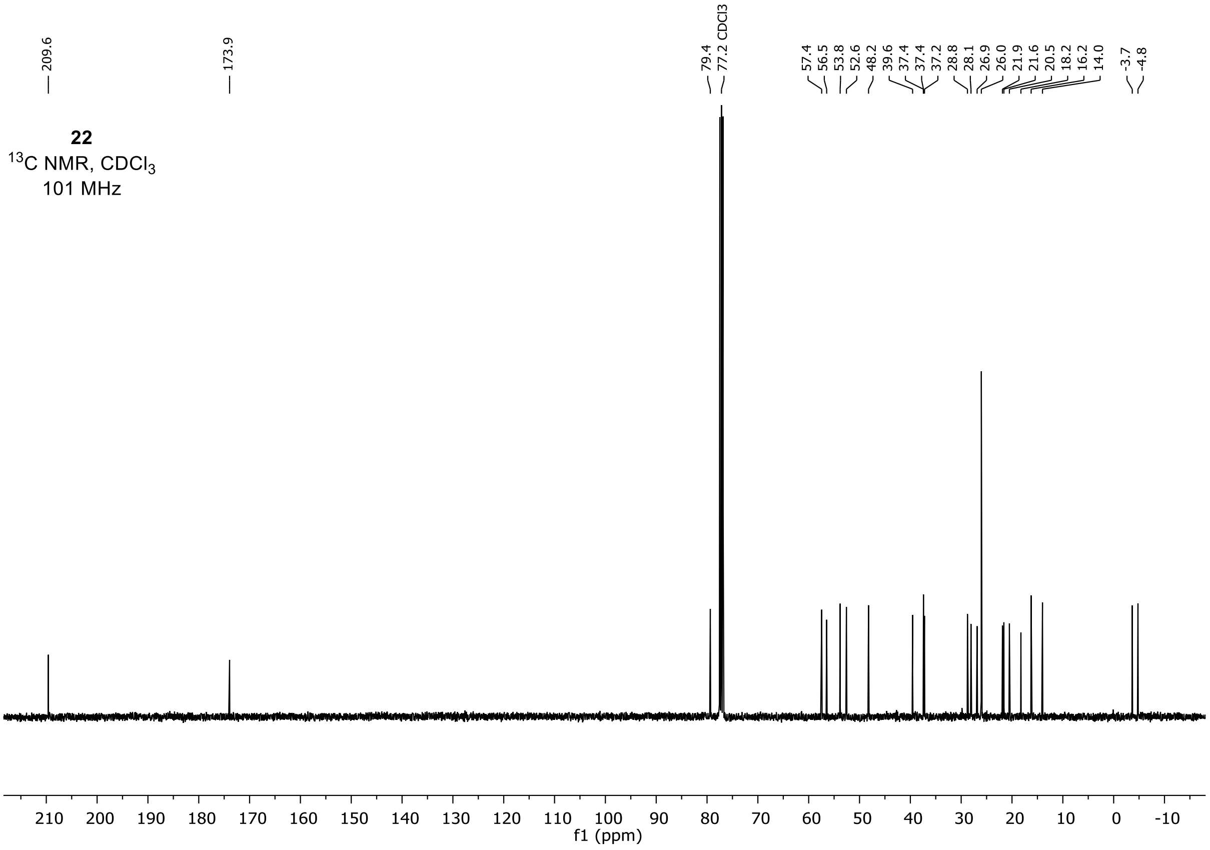
209.6
173.9
79.4
77.2 CDCl3
57.4 56.5 53.8 52.6 48.2 39.6 37.4 37.4 37.2 28.8 28.1 26.9 26.0 21.9 21.6 20.5 18.2 16.2 14.0 -3.7 -4.8
22
13C NMR, CDCl3
101 MHz
210 200 190 180 170 160 150 140 130 120 110 100 90 80 70 60 50 40 30 20 10 0 -10
f1 (ppm)

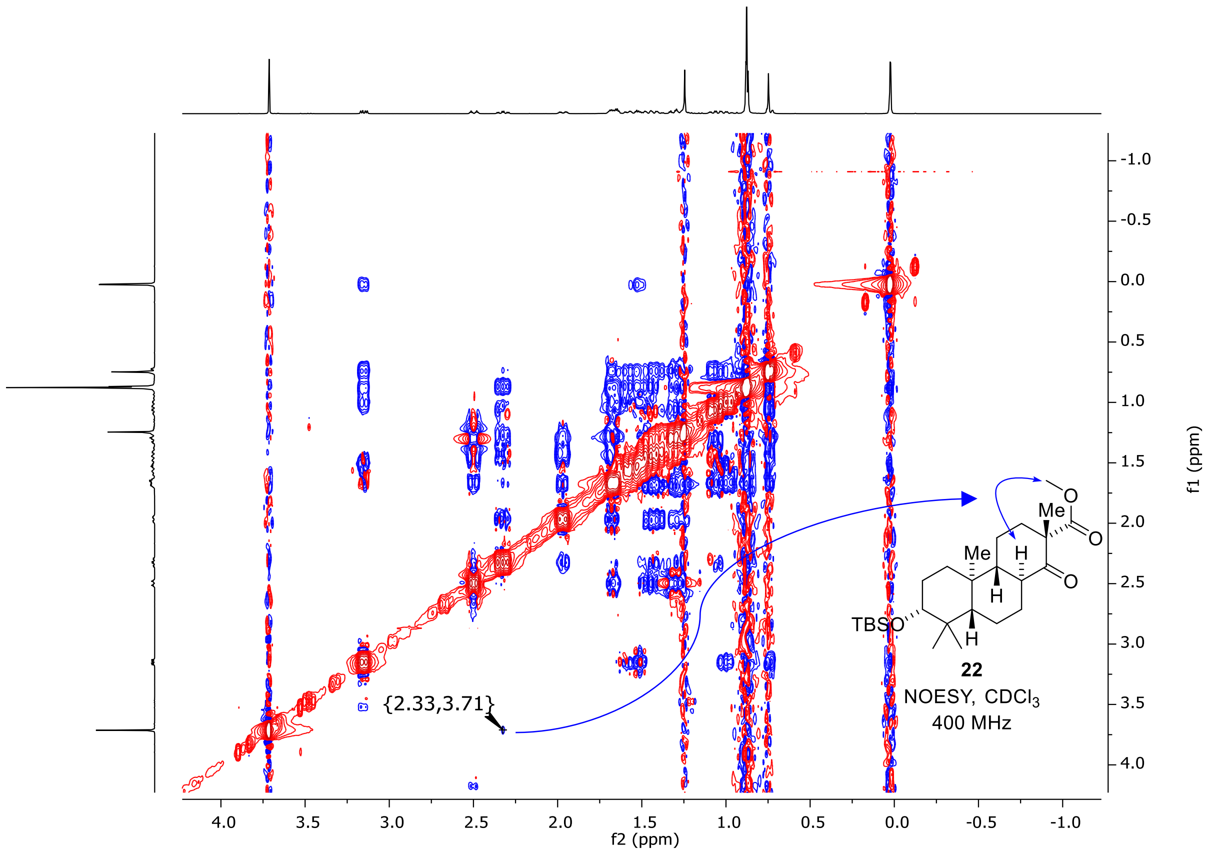
{2.33,3.71}
Me
O
O
Me
H
O
H
TBSO
H
22
NOESY, CDCl3
400 MHz
f2 (ppm)
f1 (ppm)

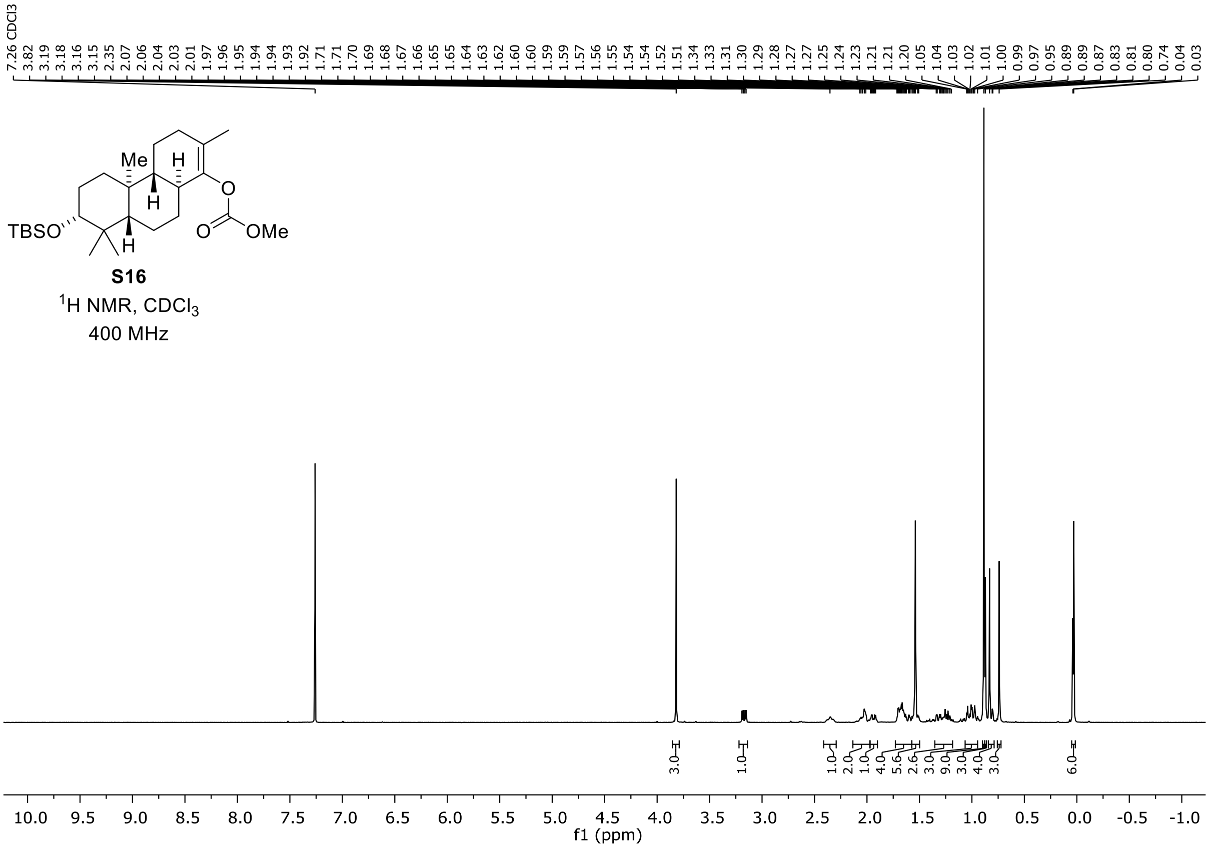
7.26 CDCl3
3.82
3.19
3.18
3.16
3.15
2.35
2.07
2.06
2.04
2.03
2.01
1.97
1.96
1.95
1.94
1.94
1.93
1.92
1.71
1.71
1.70
1.69
1.68
1.67
1.66
1.65
1.64
1.63
1.62
1.60
1.59
1.59
1.57
1.56
1.55
1.54
1.54
1.52
1.51
1.51
1.34
1.33
1.31
1.30
1.29
1.28
1.27
1.27
1.25
1.24
1.23
1.21
1.21
1.20
1.05
1.04
1.03
1.02
1.01
1.00
0.99
0.97
0.95
0.89
0.87
0.83
0.81
0.80
0.74
0.04
0.03
Me
H
H
H
O
O
OMe
TBSO
S16
^{1}H NMR, $CDCl_3$
400 MHz
3.0
1.0
1.0
2.0
1.0
4.0
5.6
2.6
3.0
9.0
3.0
4.0
3.0
6.0
f1 (ppm)

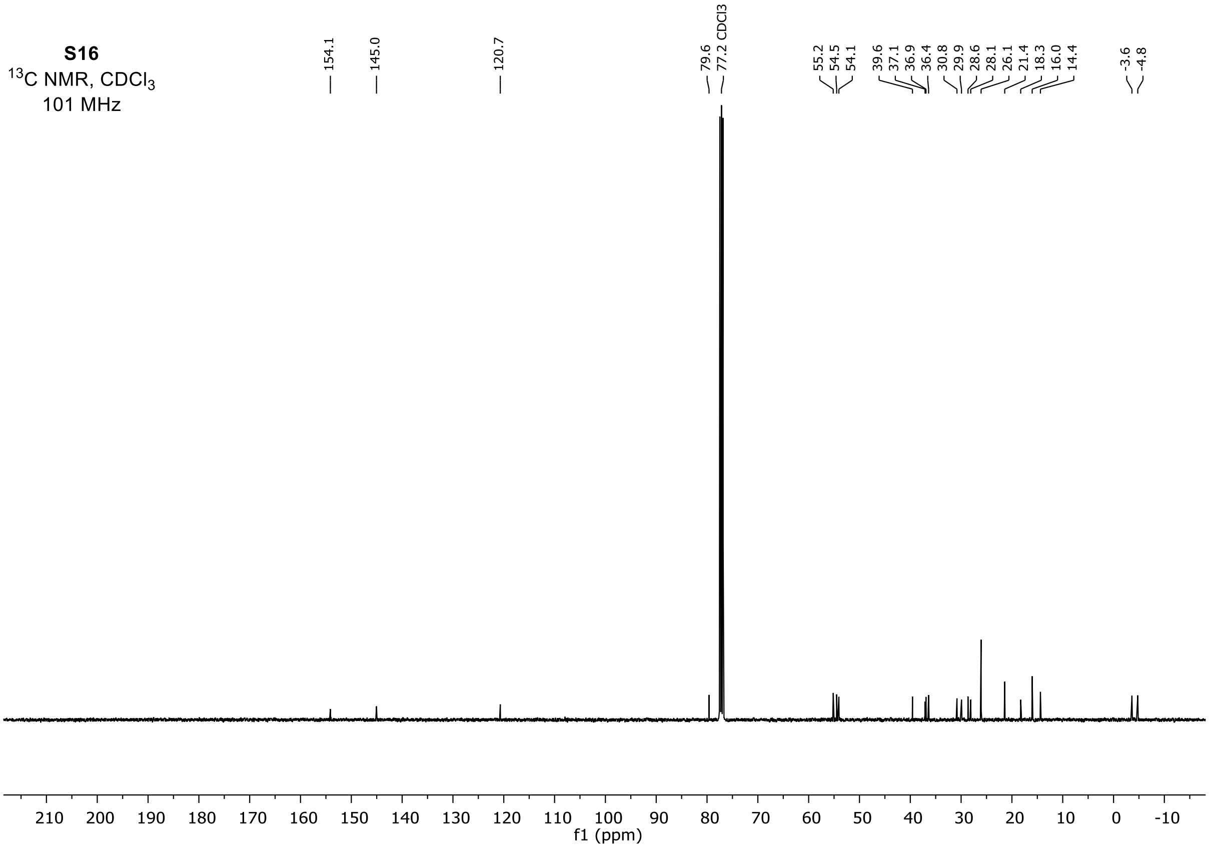
S16
^{13}C NMR, $CDCl_3$
101 MHz
154.1
145.0
120.7
79.6
77.2 CDCl3
55.2
54.5
54.1
39.6
37.1
36.9
36.4
30.8
29.9
28.6
28.1
26.1
21.4
18.3
16.0
14.4
-3.6
-4.8
f1 (ppm)

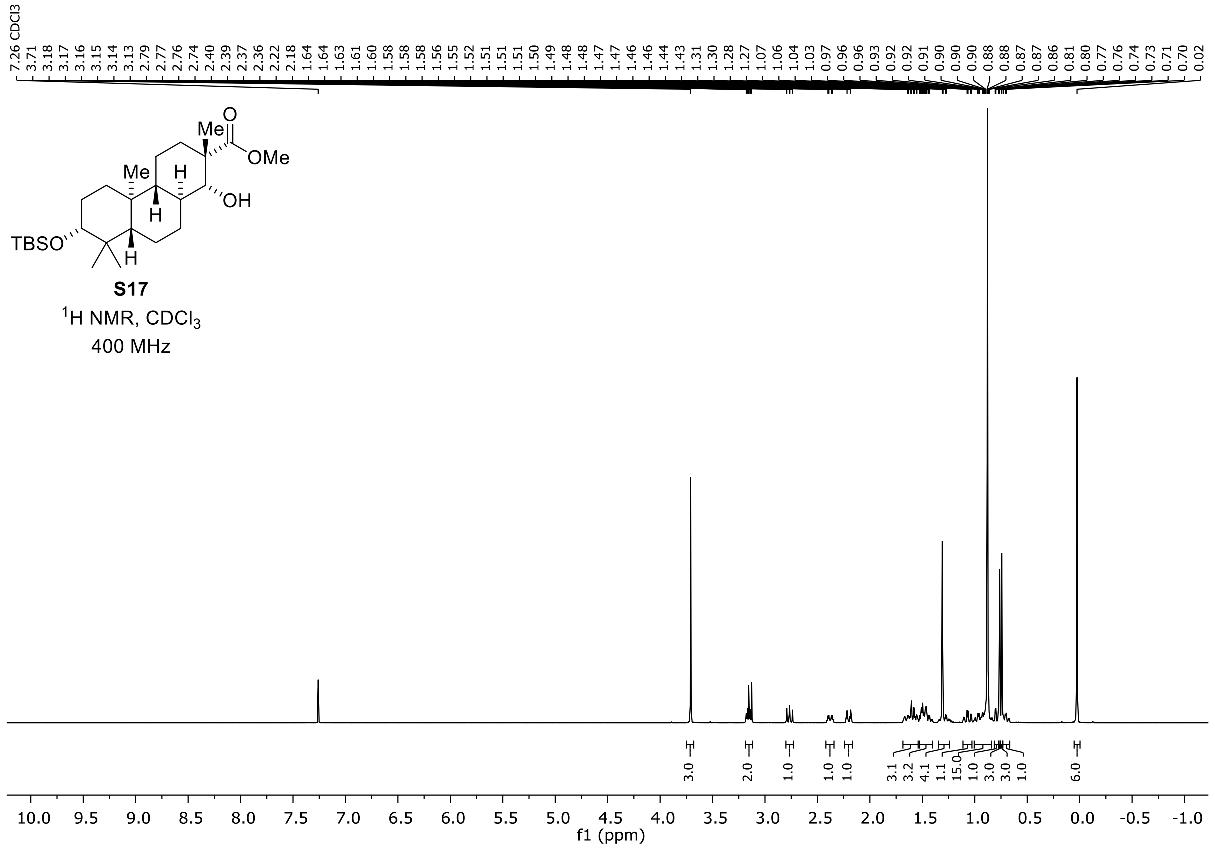

S17
1H NMR, CDCl3
400 MHz
f1 (ppm)

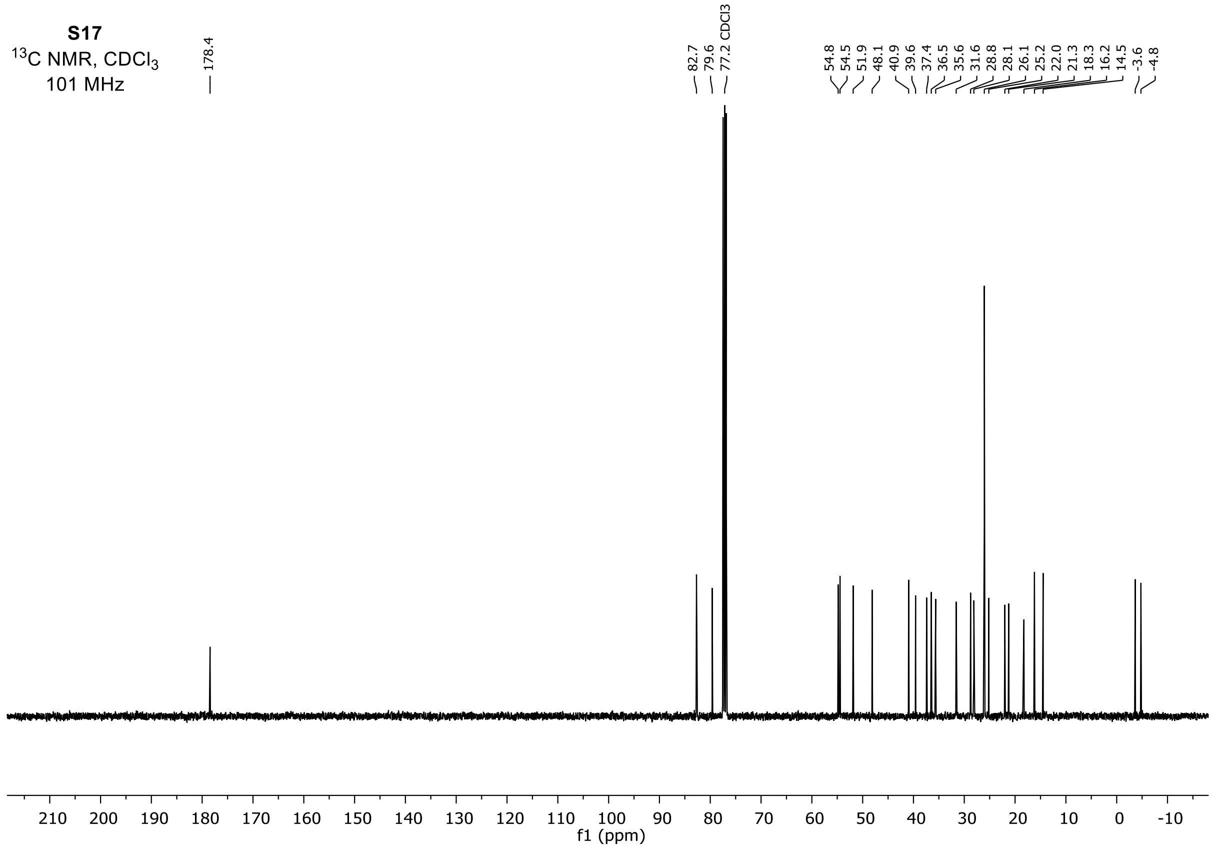

S17
13C NMR, CDCl3
101 MHz
f1 (ppm)

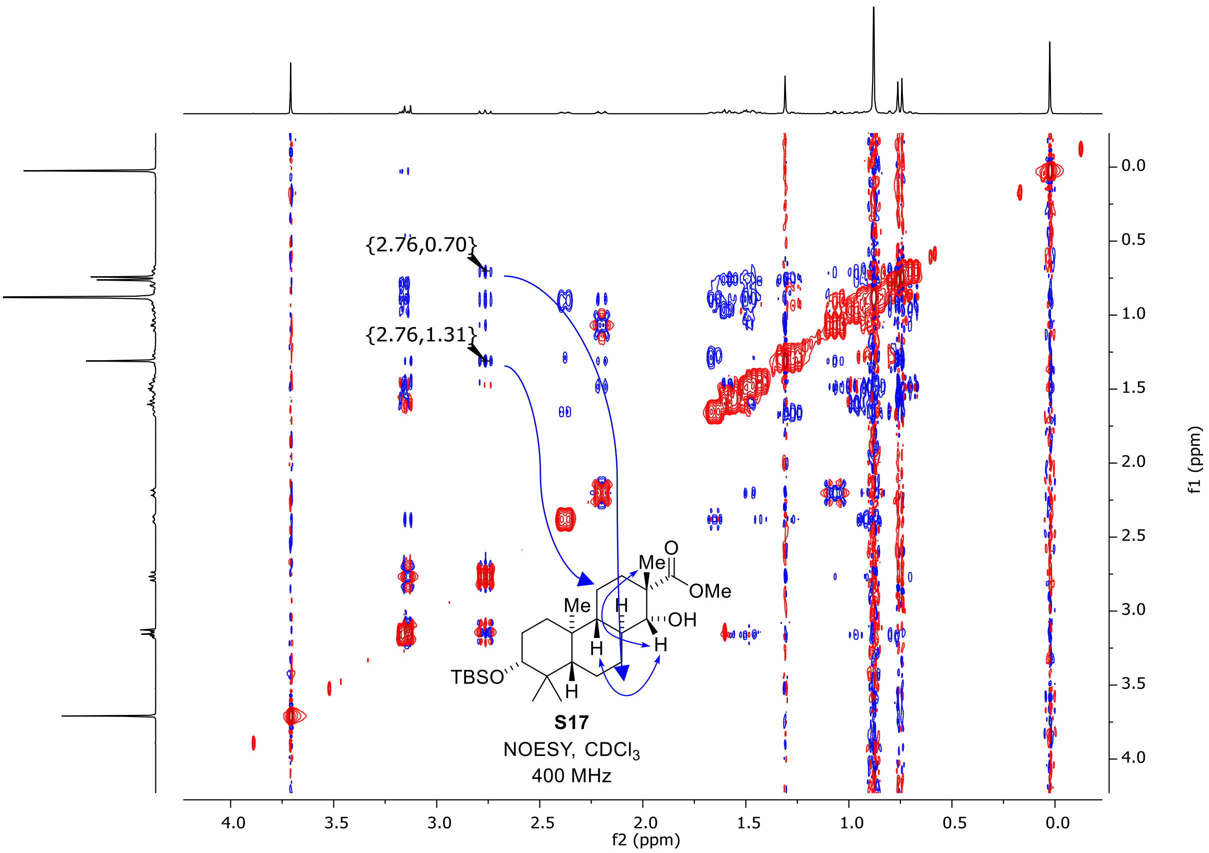

{2.76,0.70}
{2.76,1.31}
Me
O
OMe
Me
H
OH
H
H
TBSO
H
S17
NOESY, CDCl3
400 MHz
f2 (ppm)
f1 (ppm)

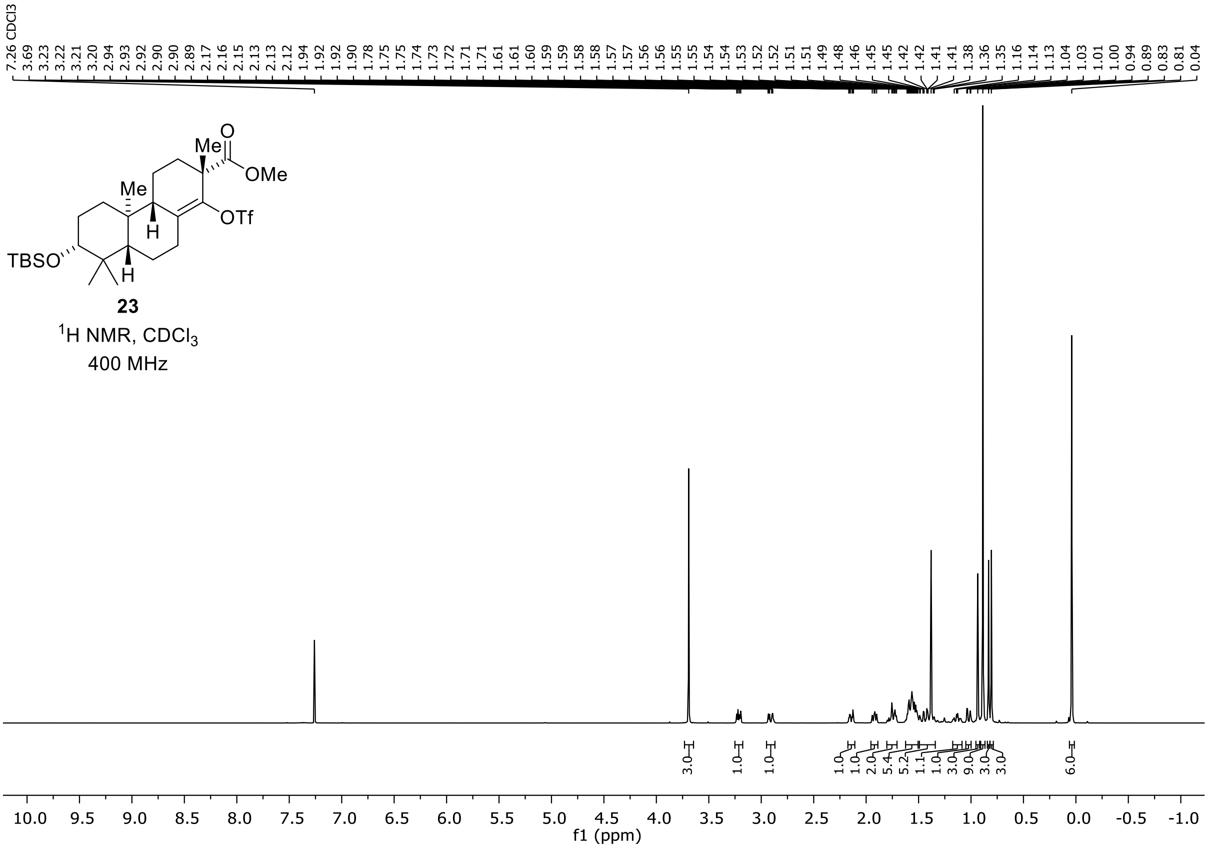
7.26 CDCl3
3.69
3.23
3.22
3.21
3.20
2.94
2.93
2.92
2.90
2.90
2.89
2.17
2.16
2.15
2.13
2.13
2.12
1.94
1.92
1.92
1.90
1.78
1.75
1.75
1.74
1.73
1.72
1.71
1.71
1.61
1.61
1.60
1.59
1.59
1.58
1.58
1.57
1.57
1.56
1.56
1.55
1.55
1.54
1.54
1.53
1.52
1.52
1.51
1.51
1.49
1.48
1.46
1.45
1.45
1.42
1.42
1.41
1.41
1.38
1.36
1.35
1.16
1.14
1.13
1.04
1.03
1.01
1.00
0.94
0.89
0.83
0.81
0.04
Me
O
OMe
Me
OTf
H
TBSO
H
23
¹H NMR, CDCl3
400 MHz
3.0
1.0
1.0
1.0
1.0
2.0
5.4
5.2
1.1
1.0
3.0
9.0
3.0
3.0
6.0
10.0 9.5 9.0 8.5 8.0 7.5 7.0 6.5 6.0 5.5 5.0 4.5 4.0 3.5 3.0 2.5 2.0 1.5 1.0 0.5 0.0 -0.5 -1.0
f1 (ppm)

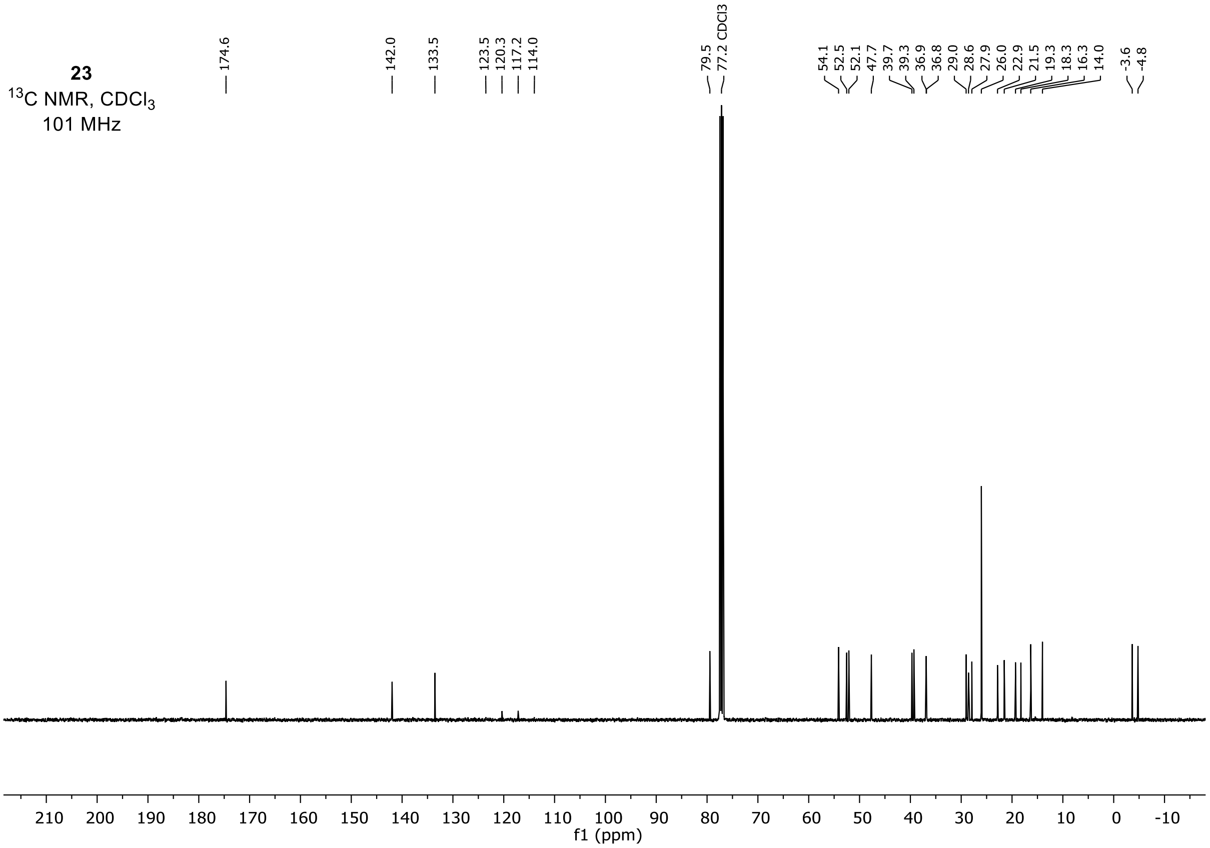
23
¹³C NMR, CDCl3
101 MHz
174.6
142.0
133.5
123.5
120.3
117.2
114.0
79.5
77.2 CDCl3
54.1
52.5
52.1
47.7
39.7
39.3
36.9
36.8
29.0
28.6
27.9
26.0
22.9
21.5
19.3
18.3
16.3
14.0
-3.6
-4.8
210 200 190 180 170 160 150 140 130 120 110 100 90 80 70 60 50 40 30 20 10 0 -10
f1 (ppm)

23
^{19}F NMR, $CDCl_3$
377 MHz

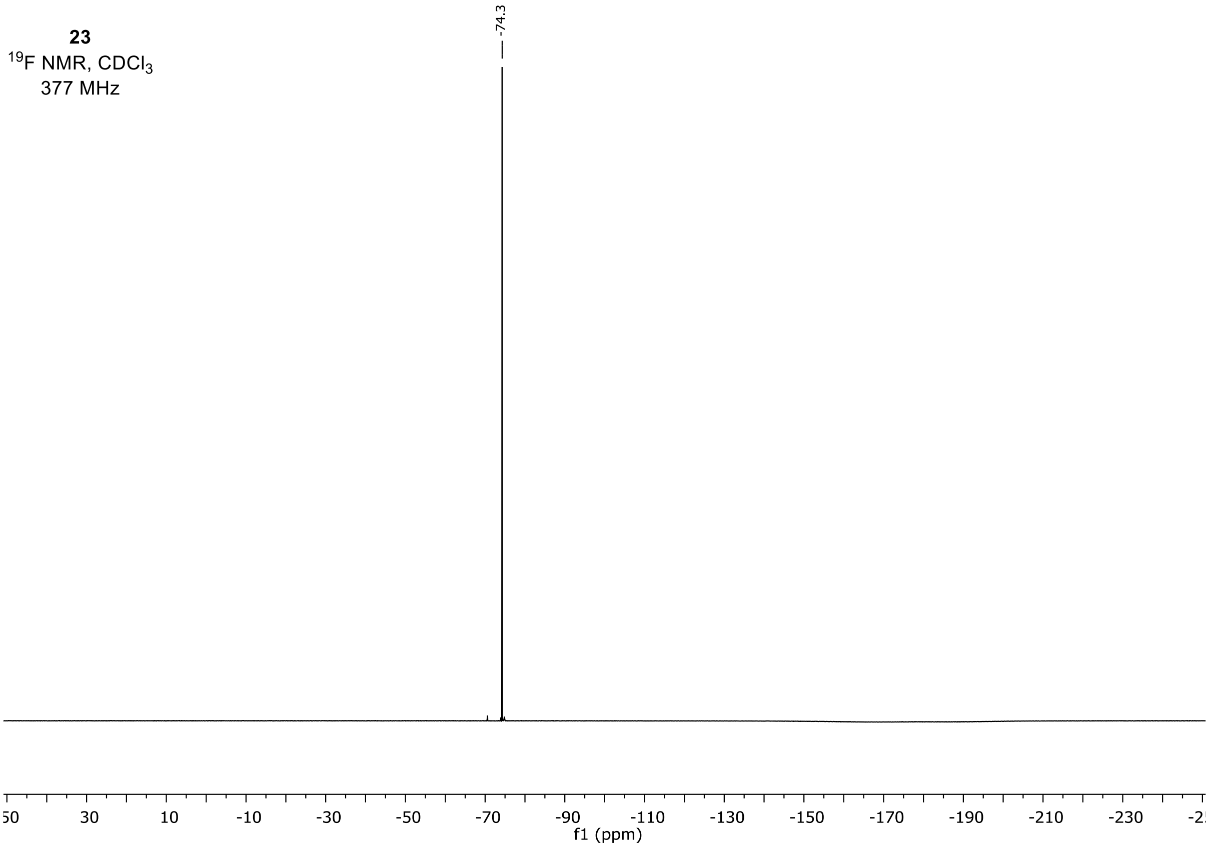

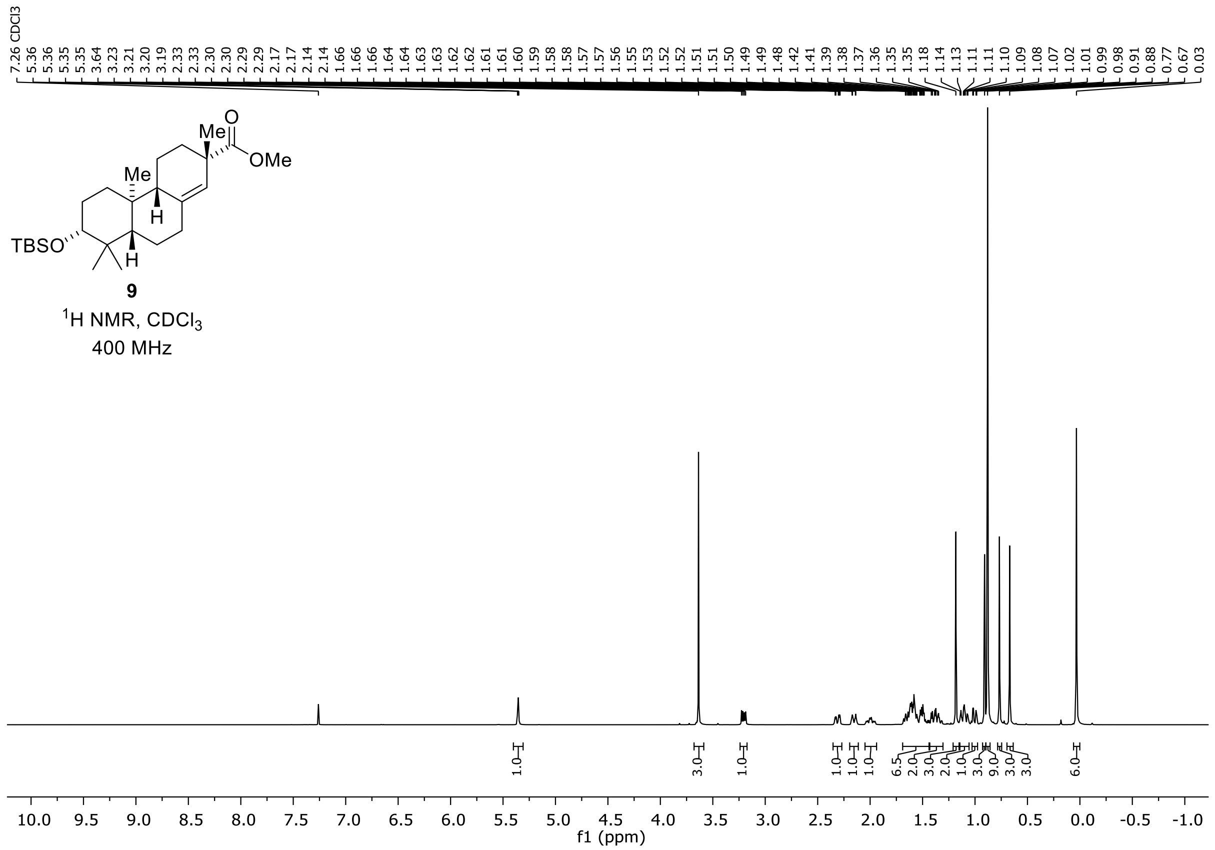
7.26 CDCl3
5.36 5.36 5.35 5.35 3.64 3.23 3.21 3.20 3.19 2.33 2.33 2.30 2.30 2.29 2.29 2.17 2.17 2.14 2.14 1.66 1.66 1.64 1.64 1.63 1.63 1.62 1.62 1.61 1.61 1.60 1.60 1.59 1.58 1.58 1.57 1.57 1.56 1.55 1.53 1.52 1.52 1.52 1.51 1.51 1.50 1.49 1.49 1.48 1.42 1.41 1.39 1.38 1.37 1.36 1.35 1.35 1.18 1.14 1.13 1.11 1.11 1.10 1.09 1.08 1.07 1.02 1.01 0.99 0.98 0.91 0.88 0.77 0.67 0.03
Me
O
OMe
Me
H
TBSO
H
9
^{1}H NMR, CDCl$_3$
400 MHz
1.0
3.0
1.0
1.0 1.0 1.0
6.5 2.0 3.0 2.0 1.0 3.0 9.0 3.0 3.0
6.0
10.0 9.5 9.0 8.5 8.0 7.5 7.0 6.5 6.0 5.5 5.0 4.5 4.0 3.5 3.0 2.5 2.0 1.5 1.0 0.5 0.0 -0.5 -1.0
f1 (ppm)

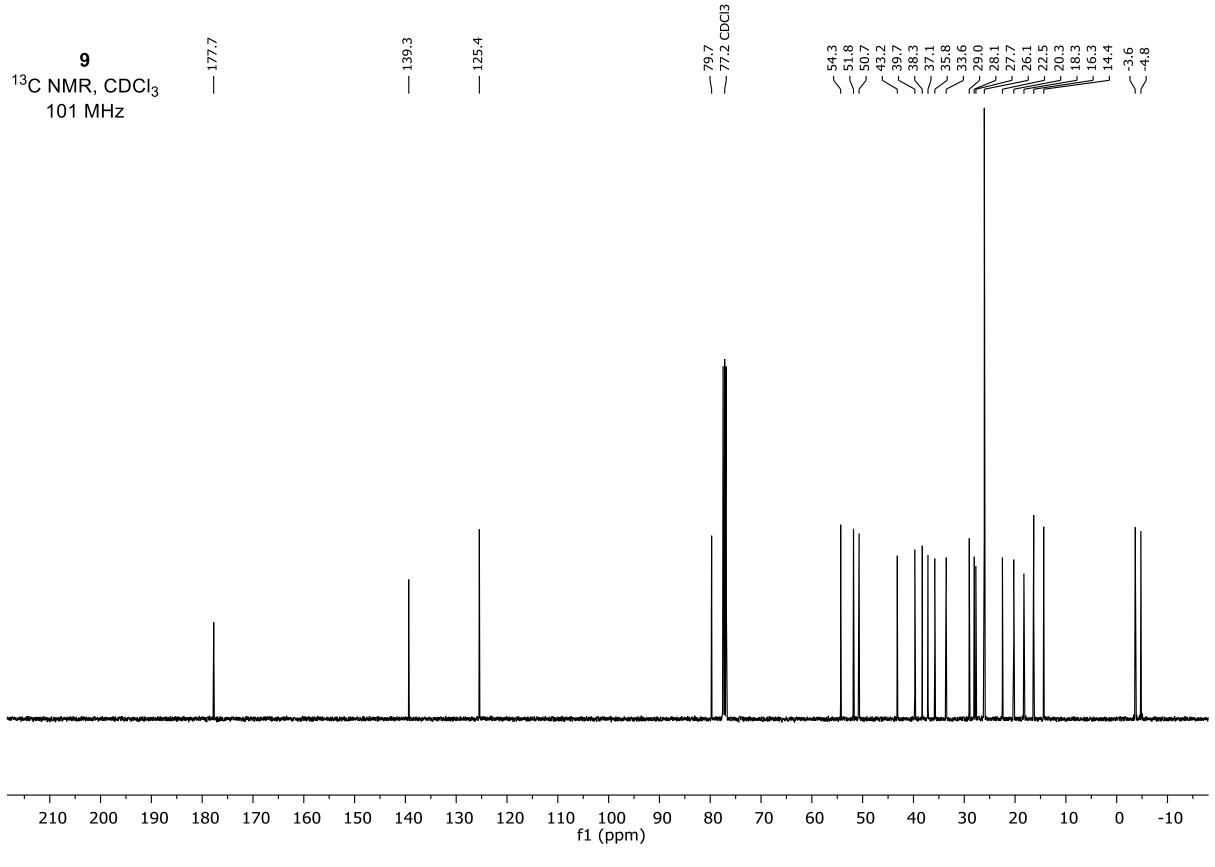
9
^{13}C NMR, CDCl$_3$
101 MHz
177.7
139.3
125.4
79.7
77.2 CDCl3
54.3 51.8 50.7 43.2 39.7 38.3 37.1 35.8 33.6 29.0 28.1 27.7 26.1 22.5 20.3 18.3 16.3 14.4
-3.6 -4.8
210 200 190 180 170 160 150 140 130 120 110 100 90 80 70 60 50 40 30 20 10 0 -10
f1 (ppm)

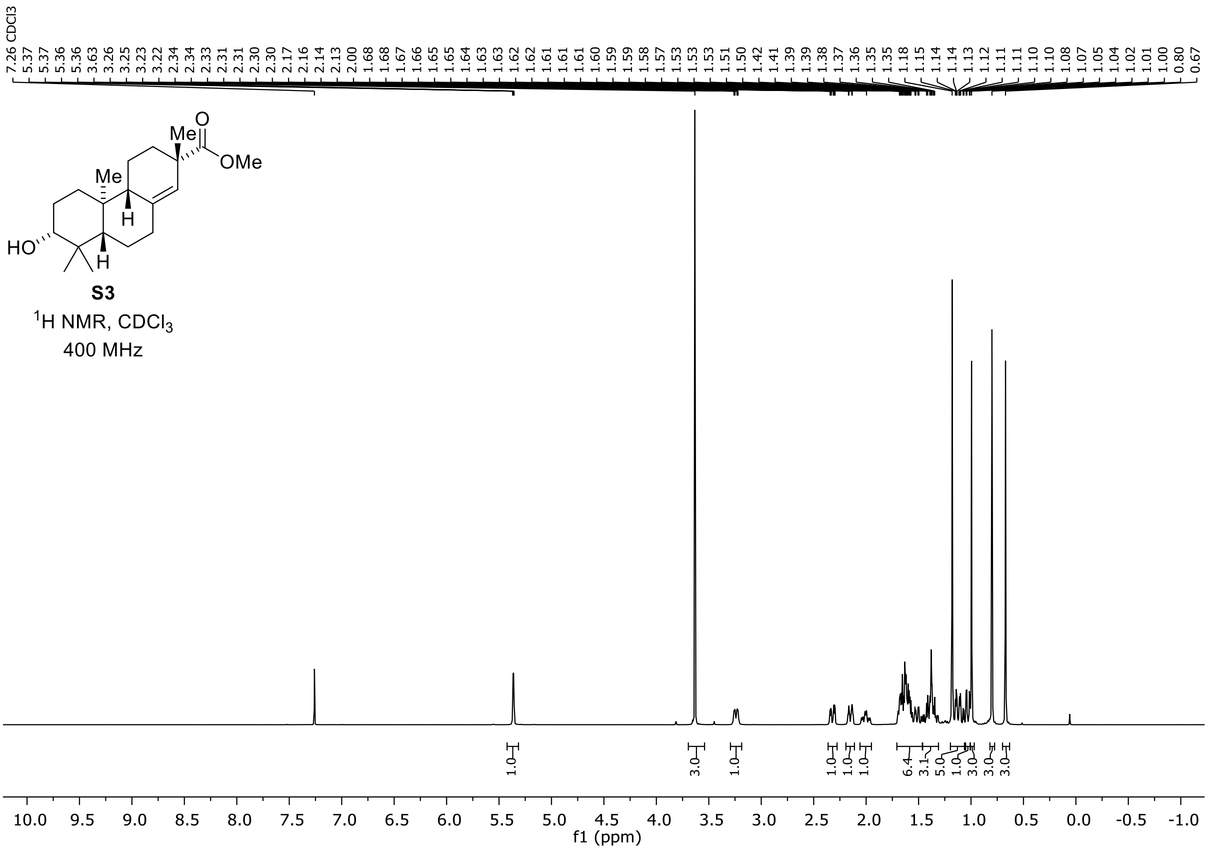
7.26 CDCl3
5.37
5.37
5.36
5.36
3.63
3.26
3.25
3.23
3.22
2.34
2.34
2.33
2.31
2.31
2.30
2.30
2.17
2.16
2.14
2.13
2.00
1.68
1.68
1.67
1.66
1.65
1.65
1.64
1.63
1.63
1.62
1.62
1.61
1.61
1.61
1.60
1.59
1.59
1.58
1.57
1.53
1.53
1.53
1.51
1.50
1.42
1.41
1.39
1.39
1.38
1.37
1.36
1.35
1.35
1.18
1.15
1.14
1.14
1.13
1.12
1.11
1.11
1.10
1.10
1.08
1.07
1.05
1.04
1.02
1.01
1.00
0.80
0.67
Me
O
OMe
Me
H
HO
H
S3
^{1}H NMR, $CDCl_3$
400 MHz
1.0
3.0
1.0
1.0
1.0
1.0
6.4
3.1
5.0
1.0
3.0
3.0
3.0
10.0 9.5 9.0 8.5 8.0 7.5 7.0 6.5 6.0 5.5 5.0 4.5 4.0 3.5 3.0 2.5 2.0 1.5 1.0 0.5 0.0 -0.5 -1.0
f1 (ppm)

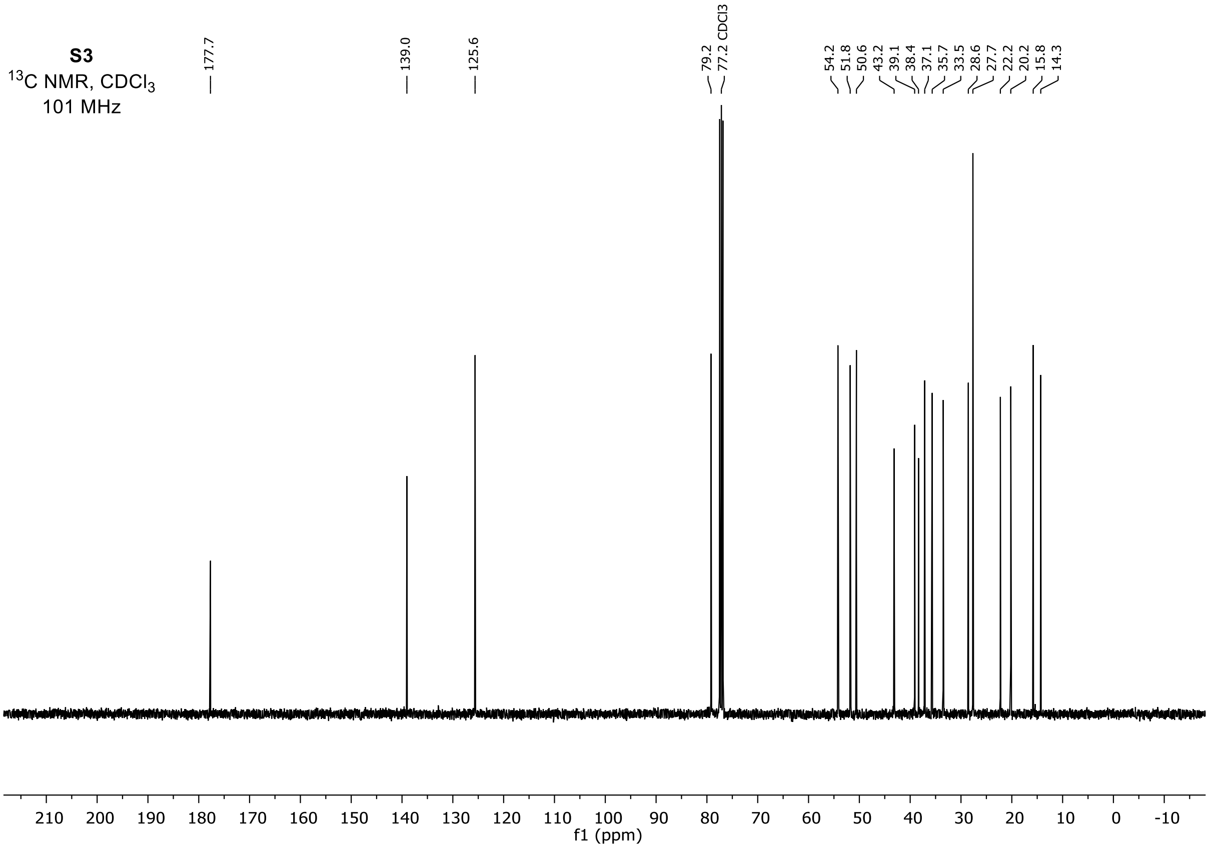
S3
^{13}C NMR, $CDCl_3$
101 MHz
177.7
139.0
125.6
79.2
77.2 CDCl3
54.2
51.8
50.6
43.2
39.1
38.4
37.1
35.7
33.5
28.6
27.7
22.2
20.2
15.8
14.3
210 200 190 180 170 160 150 140 130 120 110 100 90 80 70 60 50 40 30 20 10 0 -10
f1 (ppm)

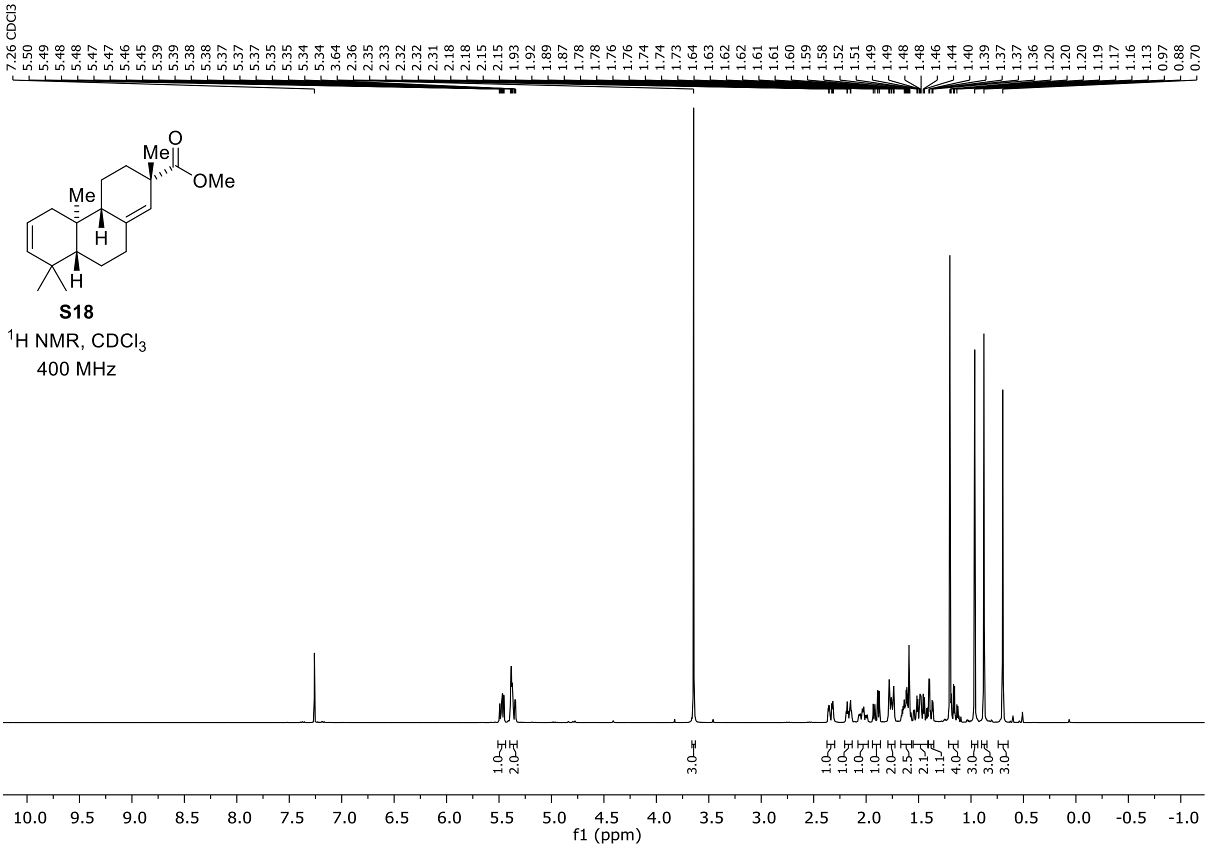
7.26 CDCl3
5.50 5.49 5.48 5.48 5.47 5.46 5.45 5.39 5.39 5.38 5.38 5.37 5.37 5.37 5.35 5.35 5.34 3.64 2.36 2.35 2.33 2.32 2.32 2.31 2.18 2.18 2.15 2.15 1.93 1.92 1.89 1.87 1.78 1.78 1.76 1.74 1.74 1.73 1.64 1.63 1.62 1.62 1.61 1.61 1.60 1.59 1.58 1.52 1.51 1.49 1.49 1.48 1.48 1.46 1.44 1.40 1.39 1.37 1.37 1.36 1.20 1.20 1.20 1.19 1.17 1.16 1.13 0.97 0.88 0.70
S18
1H NMR, CDCl3
400 MHz
f1 (ppm)

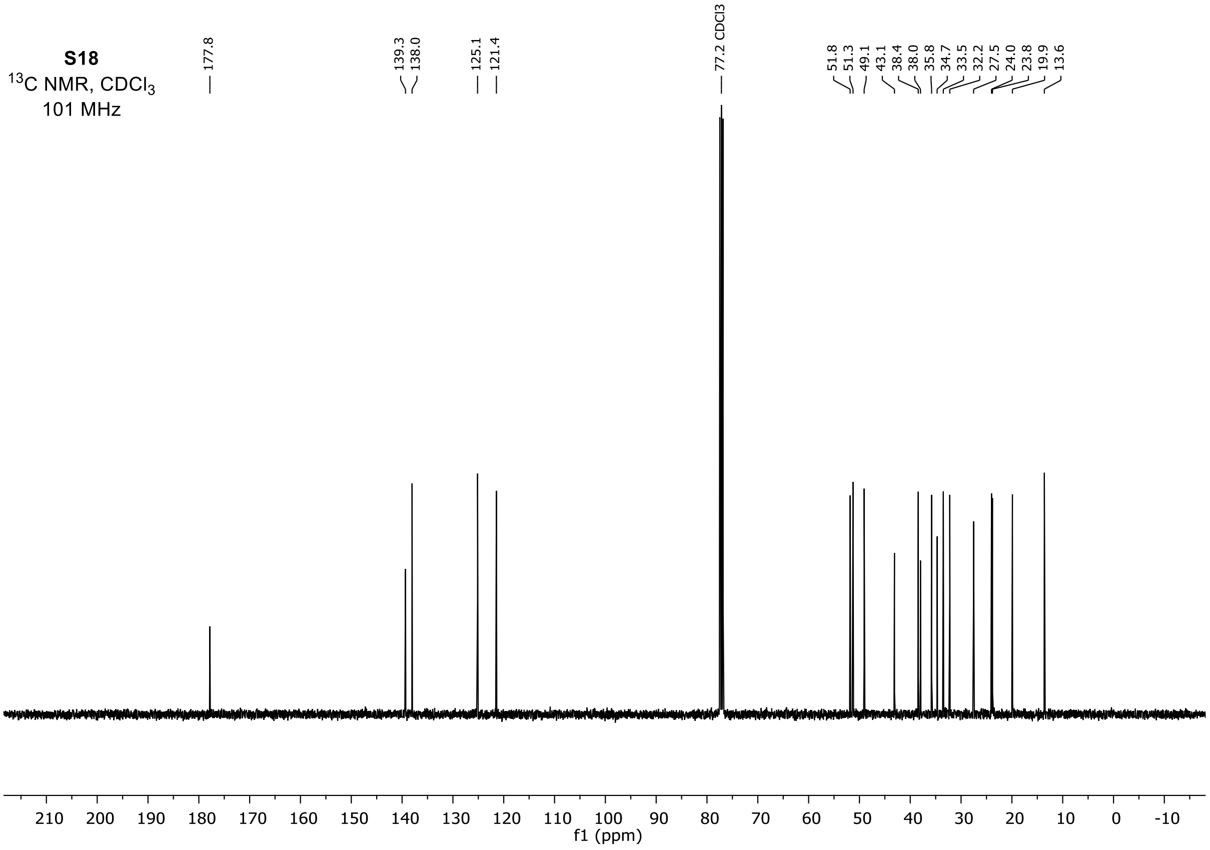
S18
13C NMR, CDCl3
101 MHz
177.8
139.3 138.0
125.1 121.4
77.2 CDCl3
51.8 51.3 49.1 43.1 38.4 38.0 35.8 34.7 33.5 32.2 27.5 24.0 23.8 19.9 13.6
f1 (ppm)

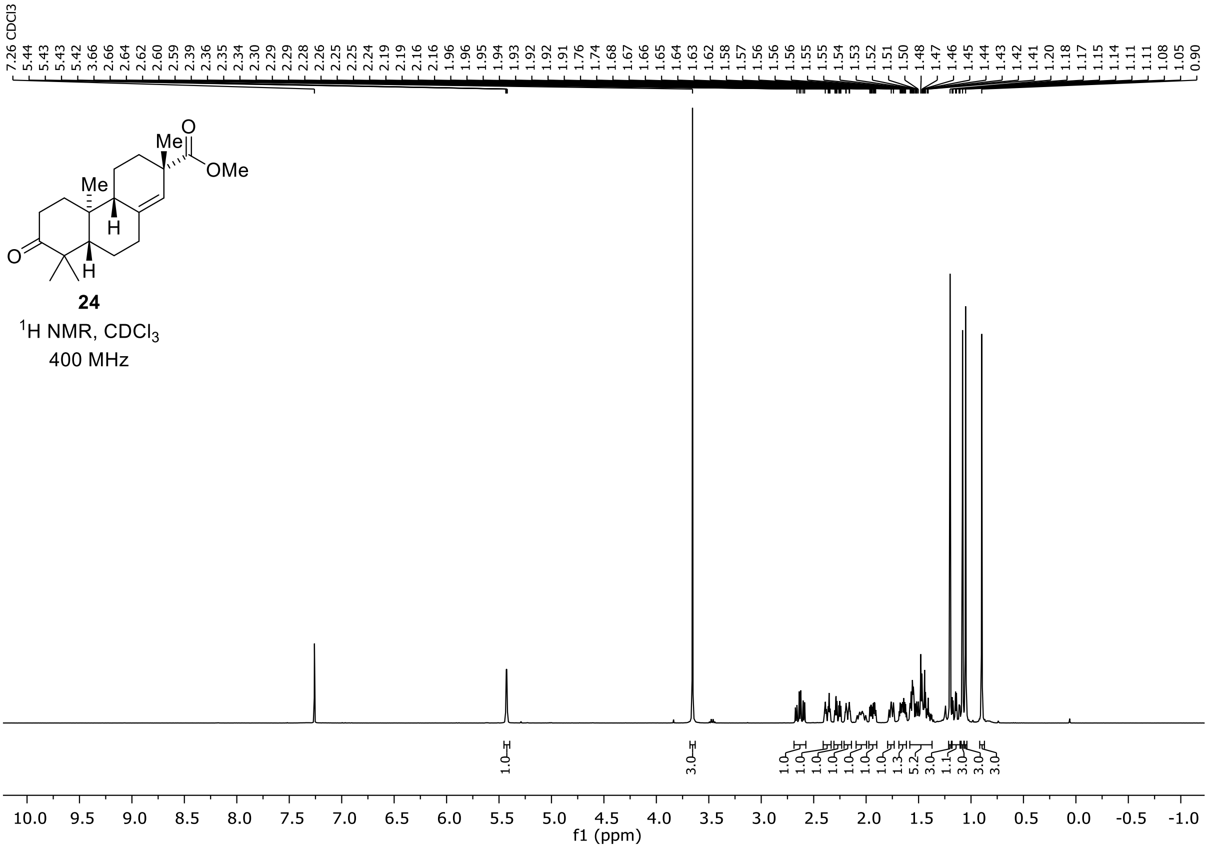

24
^{13}C NMR, $CDCl_3$
101 MHz

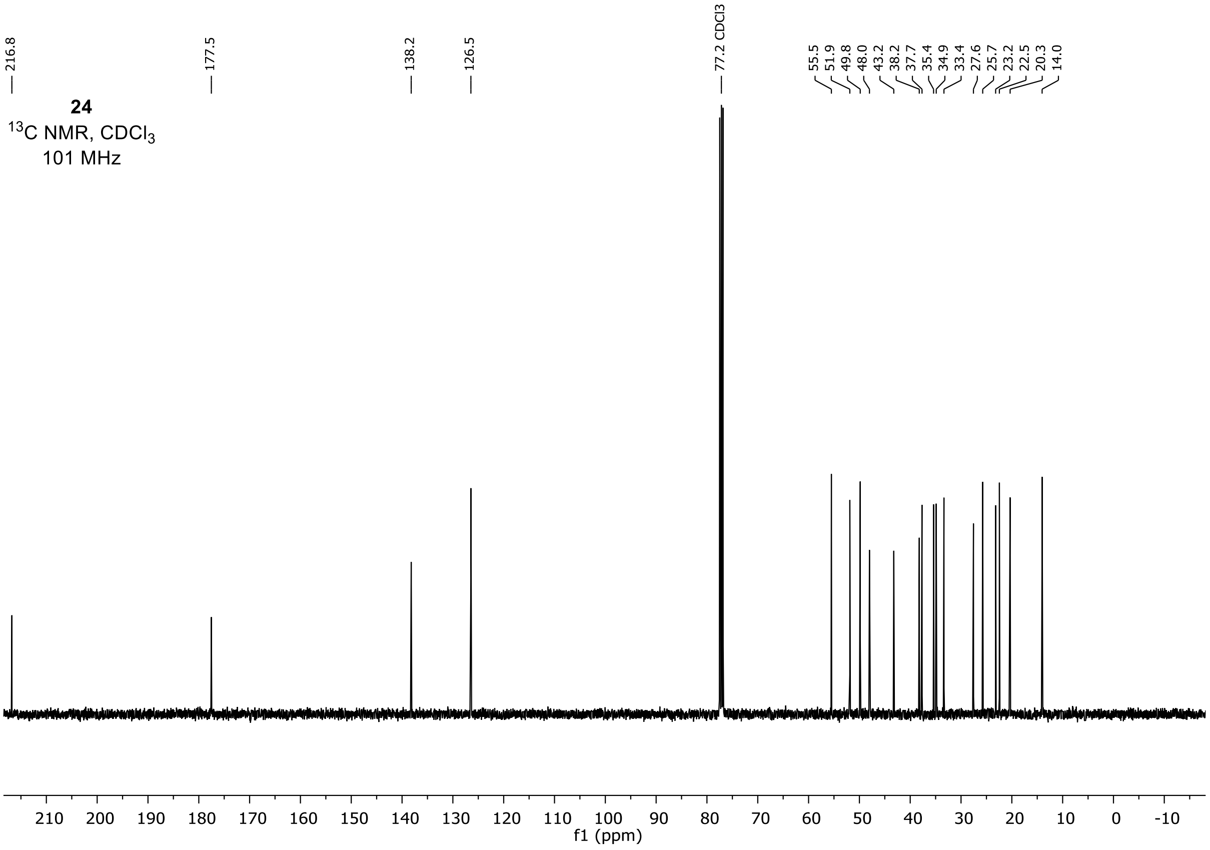

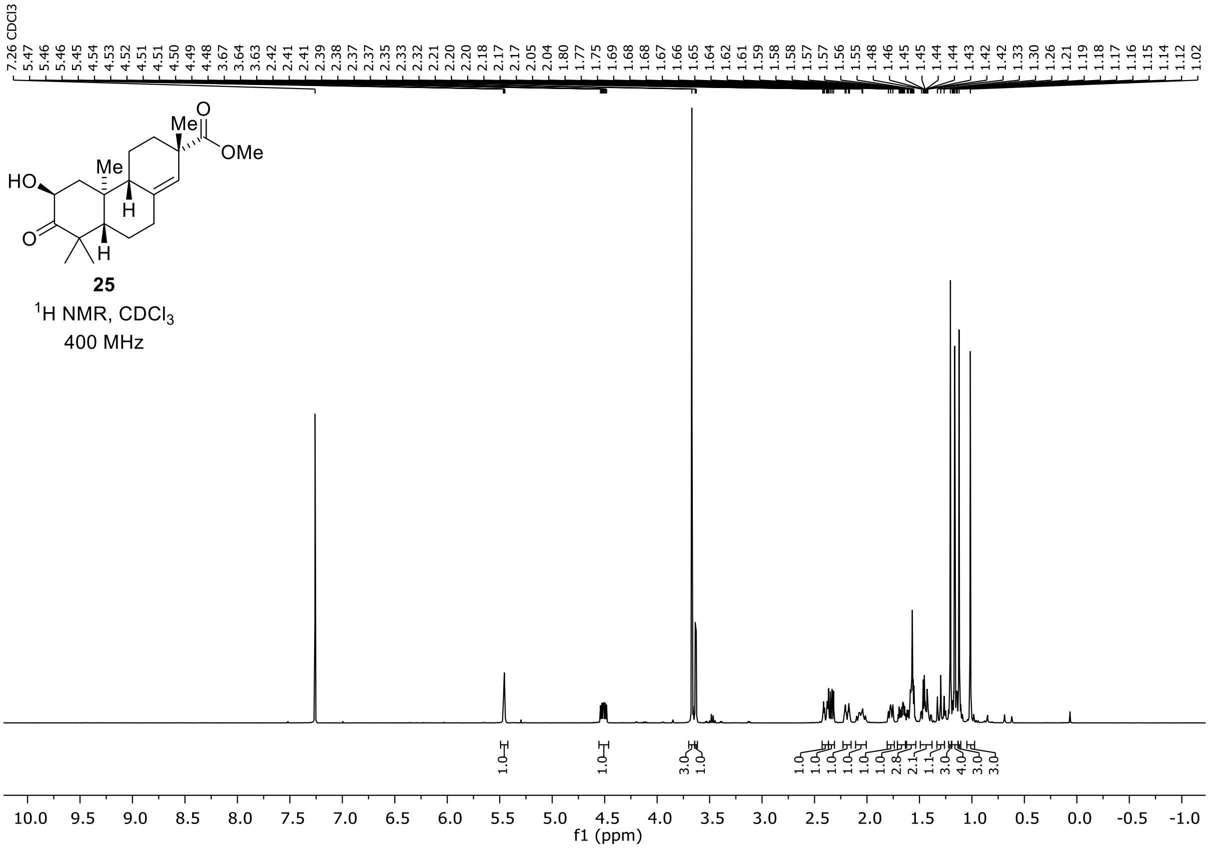
7.26 CDCl3
5.47
5.46
5.45
4.54
4.53
4.52
4.51
4.51
4.50
4.49
4.48
3.67
3.64
3.63
2.42
2.41
2.41
2.39
2.38
2.37
2.37
2.35
2.33
2.32
2.21
2.20
2.20
2.18
2.17
2.17
2.05
2.04
1.80
1.77
1.75
1.69
1.68
1.68
1.67
1.66
1.65
1.64
1.62
1.61
1.59
1.58
1.57
1.57
1.56
1.55
1.48
1.46
1.45
1.45
1.44
1.44
1.43
1.42
1.42
1.33
1.30
1.26
1.21
1.19
1.18
1.17
1.16
1.15
1.14
1.12
1.02
HO
O
Me
Me
O
OMe
H
H
25
1H NMR, CDCl3
400 MHz
1.0
1.0
3.0
1.0
1.0
1.0
1.0
1.0
1.0
1.0
2.8
2.1
1.1
3.0
4.0
3.0
3.0
10.0 9.5 9.0 8.5 8.0 7.5 7.0 6.5 6.0 5.5 5.0 4.5 4.0 3.5 3.0 2.5 2.0 1.5 1.0 0.5 0.0 -0.5 -1.0
f1 (ppm)

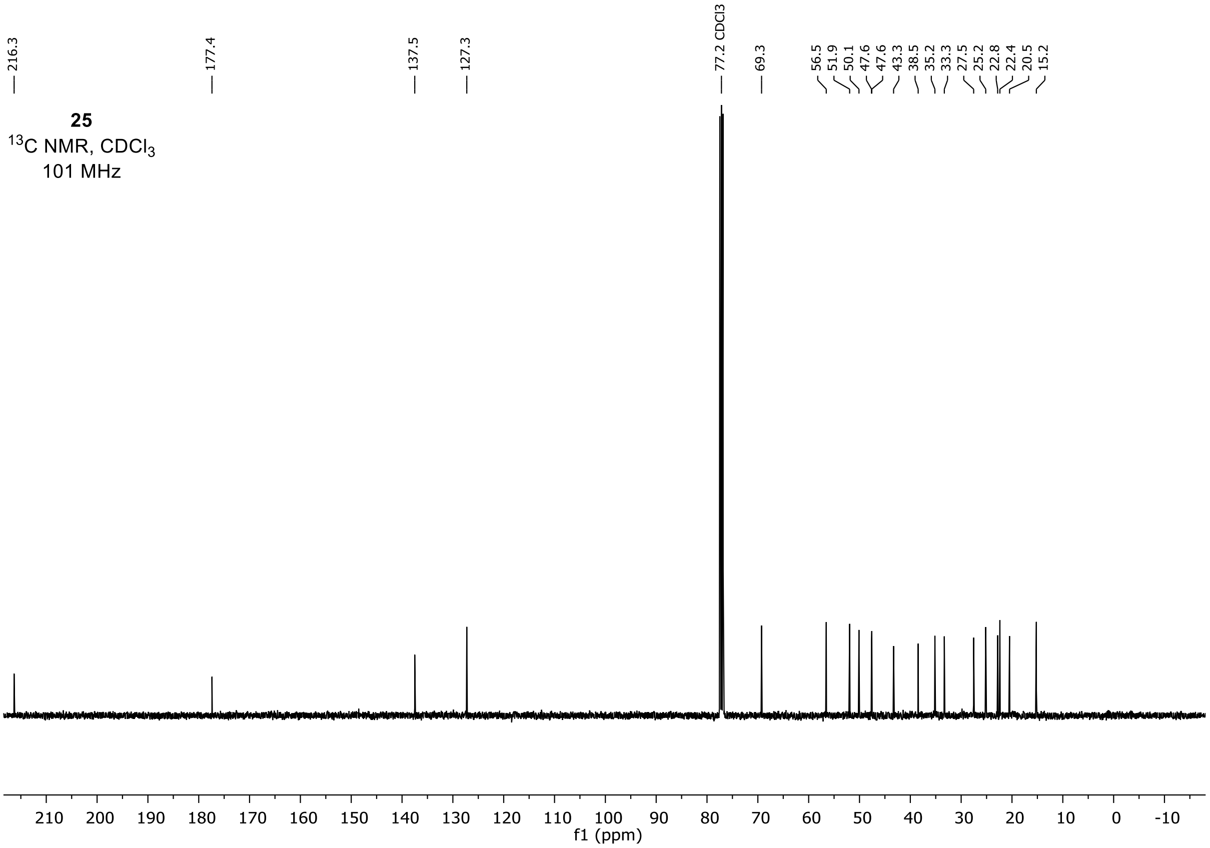
216.3
177.4
137.5
127.3
77.2 CDCl3
69.3
56.5
51.9
50.1
47.6
47.6
43.3
38.5
35.2
33.3
27.5
25.2
22.8
22.4
20.5
15.2
25
13C NMR, CDCl3
101 MHz
210 200 190 180 170 160 150 140 130 120 110 100 90 80 70 60 50 40 30 20 10 0 -10
f1 (ppm)

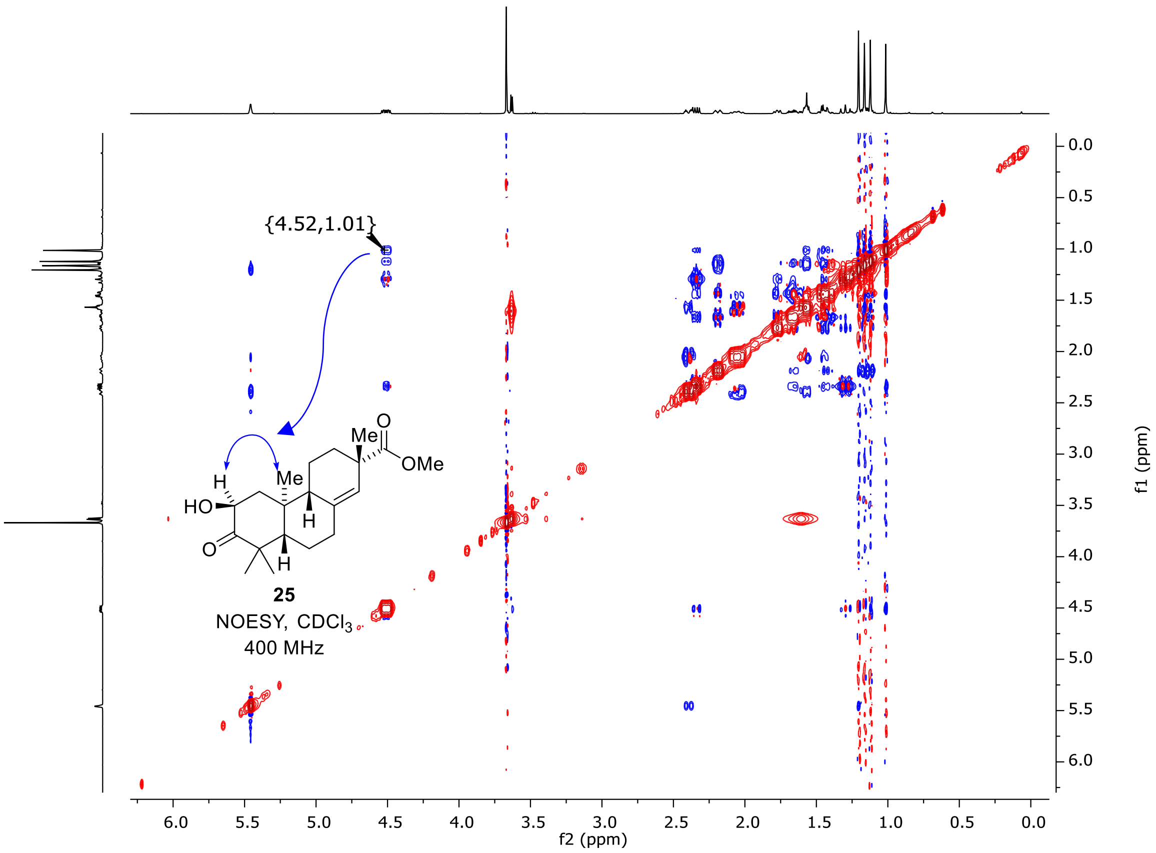
{4.52,1.01}
Me
O
OMe
H
Me
HO
H
O
H
25
NOESY, CDCl3
400 MHz
f2 (ppm)
f1 (ppm)

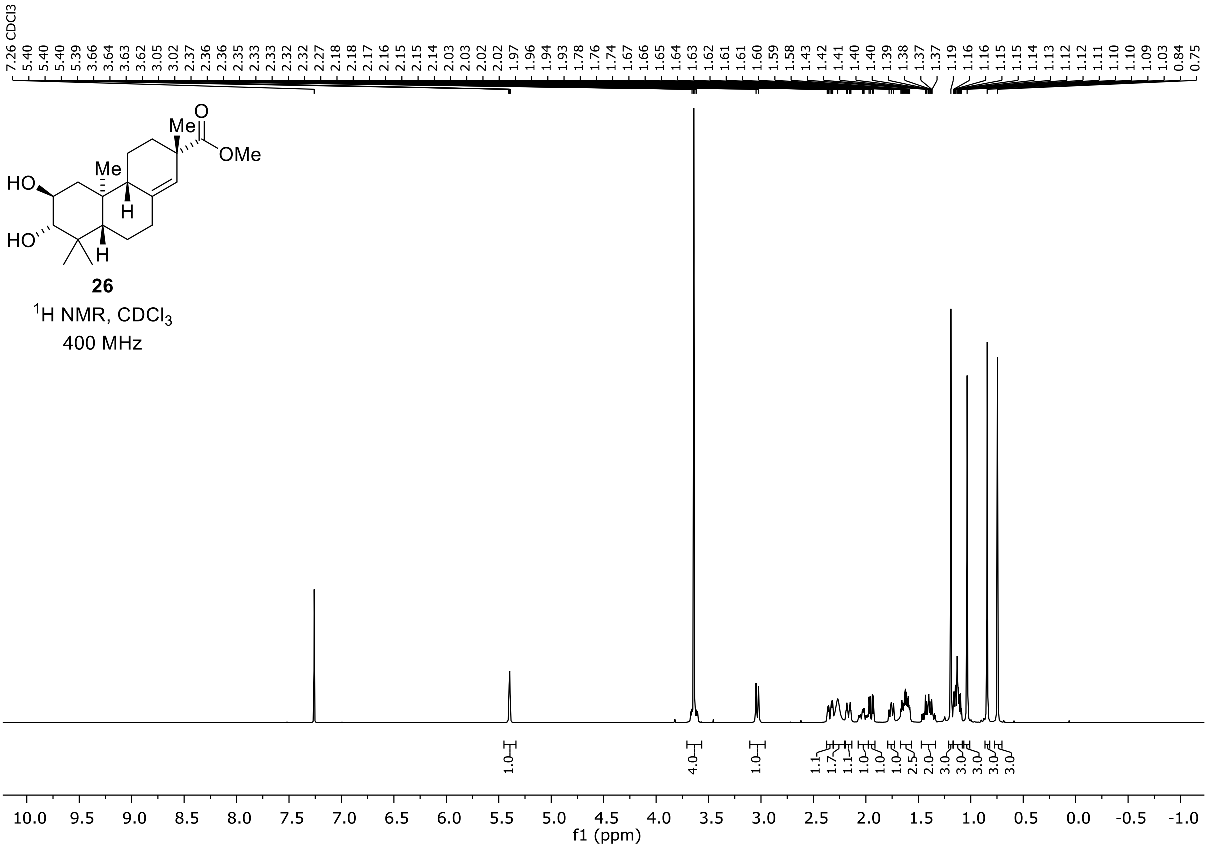
26
^{1}H NMR, CDCl$_3$
400 MHz
f1 (ppm)

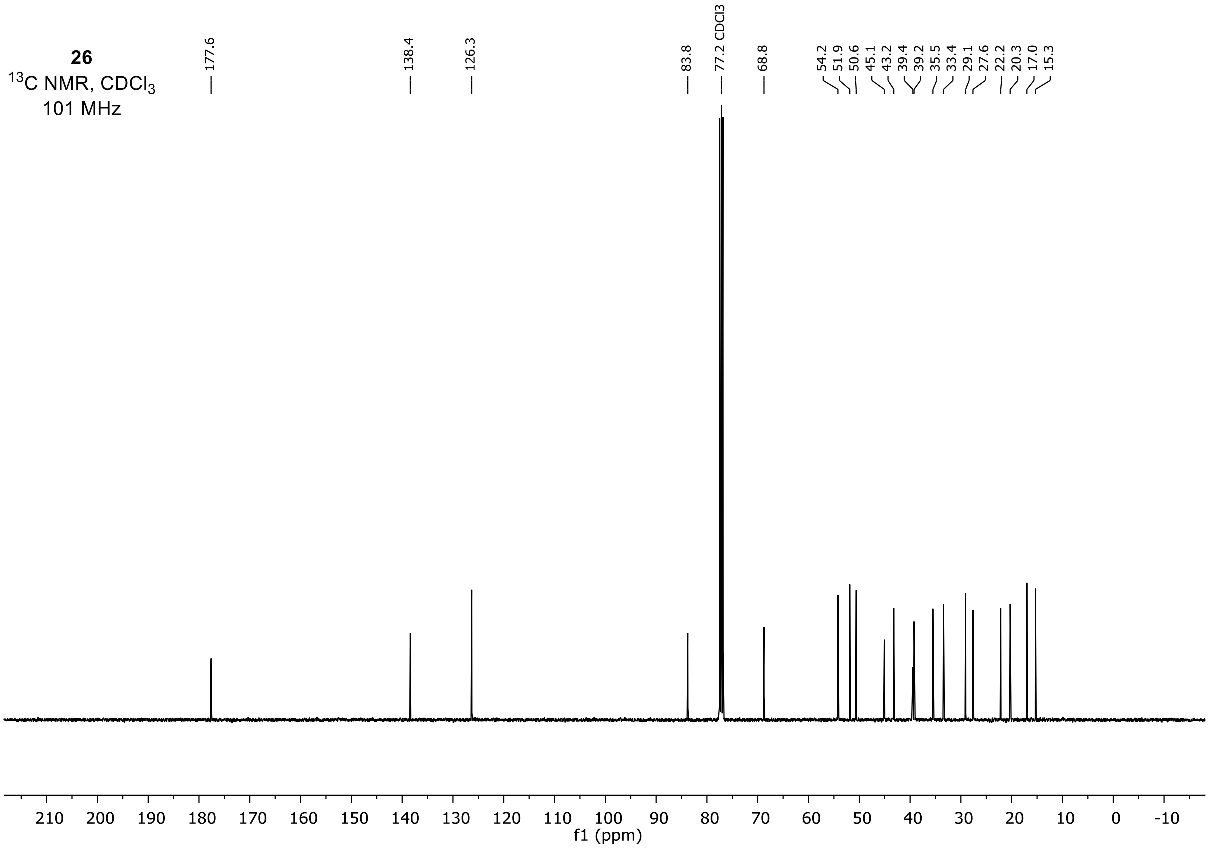
26
^{13}C NMR, CDCl$_3$
101 MHz
177.6
138.4
126.3
83.8
77.2 CDCl3
68.8
54.2
51.9
50.6
45.1
43.2
39.4
39.2
35.5
33.4
29.1
27.6
22.2
20.3
17.0
15.3
f1 (ppm)

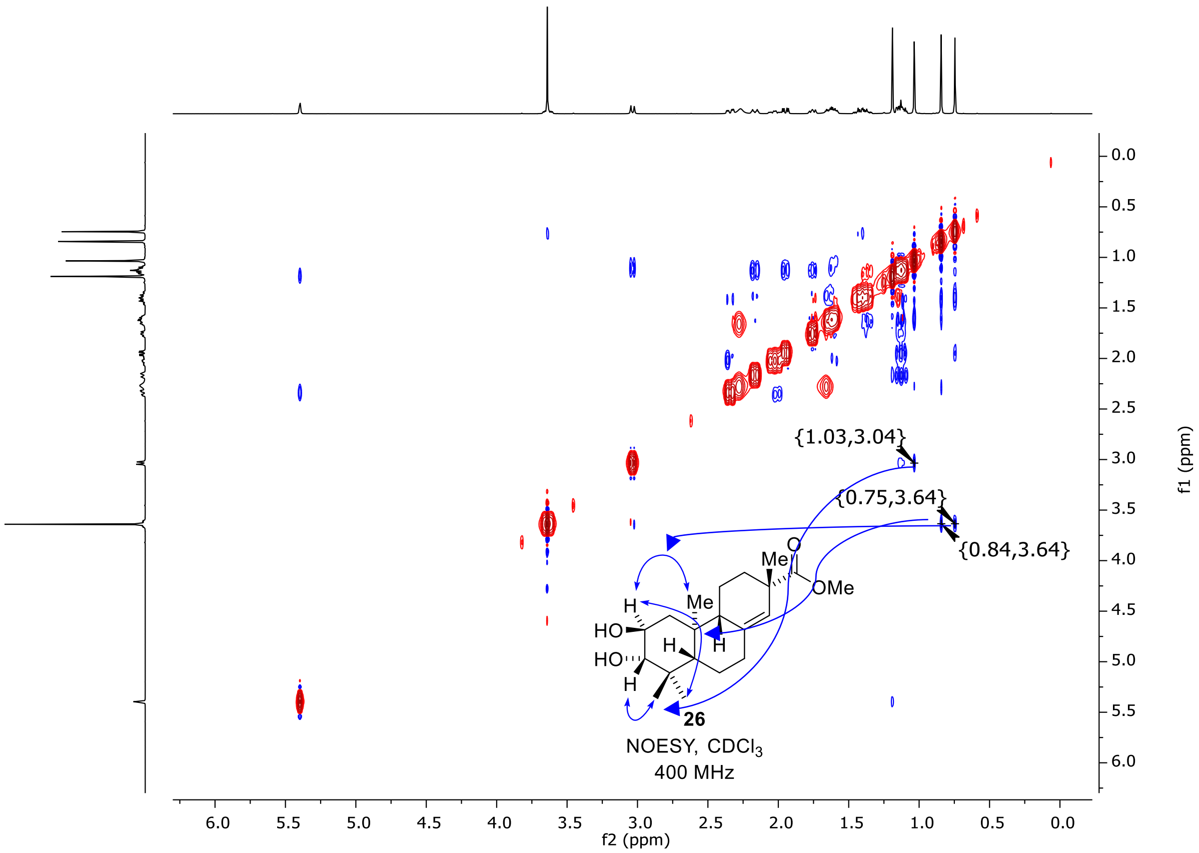
{1.03,3.04}
{0.75,3.64}
{0.84,3.64}
Me
O
OMe
H
Me
HO
HO
H
H
H
26
NOESY, CDCl3
400 MHz
f1 (ppm)
f2 (ppm)

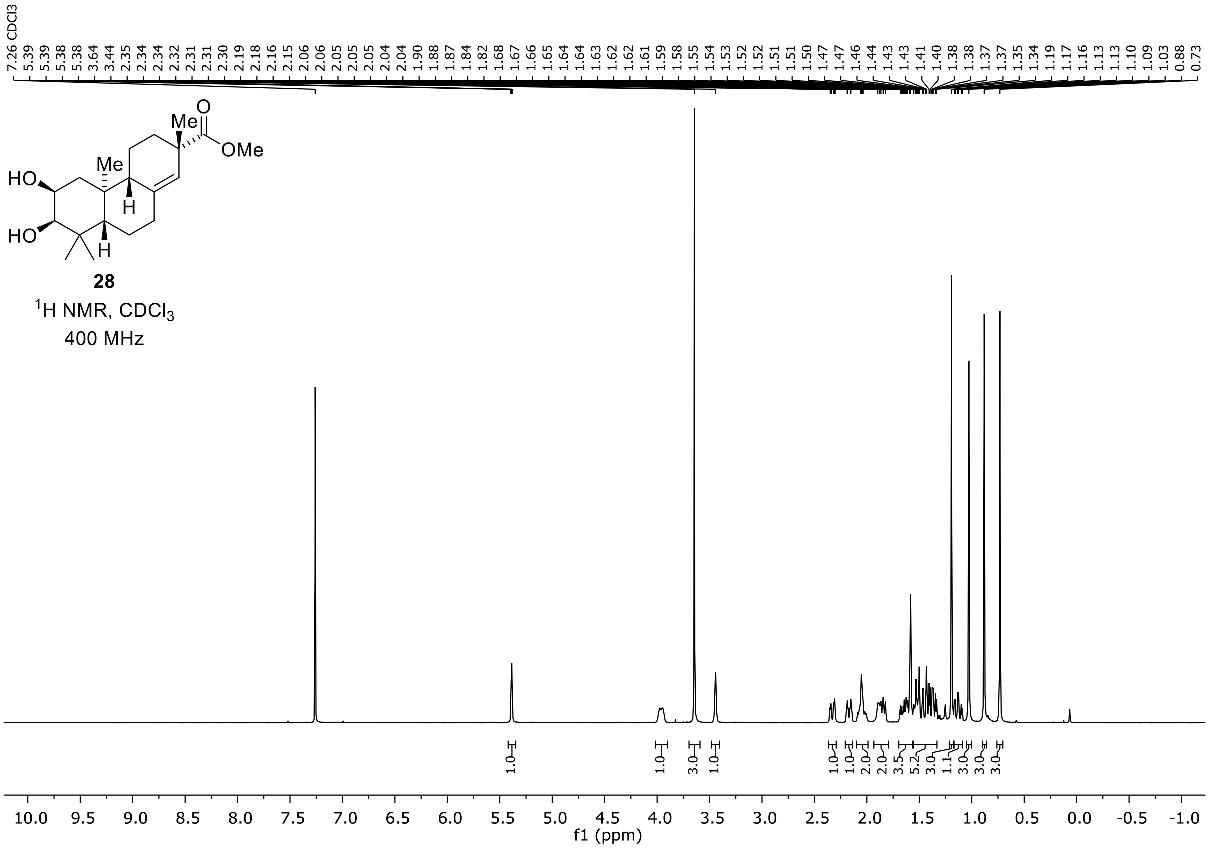
7.26 CDCl3
5.39
5.39
5.38
5.38
3.64
3.44
2.35
2.34
2.34
2.34
2.32
2.31
2.31
2.30
2.19
2.18
2.16
2.15
2.06
2.06
2.05
2.05
2.05
2.04
2.04
1.90
1.88
1.87
1.84
1.82
1.68
1.67
1.66
1.65
1.64
1.64
1.63
1.62
1.62
1.61
1.59
1.58
1.55
1.54
1.53
1.52
1.52
1.51
1.51
1.50
1.47
1.47
1.46
1.44
1.43
1.43
1.41
1.40
1.38
1.38
1.37
1.37
1.35
1.34
1.19
1.17
1.16
1.13
1.13
1.10
1.09
1.03
0.88
0.73
O
Me
OMe
Me
HO
H
HO
H
28
¹H NMR, CDCl₃
400 MHz
1.0
1.0
3.0
1.0
1.0
1.0
2.0
2.0
3.5
5.2
3.0
1.1
3.0
3.0
3.0
10.0 9.5 9.0 8.5 8.0 7.5 7.0 6.5 6.0 5.5 5.0 4.5 4.0 3.5 3.0 2.5 2.0 1.5 1.0 0.5 0.0 -0.5 -1.0
f1 (ppm)

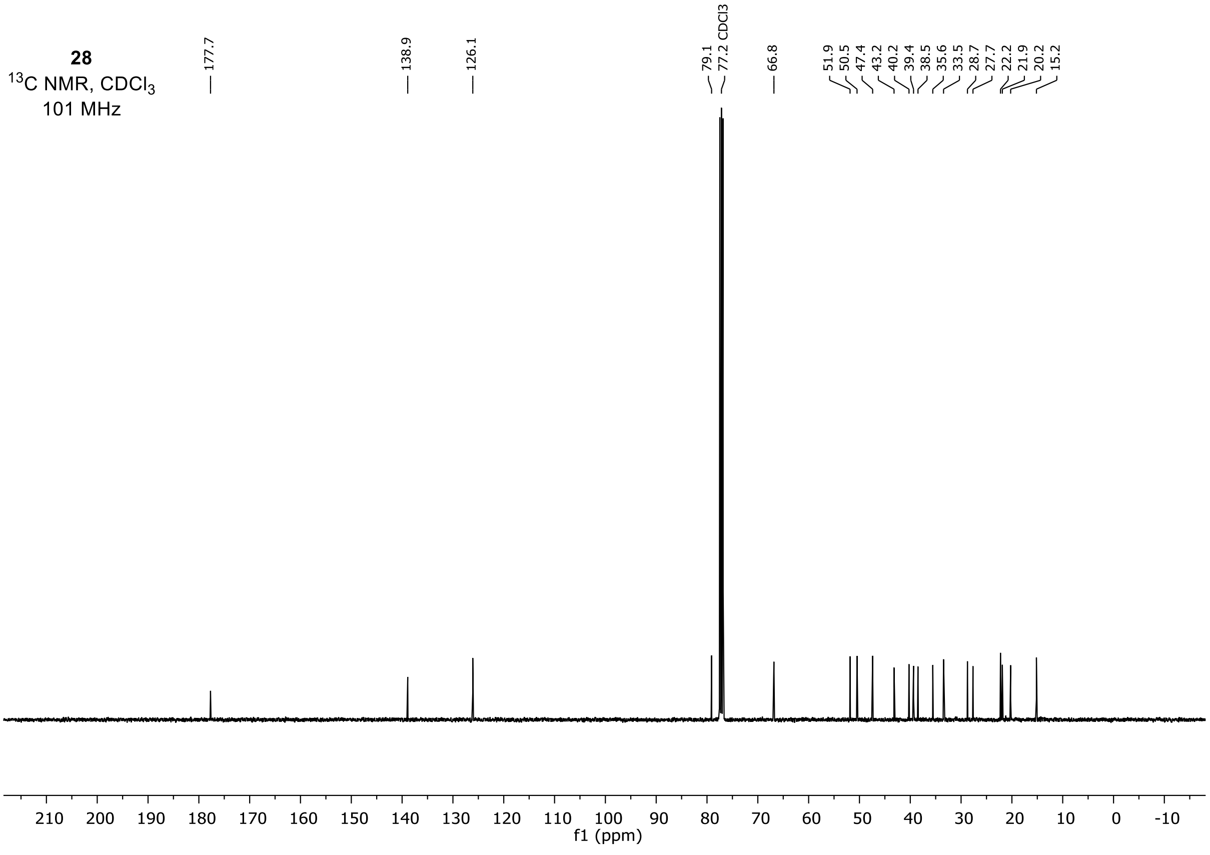
28
¹³C NMR, CDCl₃
101 MHz
177.7
138.9
126.1
79.1
77.2 CDCl3
66.8
51.9
50.5
47.4
43.2
40.2
39.4
38.5
35.6
33.5
28.7
27.7
22.2
21.9
20.2
15.2
210 200 190 180 170 160 150 140 130 120 110 100 90 80 70 60 50 40 30 20 10 0 -10
f1 (ppm)

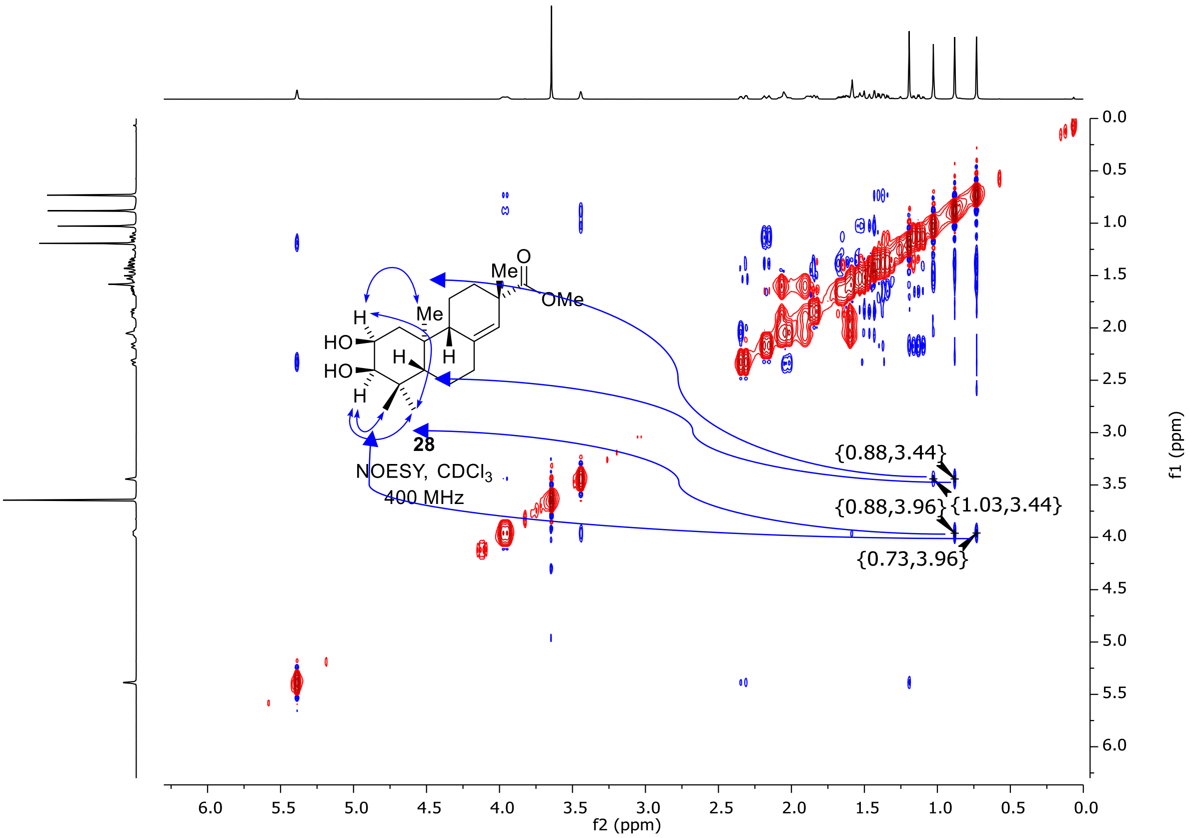

HO
HO
H
H
Me
H
H
Me
O
OMe
28
NOESY, CDCl3
400 MHz
{0.88,3.44}
{0.88,3.96}
{1.03,3.44}
{0.73,3.96}
f2 (ppm)
f1 (ppm)

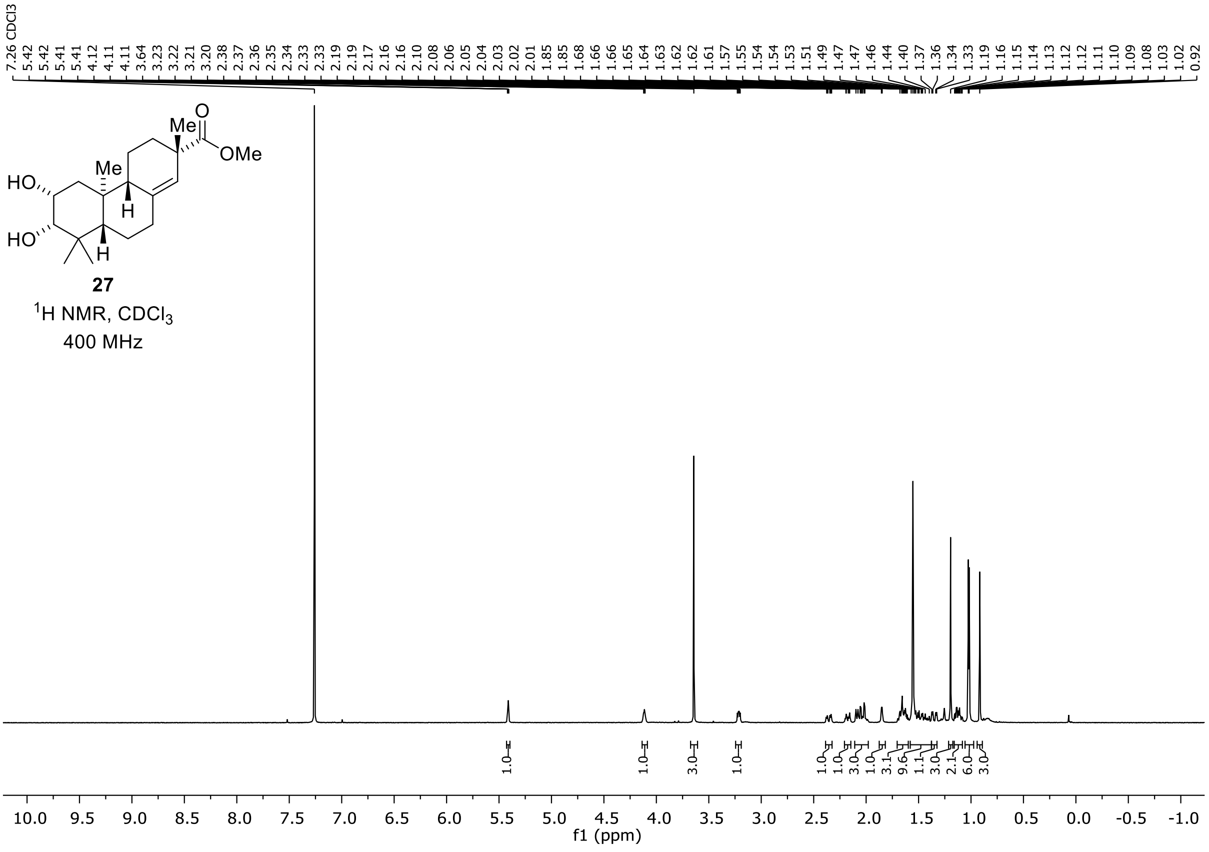
7.26 CDCl3
5.42
5.42
5.41
5.41
4.12
4.11
4.11
3.64
3.23
3.22
3.21
3.20
2.38
2.37
2.36
2.35
2.34
2.33
2.33
2.19
2.19
2.17
2.16
2.16
2.10
2.08
2.06
2.05
2.04
2.03
2.03
2.02
2.01
1.85
1.85
1.68
1.66
1.66
1.65
1.64
1.63
1.62
1.62
1.61
1.57
1.55
1.54
1.54
1.53
1.51
1.49
1.47
1.47
1.46
1.44
1.40
1.37
1.36
1.34
1.33
1.19
1.16
1.15
1.14
1.13
1.12
1.12
1.11
1.10
1.09
1.08
1.03
1.02
0.92
Me
O
OMe
Me
HO
H
HO
H
27
^{1}H NMR, $CDCl_3$
400 MHz
1.0
1.0
3.0
1.0
1.0
1.0
3.0
1.0
3.1
9.6
1.1
3.0
2.1
6.0
3.0
10.0 9.5 9.0 8.5 8.0 7.5 7.0 6.5 6.0 5.5 5.0 4.5 4.0 3.5 3.0 2.5 2.0 1.5 1.0 0.5 0.0 -0.5 -1.0
f1 (ppm)

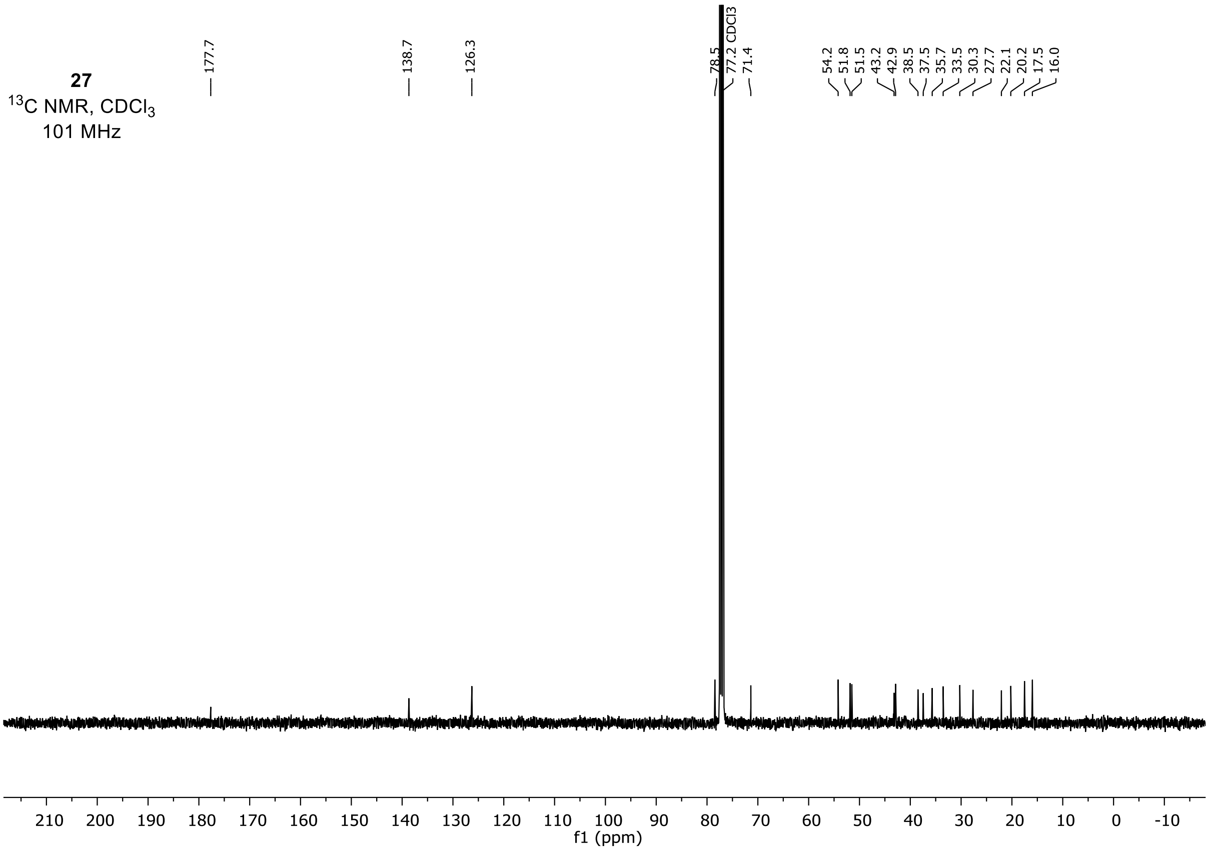
27
^{13}C NMR, $CDCl_3$
101 MHz
177.7
138.7
126.3
78.5
77.2 CDCl3
71.4
54.2
51.8
51.5
43.2
42.9
38.5
37.5
35.7
33.5
30.3
27.7
22.1
20.2
17.5
16.0
210 200 190 180 170 160 150 140 130 120 110 100 90 80 70 60 50 40 30 20 10 0 -10
f1 (ppm)

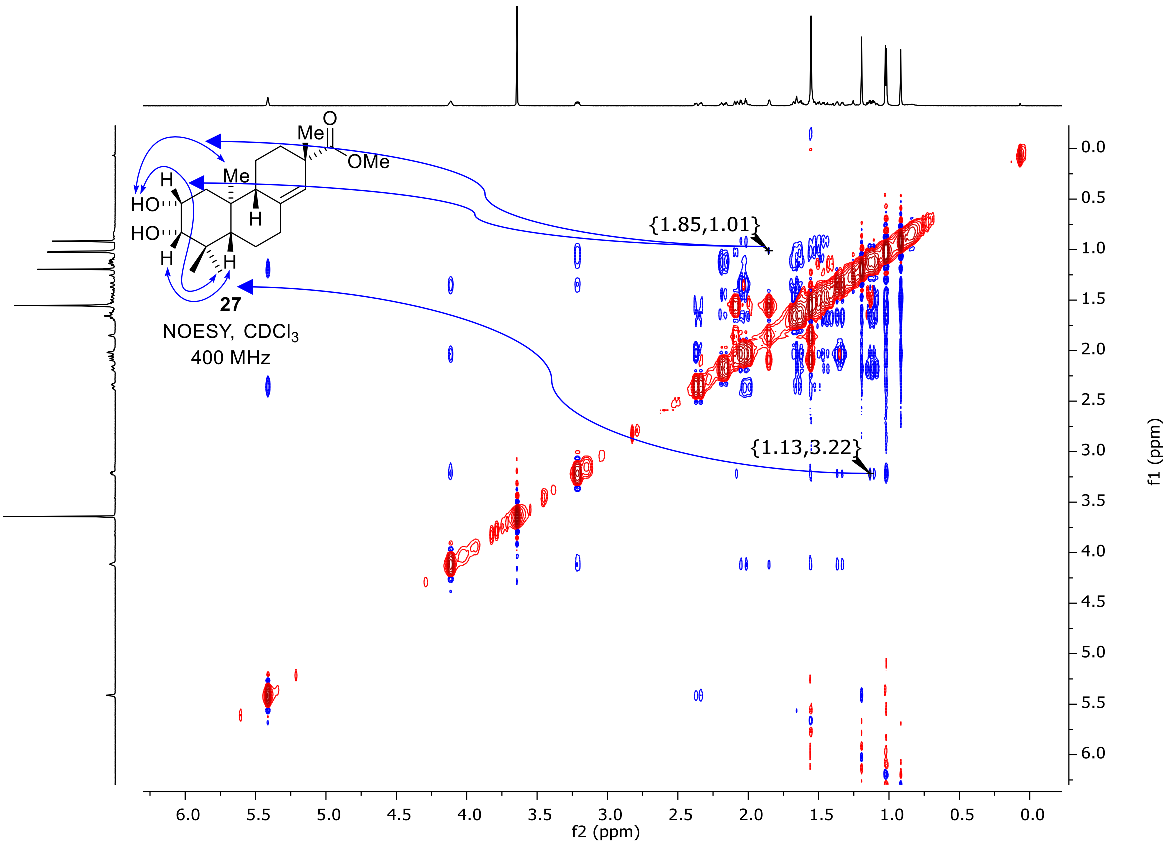

O
Me
OMe
Me
H
HO
HO
H
H
H
27
NOESY, CDCl3
400 MHz
{1.85,1.01}
{1.13,3.22}
f1 (ppm)
f2 (ppm)

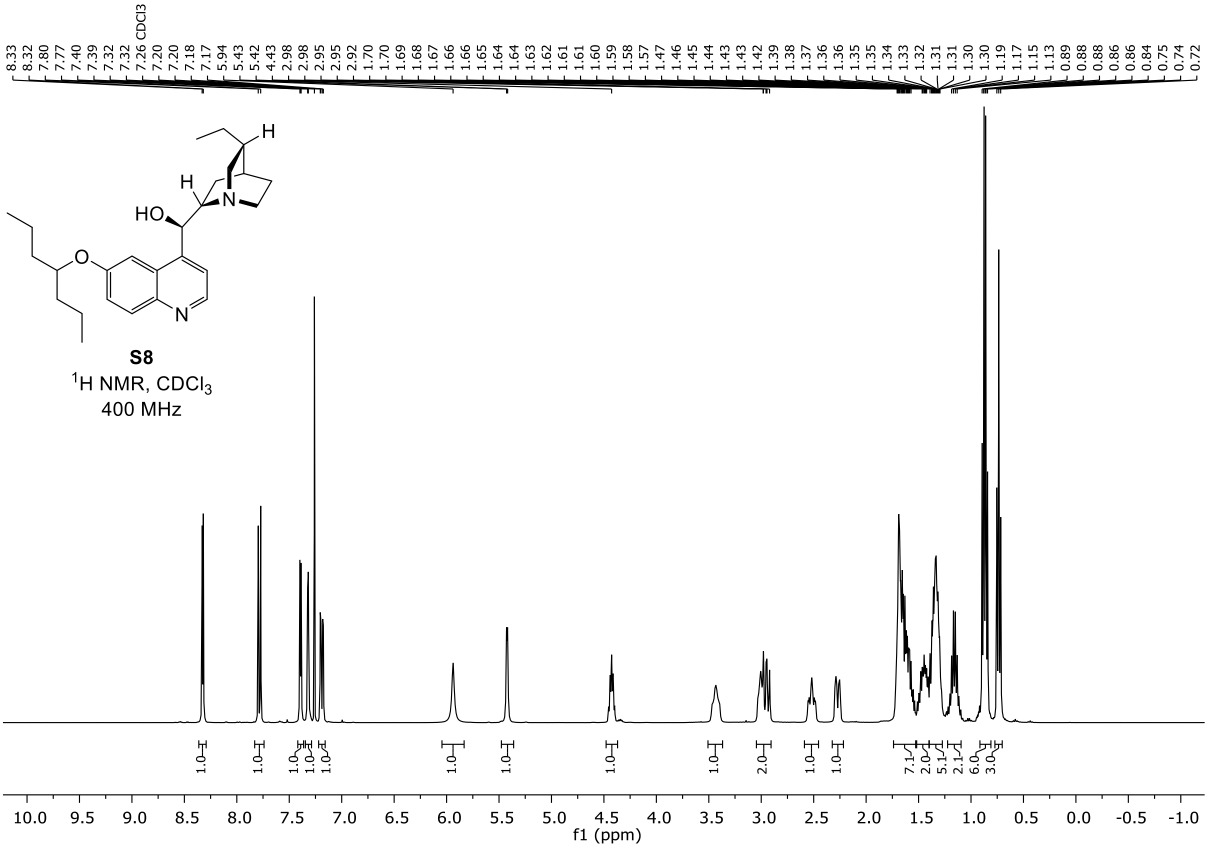

S8
^{1}H NMR, $CDCl_3$
400 MHz

S8
^{13}C NMR, $CDCl_3$
101 MHz

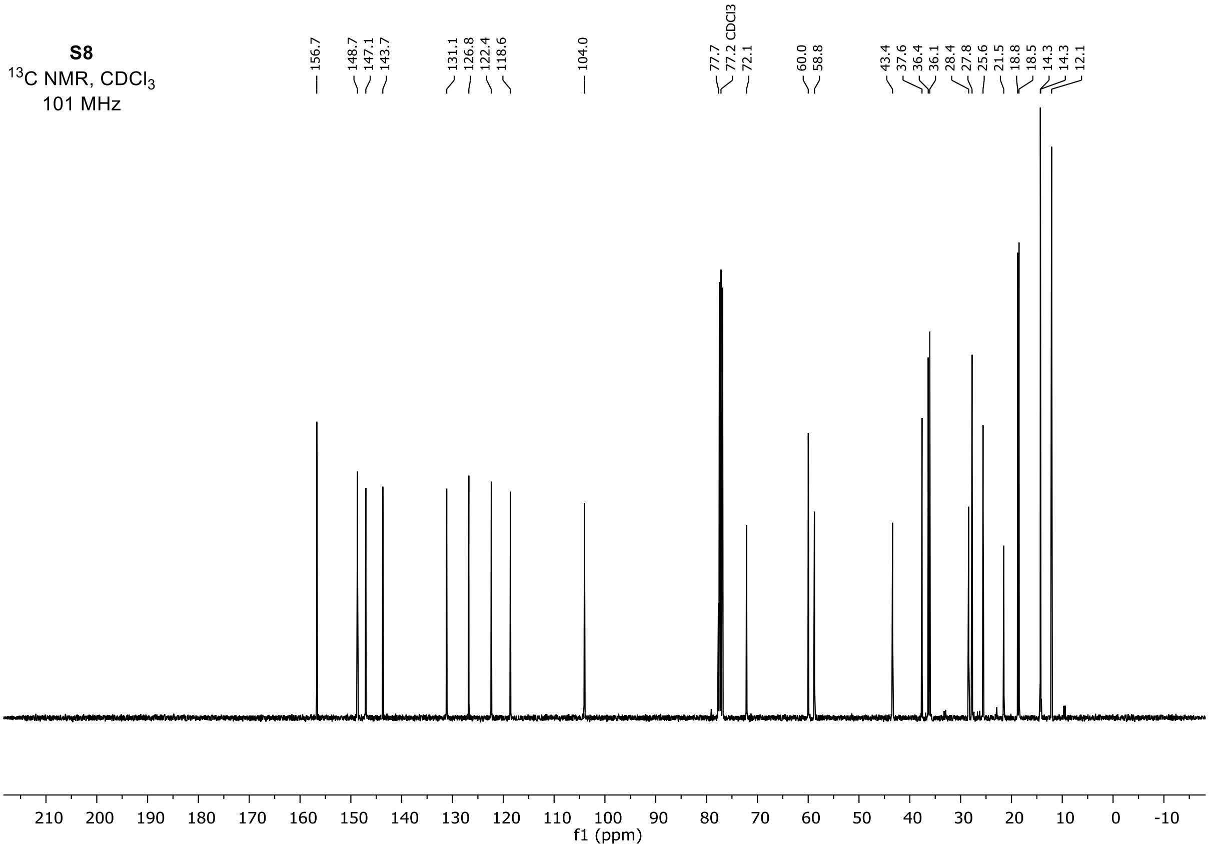

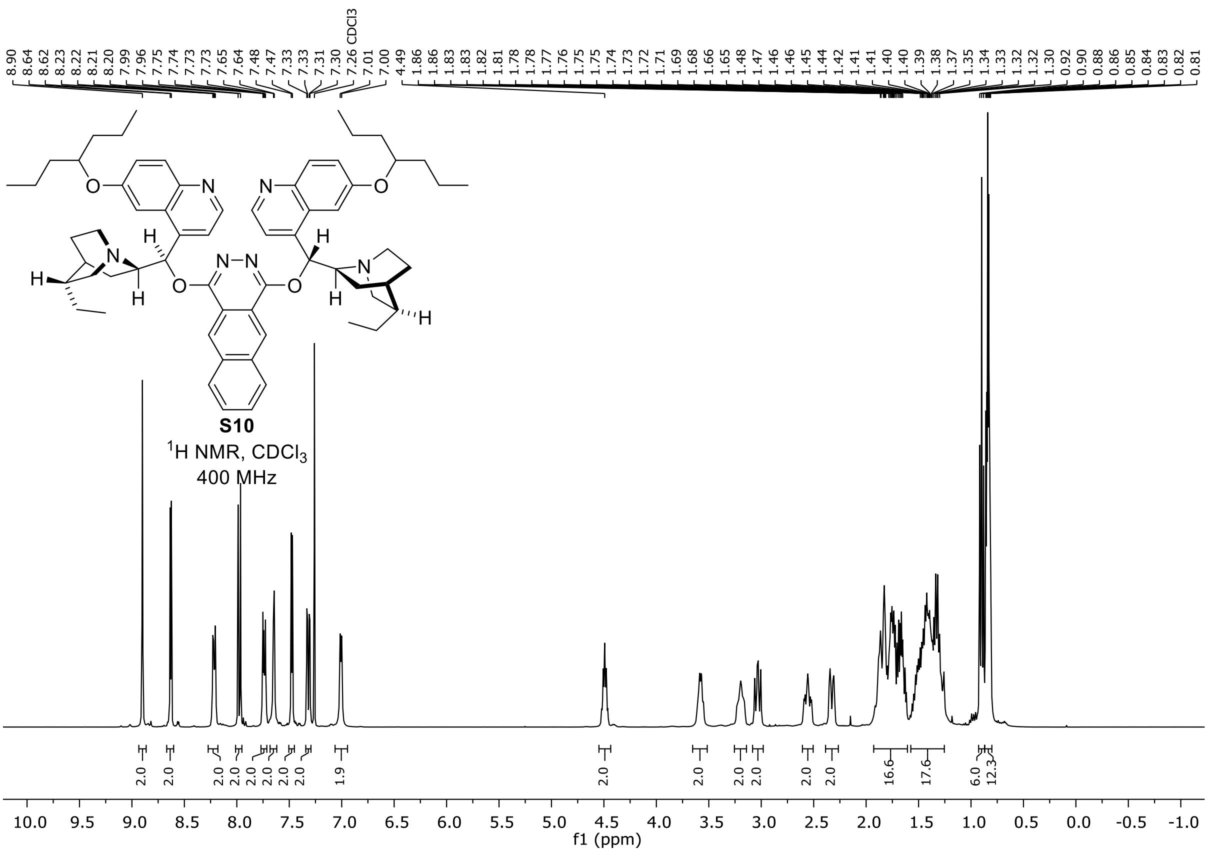
8.90 8.64 8.62 8.23 8.22 8.21 8.20 7.99 7.96 7.75 7.74 7.73 7.73 7.65 7.64 7.48 7.47 7.33 7.33 7.31 7.30 7.26 CDCl3 7.01 7.00 4.49 1.86 1.86 1.83 1.82 1.82 1.81 1.78 1.78 1.77 1.76 1.75 1.75 1.74 1.73 1.72 1.71 1.69 1.68 1.66 1.65 1.48 1.47 1.46 1.46 1.45 1.44 1.42 1.41 1.41 1.40 1.40 1.39 1.38 1.37 1.35 1.34 1.33 1.33 1.32 1.32 1.30 0.92 0.90 0.88 0.86 0.85 0.84 0.83 0.82 0.81
S10
^{1}H NMR, $CDCl_3$
400 MHz
2.0 2.0 2.0 2.0 2.0 2.0 2.0 2.0 1.9 2.0 2.0 2.0 2.0 2.0 2.0 16.6 17.6 6.0 12.3
f1 (ppm)

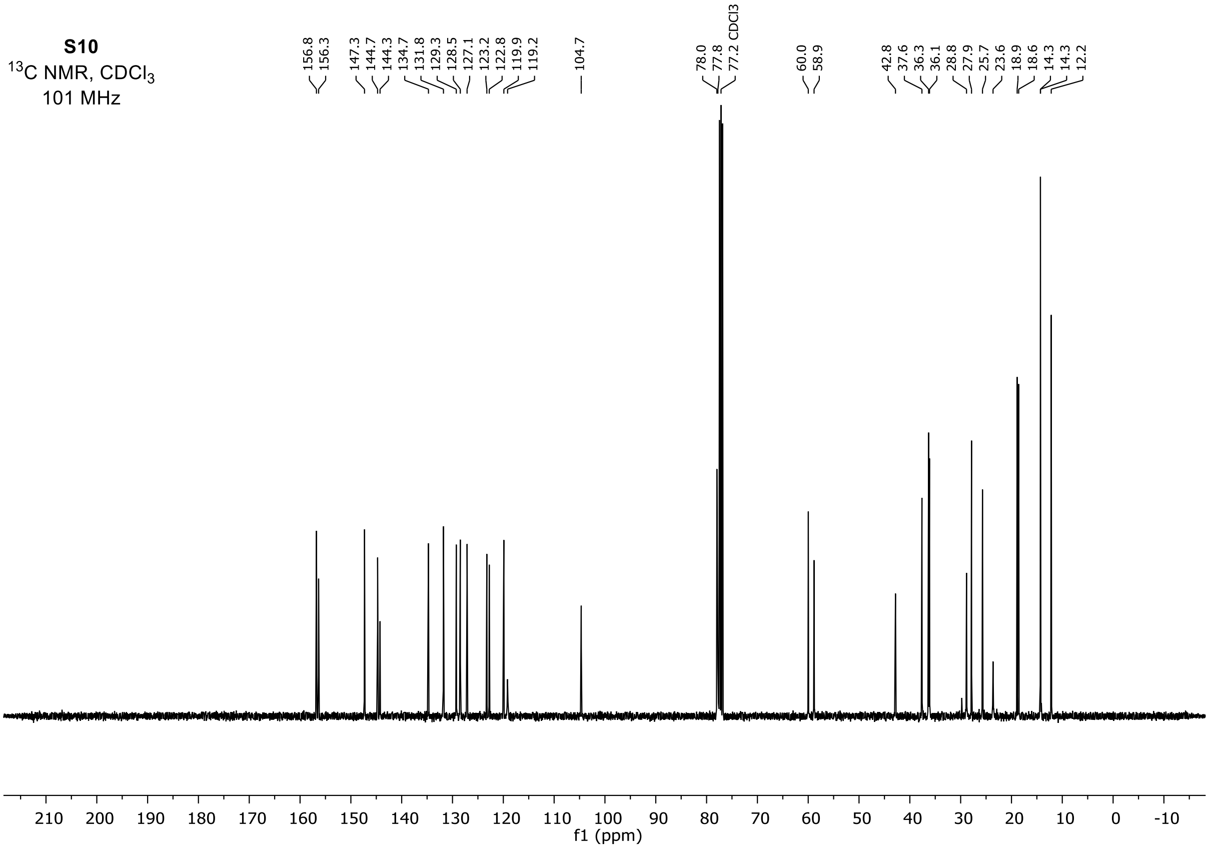
S10
^{13}C NMR, $CDCl_3$
101 MHz
156.8 156.3 147.3 144.7 144.3 134.7 131.8 129.3 128.5 127.1 123.2 122.8 119.9 119.2 104.7 78.0 77.8 77.2 CDCl3 60.0 58.9 42.8 37.6 36.3 36.1 28.8 27.9 25.7 23.6 18.9 18.6 14.3 14.3 12.2
f1 (ppm)

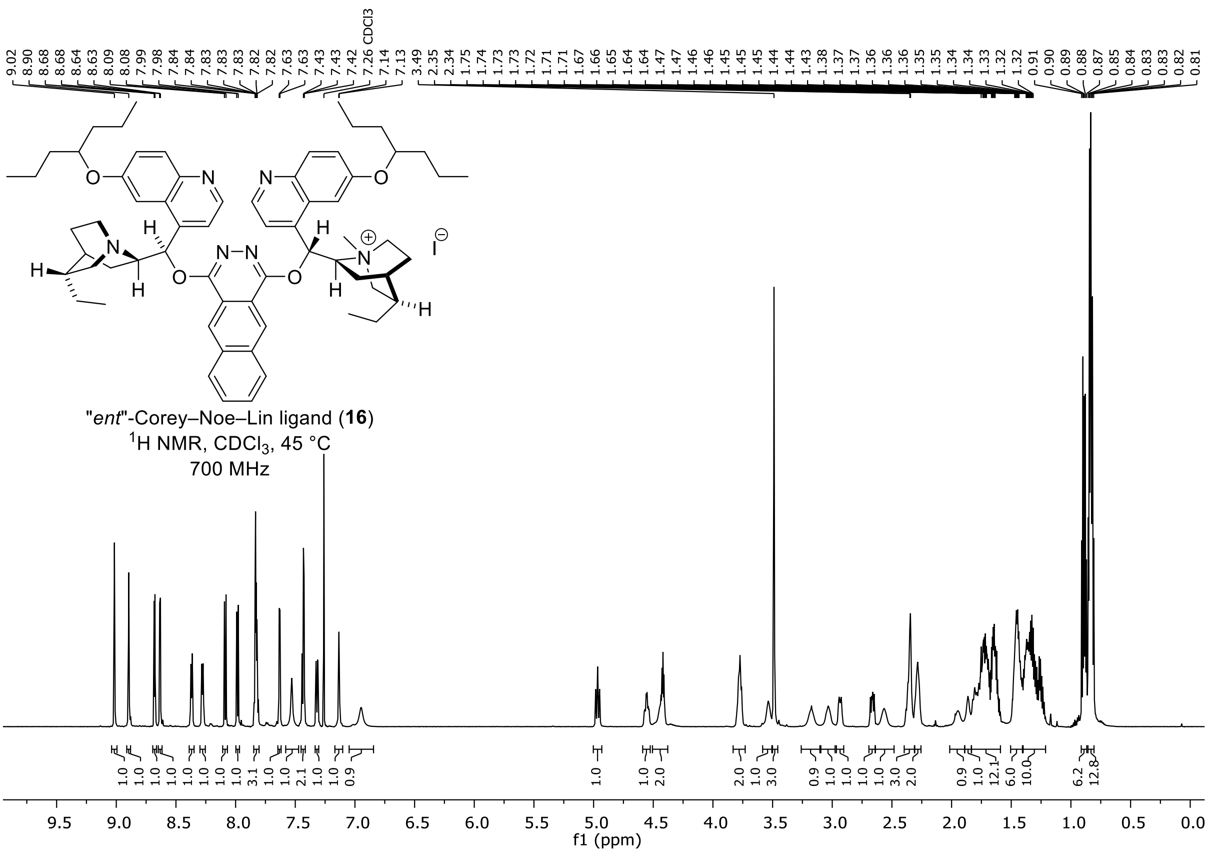
9.02 8.90 8.68 8.68 8.64 8.63 8.09 8.08 7.99 7.98 7.84 7.84 7.83 7.83 7.82 7.82 7.63 7.63 7.43 7.43 7.42 7.26 CDCl3 7.14 7.13 3.49 2.35 2.34 1.75 1.74 1.74 1.73 1.73 1.72 1.71 1.71 1.67 1.66 1.65 1.64 1.64 1.47 1.47 1.47 1.46 1.45 1.45 1.45 1.44 1.44 1.43 1.38 1.37 1.37 1.36 1.36 1.36 1.35 1.35 1.34 1.34 1.33 1.32 1.32 0.91 0.90 0.89 0.88 0.87 0.85 0.84 0.83 0.83 0.82 0.81
"*ent*"-Corey–Noe–Lin ligand (**16**)
^{1}H NMR, $CDCl_3$, 45 °C
700 MHz
f1 (ppm)

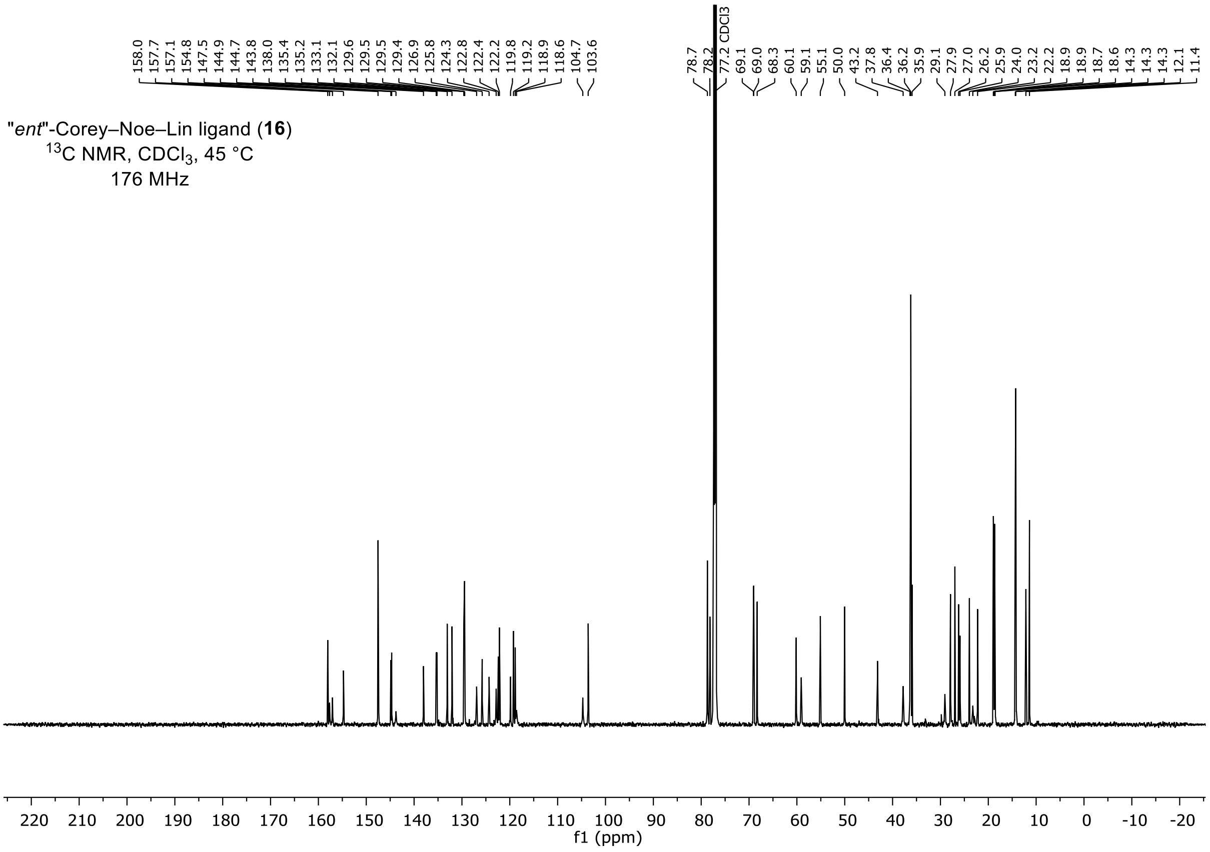
158.0 157.7 157.1 154.8 147.5 144.9 144.7 143.8 138.0 135.4 135.2 133.1 132.1 129.6 129.5 129.5 129.4 126.9 125.8 124.3 122.8 122.4 122.2 119.8 119.2 118.9 118.6 104.7 103.6 78.7 78.2 77.2 CDCl3 69.1 69.0 68.3 60.1 59.1 55.1 50.0 43.2 37.8 36.4 36.2 35.9 29.1 27.9 27.0 26.2 25.9 24.0 23.2 22.2 18.9 18.9 18.7 18.6 14.3 14.3 14.3 12.1 11.4
"*ent*"-Corey–Noe–Lin ligand (**16**)
^{13}C NMR, $CDCl_3$, 45 °C
176 MHz
f1 (ppm)

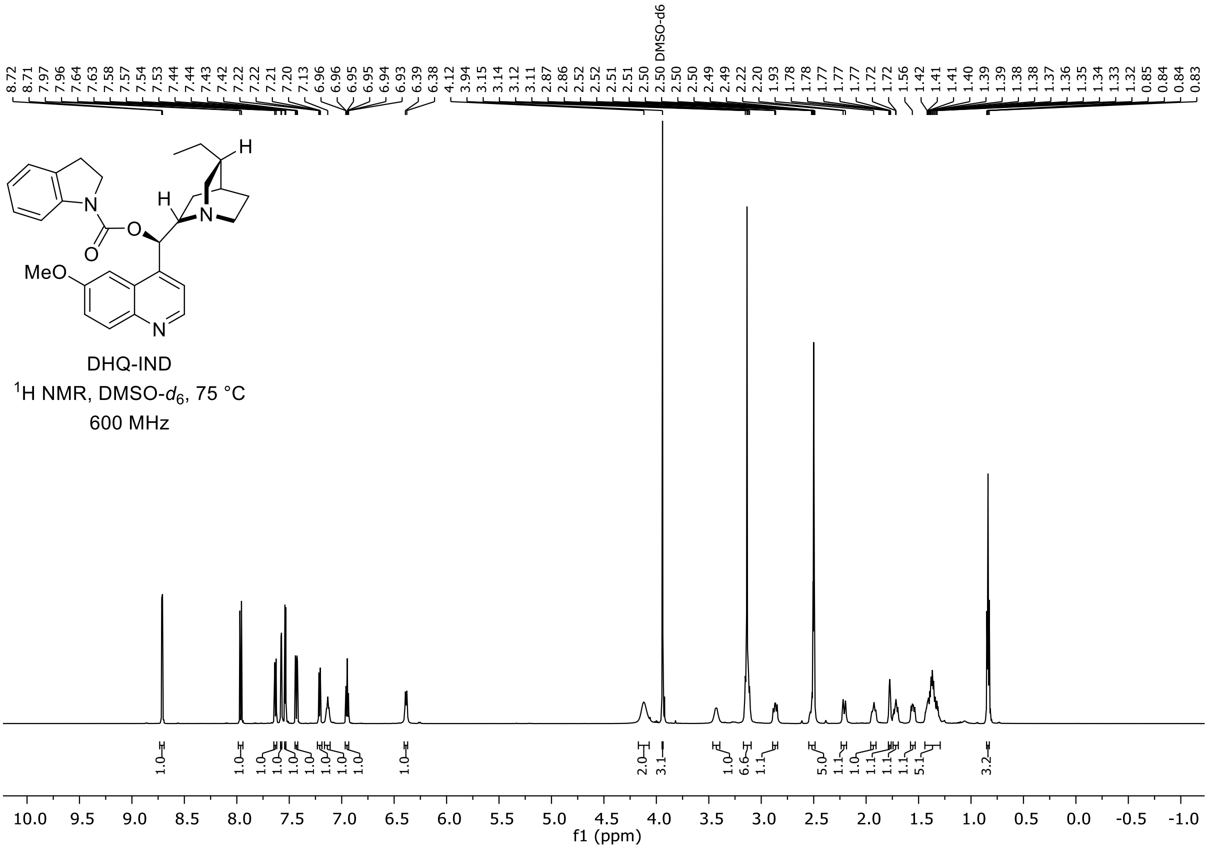
DHQ-IND
1H NMR, DMSO-d6, 75 °C
600 MHz
MeO
N
O
H
DMSO-d6
f1 (ppm)

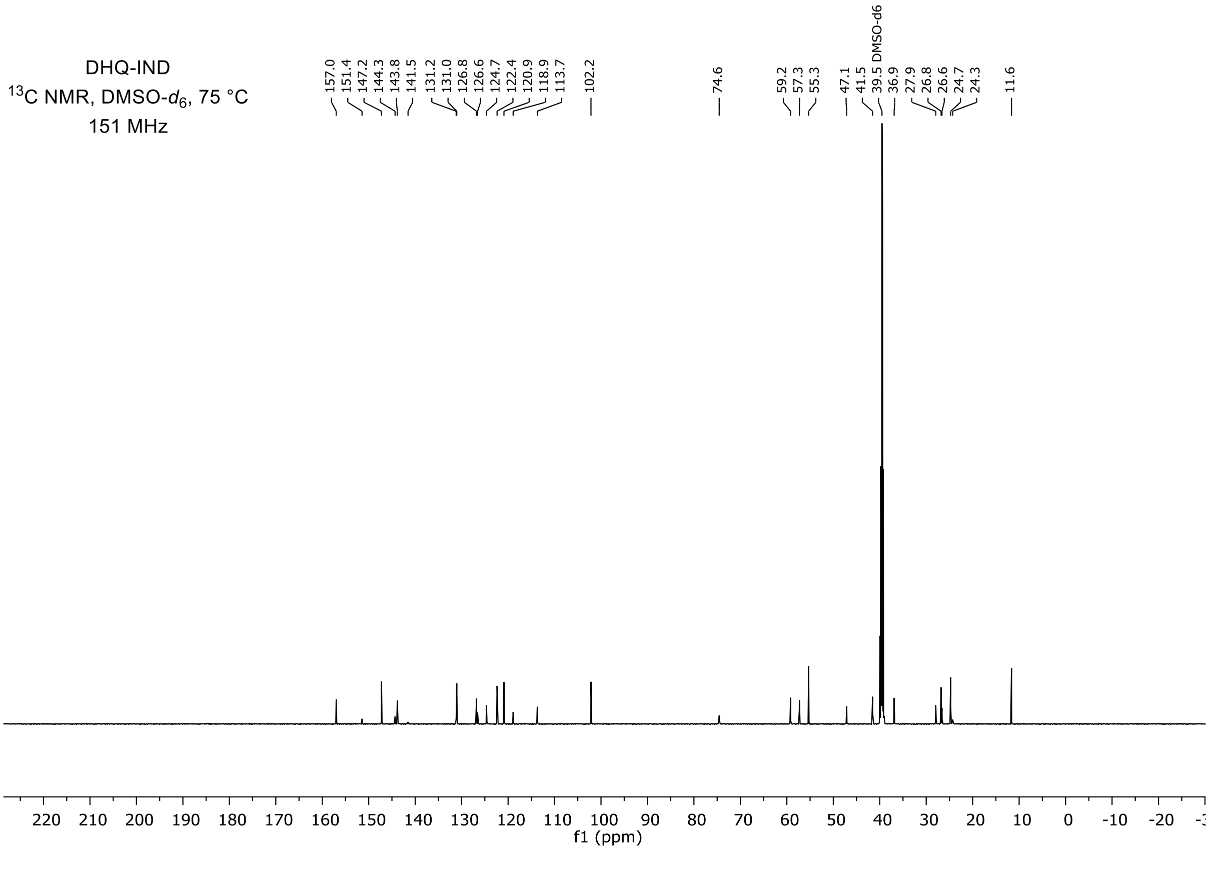
DHQ-IND
13C NMR, DMSO-d6, 75 °C
151 MHz
157.0
151.4
147.2
144.3
143.8
141.5
131.2
131.0
126.8
126.6
124.7
122.4
120.9
118.9
113.7
102.2
74.6
59.2
57.3
55.3
47.1
41.5
39.5 DMSO-d6
36.9
27.9
26.8
26.6
24.7
24.3
11.6
f1 (ppm)

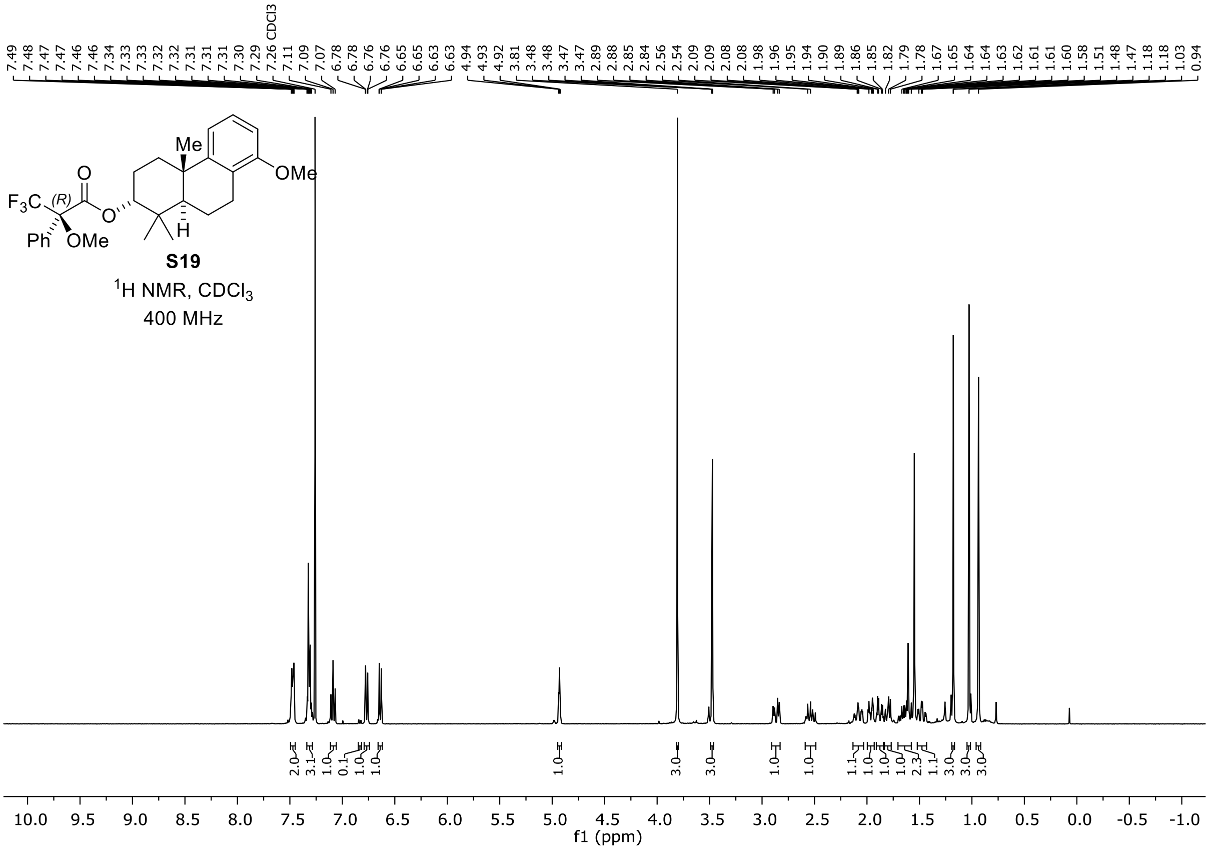
7.49 7.48 7.47 7.47 7.46 7.46 7.34 7.33 7.33 7.32 7.32 7.31 7.31 7.31 7.30 7.29 7.26 CDCl3 7.11 7.09 7.07 6.78 6.78 6.76 6.76 6.65 6.65 6.63 6.63 4.94 4.93 4.92 3.81 3.48 3.48 3.47 3.47 2.89 2.88 2.85 2.84 2.56 2.54 2.09 2.09 2.08 2.08 1.98 1.96 1.95 1.94 1.90 1.89 1.86 1.85 1.82 1.79 1.78 1.67 1.65 1.64 1.64 1.63 1.62 1.61 1.61 1.60 1.58 1.51 1.48 1.47 1.18 1.18 1.03 0.94
Me
OMe
O
F3C (R) O
Ph OMe
H
S19
1H NMR, CDCl3
400 MHz
2.0 3.1 1.0 0.1 1.0 1.0 1.0 3.0 3.0 1.0 1.0 1.1 1.0 1.0 1.0 2.3 1.1 3.0 3.0 3.0
10.0 9.5 9.0 8.5 8.0 7.5 7.0 6.5 6.0 5.5 5.0 4.5 4.0 3.5 3.0 2.5 2.0 1.5 1.0 0.5 0.0 -0.5 -1.0
f1 (ppm)

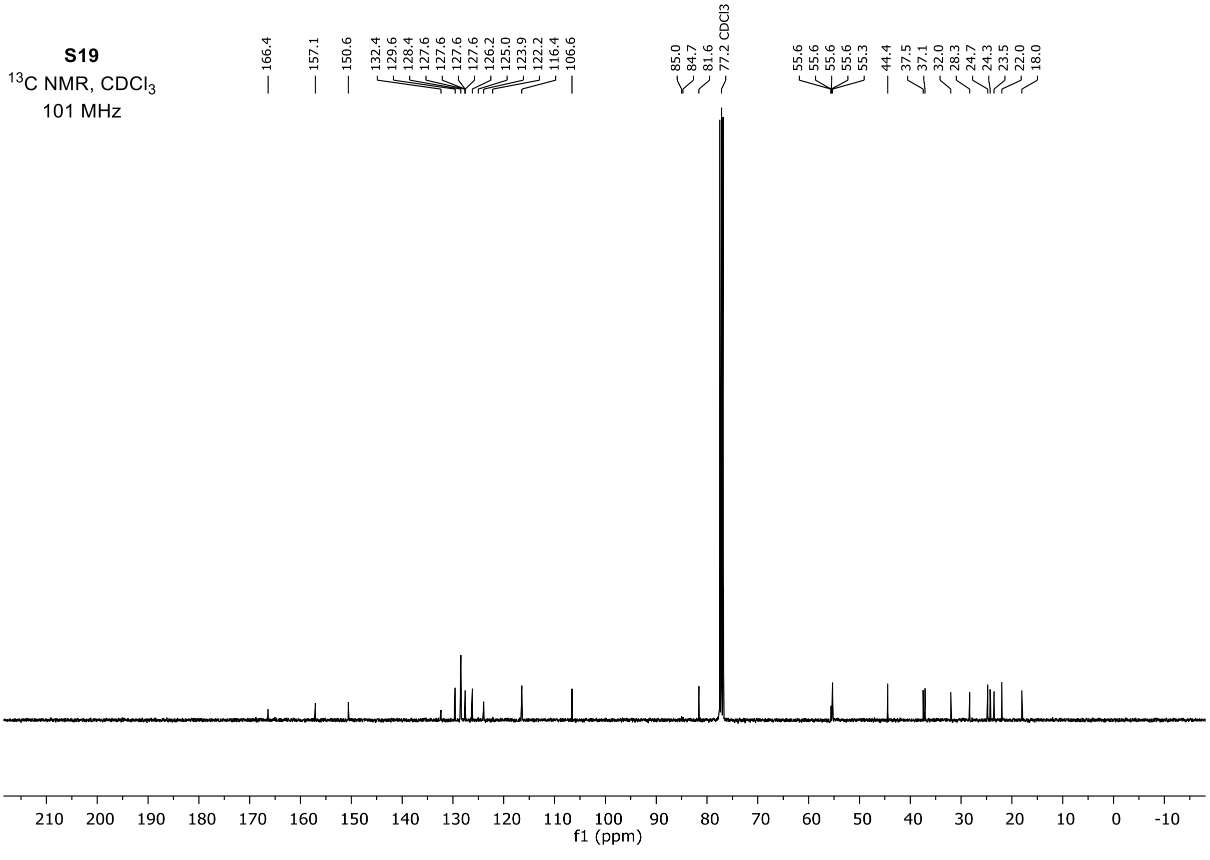
S19
13C NMR, CDCl3
101 MHz
166.4 157.1 150.6 132.4 129.6 128.4 127.6 127.6 127.6 127.6 126.2 125.0 123.9 122.2 116.4 106.6 85.0 84.7 81.6 77.2 CDCl3 55.6 55.6 55.6 55.6 55.3 44.4 37.5 37.1 32.0 28.3 24.7 24.3 23.5 22.0 18.0
210 200 190 180 170 160 150 140 130 120 110 100 90 80 70 60 50 40 30 20 10 0 -10
f1 (ppm)

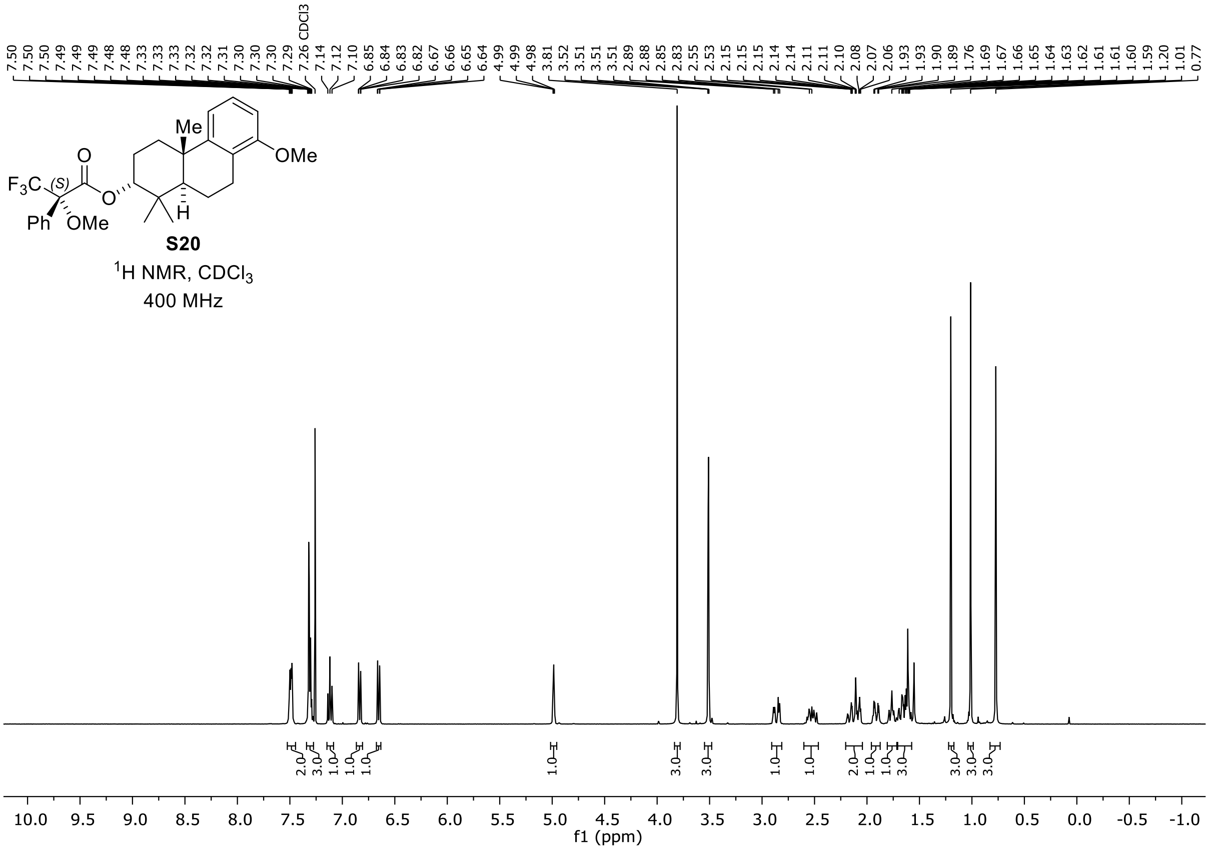
7.50 7.50 7.49 7.49 7.49 7.48 7.48 7.33 7.33 7.33 7.32 7.32 7.31 7.30 7.30 7.30 7.29 7.26 CDCl3 7.14 7.12 7.10 6.85 6.84 6.83 6.82 6.67 6.66 6.65 6.64 4.99 4.99 4.98 3.81 3.52 3.51 3.51 3.51 2.89 2.88 2.85 2.83 2.55 2.53 2.15 2.15 2.15 2.14 2.14 2.11 2.11 2.10 2.08 2.07 2.06 1.93 1.93 1.90 1.89 1.76 1.69 1.67 1.66 1.65 1.65 1.64 1.63 1.62 1.61 1.61 1.60 1.59 1.20 1.01 0.77
Me
OMe
O
F_3C
(S)
O
H
Ph
OMe
S20
^{1}H NMR, $CDCl_3$
400 MHz
2.0 3.0 1.0 1.0 1.0
1.0
3.0 3.0
1.0 1.0
2.0 1.0 1.0 1.0 3.0
3.0 3.0 3.0
10.0 9.5 9.0 8.5 8.0 7.5 7.0 6.5 6.0 5.5 5.0 4.5 4.0 3.5 3.0 2.5 2.0 1.5 1.0 0.5 0.0 -0.5 -1.0
f1 (ppm)

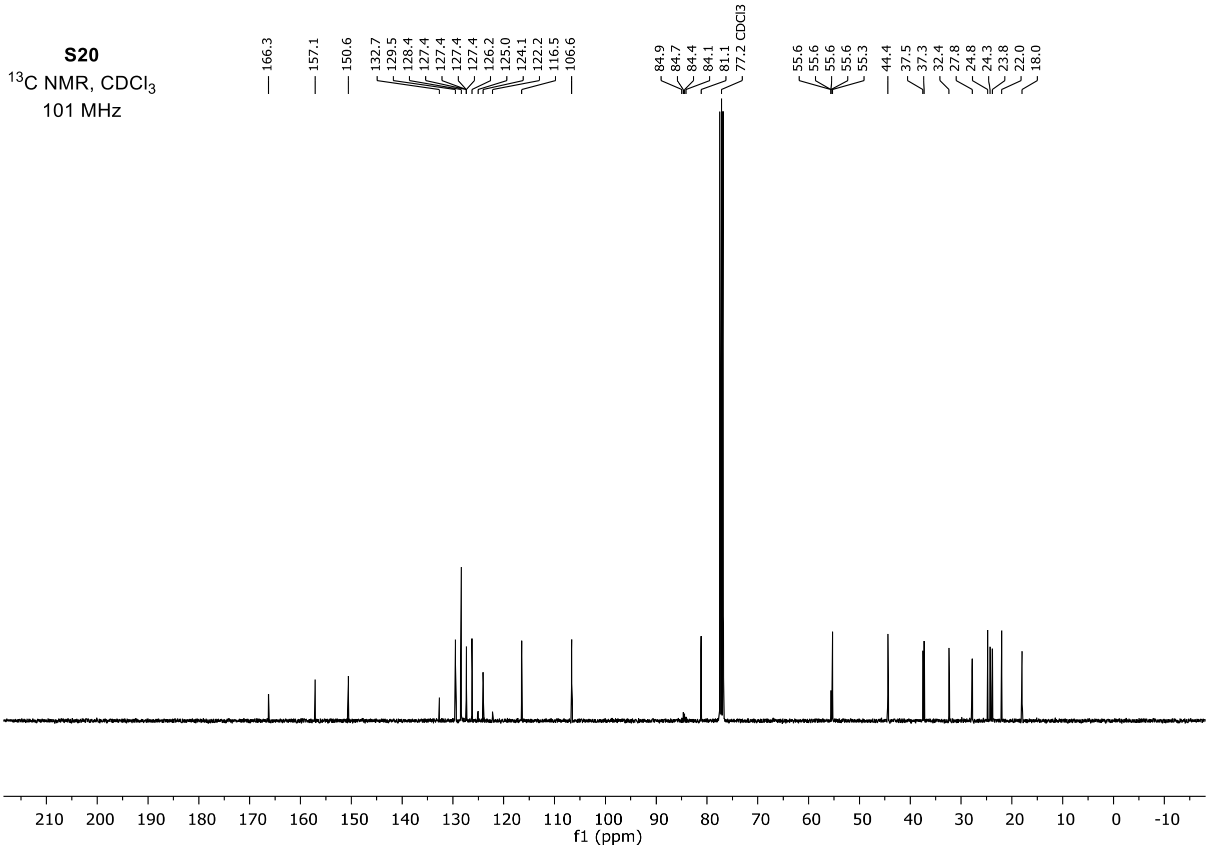
S20
^{13}C NMR, $CDCl_3$
101 MHz
166.3 157.1 150.6 132.7 129.5 128.4 127.4 127.4 127.4 127.4 126.2 125.0 124.1 122.2 116.5 106.6 84.9 84.7 84.4 84.1 81.1 77.2 CDCl3 55.6 55.6 55.6 55.6 55.3 44.4 37.5 37.3 32.4 27.8 24.8 24.3 23.8 22.0 18.0
210 200 190 180 170 160 150 140 130 120 110 100 90 80 70 60 50 40 30 20 10 0 -10
f1 (ppm)

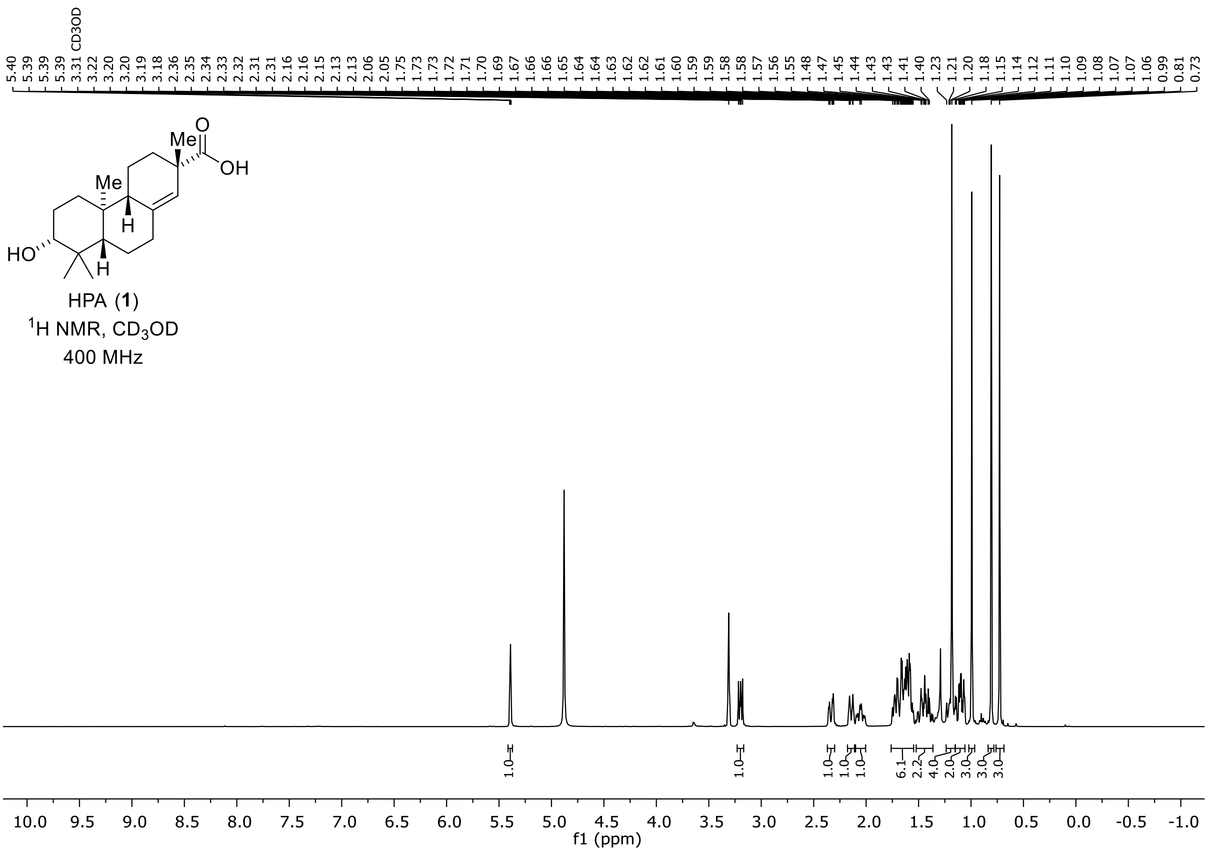

5.40 5.39 5.39 5.39 3.31 CD3OD 3.22 3.20 3.20 3.19 3.18 2.36 2.35 2.34 2.33 2.32 2.31 2.31 2.16 2.16 2.15 2.13 2.13 2.06 2.05 1.75 1.73 1.73 1.72 1.71 1.70 1.69 1.67 1.66 1.66 1.65 1.64 1.64 1.63 1.62 1.62 1.61 1.60 1.59 1.59 1.58 1.58 1.57 1.56 1.55 1.48 1.47 1.45 1.44 1.43 1.43 1.41 1.40 1.23 1.21 1.20 1.18 1.15 1.14 1.12 1.11 1.10 1.09 1.08 1.07 1.07 1.06 0.99 0.81 0.73
Me
O
OH
Me
H
HO
H
HPA (1)
^{1}H NMR, CD$_3$OD
400 MHz
1.0
1.0
1.0
1.0
1.0
6.1
2.2
4.0
2.0
3.0
3.0
3.0
10.0 9.5 9.0 8.5 8.0 7.5 7.0 6.5 6.0 5.5 5.0 4.5 4.0 3.5 3.0 2.5 2.0 1.5 1.0 0.5 0.0 -0.5 -1.0
f1 (ppm)

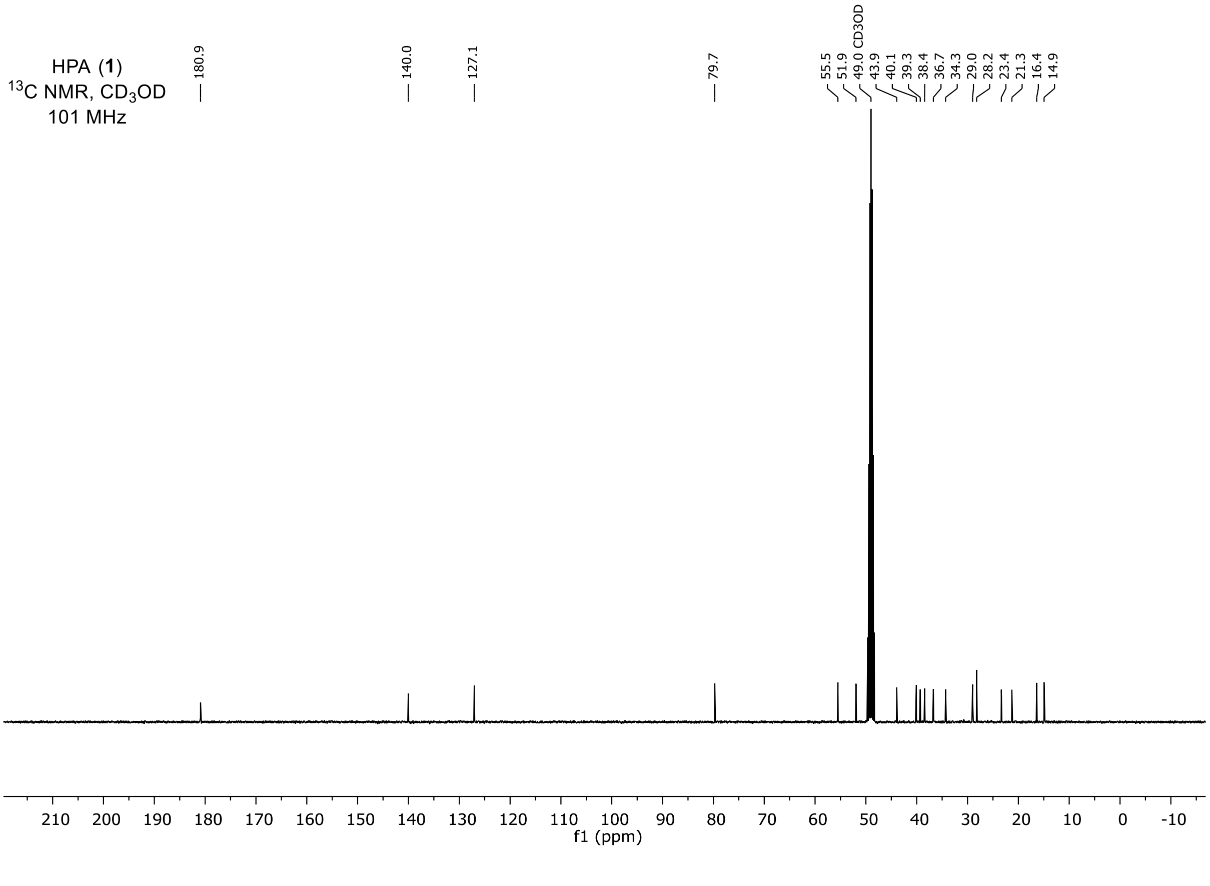

HPA (1)
^{13}C NMR, CD$_3$OD
101 MHz
180.9
140.0
127.1
79.7
55.5 51.9 49.0 CD3OD 43.9 40.1 39.3 38.4 36.7 34.3 29.0 28.2 23.4 21.3 16.4 14.9
210 200 190 180 170 160 150 140 130 120 110 100 90 80 70 60 50 40 30 20 10 0 -10
f1 (ppm)

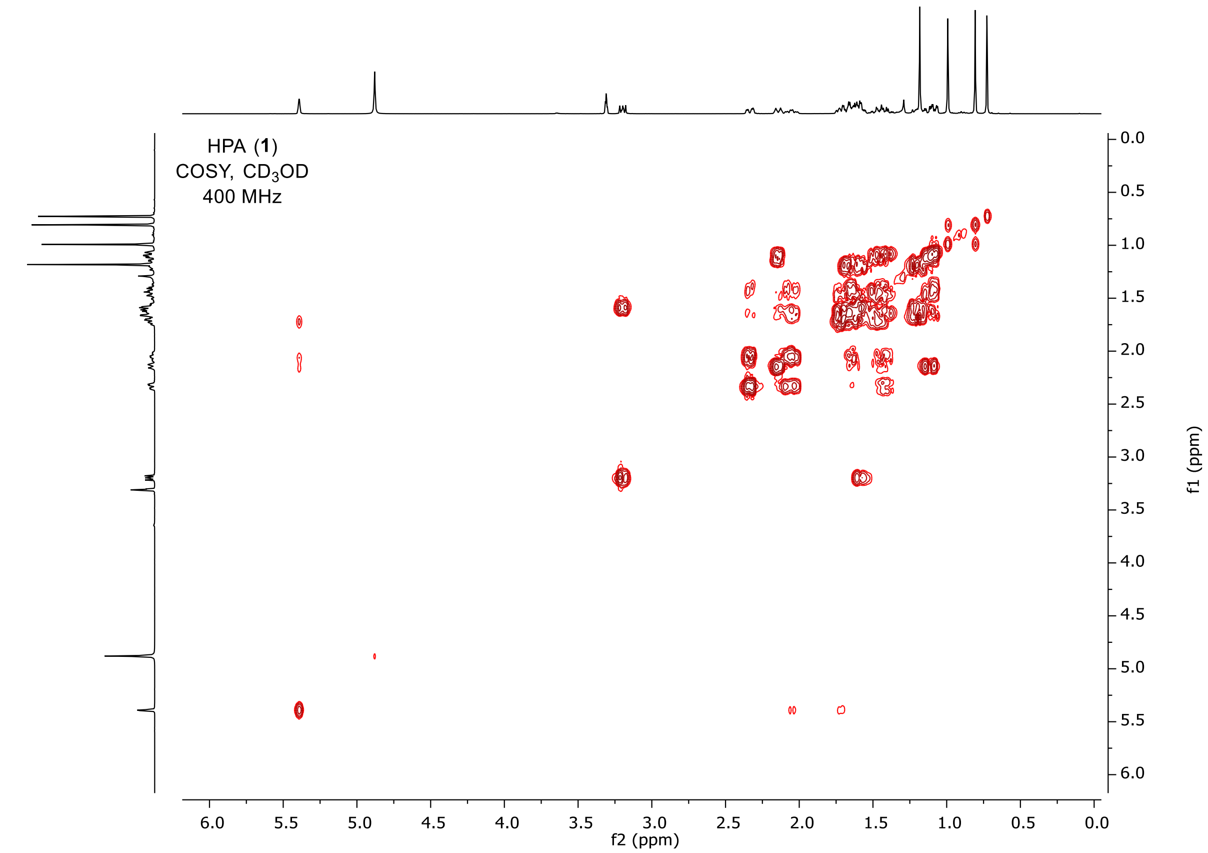
HPA (**1**)
COSY, CD3OD
400 MHz
f1 (ppm)
f2 (ppm)

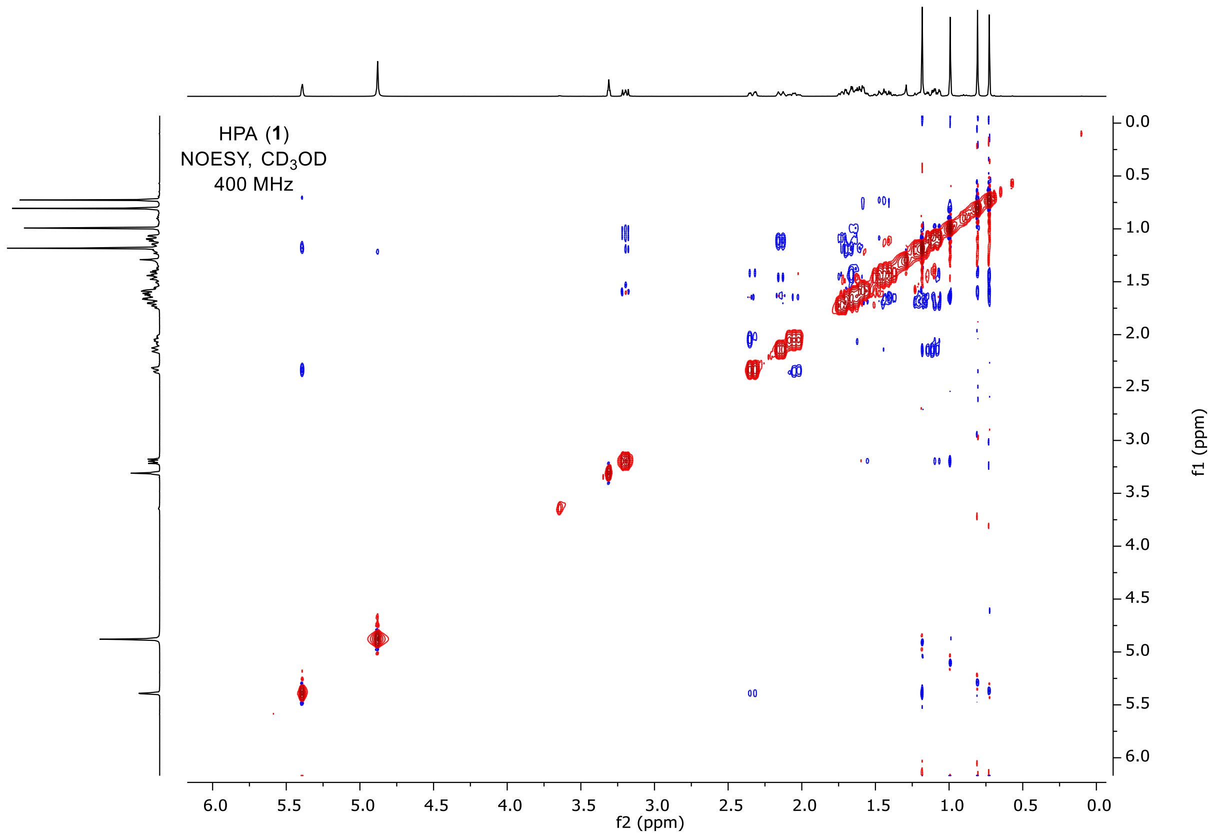
HPA (**1**)
NOESY, CD3OD
400 MHz
f1 (ppm)
f2 (ppm)

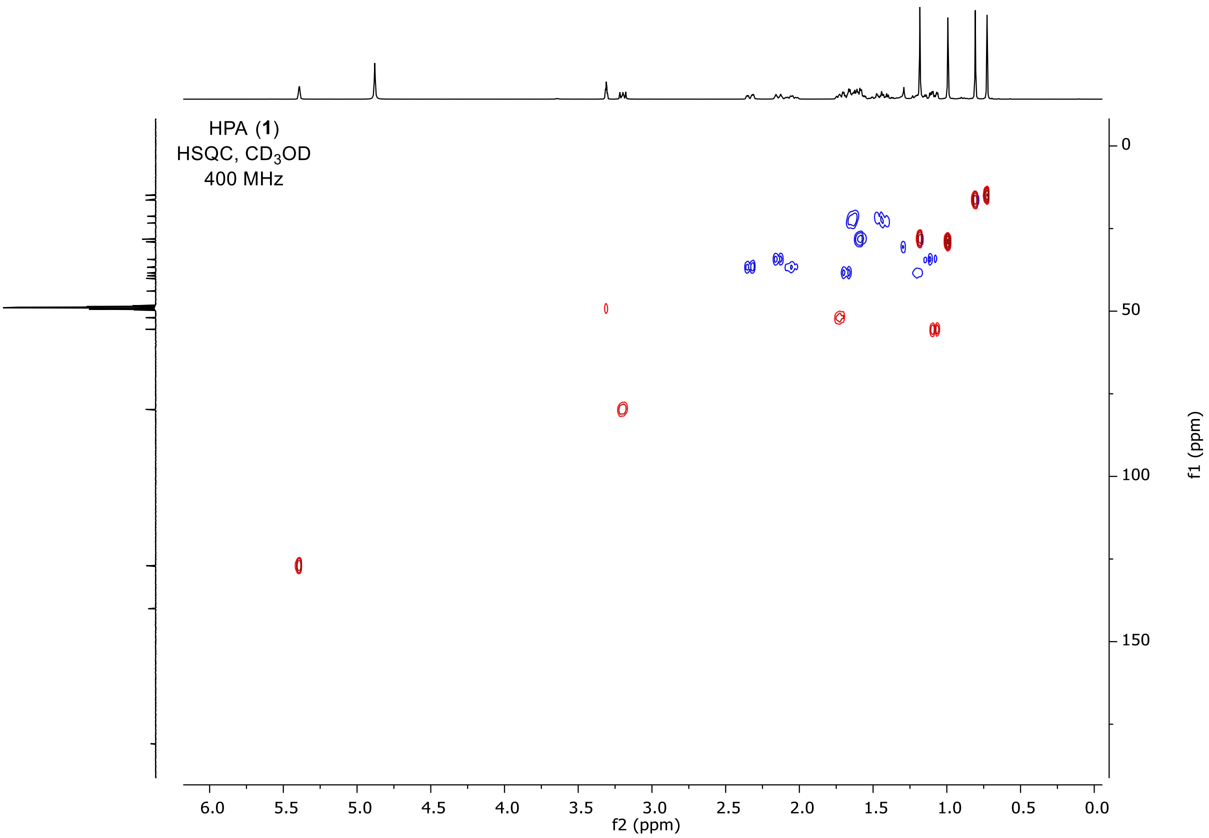
HPA (**1**)
HSQC, CD3OD
400 MHz
f2 (ppm)
f1 (ppm)

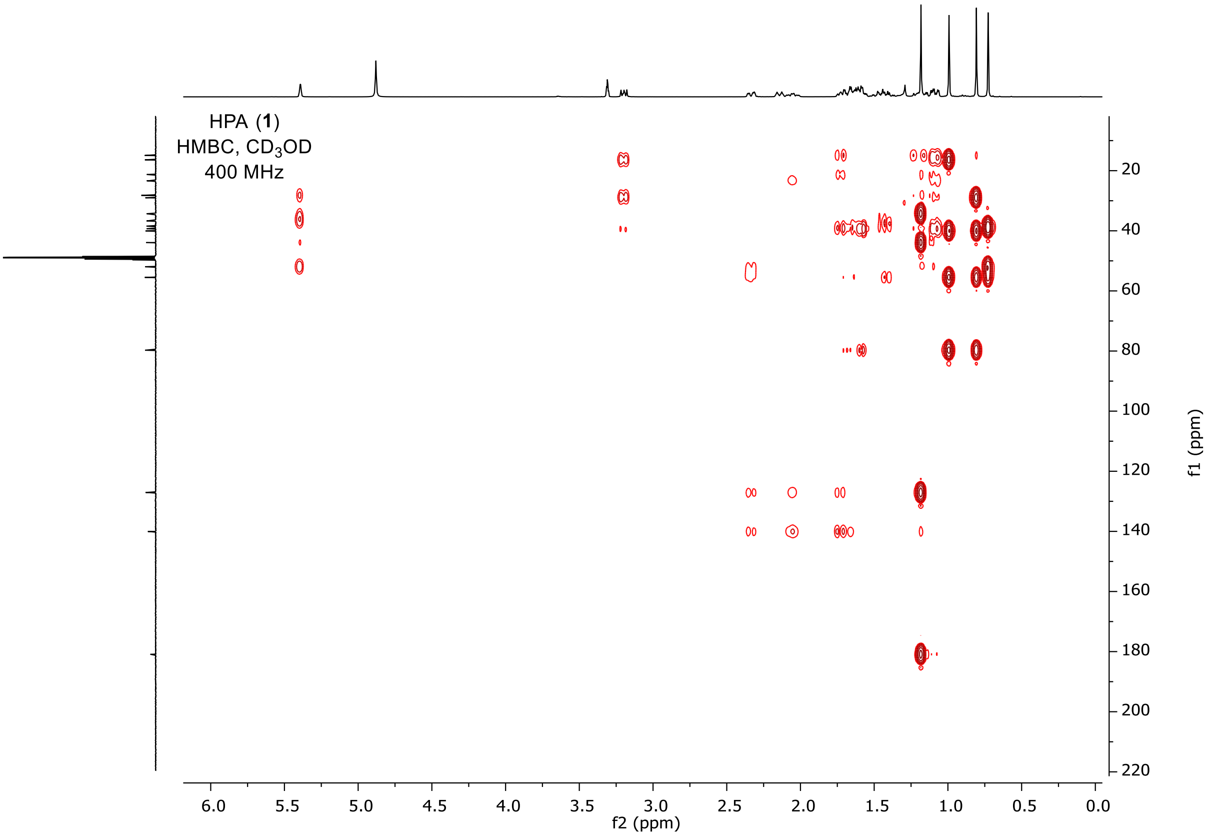
HPA (**1**)
HMBC, CD3OD
400 MHz
f2 (ppm)
f1 (ppm)

norflickinflimiod C (**5**)
^{1}H NMR, CD_3OD
400 MHz

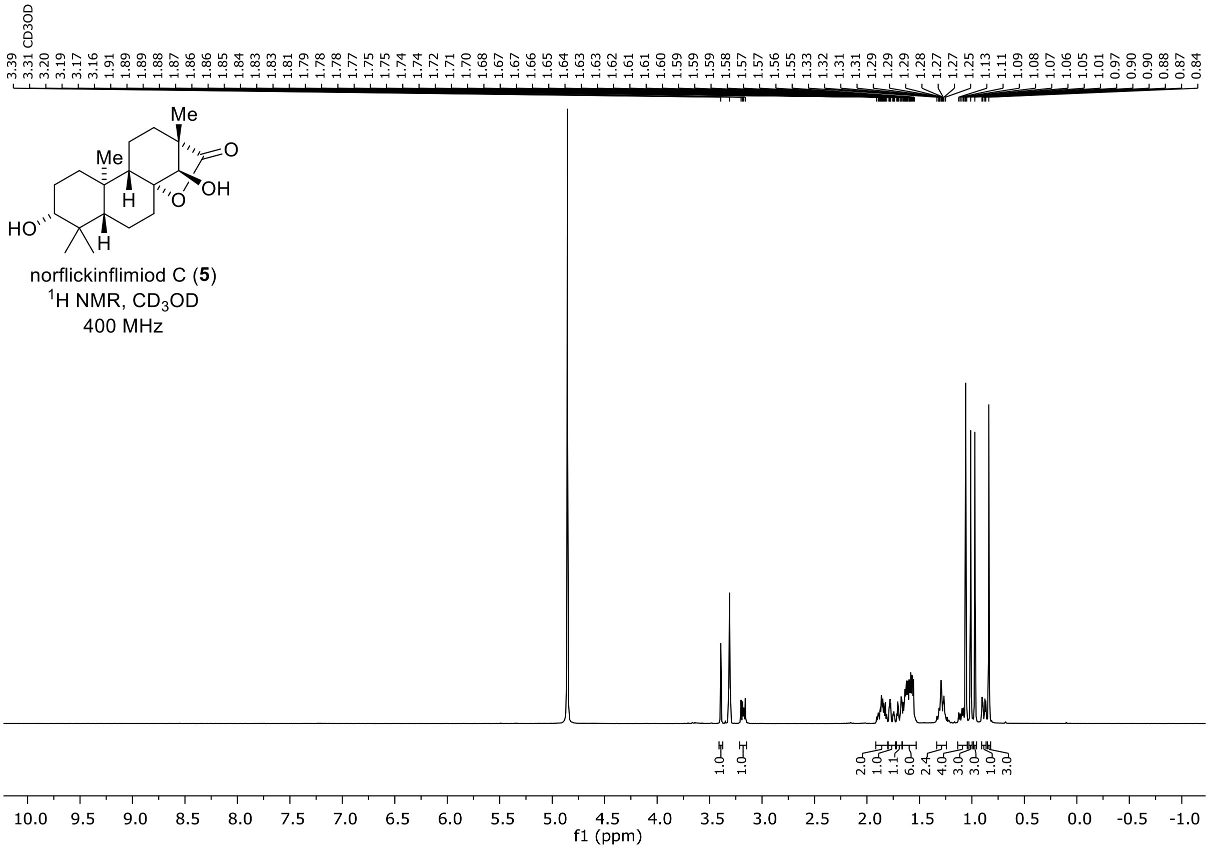

norflickinflimiod C (**5**)
^{13}C NMR, CD_3OD
101 MHz

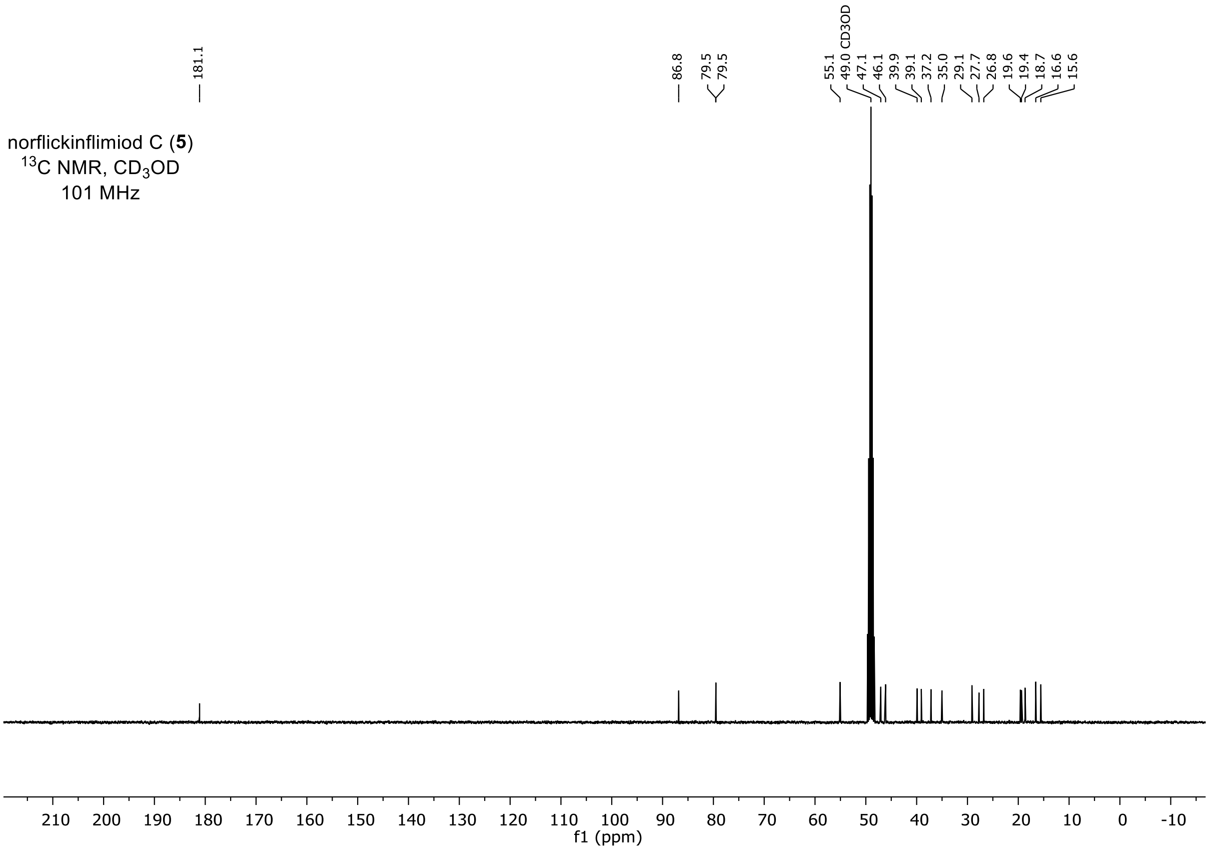

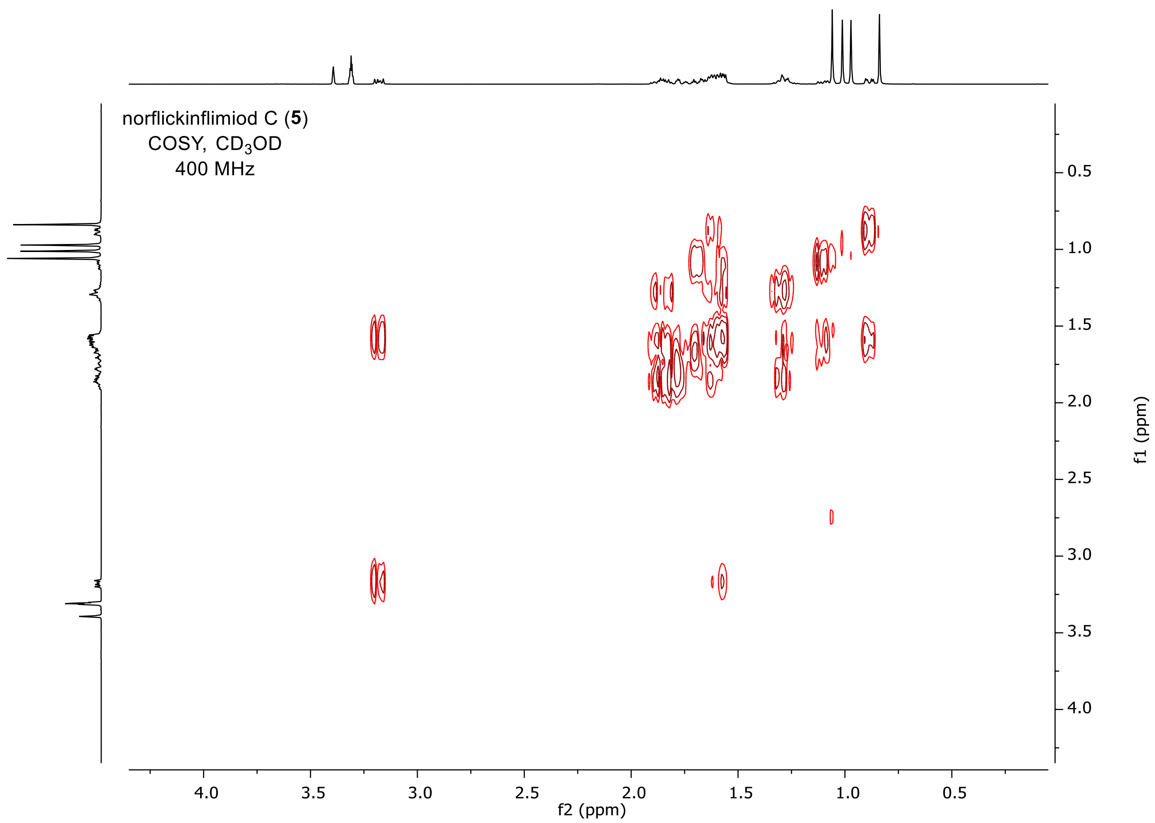
norflickinflimiod C (**5**)
COSY, CD_3OD
400 MHz
f1 (ppm)
f2 (ppm)

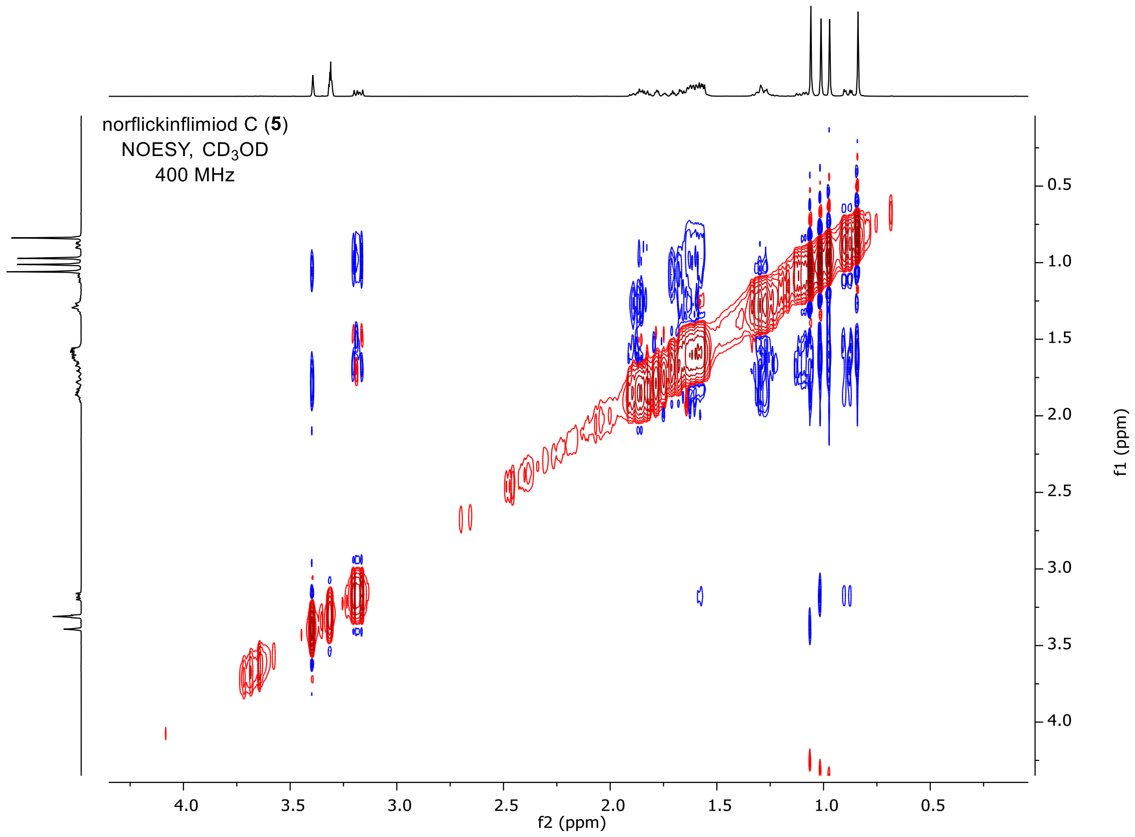
norflickinflimiod C (**5**)
NOESY, CD_3OD
400 MHz
f1 (ppm)
f2 (ppm)

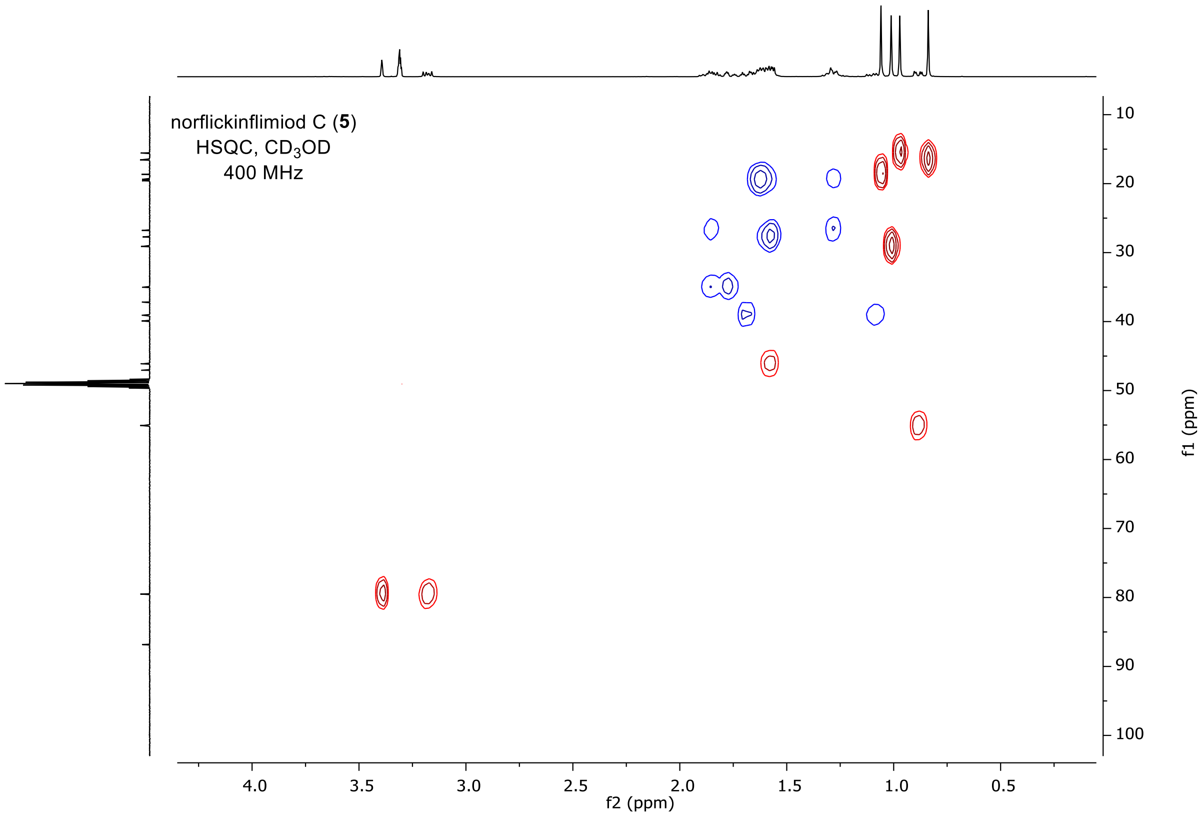
norflickinflimiod C (5)
HSQC, CD3OD
400 MHz
f1 (ppm)
f2 (ppm)

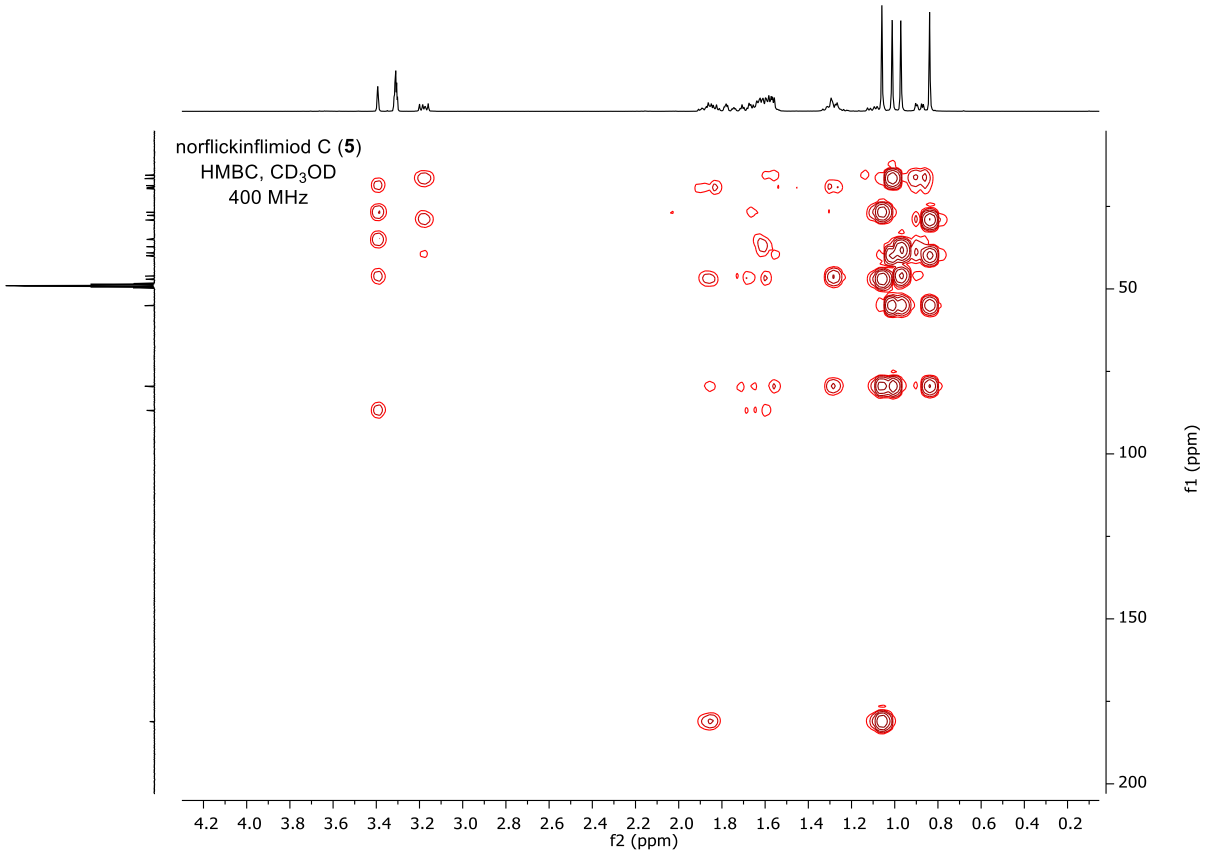
norflickinflimiod C (5)
HMBC, CD3OD
400 MHz
f1 (ppm)
f2 (ppm)

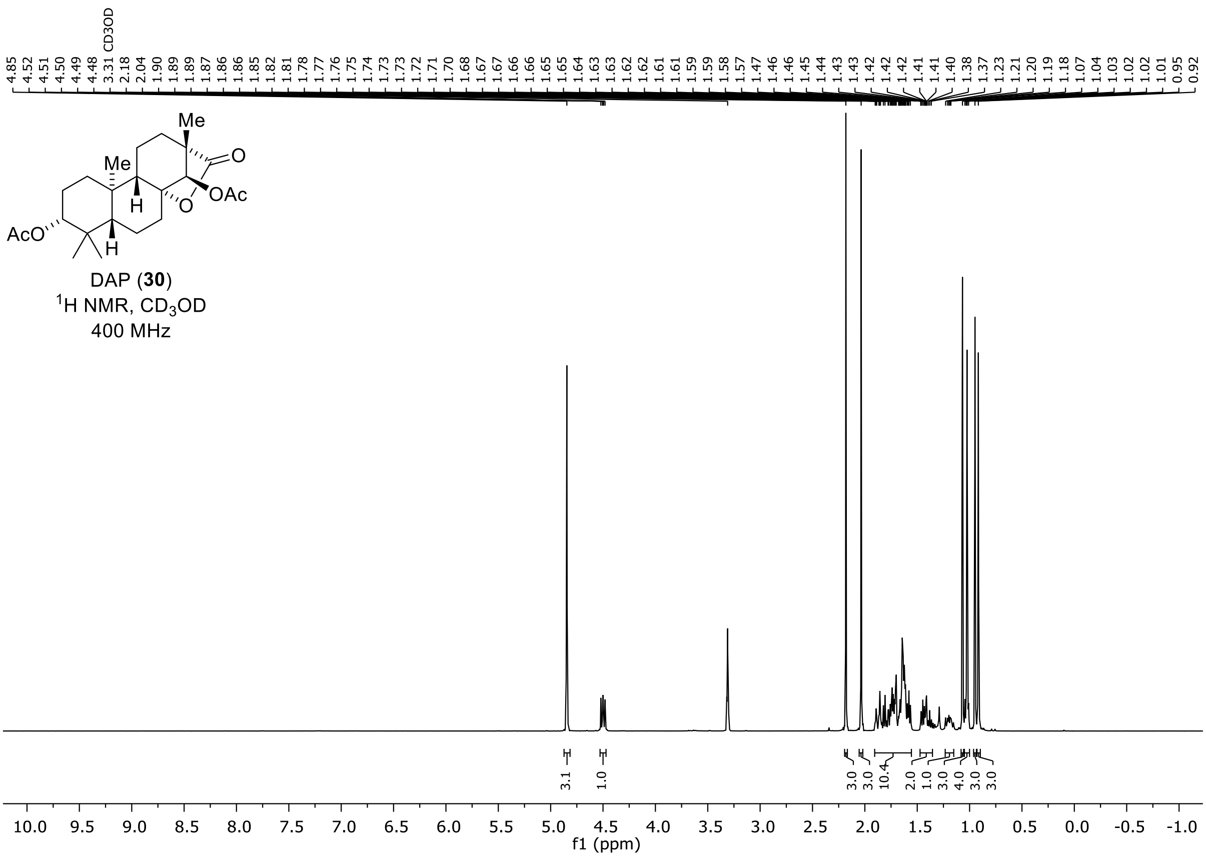

DAP (**30**)
^{1}H NMR, CD_3OD
400 MHz

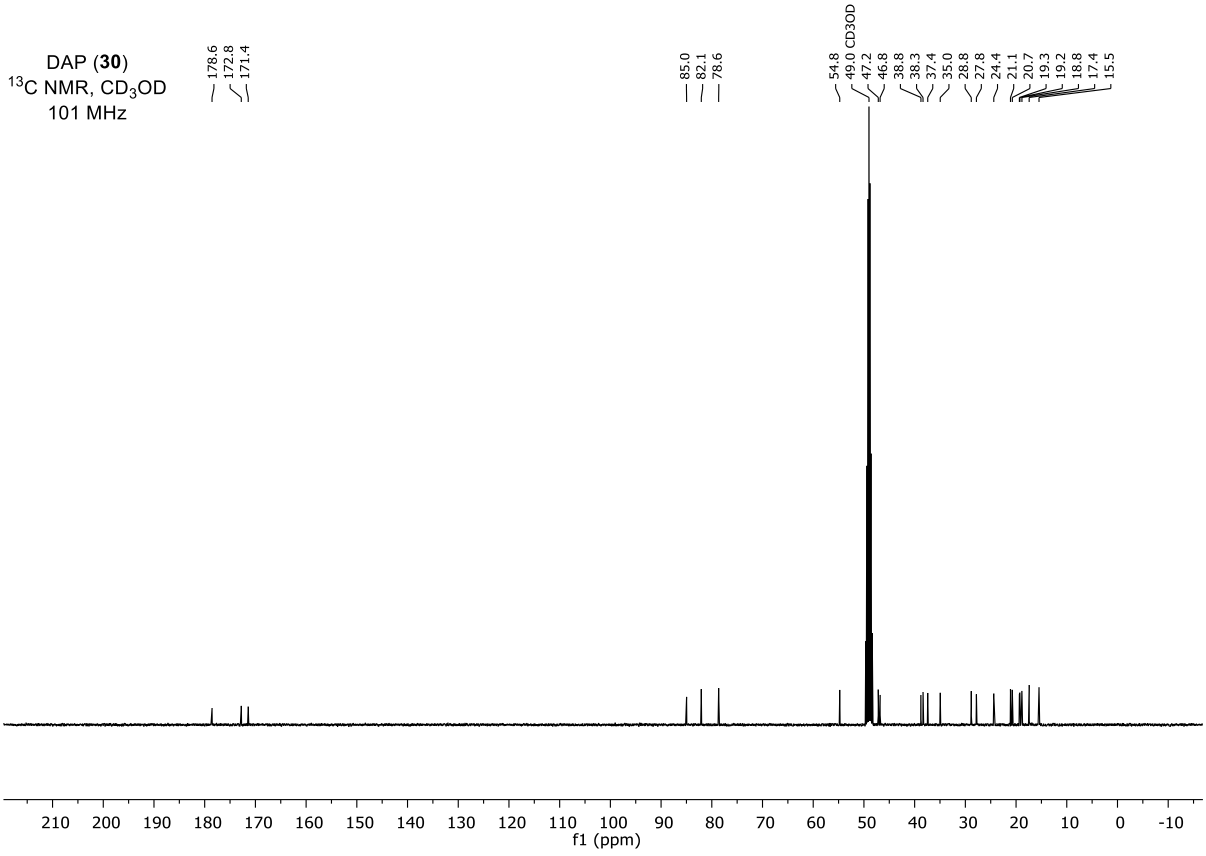

DAP (**30**)
^{13}C NMR, CD_3OD
101 MHz

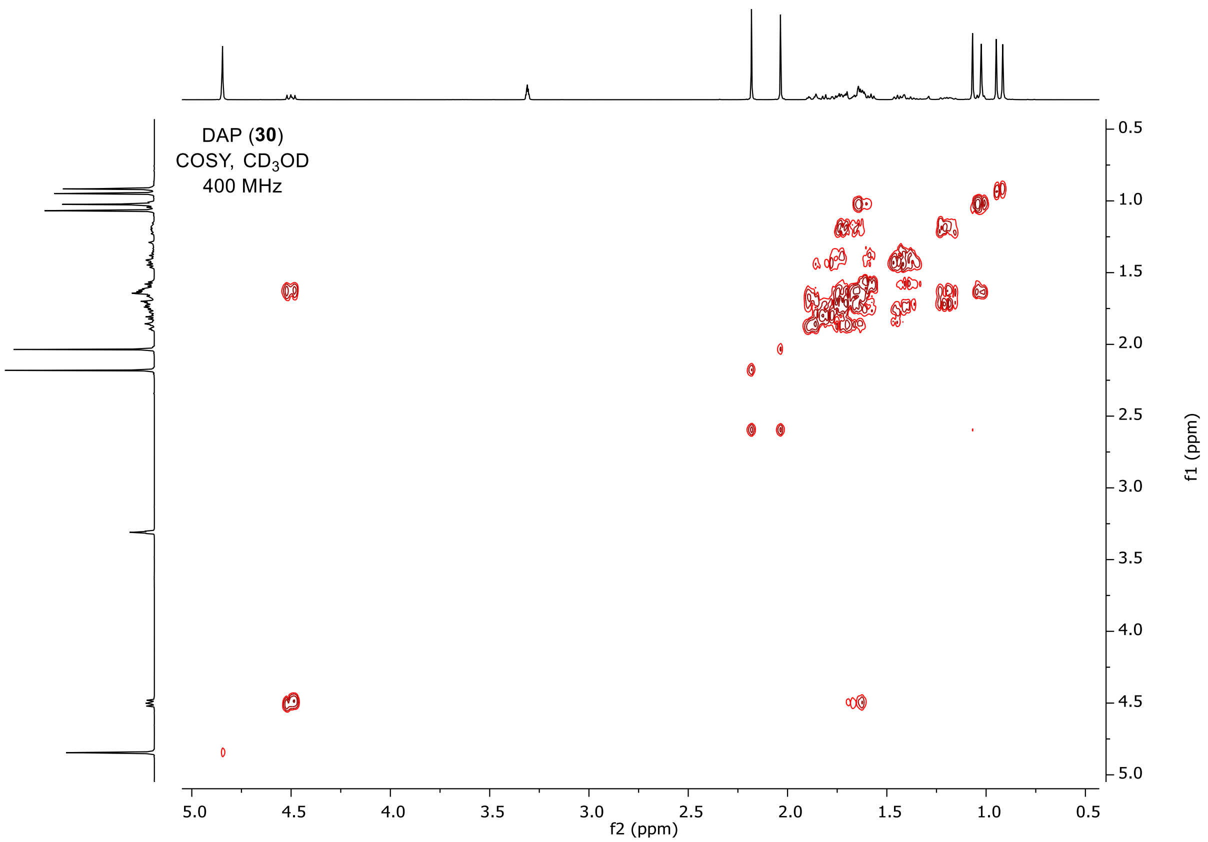
DAP (**30**)
COSY, CD_3OD
400 MHz
f2 (ppm)
f1 (ppm)

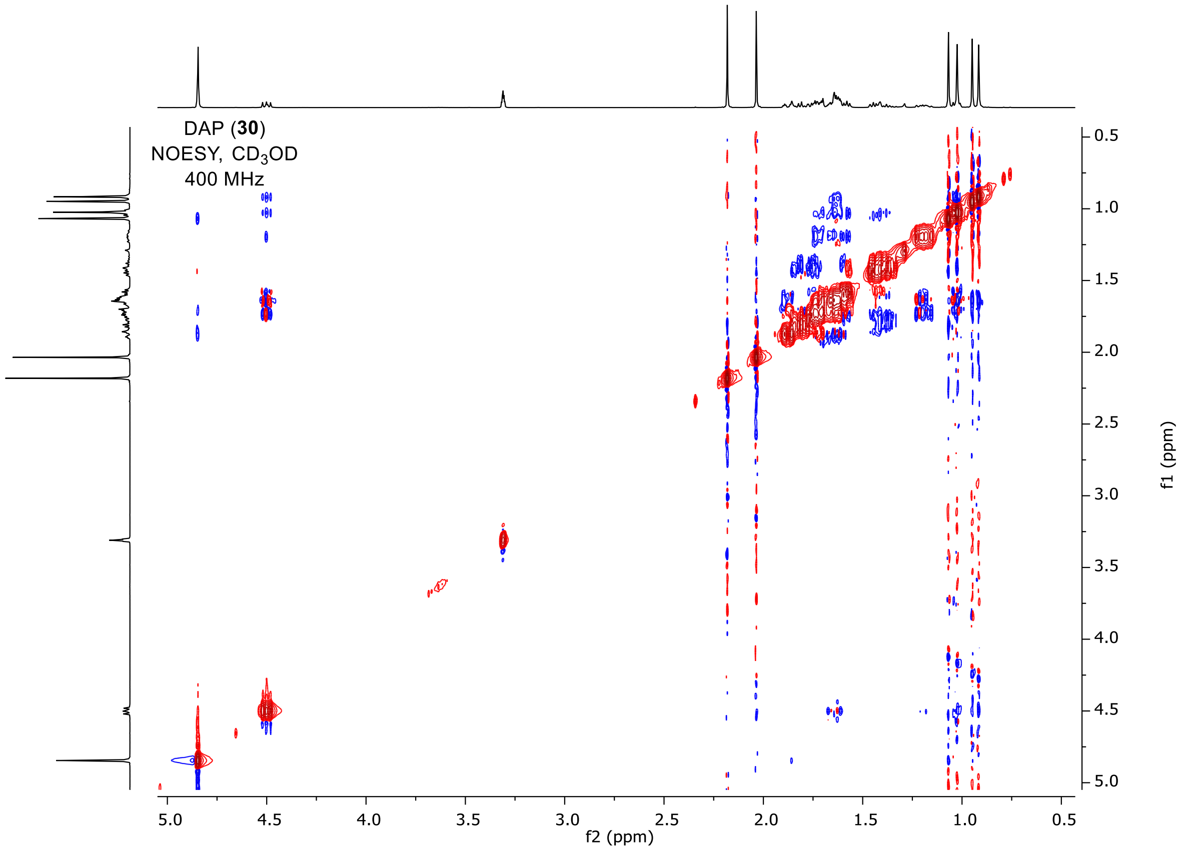
DAP (**30**)
NOESY, CD_3OD
400 MHz
f2 (ppm)
f1 (ppm)

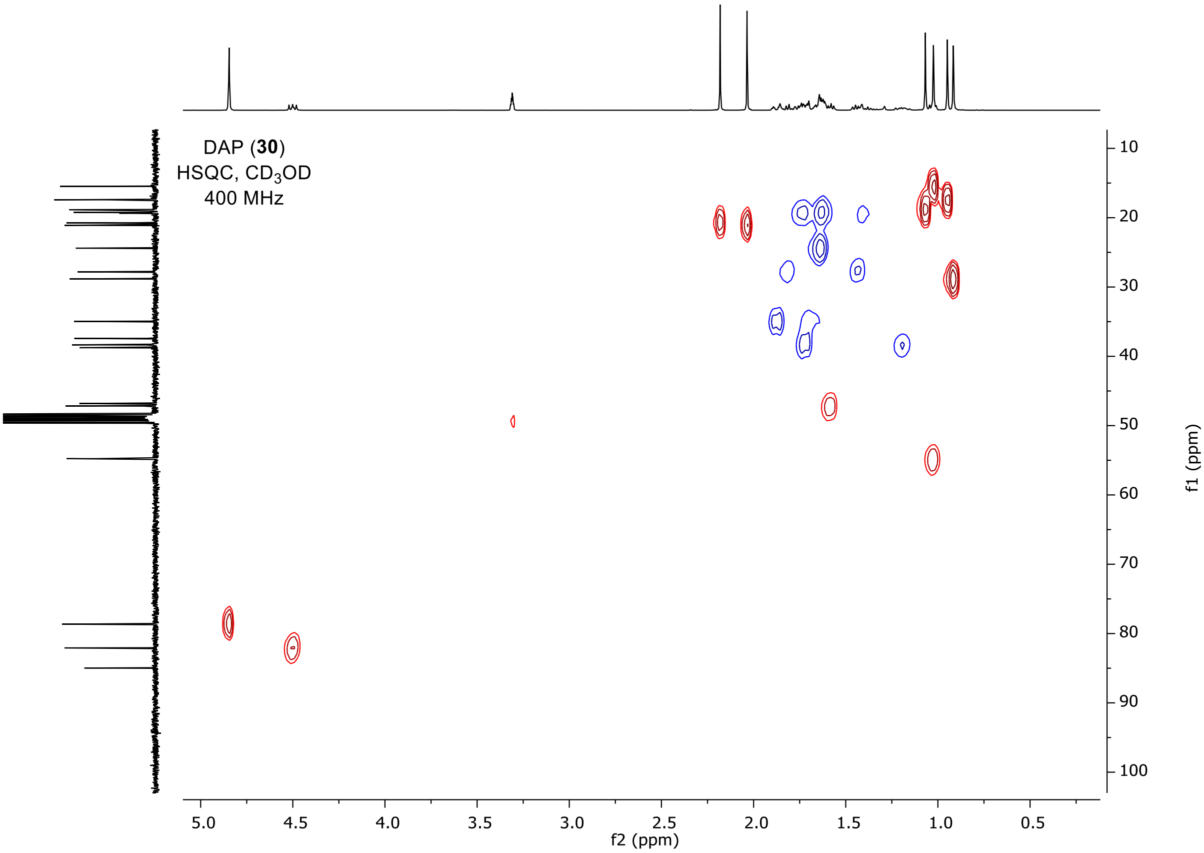
DAP (30)
HSQC, CD3OD
400 MHz
f2 (ppm)
f1 (ppm)

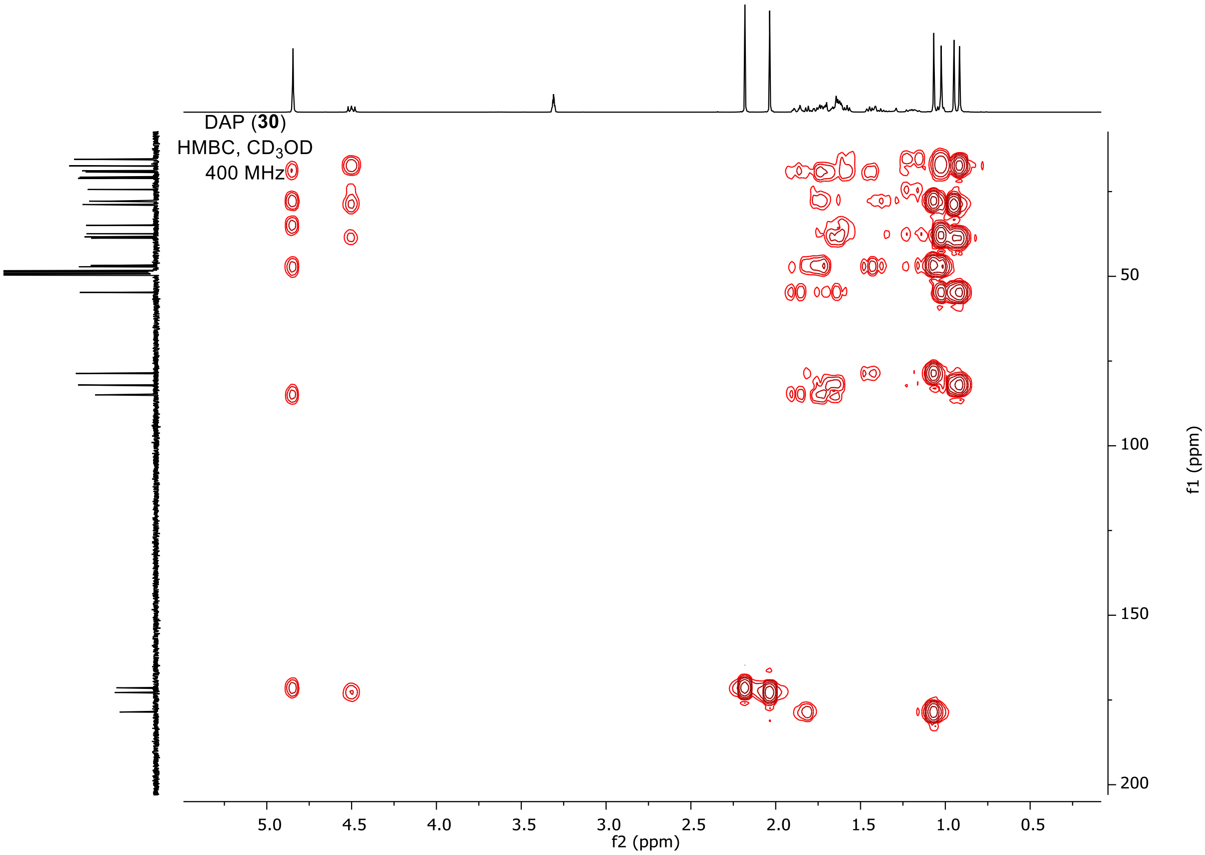
DAP (30)
HMBC, CD3OD
400 MHz
f2 (ppm)
f1 (ppm)

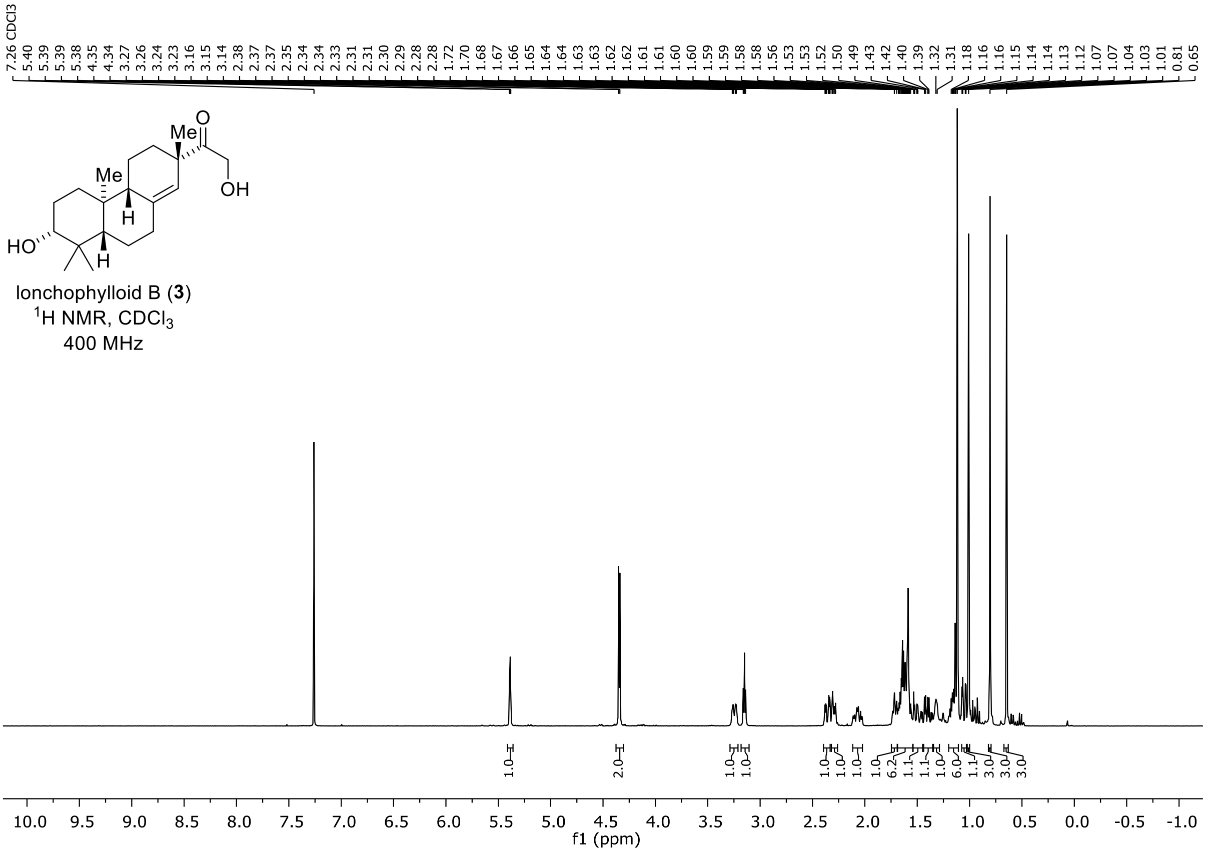
7.26 CDCl3
5.40 5.39 5.39 5.38 4.35 4.34 3.27 3.26 3.24 3.23 3.16 3.15 3.14 2.38 2.37 2.37 2.35 2.34 2.34 2.33 2.31 2.31 2.30 2.29 2.28 2.28 1.72 1.70 1.68 1.67 1.66 1.65 1.64 1.64 1.63 1.63 1.62 1.62 1.61 1.61 1.60 1.60 1.59 1.59 1.58 1.58 1.56 1.53 1.52 1.50 1.49 1.43 1.42 1.40 1.39 1.32 1.31 1.18 1.16 1.16 1.15 1.14 1.14 1.13 1.12 1.07 1.07 1.04 1.03 1.01 0.81 0.65
Me
O
OH
Me
H
HO
H
Ionchophylloid B (3)
^{1}H NMR, CDCl$_3$
400 MHz
1.0 2.0 1.0 1.0 1.0 1.0 1.0 1.0 6.2 1.1 1.1 1.0 6.0 1.1 3.0 3.0 3.0
10.0 9.5 9.0 8.5 8.0 7.5 7.0 6.5 6.0 5.5 5.0 4.5 4.0 3.5 3.0 2.5 2.0 1.5 1.0 0.5 0.0 -0.5 -1.0
f1 (ppm)

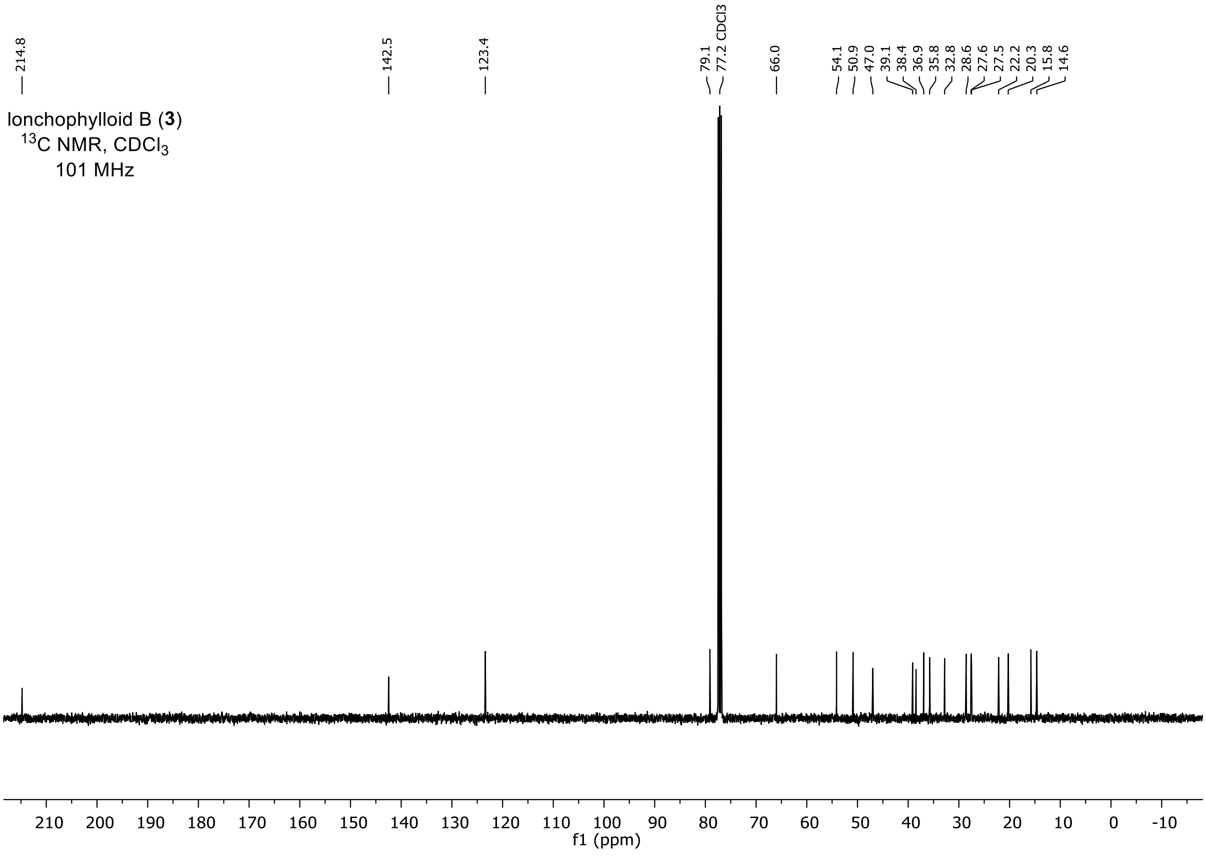
214.8
142.5
123.4
79.1
77.2 CDCl3
66.0
54.1 50.9 47.0 39.1 38.4 36.9 35.8 32.8 28.6 27.6 27.5 22.2 20.3 15.8 14.6
Ionchophylloid B (3)
^{13}C NMR, CDCl$_3$
101 MHz
210 200 190 180 170 160 150 140 130 120 110 100 90 80 70 60 50 40 30 20 10 0 -10
f1 (ppm)

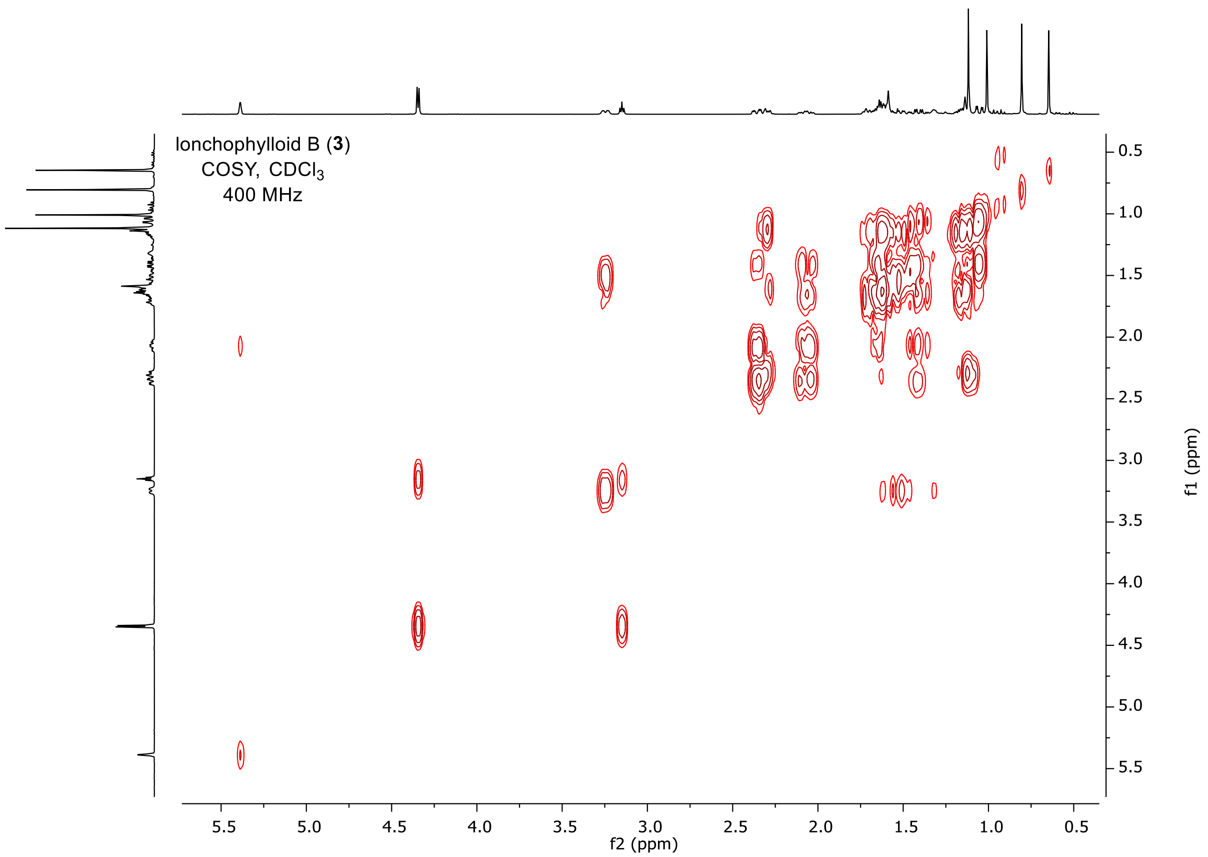

lonchophylloid B (**3**)
COSY, CDCl3
400 MHz
f2 (ppm)
f1 (ppm)

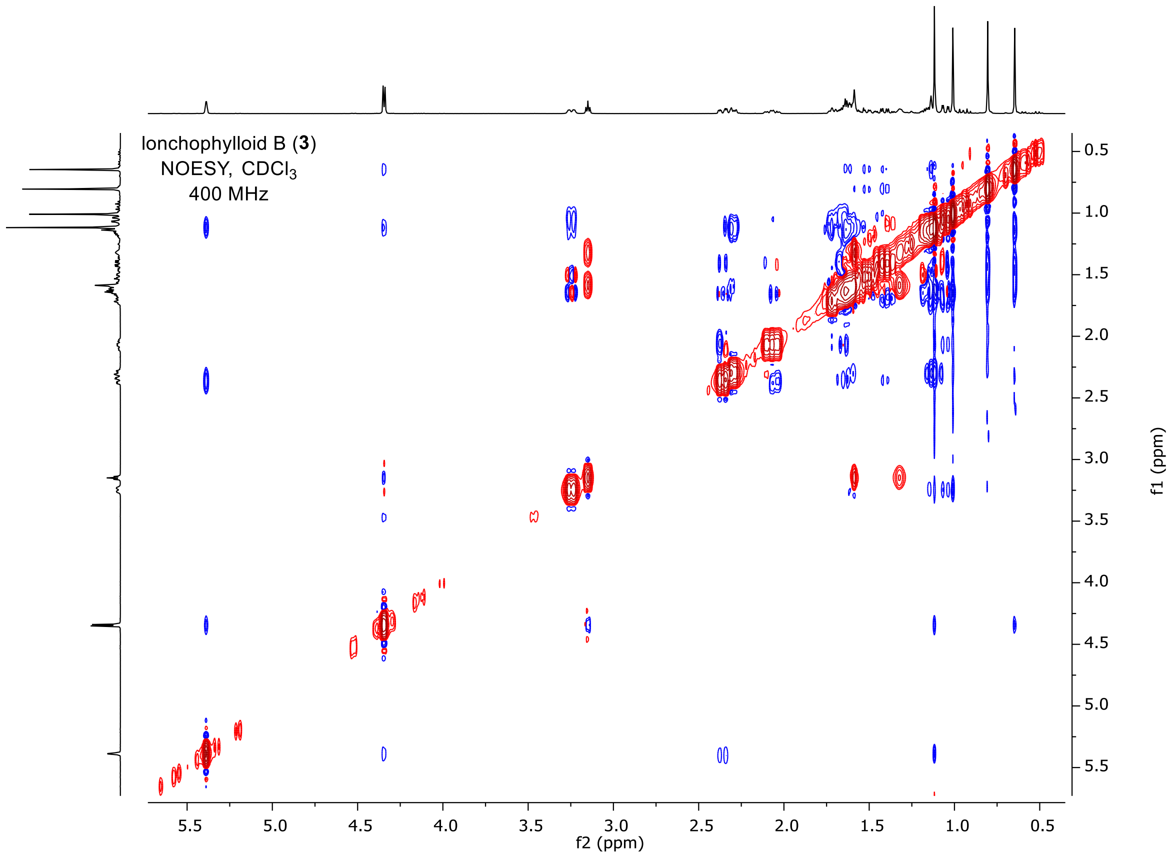

lonchophylloid B (**3**)
NOESY, CDCl3
400 MHz
f2 (ppm)
f1 (ppm)

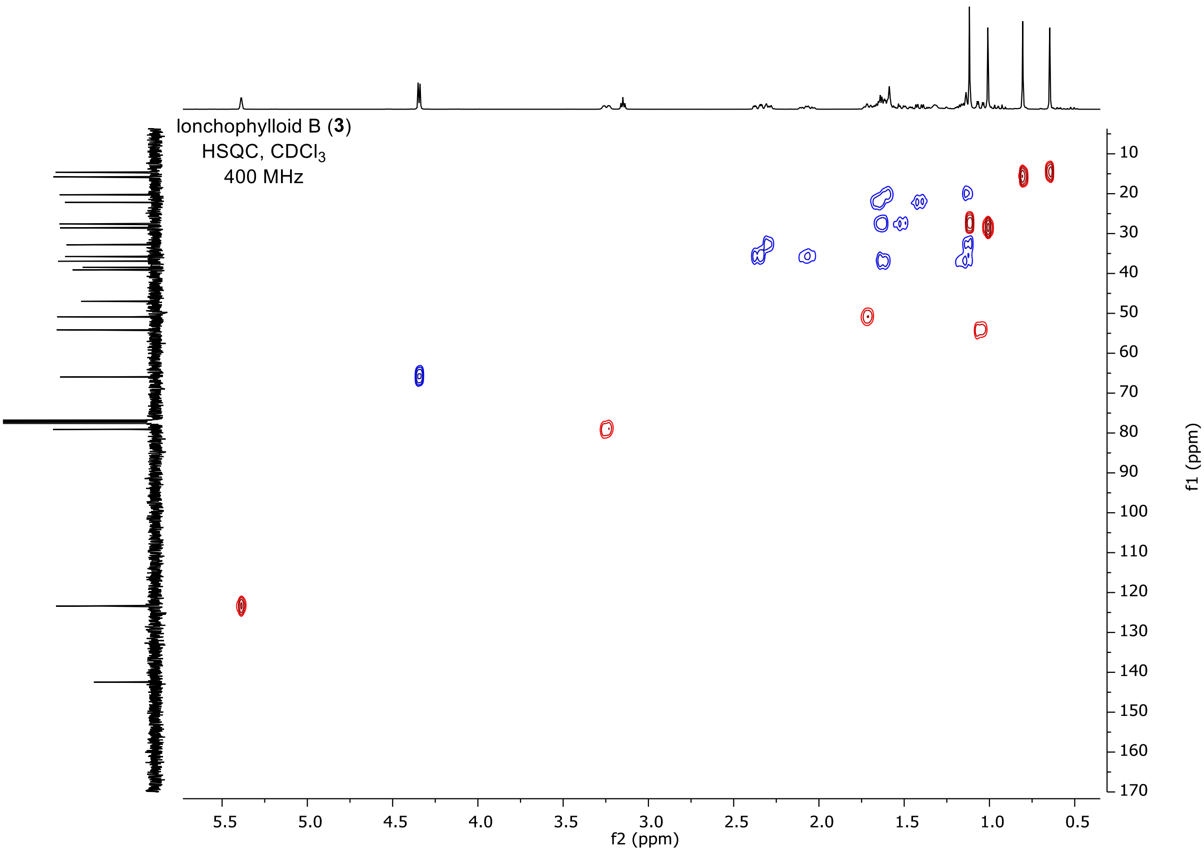
Ionchophylloid B (3)
HSQC, CDCl3
400 MHz
f2 (ppm)
f1 (ppm)

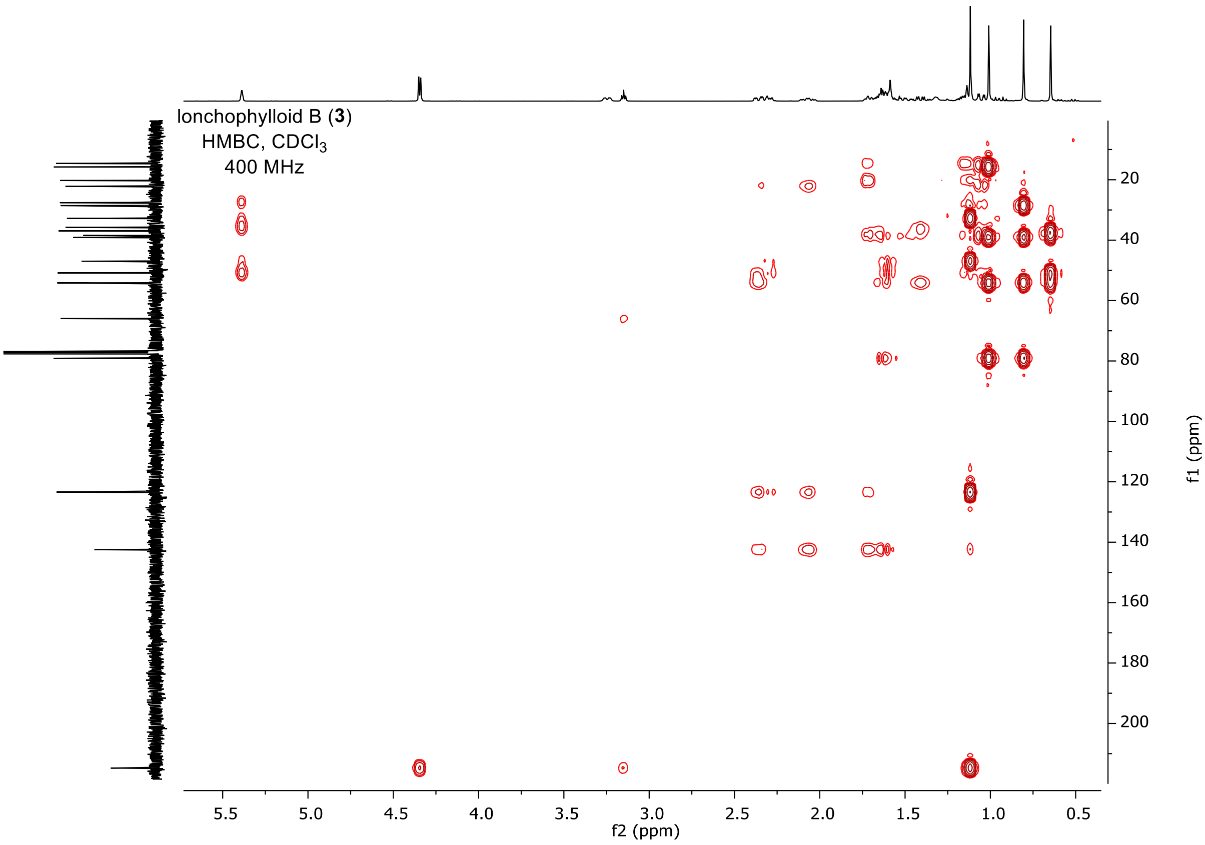
Ionchophylloid B (3)
HMBC, CDCl3
400 MHz
f2 (ppm)
f1 (ppm)

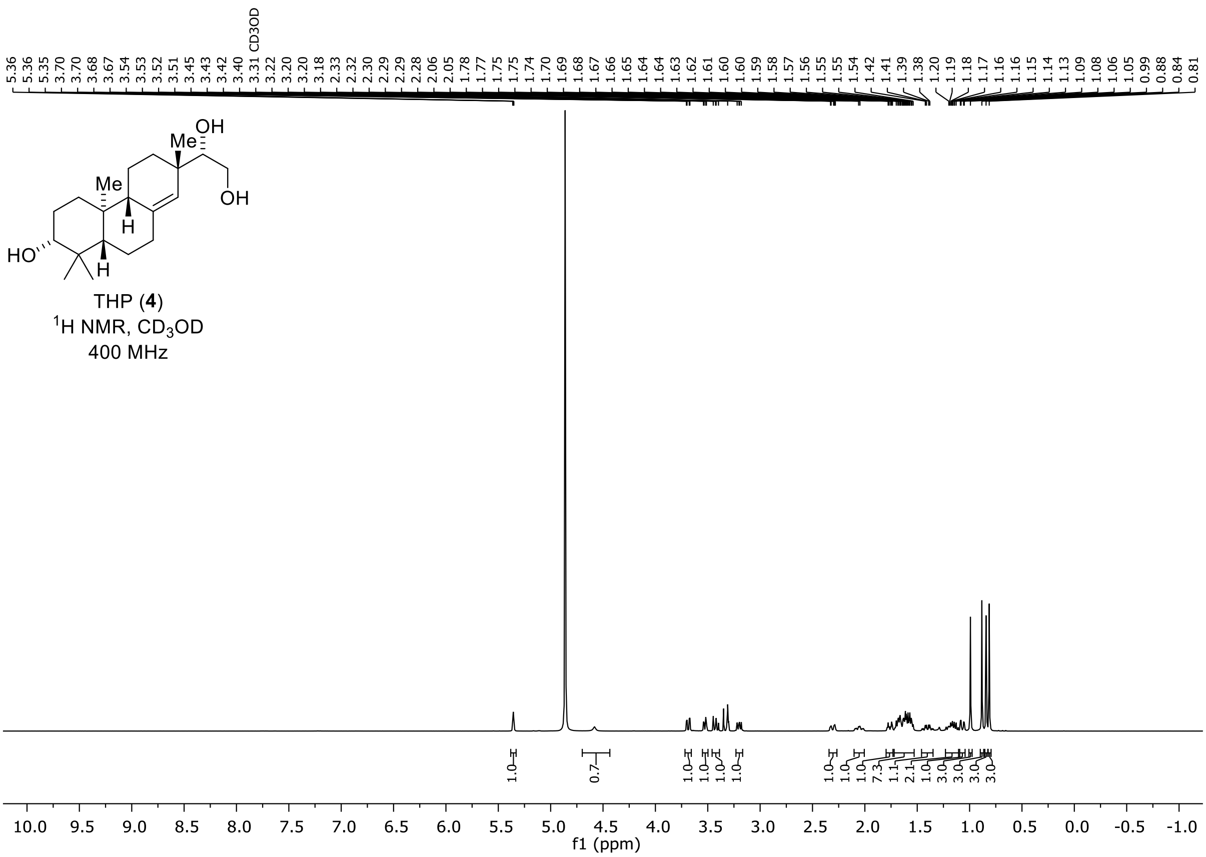
5.36 5.36 5.35 3.70 3.70 3.68 3.67 3.54 3.53 3.52 3.51 3.45 3.43 3.42 3.40 3.31 CD3OD 3.22 3.20 3.20 3.18 2.33 2.32 2.30 2.29 2.29 2.28 2.06 2.05 1.78 1.77 1.75 1.75 1.74 1.70 1.69 1.68 1.67 1.66 1.65 1.64 1.64 1.63 1.62 1.61 1.60 1.60 1.59 1.58 1.57 1.56 1.55 1.55 1.54 1.42 1.41 1.39 1.38 1.20 1.19 1.18 1.17 1.16 1.16 1.15 1.14 1.13 1.09 1.08 1.06 1.05 0.99 0.88 0.84 0.81
OH
Me
Me
OH
H
HO
H
THP (4)
1H NMR, CD3OD
400 MHz
f1 (ppm)

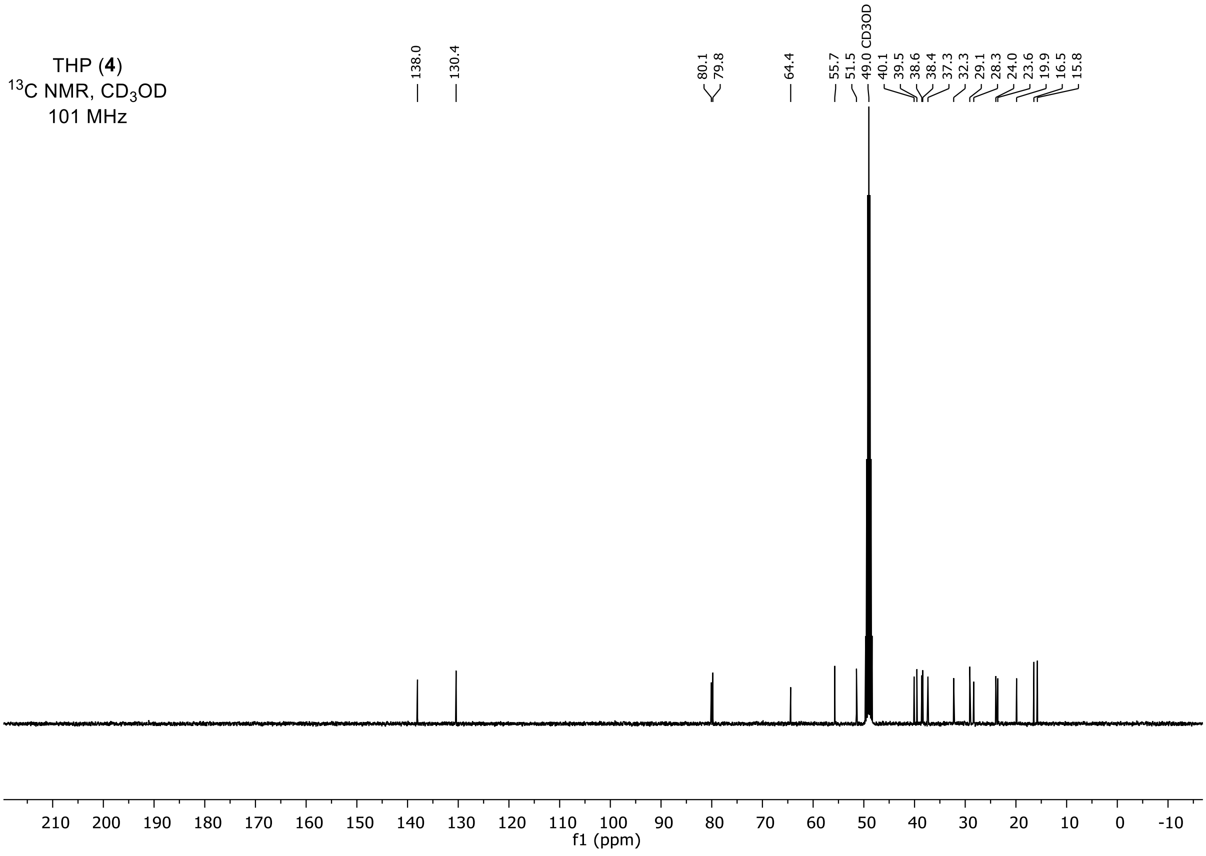
THP (4)
13C NMR, CD3OD
101 MHz
138.0 130.4 80.1 79.8 64.4 55.7 51.5 49.0 CD3OD 40.1 39.5 38.6 38.4 37.3 32.3 29.1 28.3 24.0 23.6 19.9 16.5 15.8
f1 (ppm)

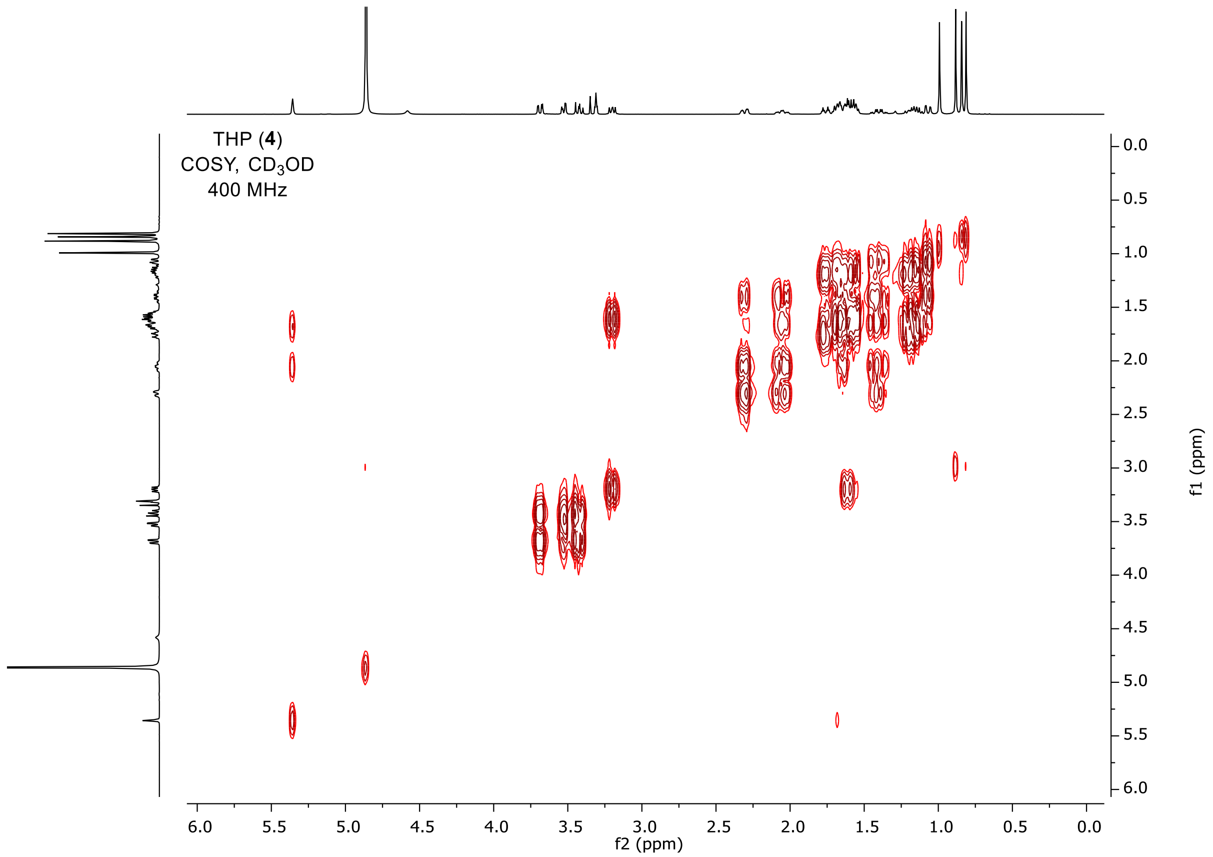
THP (4)
COSY, CD3OD
400 MHz
f2 (ppm)
f1 (ppm)

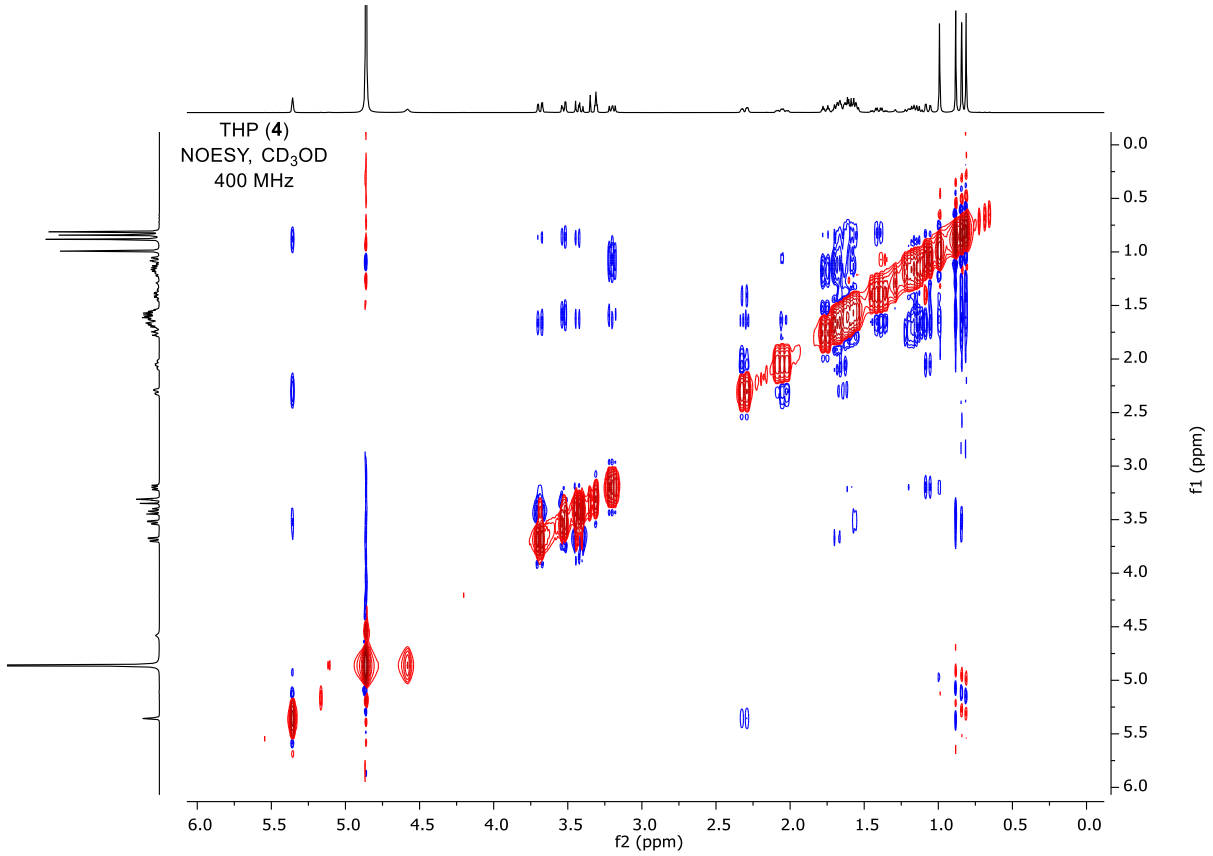
THP (4)
NOESY, CD3OD
400 MHz
f2 (ppm)
f1 (ppm)

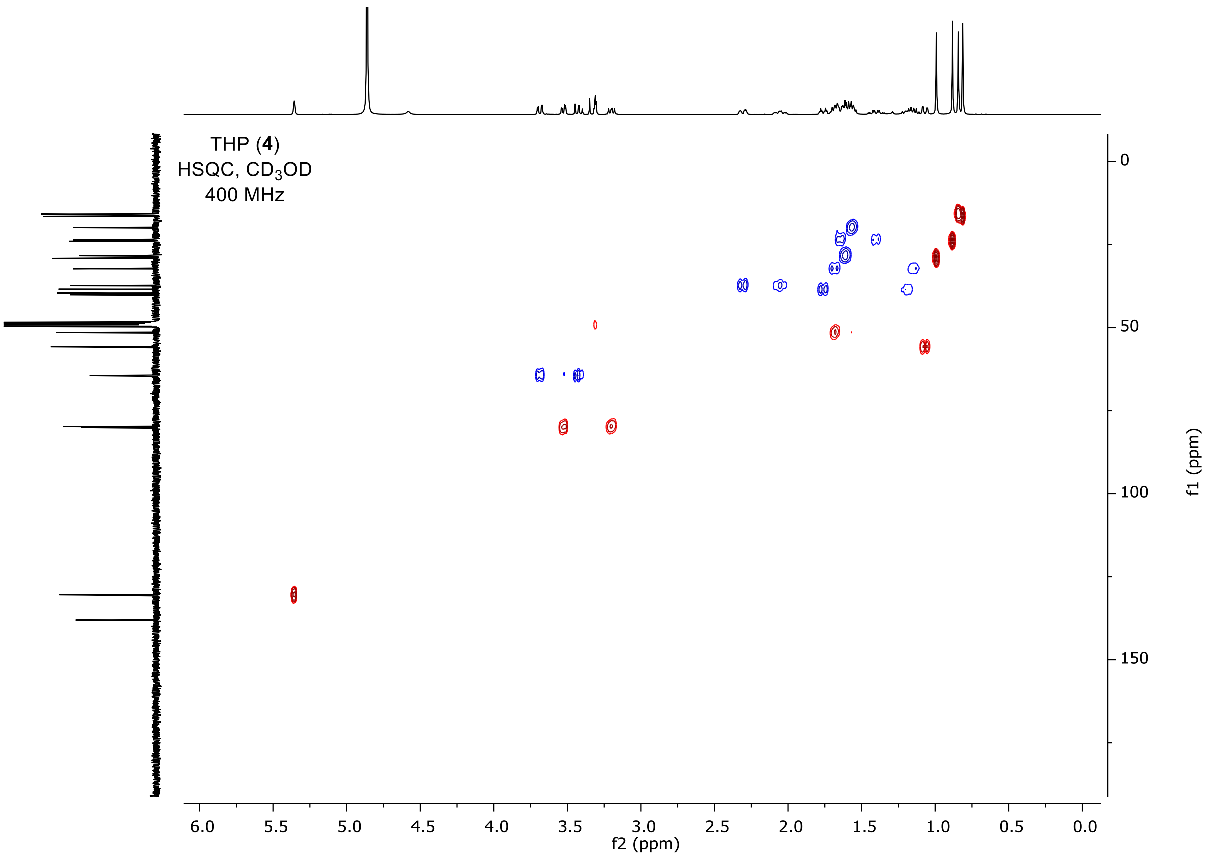
THP (4)
HSQC, CD_3OD
400 MHz
f2 (ppm)
f1 (ppm)

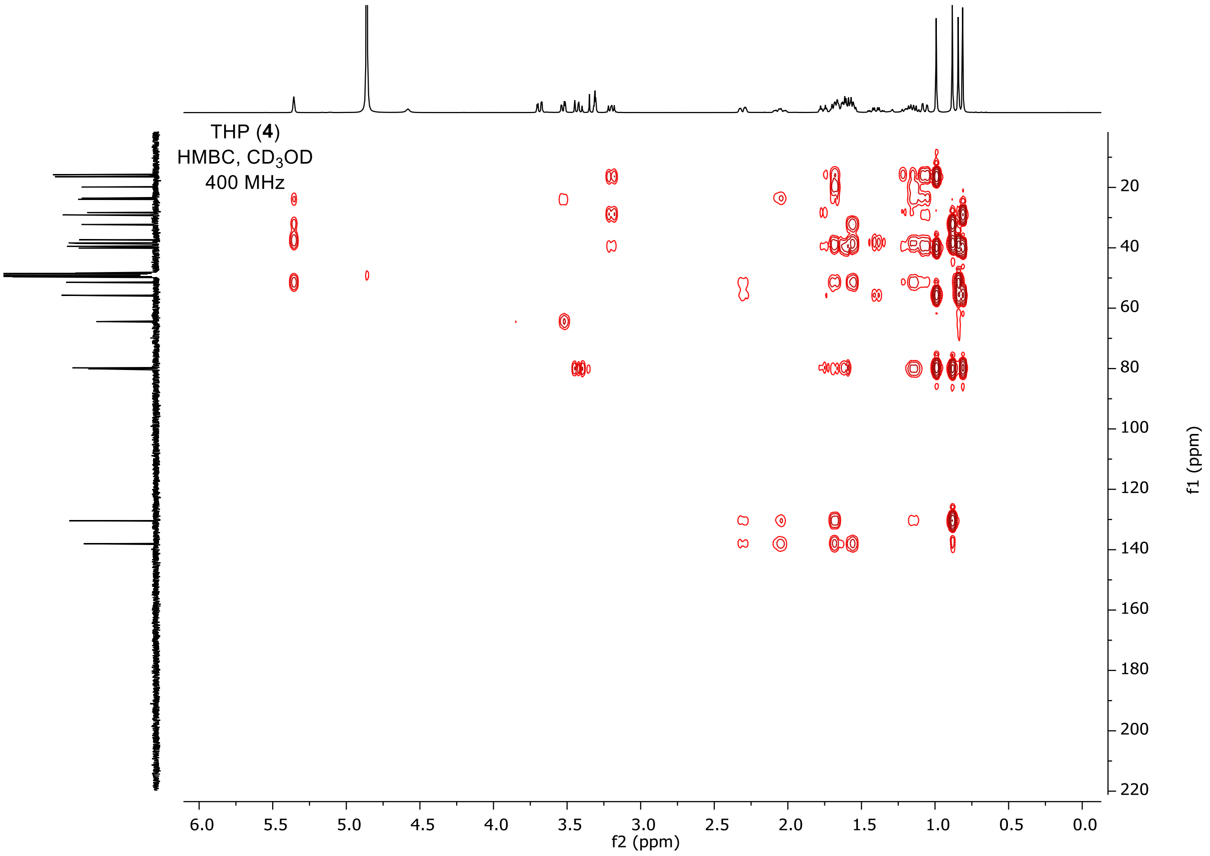
THP (4)
HMBC, CD_3OD
400 MHz
f2 (ppm)
f1 (ppm)

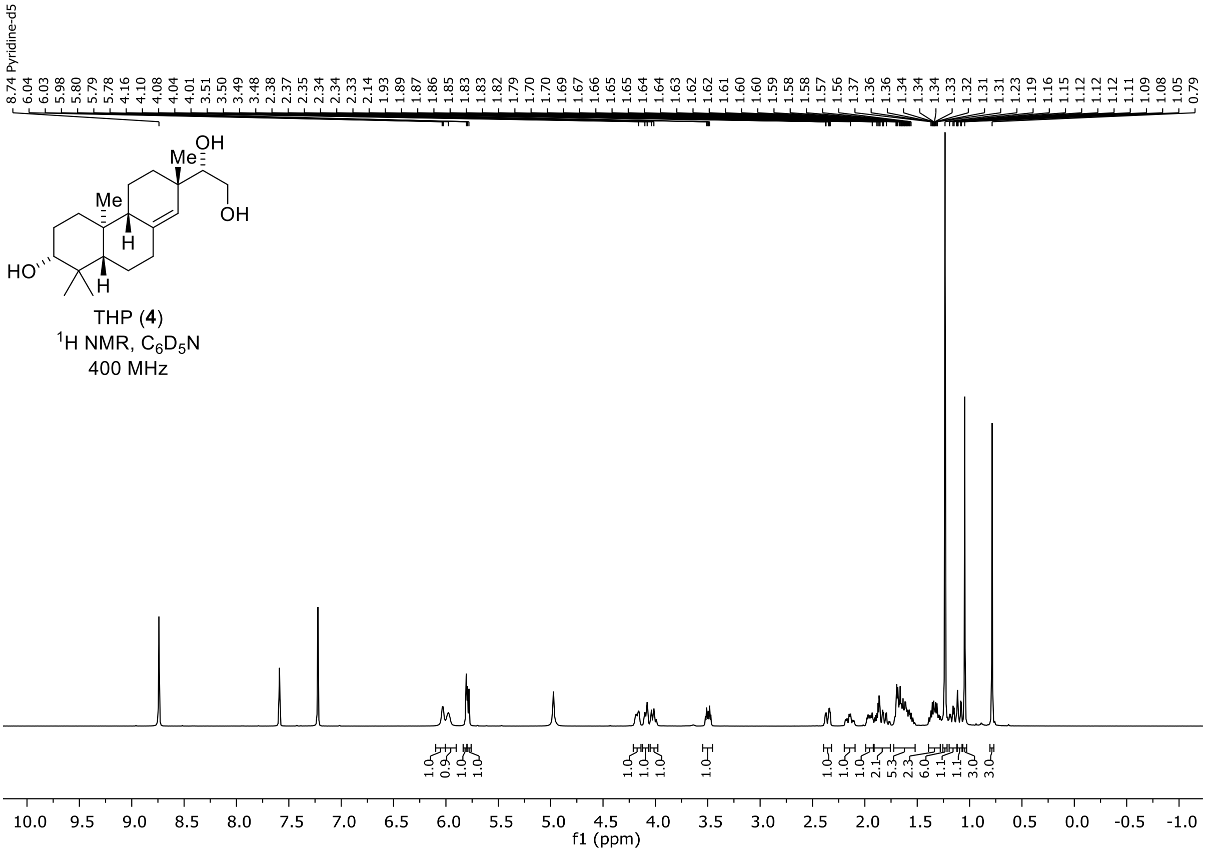
8.74 Pyridine-d5
6.04 6.03 5.98 5.80 5.79 5.78 4.16 4.10 4.08 4.04 4.01 3.51 3.50 3.49 3.48 2.38 2.37 2.35 2.34 2.34 2.33 2.14 1.93 1.89 1.87 1.86 1.85 1.83 1.83 1.82 1.79 1.70 1.70 1.69 1.67 1.66 1.65 1.65 1.64 1.64 1.63 1.62 1.62 1.61 1.60 1.60 1.59 1.58 1.58 1.57 1.56 1.37 1.36 1.36 1.34 1.34 1.34 1.33 1.32 1.31 1.31 1.23 1.19 1.16 1.15 1.12 1.12 1.12 1.11 1.09 1.08 1.05 0.79
OH
Me
OH
Me
H
HO
H
THP (4)
1H NMR, C6D5N
400 MHz
1.0 0.9 1.0 1.0 1.0 1.0 1.0 1.0 1.0 1.0 1.0 2.1 5.3 2.3 6.0 1.1 1.1 3.0 3.0
10.0 9.5 9.0 8.5 8.0 7.5 7.0 6.5 6.0 5.5 5.0 4.5 4.0 3.5 3.0 2.5 2.0 1.5 1.0 0.5 0.0 -0.5 -1.0
f1 (ppm)

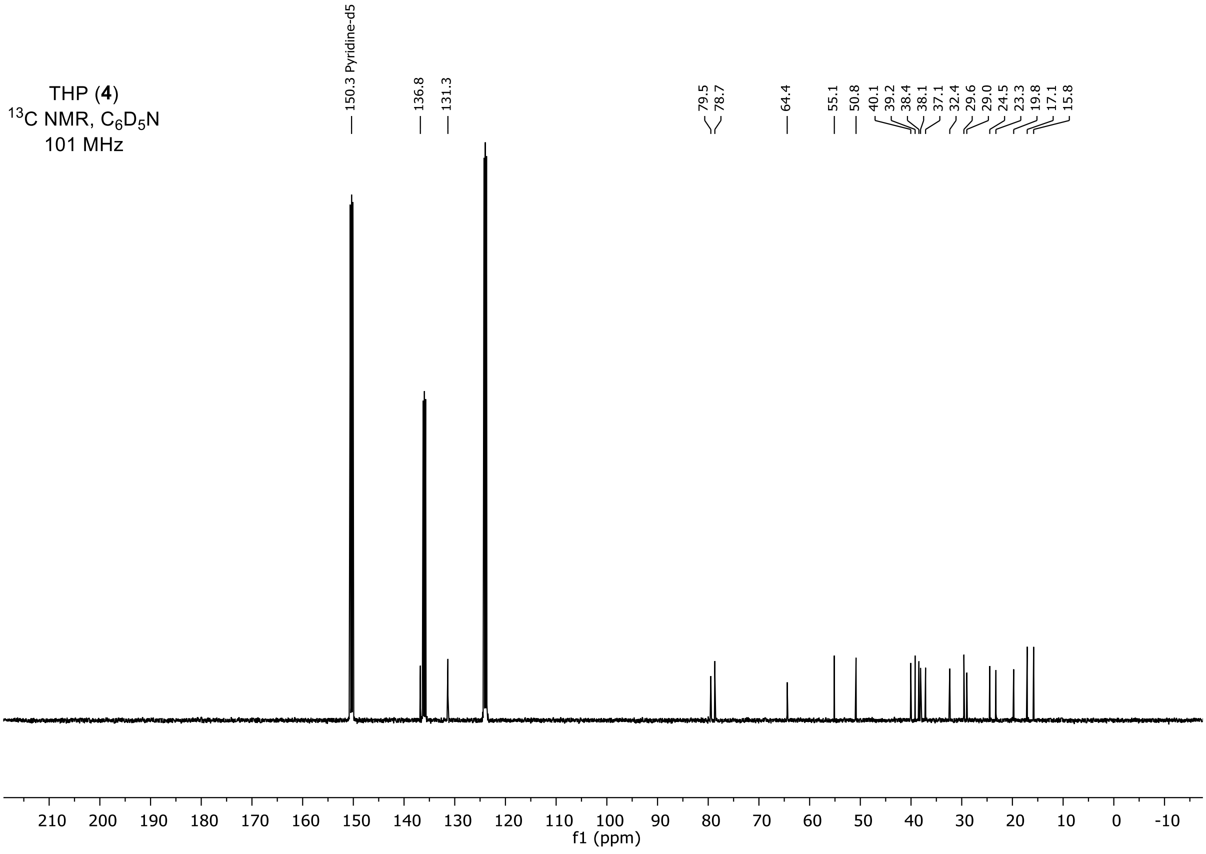
THP (4)
13C NMR, C6D5N
101 MHz
150.3 Pyridine-d5
136.8
131.3
79.5 78.7
64.4
55.1
50.8
40.1 39.2 38.4 38.1 37.1 32.4 29.6 29.0 24.5 23.3 19.8 17.1 15.8
210 200 190 180 170 160 150 140 130 120 110 100 90 80 70 60 50 40 30 20 10 0 -10
f1 (ppm)

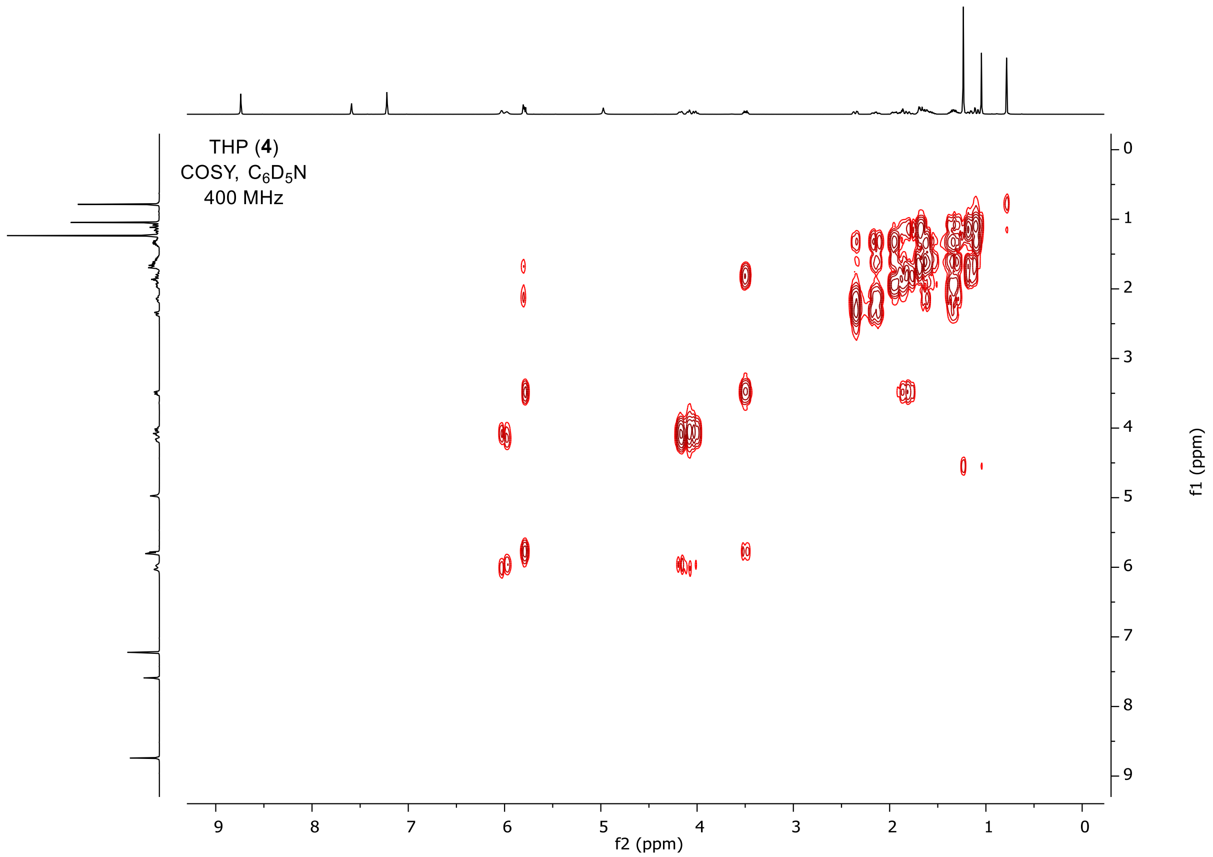
THP (**4**)
COSY, C_6D_5N
400 MHz
f2 (ppm)
f1 (ppm)

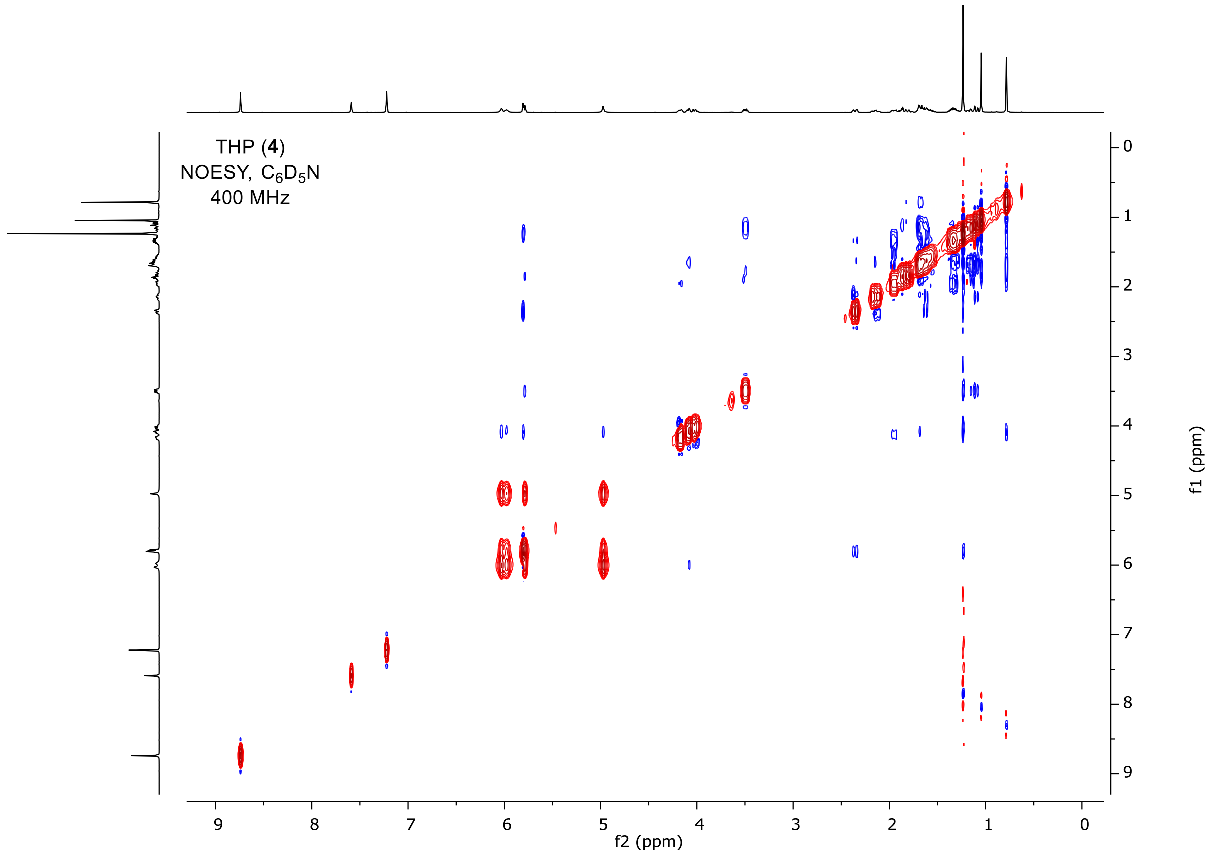
THP (**4**)
NOESY, C_6D_5N
400 MHz
f2 (ppm)
f1 (ppm)

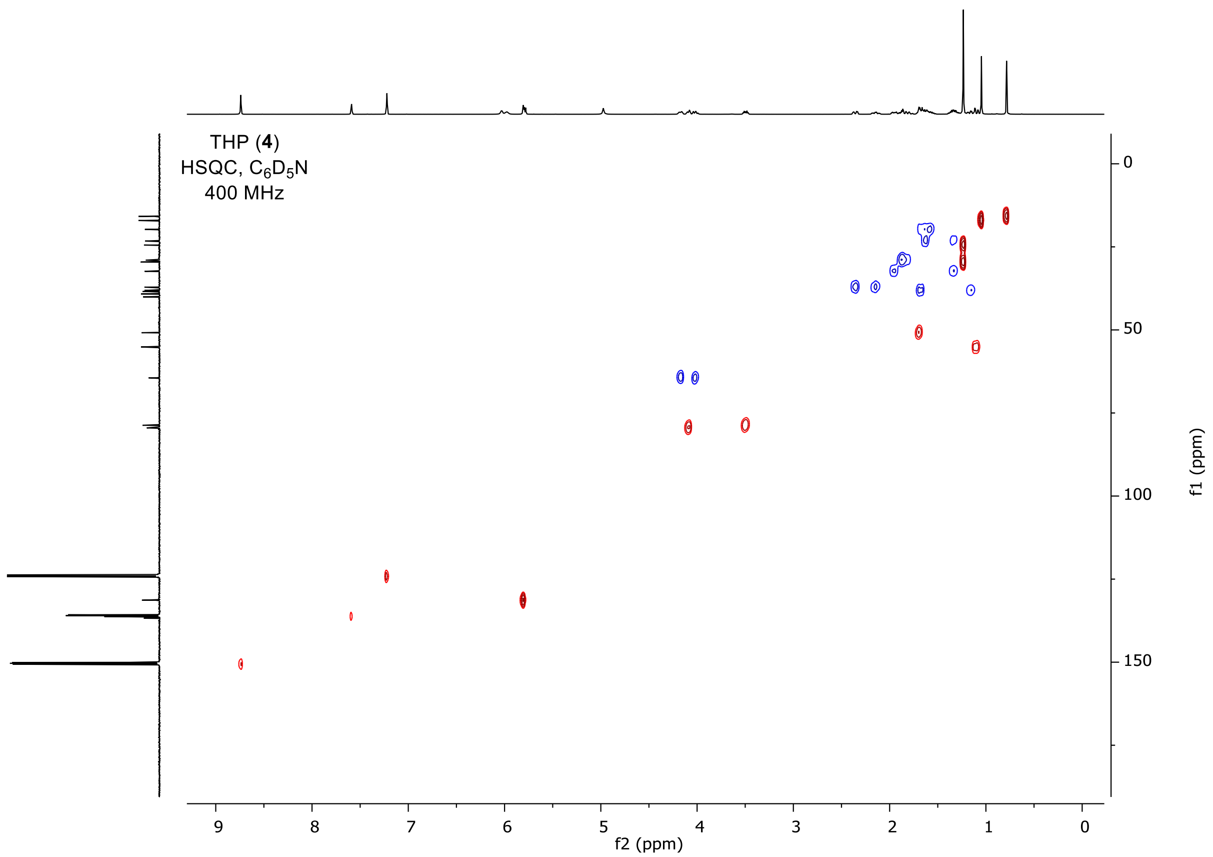
THP (**4**)
HSQC, C_6D_5N
400 MHz
f2 (ppm)
f1 (ppm)

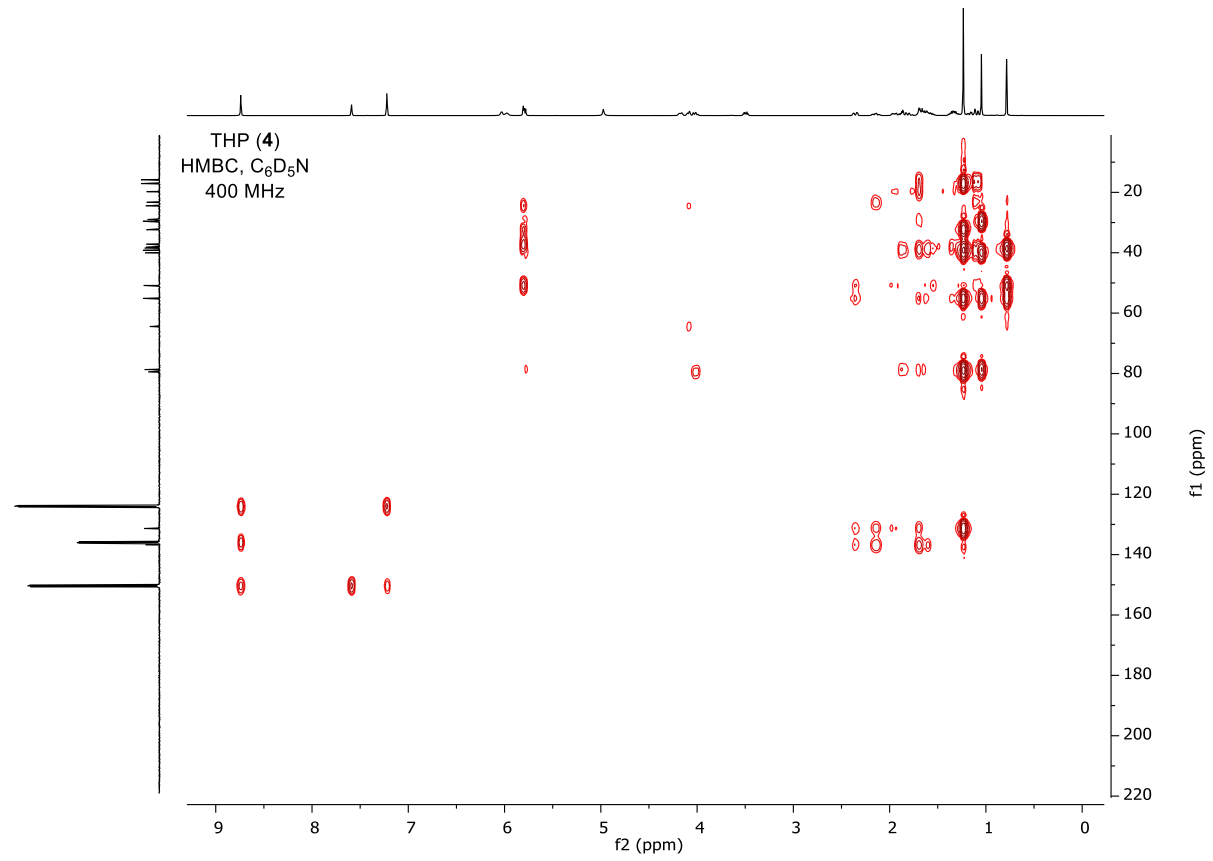
THP (**4**)
HMBC, C_6D_5N
400 MHz
f2 (ppm)
f1 (ppm)

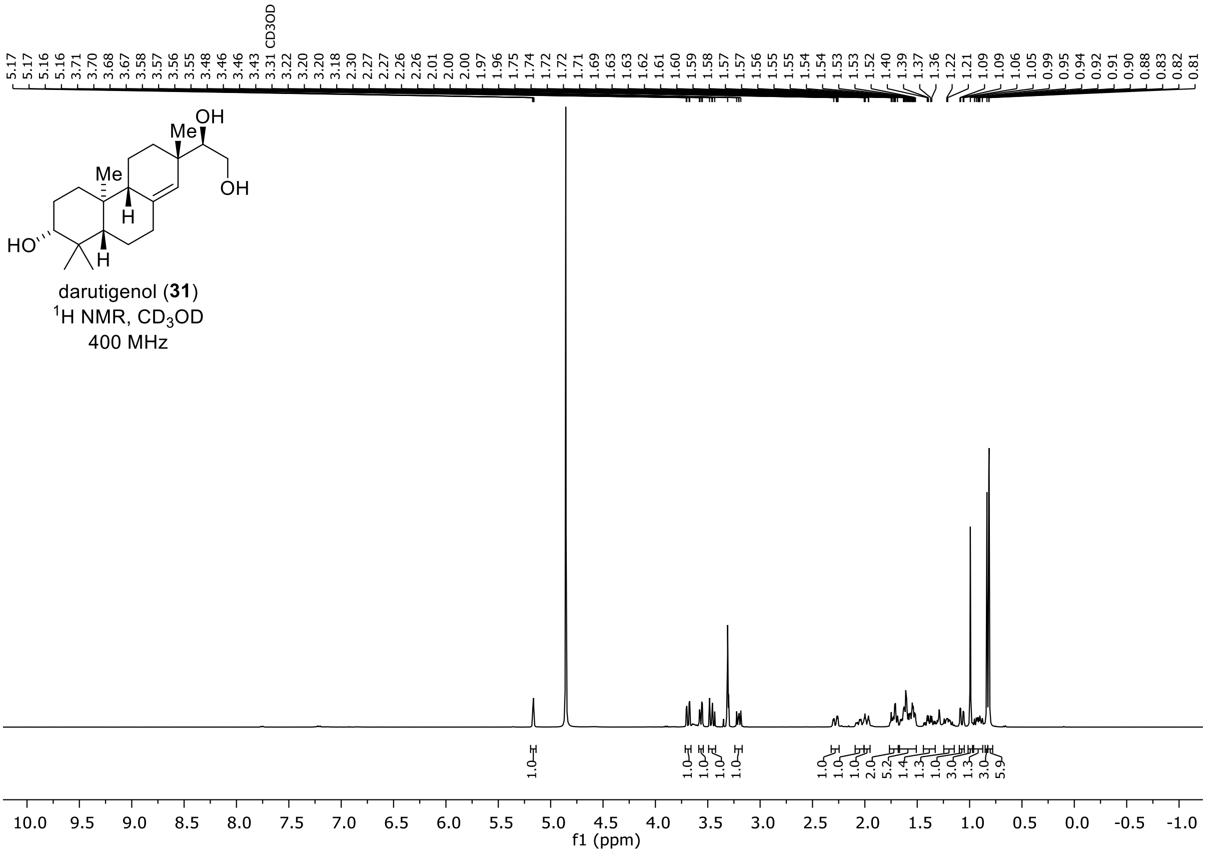

darutigenol (**31**)
^{1}H NMR, CD_3OD
400 MHz

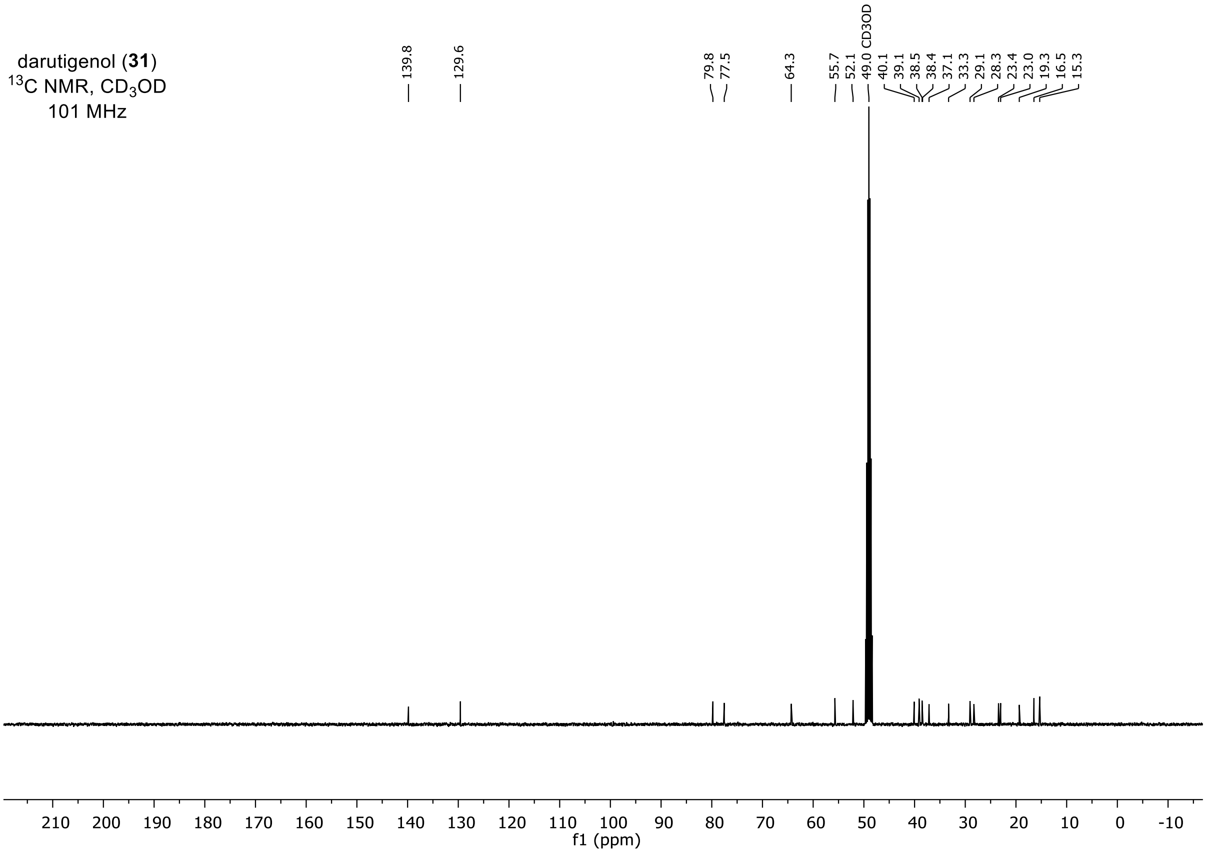

darutigenol (**31**)
^{13}C NMR, CD_3OD
101 MHz

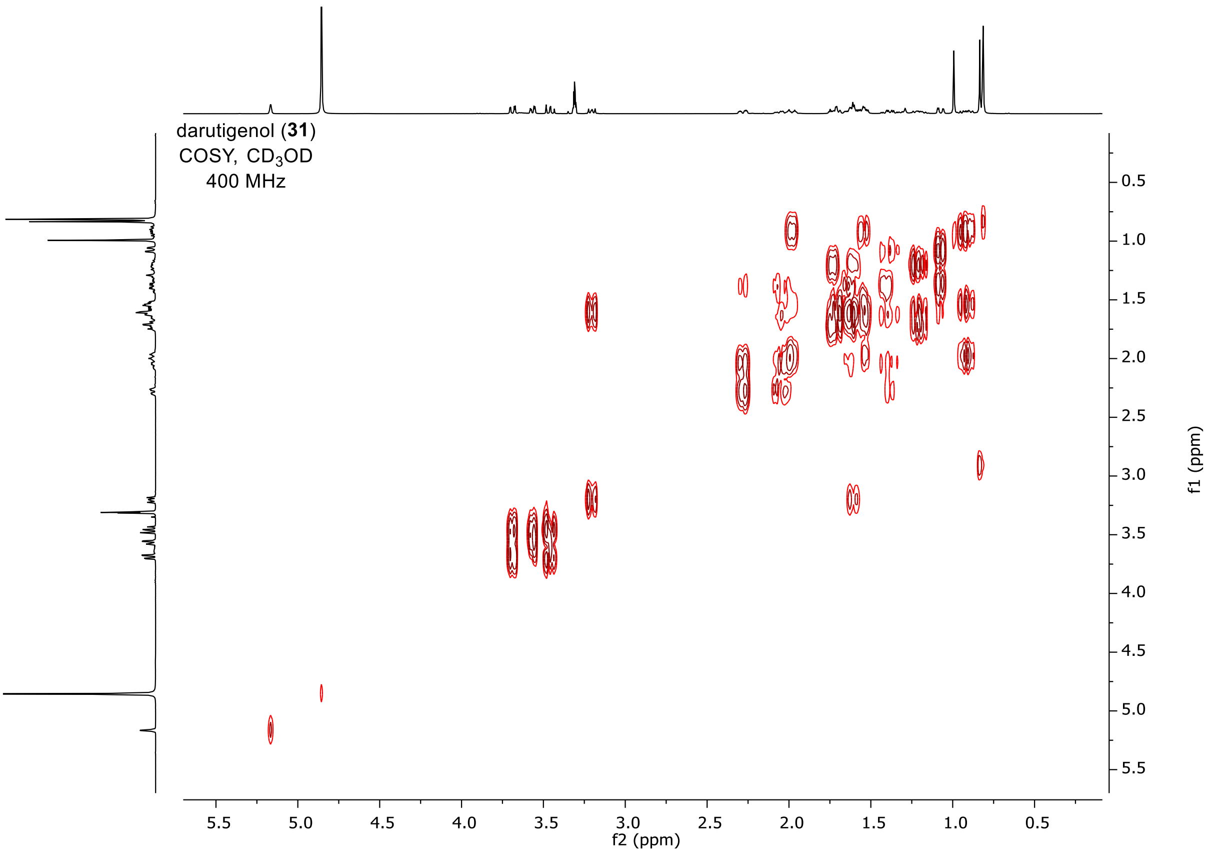
darutigenol (**31**)
COSY, CD_3OD
400 MHz
f2 (ppm)
f1 (ppm)

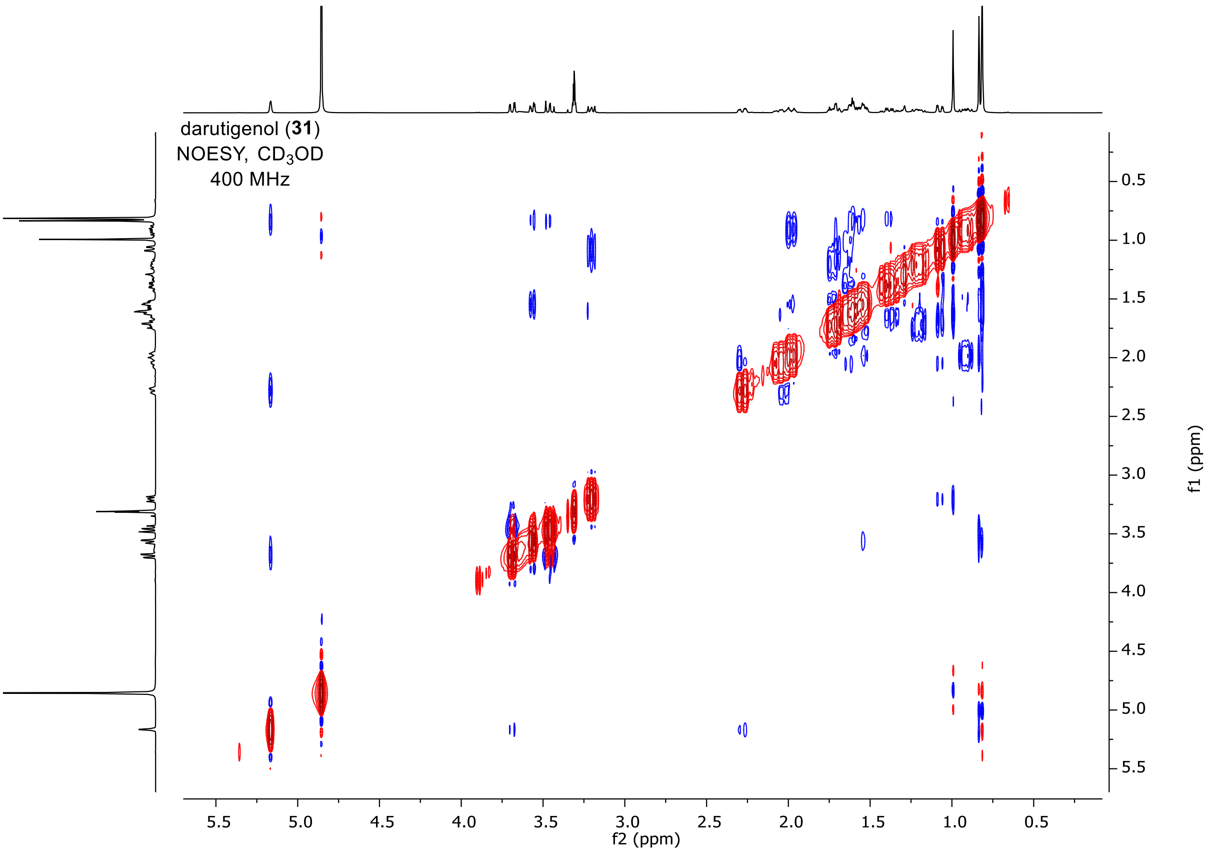
darutigenol (**31**)
NOESY, CD_3OD
400 MHz
f2 (ppm)
f1 (ppm)

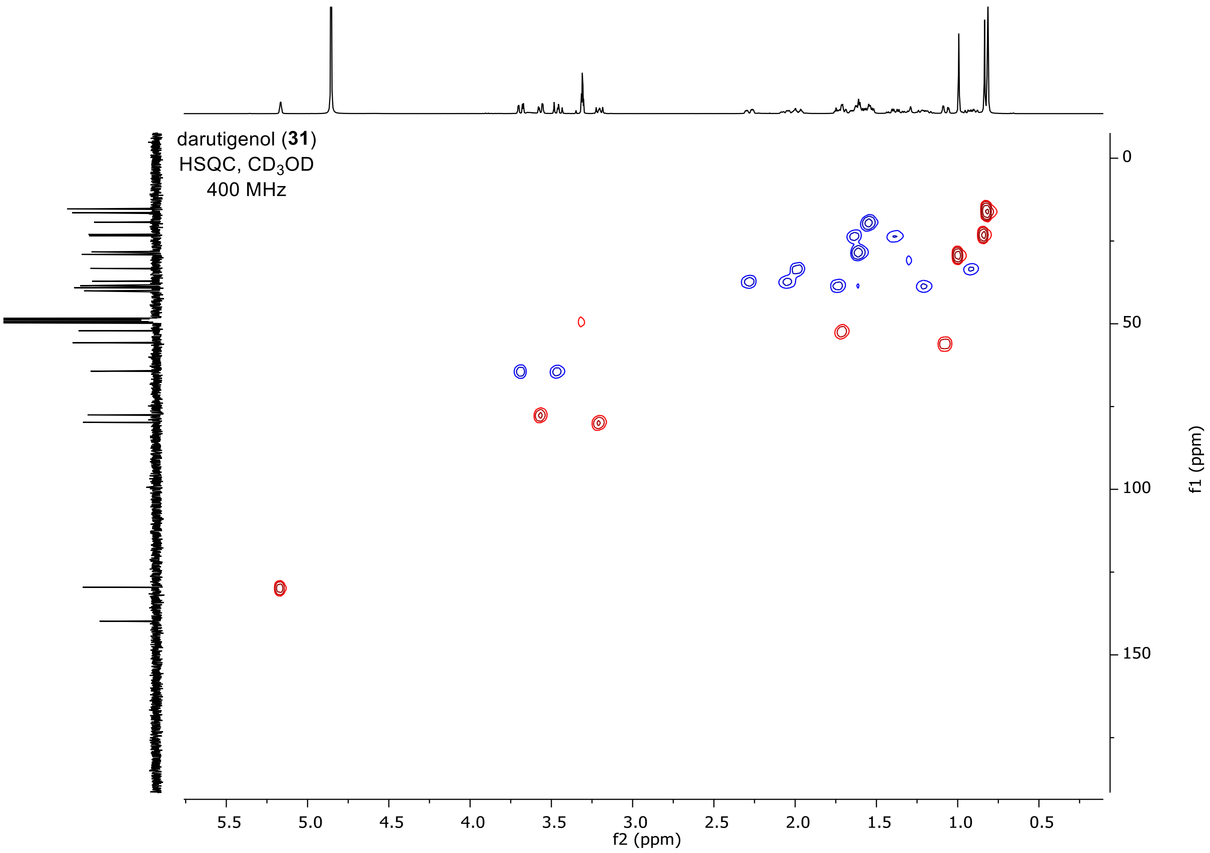

darutigenol (**31**)
HSQC, CD_3OD
400 MHz
f2 (ppm)
f1 (ppm)

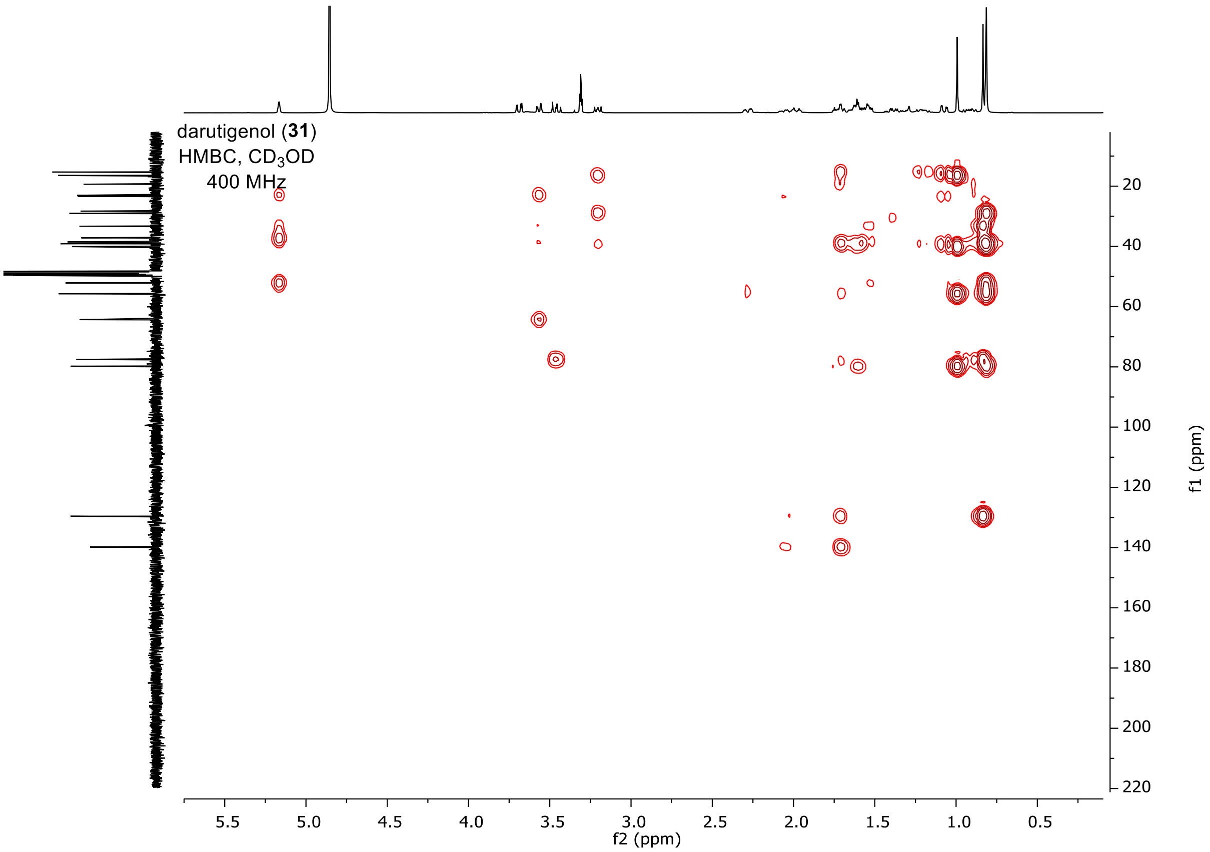

darutigenol (**31**)
HMBC, CD_3OD
400 MHz
f2 (ppm)
f1 (ppm)

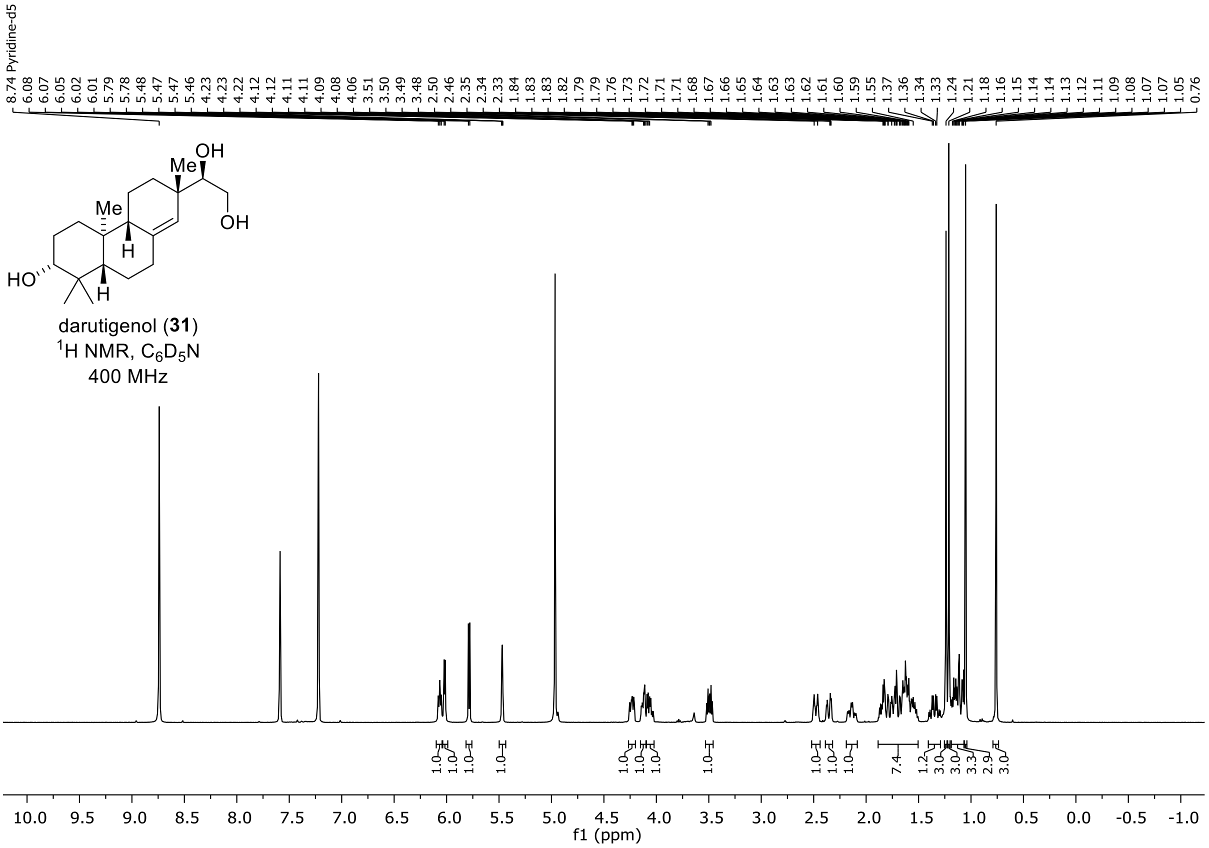
8.74 Pyridine-d5
6.08 6.07 6.05 6.02 6.01 5.79 5.78 5.48 5.47 5.46 4.23 4.23 4.22 4.12 4.12 4.11 4.11 4.09 4.08 4.06 3.51 3.50 3.49 3.48 2.50 2.46 2.35 2.34 2.33 1.84 1.83 1.83 1.82 1.79 1.79 1.76 1.73 1.72 1.71 1.71 1.68 1.67 1.66 1.65 1.64 1.63 1.63 1.62 1.61 1.60 1.59 1.55 1.37 1.36 1.34 1.33 1.24 1.21 1.18 1.16 1.15 1.14 1.14 1.13 1.12 1.11 1.09 1.08 1.07 1.07 1.05 0.76
Me
OH
Me
OH
H
HO
H
darutigenol (31)
1H NMR, C6D5N
400 MHz
f1 (ppm)

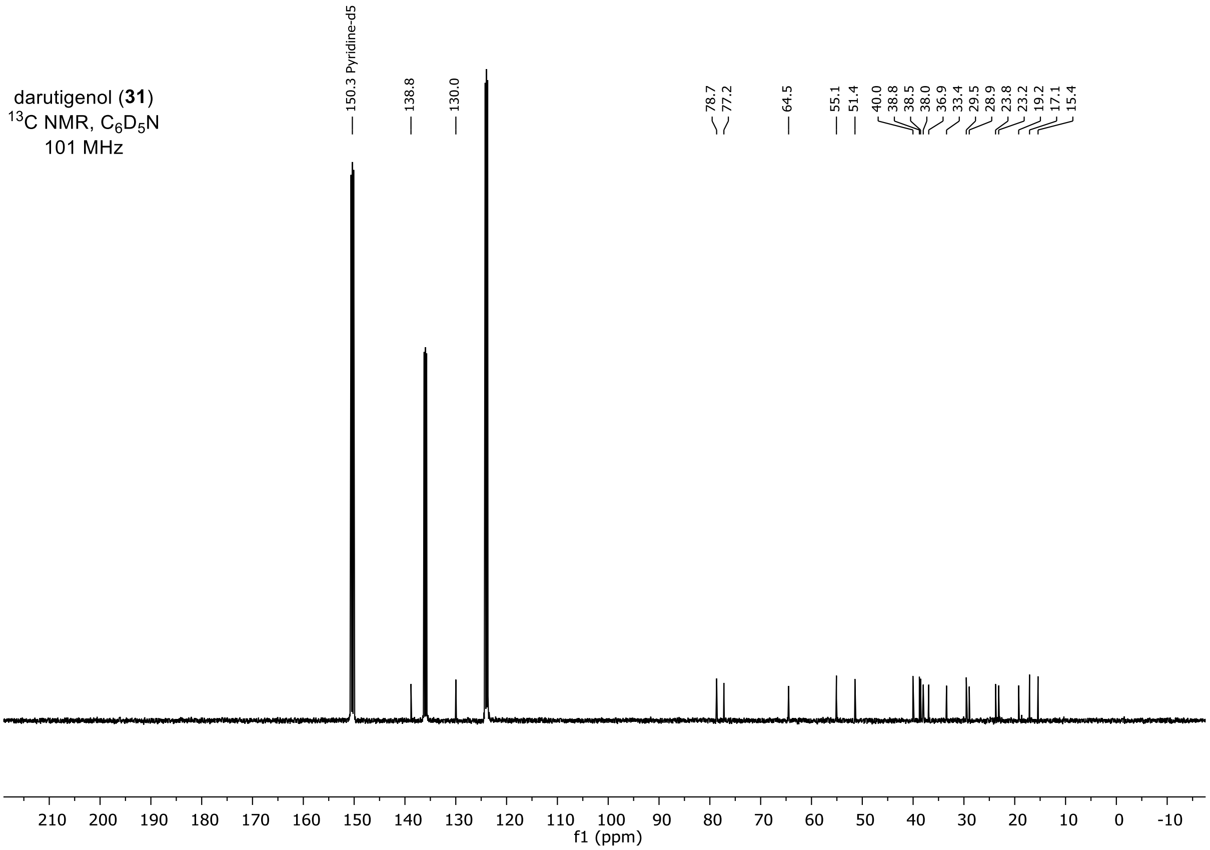
darutigenol (31)
13C NMR, C6D5N
101 MHz
150.3 Pyridine-d5
138.8
130.0
78.7 77.2
64.5
55.1 51.4
40.0 38.8 38.5 38.0 36.9 33.4 29.5 28.9 23.8 23.2 19.2 17.1 15.4
f1 (ppm)

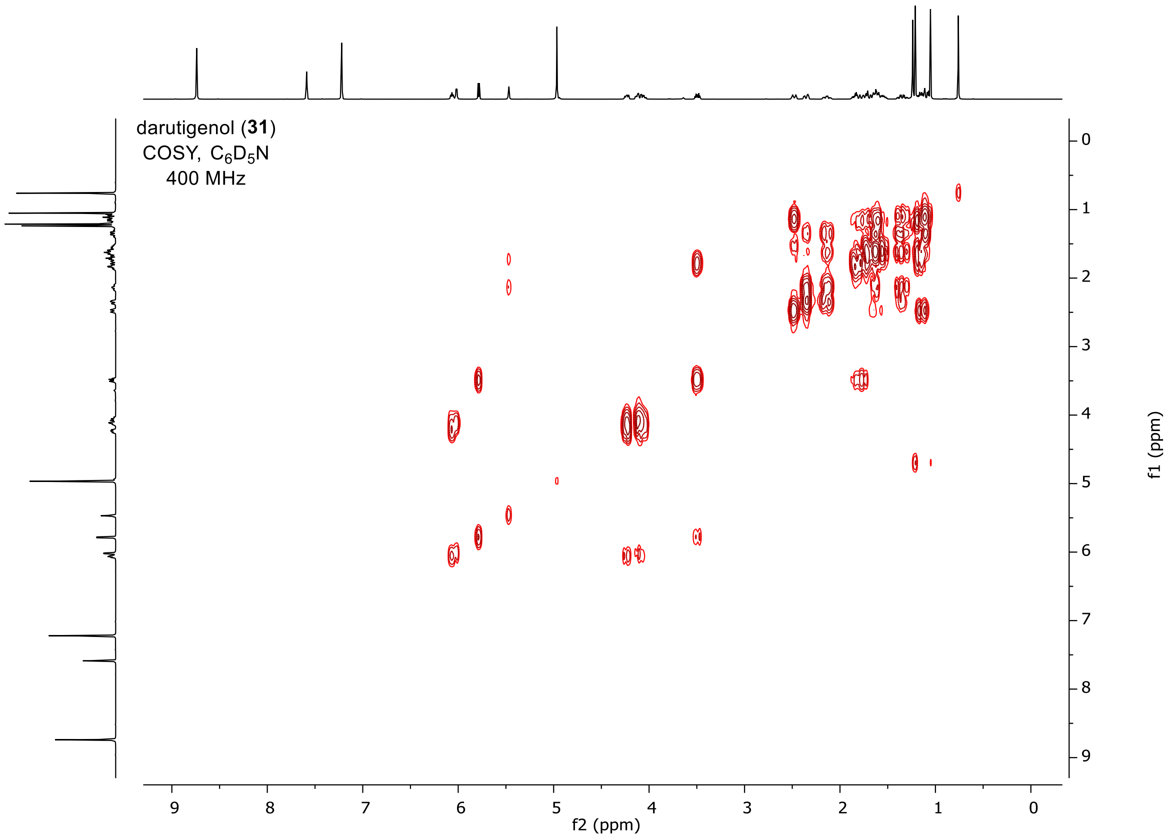
darutigenol (**31**)
COSY, C_6D_5N
400 MHz
f1 (ppm)
f2 (ppm)

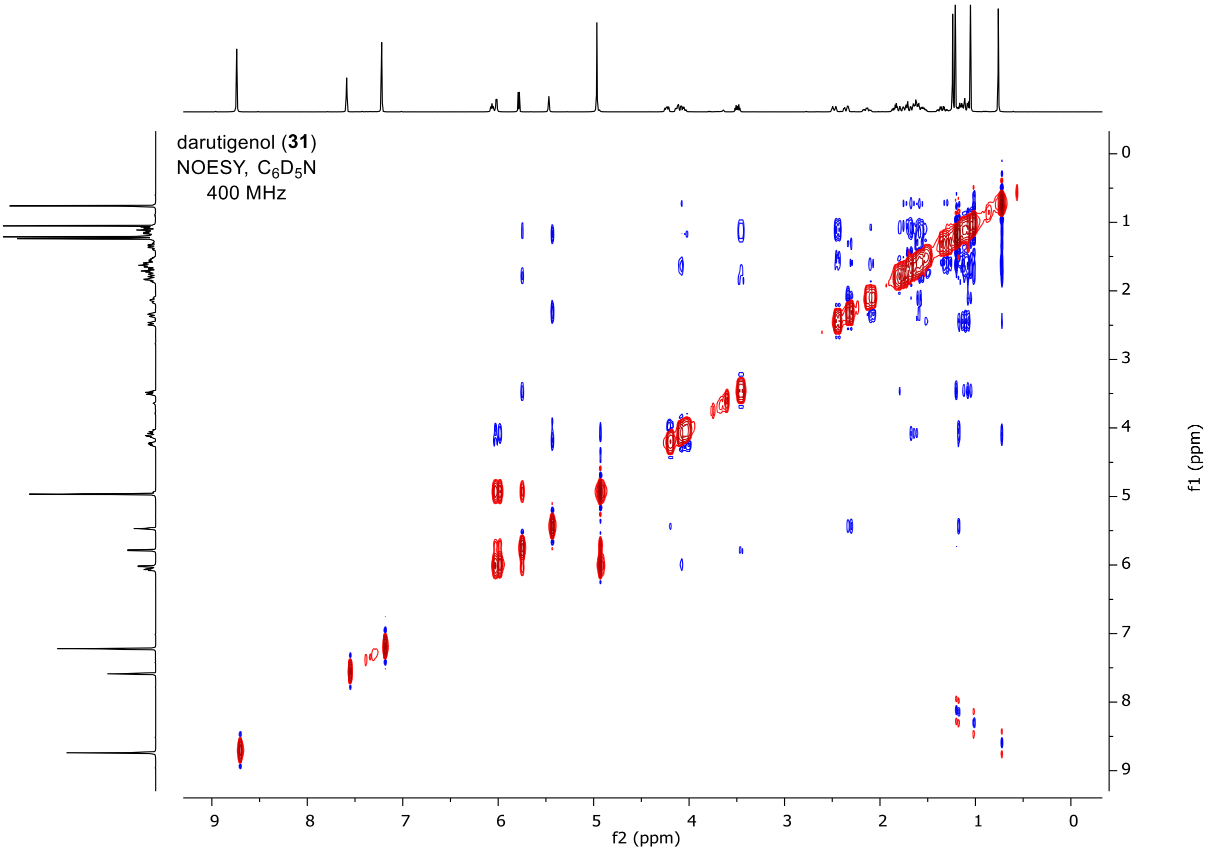
darutigenol (**31**)
NOESY, C_6D_5N
400 MHz
f1 (ppm)
f2 (ppm)

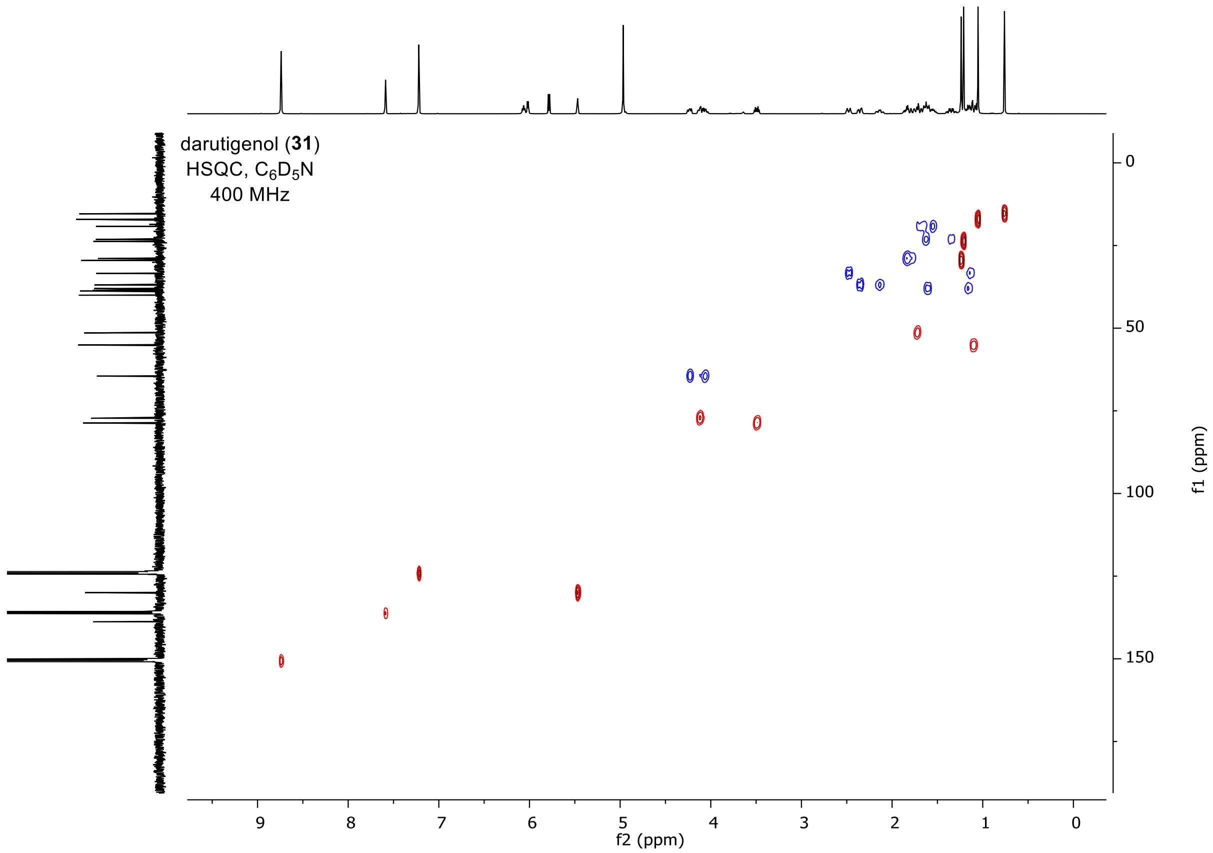
darutigenol (**31**)
HSQC, C_6D_5N
400 MHz
f1 (ppm)
f2 (ppm)

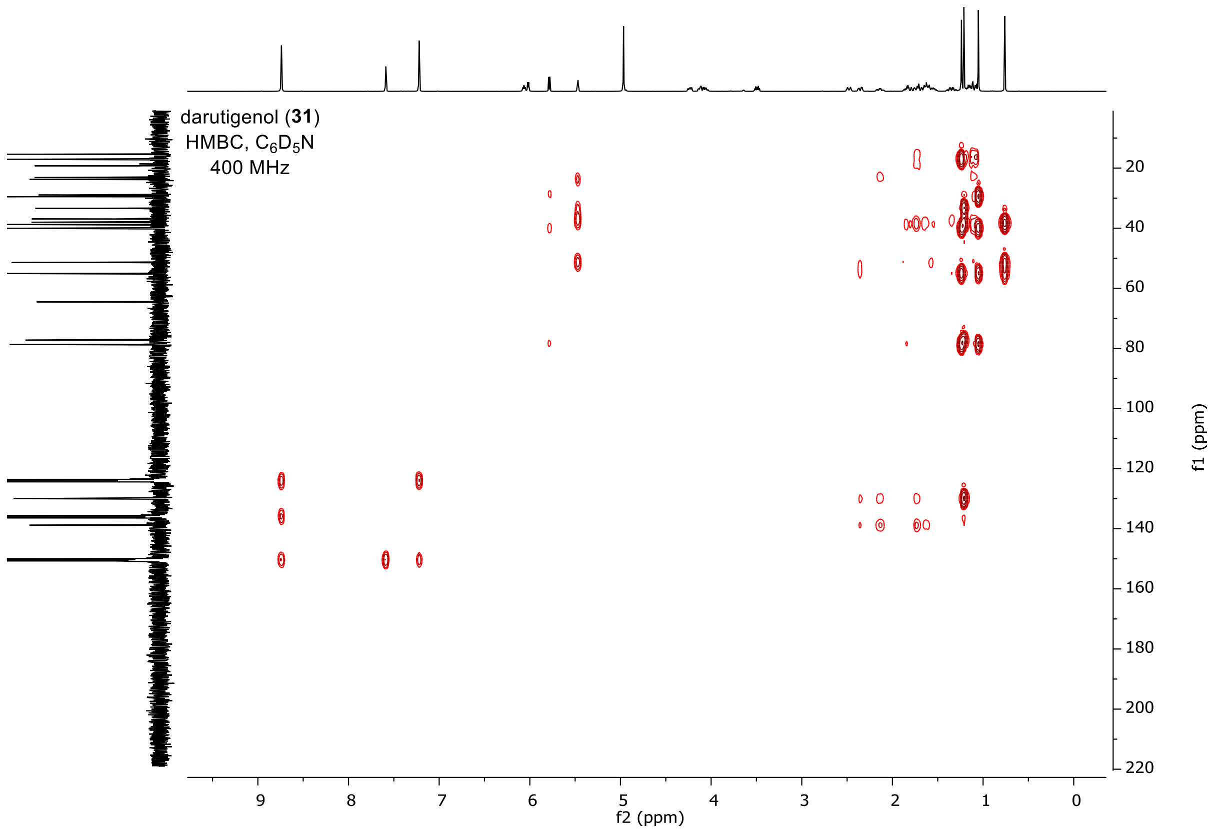
darutigenol (**31**)
HMBC, C_6D_5N
400 MHz
f1 (ppm)
f2 (ppm)

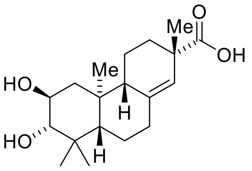

DHPA (**29**)
^{1}H NMR, CD_3OD
400 MHz

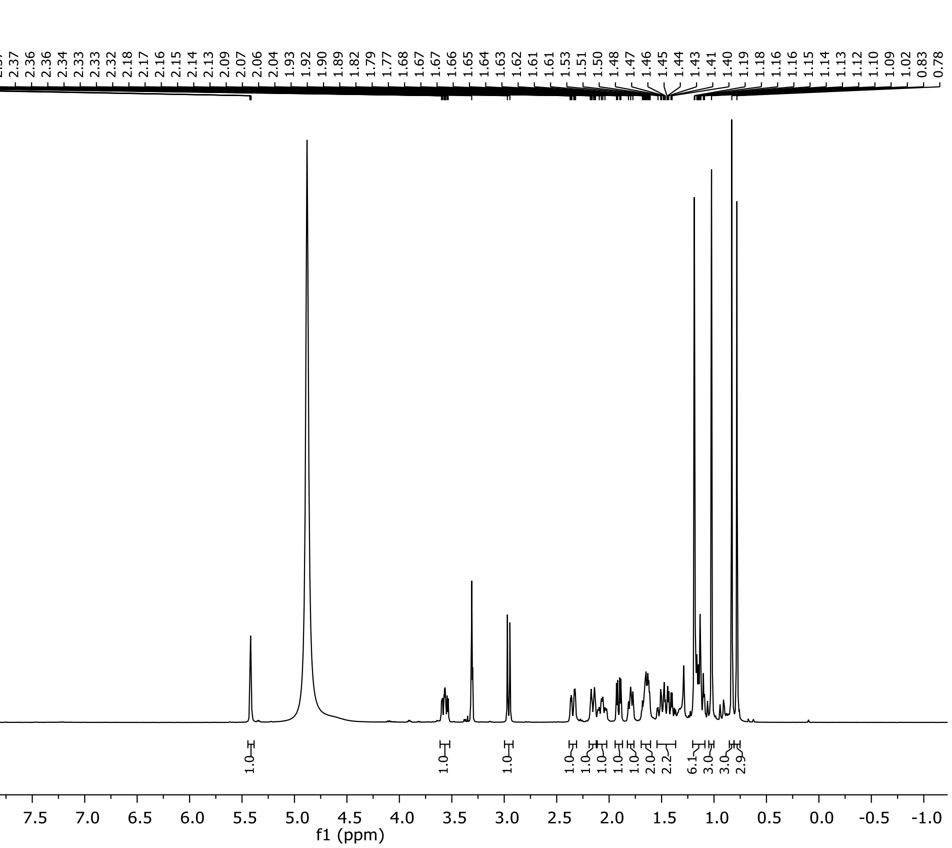

DHPA (**29**)
^{13}C NMR, CD_3OD
101 MHz

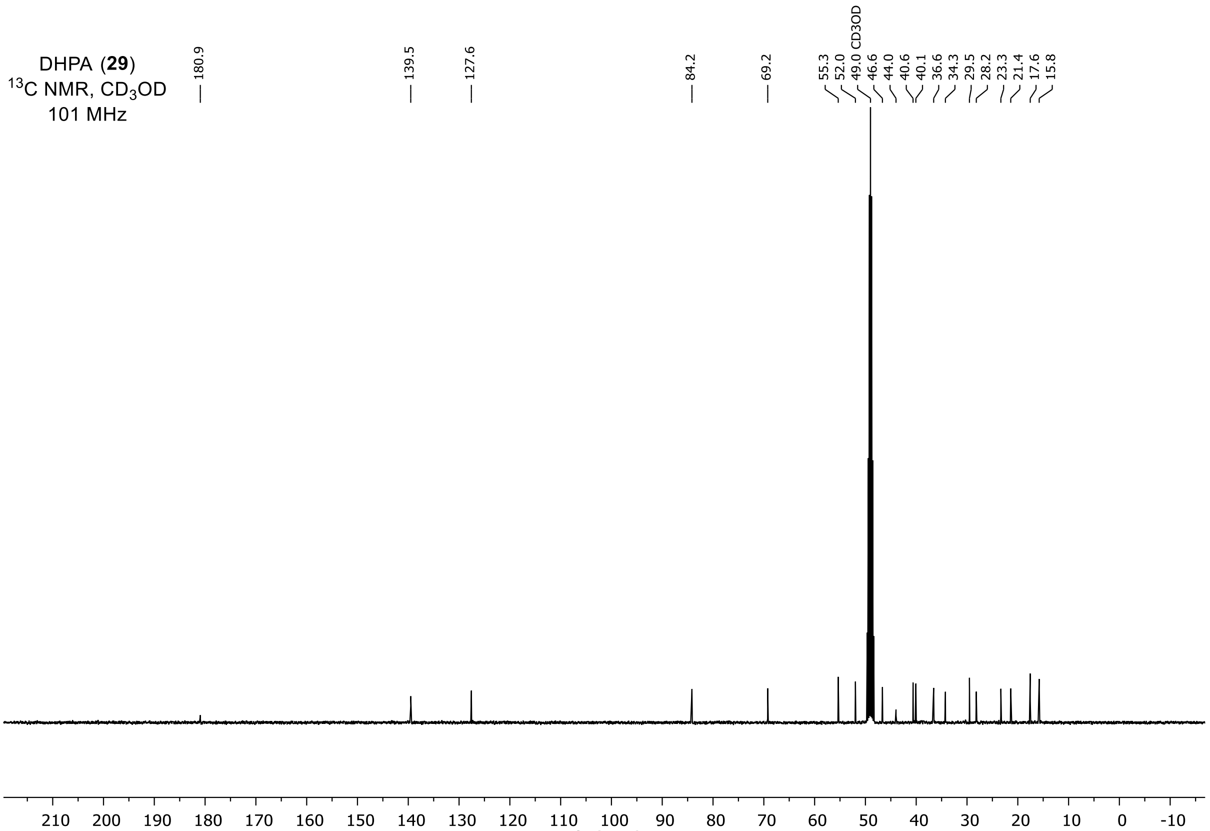

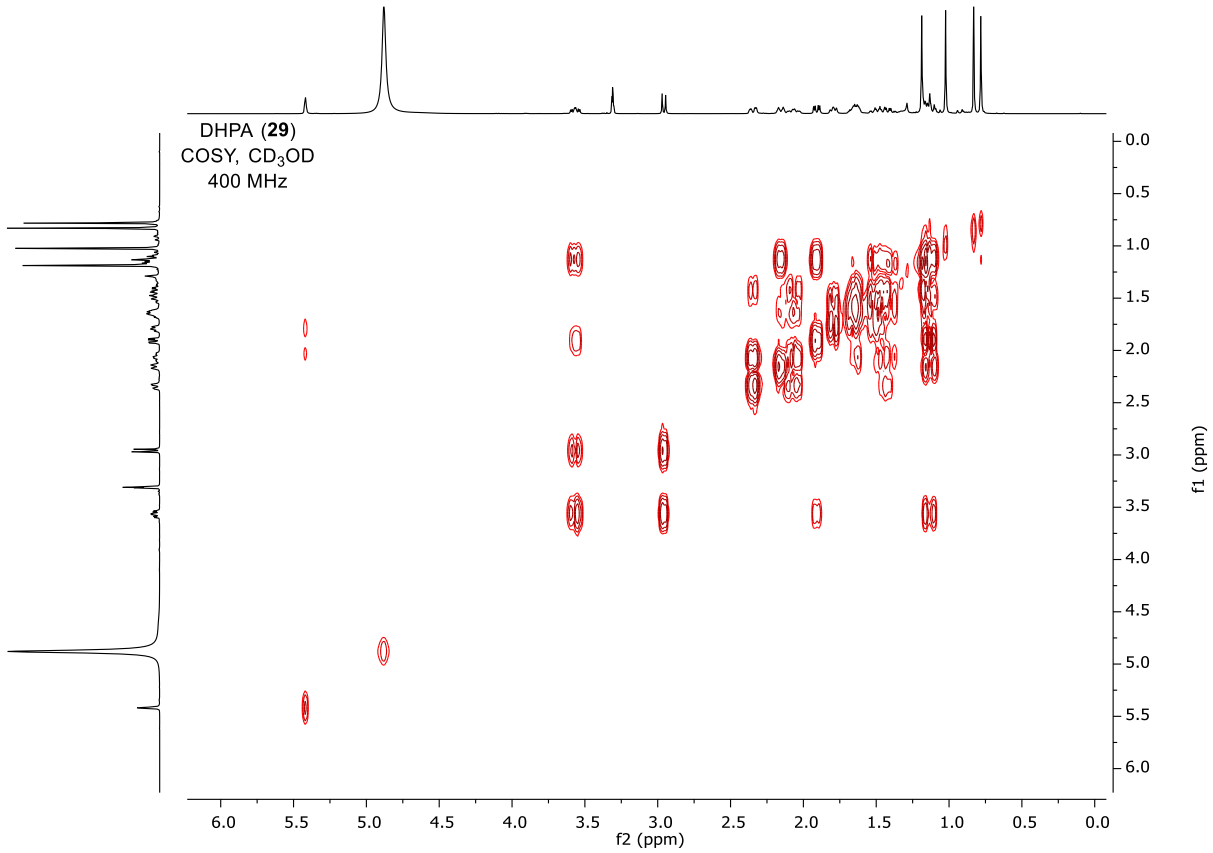

DHPA (**29**)
COSY, CD_3OD
400 MHz
f2 (ppm)
f1 (ppm)

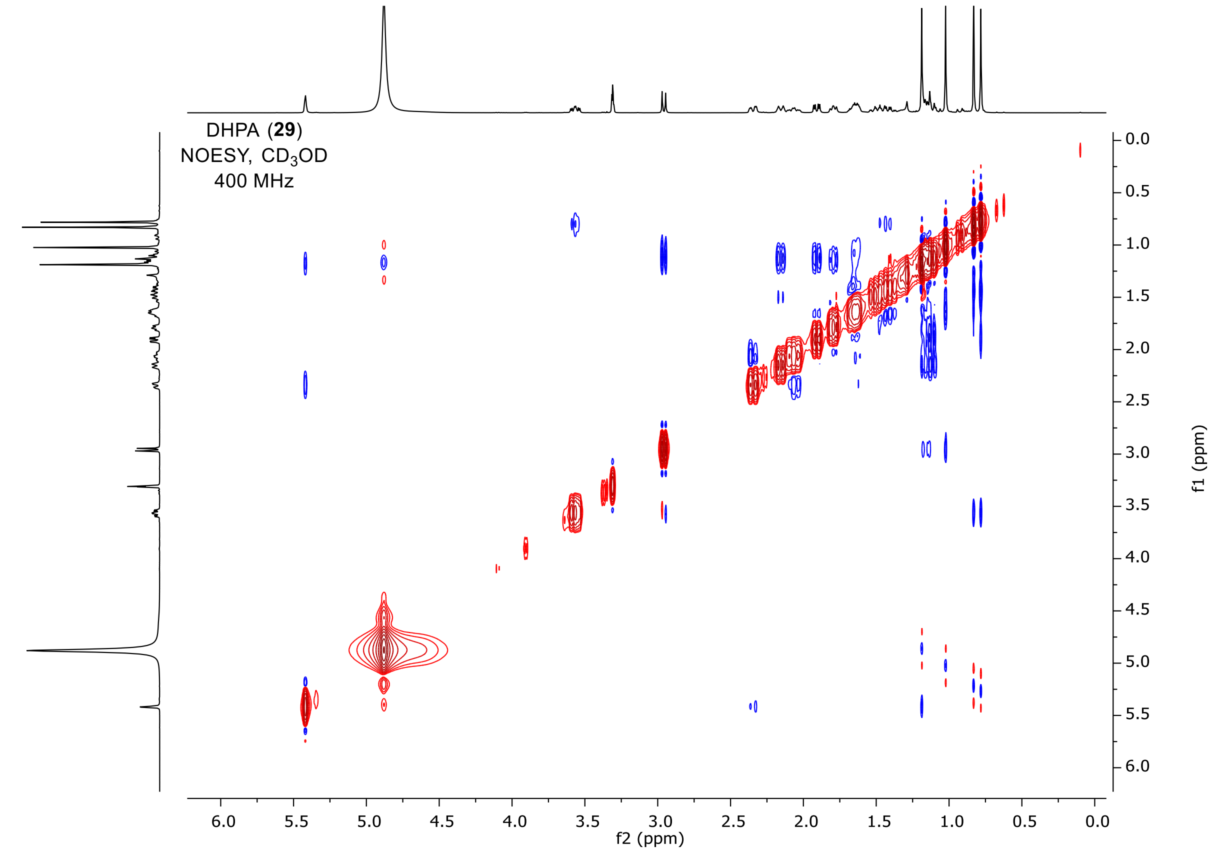

DHPA (**29**)
NOESY, CD_3OD
400 MHz
f2 (ppm)
f1 (ppm)

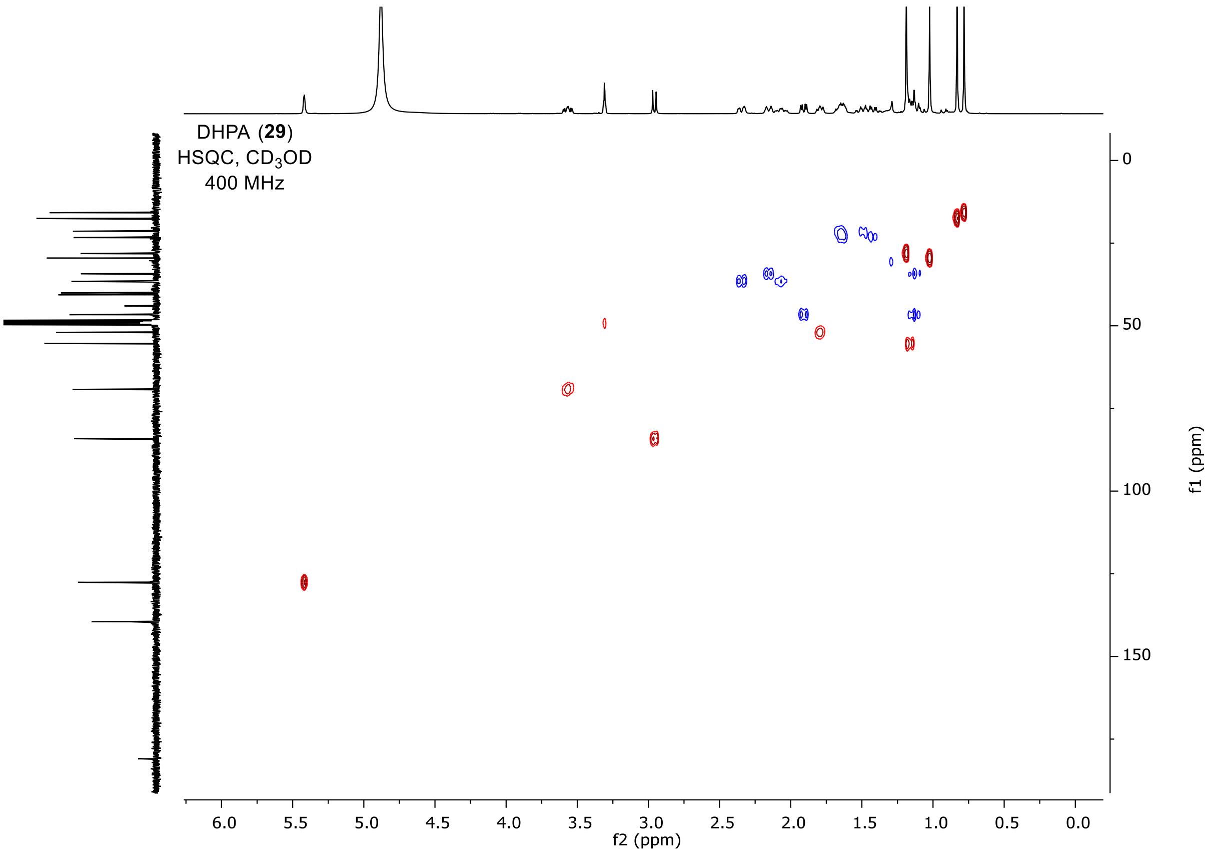
DHPA (**29**)
HSQC, CD_3OD
400 MHz
f1 (ppm)
f2 (ppm)

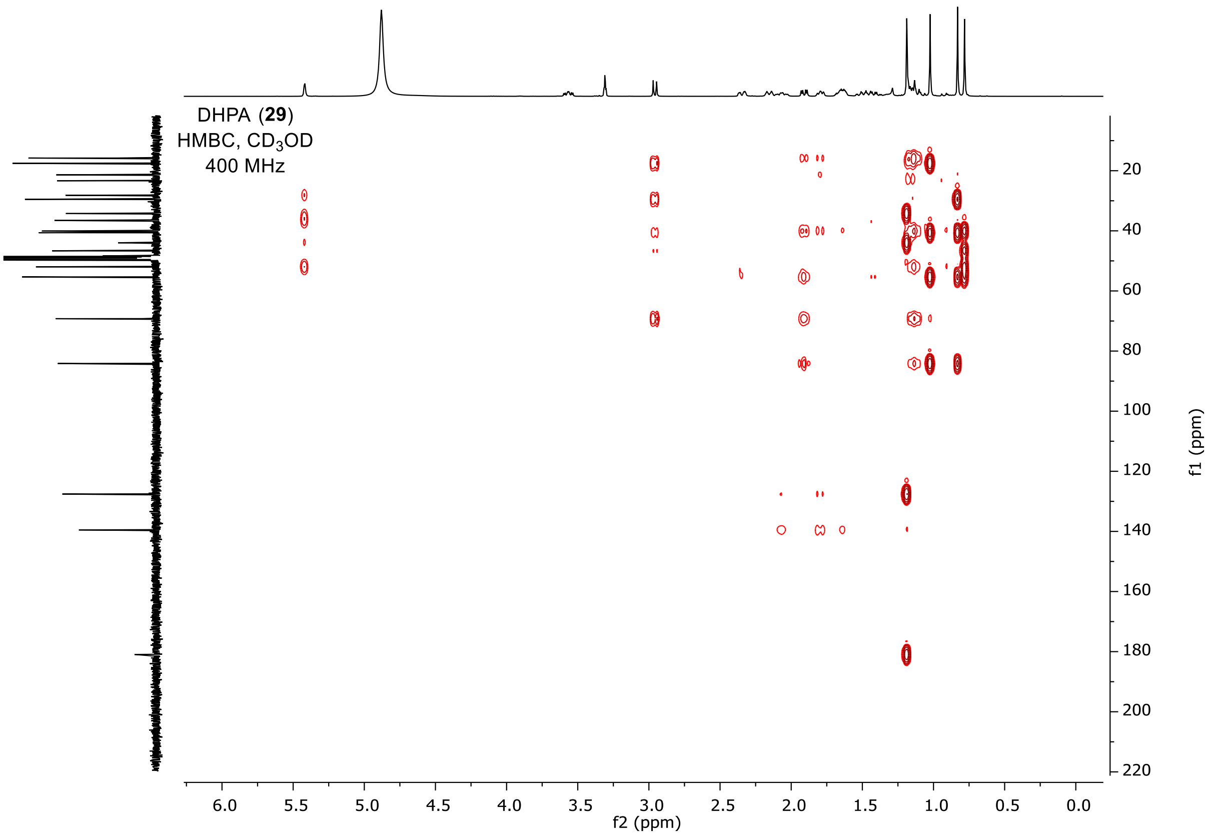
DHPA (**29**)
HMBC, CD_3OD
400 MHz
f1 (ppm)
f2 (ppm)

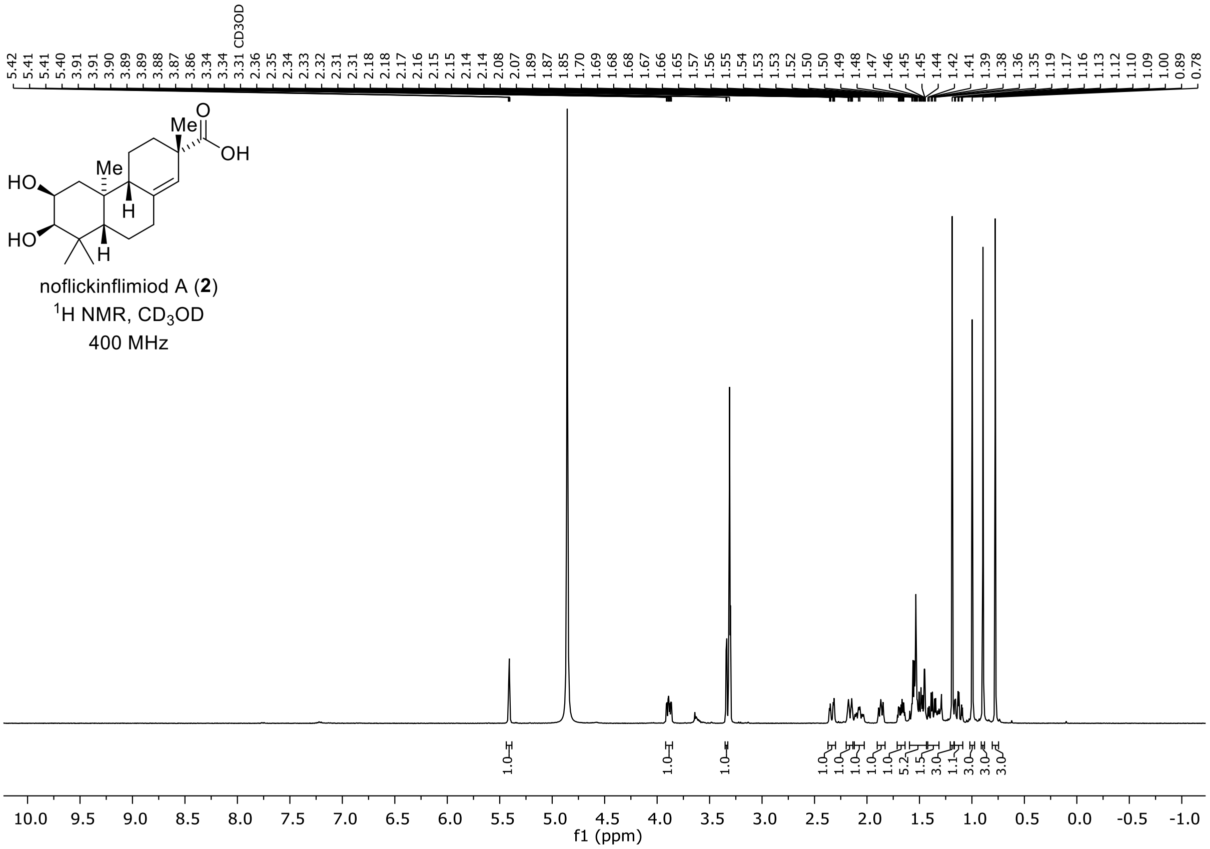
noflickinflimiod A (2)
1H NMR, CD3OD
400 MHz
f1 (ppm)

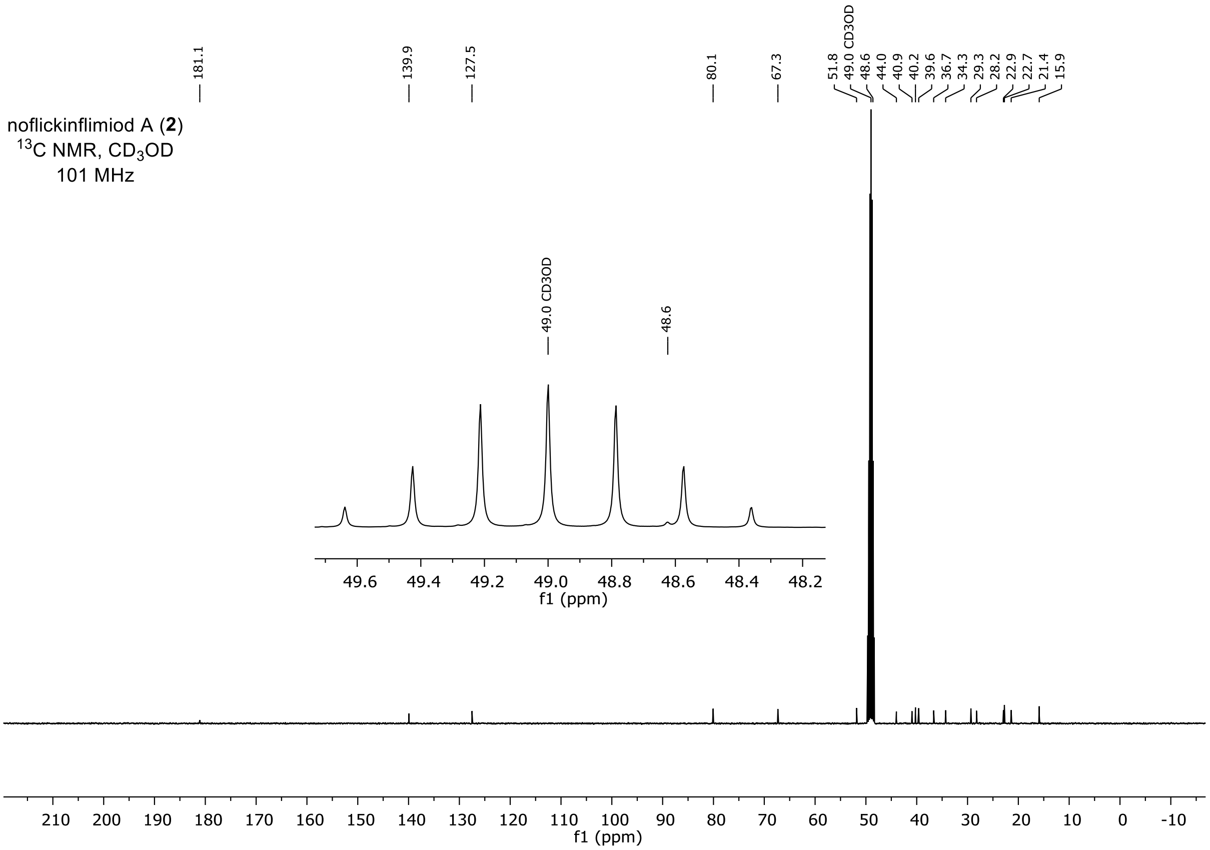
noflickinflimiod A (2)
13C NMR, CD3OD
101 MHz
181.1
139.9
127.5
80.1
67.3
51.8
49.0 CD3OD
48.6
44.0
40.9
40.2
39.6
36.7
34.3
29.3
28.2
22.9
22.7
21.4
15.9
f1 (ppm)

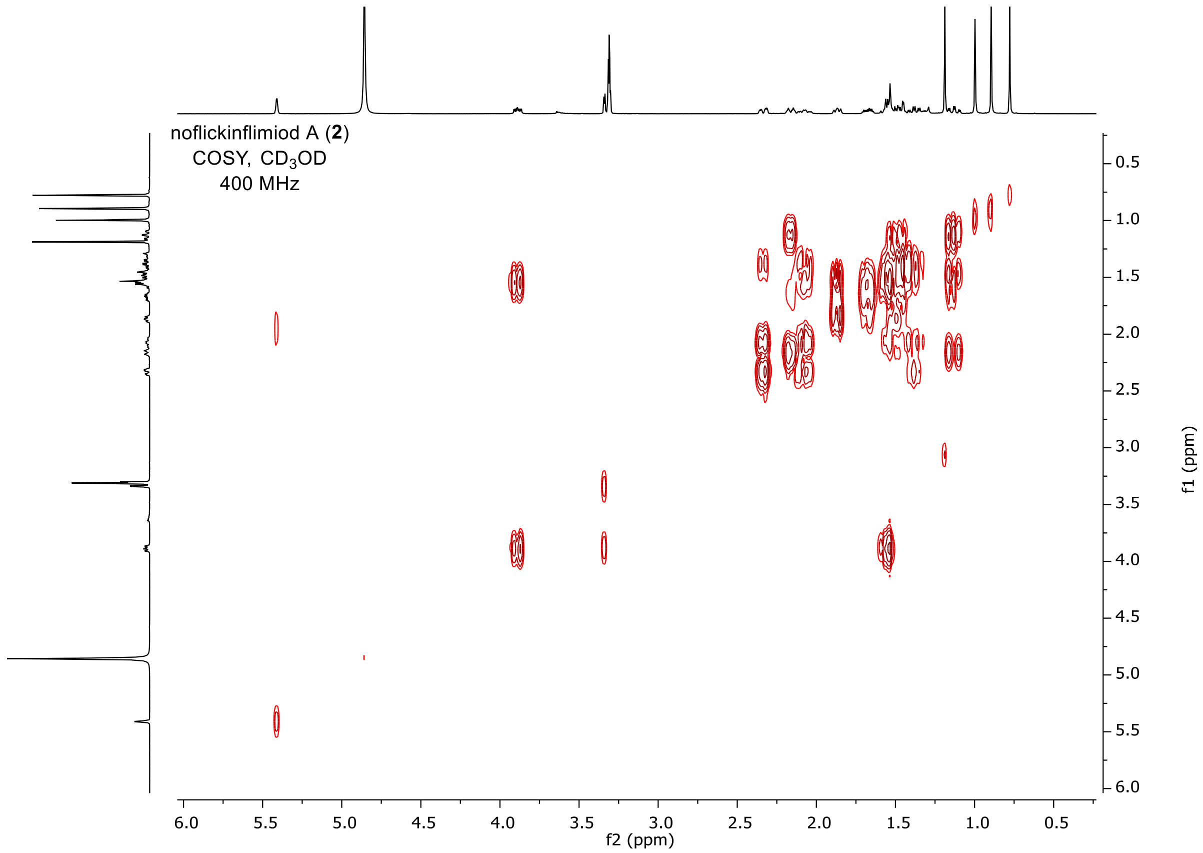
noflickinflimiod A (2)
COSY, CD3OD
400 MHz
f1 (ppm)
f2 (ppm)

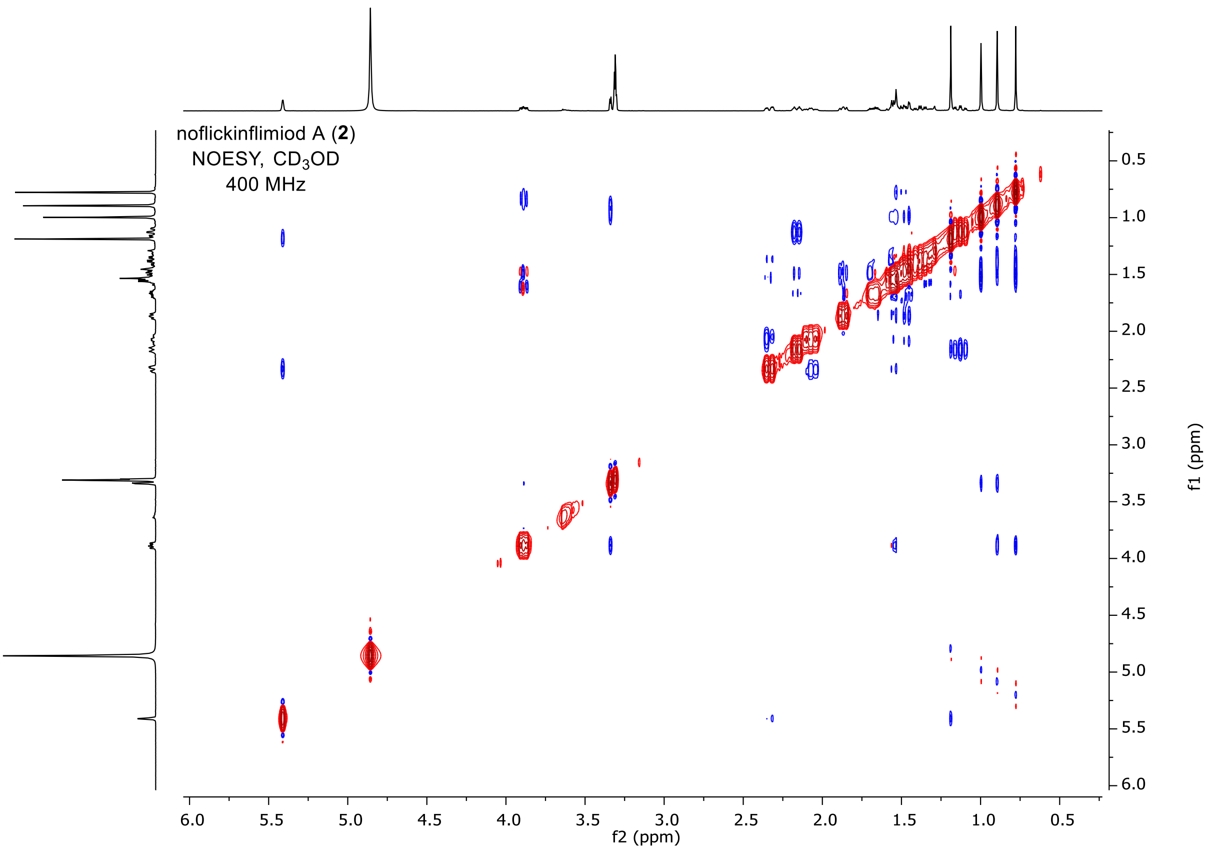
noflickinflimiod A (2)
NOESY, CD3OD
400 MHz
f1 (ppm)
f2 (ppm)

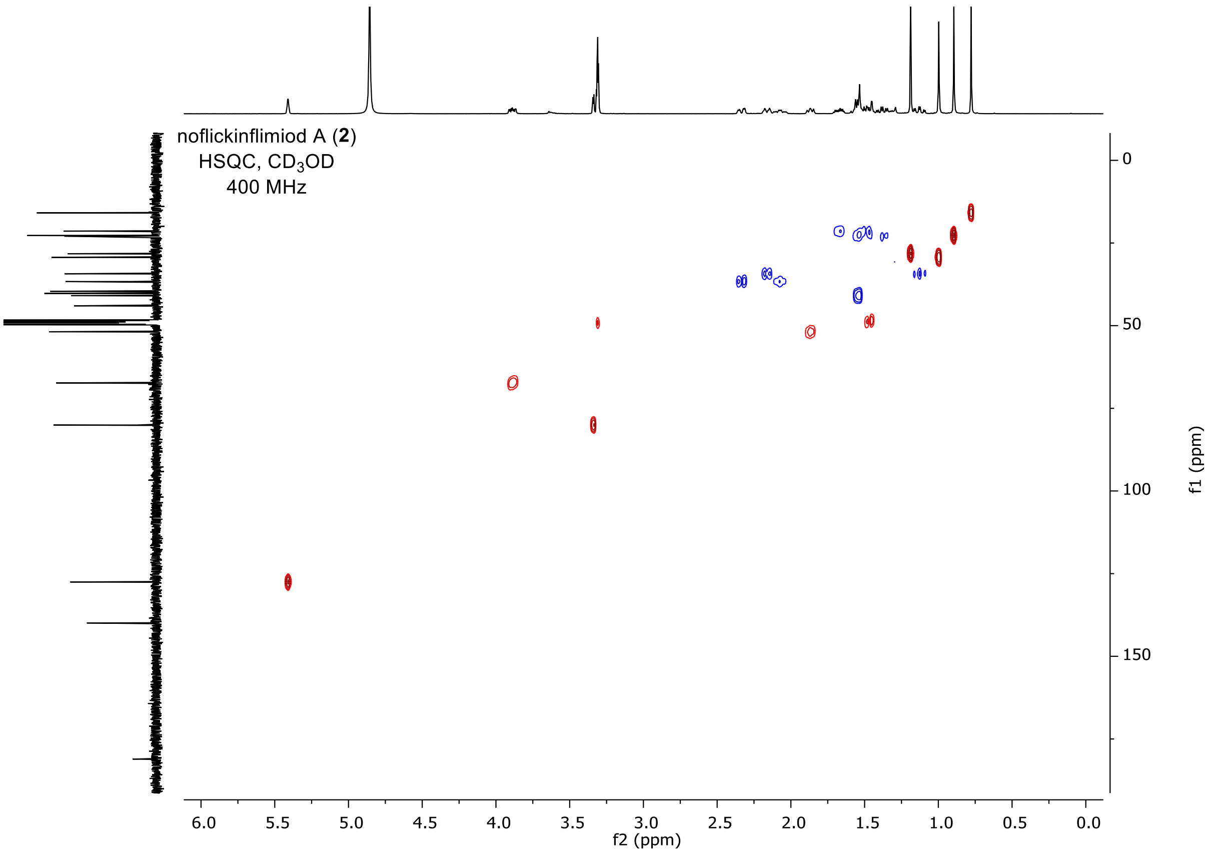
noflickinflimiod A (**2**)
HSQC, CD_3OD
400 MHz
f2 (ppm)
f1 (ppm)

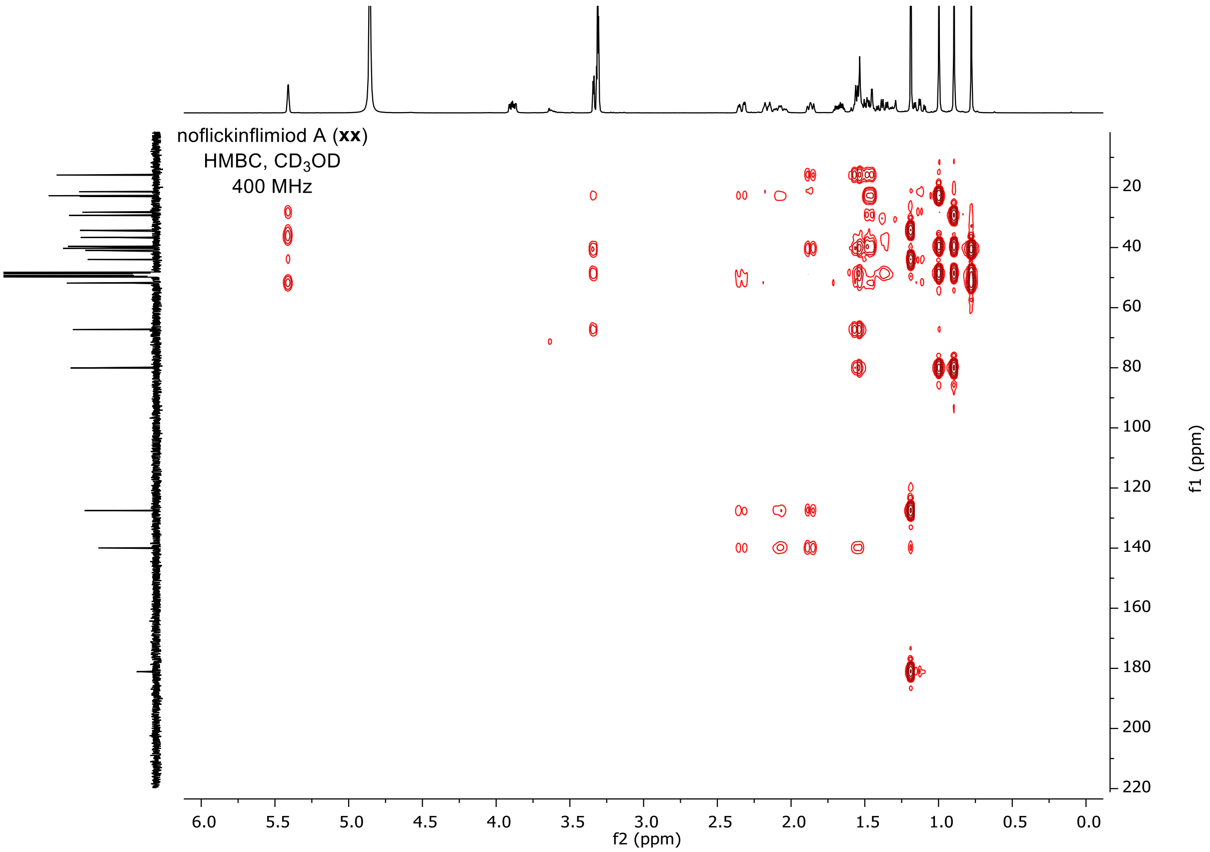
noflickinflimiod A (**xx**)
HMBC, CD_3OD
400 MHz
f2 (ppm)
f1 (ppm)

5. References

(1) Grieco, P. A.; Masaki, Y. General 1,5-Diene Synthesis. Application to the Synthesis of Squalene. *J. Org. Chem.* **1974**, *39*, 2135–2136.

(2) Gosselin, P.; Maignan, C.; Rouessac, F. Stereospecific Synthesis of Homogeranic and Homoneric Acids. *Synthesis* **1984**, 876–881.

(3) Lin, S.-C.; Chein, R.-J. Total Synthesis of the Labdane Diterpenes Galanal A and B from Geraniol. *J. Org. Chem.* **2017**, *82*, 1575–1583.

(4) (a) Zhuo, J.; Zhu, C.; Wu, J.; Li, Z.; Li, C. Reductive Radical Annulation Strategy toward Bicyclo[3.2.1]octanes: Synthesis of *ent*-Kaurane and Beyerane Diterpenoids. *J. Am. Chem. Soc.* **2022**, *144*, 99–105. (b) Zhang, Y.; Ji, Y.; Franzoni, I.; Guo, C.; Jia, H.; Hong, B.; Li, H. Enantioselective Total Synthesis of Berkeleyone A and Preaustinoids. *Angew. Chem., Int. Ed.* **2021**, *60*, 14869–14874. (c) Corey, E. J.; Noe, M. C.; Lin, S. A Mechanistically Designed Bis-cinchona Alkaloid Ligand Allows Position- and Enantioselective Dihydroxylation of Farnesol and Other Oligoprenyl Derivatives at the Terminal Isopropylidene Unit. *Tetrahedron Lett.* **1995**, *36*, 8741–8744.

(5) Crispino, G. A.; Sharpless, K. B. Enantioselective Synthesis of Juvenile Hormone III in Three Steps from Methyl Farnesoate. *Synthesis* **1993**, *8*, 777–779.

(6) Maegawa, T.; Akashi, A.; Yaguchi, K.; Iwasaki, Y.; Shigetsura, M.; Monguchi, Y.; Sajiki, H. Efficient and Practical Arene Hydrogenation by Heterogeneous Catalysts under Mild Conditions. *Chem. - Eur. J.* **2009**, *15*, 6953–6963.

(7) Gilissen, P. J.; Blanco-Ania, D.; Rutjes, F. P. J. T. Oxidation of Secondary Methyl Ethers to Ketones. *J. Org. Chem.* **2017**, *82*, 6671–6679.

(8) Crabtree, S. R.; Chu, W. L. A.; Mander, L. N. C-Acylation of Enolates by Methyl Cyanoformate: An Examination of Site- and Stereoselectivity. *Synlett* **1990**, *1990*, 169–170.

(9) Rubottom, G. M.; Vazquez, M. A.; Pelegrina, D. R. Peracid oxidation of trimethylsilyl enol ethers: A facile α-hydroxylation procedure. *Tetrahedron Lett.* **1974**, *15*, 4319–4322.

(10) Gould, K. J.; Hacker, N. P.; McOmie, J. F. W.; Perry, D. H. Benzocyclobutenes. Part 4. Synthesis of benzocyclobutene-1,2-diones by pyrolytic methods. *J. Chem. Soc., Perkin Trans. 1* **1980**, 1834–1840.

(11) Periyasami, G.; Martelo, L.; Baleizão, C.; Berberan-Santos, M. N. Strong green chemiluminescence from naphthalene analogues of luminol. *New J. Chem.* **2014**, *38*, 2258.

(12) Hill, J.; Ehrlich, J. Nucleophilic Heteroaromatic Substitution. II. Phthalazines. *J. Org. Chem.* **1971**, *36*, 3248–3251.

(13) Choudhury, A. R.; Mukherjee, S. Organocatalytic asymmetric direct vinylogous Michael addition of α,β-unsaturated γ-butyrolactam to nitroolefins. *Org. Biomol. Chem.* **2012**, *10*, 7313–7320.

(14) Jiang, R.; Kuang, Y.; Sun, X.; Zhang, S. An improved catalytic system for recycling OsO_4 and chiral ligands in the asymmetric dihydroxylation of olefins. *Tetrahedron: Asymmetry* **2004**, *15*, 743–746.

(15) Song, L.; Zhu, G.; Liu, Y.; Liu, B.; Qin, S. Total Synthesis of Atisane-Type Diterpenoids: Application of Diels–Alder Cycloadditions of Podocarpane-Type Unmasked *ortho*-Benzoquinones. *J. Am. Chem. Soc.* **2015**, *137*, 13706–13714.

(16) Li, H.; Zhao, J.-J.; Chen, J.-L.; Zhu, L.-P.; Wang, D.-M.; Jiang, L.; Yang, D.-P.; Zhao, Z.-M. Diterpenoids from aerial parts of *Flickingeria fimbriata* and their nuclear factor-kappaB inhibitory activities. *Phytochemistry* **2015**, *117*, 400–409.

(17) Chen, J.-L.; Zhao, Z.-M.; Xue, X.; Tang, G.-H.; Zhu, L.-P.; Yang, D.-P.; Jiang, L. Bioactive norditerpenoids from *Flickingeria fimbriata*. *RSC Adv.* **2014**, *4*, 14447–14456.

(18) (a) Fehr, C.; Galindo, J. Synthesis of (*E*)-1-Propenyl Ketones from Carboxylic Esters and Carboxamides by Use of Mixed Organolithium-Magnesium Reagents. Synthesis of α-Damascone, β-Damascone, and β-Damascenone. *Helv. Chim. Acta* **1986**, *69*, 228–235. (b) Fehr, C.; Galindo, J.; Perret, R. General Synthesis of Ketones from Carboxylic Esters and Carboxamides by Use of Mixed Organolithium-Magnesium Reagents: Syntheses of Artemisia Ketone. *Helv. Chim. Acta* **1987**, *70*, 1745–1752. (c) Taber, D. F.; Frankowski, K. J. Synthesis of (+)-Sulcatine G. *J. Org. Chem.* **2005**, *70*, 6417–6421.

(19) Ma, G. X.; Wang, T. S.; Yin, L.; Pan, Y.; Guo, Y. L.; LeBlanc, G. A.; Reinecke, M. G.; Watson, W. H.; Krawiec, M. Two Pimarane Diterpenoids from *Ephemerantha lonchophylla* and Their Evaluation as Modulators of the Multidrug Resistance Phenotype. *J. Nat. Prod.* **1998**, *61*, 112–115.

(20) Wang, J.; Duan, H.; Wang, Y.; Pan, B.; Gao, C.; Gai, C.; Wu, Q.; Fu, H. *ent*-Strobane and *ent*-Pimarane Diterpenoids from *Siegesbeckia pubescens*. *J. Nat. Prod.* **2017**, *80*, 19–29.

(21) Kim, J. H.; Han, K. D.; Yamasaki, K.; Tanaka, O. Darutoside, a diterpenoid from *Siegesbeckia pubescens* and its structure revision. *Phytochemistry* **1979**, *18*, 894–895.

(22) Barua, R. N.; Sharma, R. P.; Thyagarajan, G.; Herz, W.; Govindan, S. V. New melampolides and darutigenol from *Sigesbeckia orientalis*. *Phytochemistry* **1980**, *19*, 323–325.

(23) Pudles, I., A. Diara, and E. Lederer. The chemical constitution of darutigenol, a tricyclic diterpenetriol. *Bull. Soc. Chim. Fr.* **1959**, 693–700.

(24) Giang, P. M.; Son, P. T.; Otsuka, H. *ent*-Pimarane-Type Diterpenoids from *Siegesbeckia orientalis* L. *Chem. Pharm. Bull.* **2005**, *53*, 232–234.

(25) For selected examples of related cationic bicyclizations see: (a) Castillo, A.; Del Moral, J. F. Q.; Barrero, A. F. Studies in Cyclization of Aromatic Epoxy-acyclicpolyprenes: Lewis Superacids and Titanocene Chloride. *Nat. Prod. Commun.* **2017**, *12*, 657–658. (b) Cherney, E. C.; Green, J. C.; Baran, P. S. Synthesis of *ent*-Kaurane and Beyerane Diterpenoids by Controlled Fragmentations of Overbred Intermediates. *Angew. Chem., Int. Ed.* **2013**, *52*, 9019–9022. (c) Kim, M. B.; Shaw, J. T. Synthesis of Antimicrobial Natural Products Targeting FtsZ: (+)-Totarol and Related Totarane Diterpenes. *Org. Lett.* **2010**, *12*, 3324–3327. (d) Mai, D.; Uchenik, D.; Vanderwal, C. Efforts Toward a Synthesis of Crotogoudin and Crotobarin. *Synlett* **2017**, *28*, 1758–1762. (e) Onyango, E. O.; Fu, L.; Gribble, G. W. Synthesis of a Dicyano Abietane, a Key Intermediate for the Anti-inflammatory Agent TBE-31. *Org. Lett.* **2014**, *16*, 322–324. (f) Rajendar, G.; Corey, E. J. A Systematic Study of Functionalized Oxiranes as Initiating Groups for Cationic Polycyclization Reactions. *J. Am. Chem. Soc.* **2015**, *137*, 5837–5844. (g) Tian, Y.; Xu, X.; Zhang, L.; Qu, J. Tetraphenylphosphonium Tetrafluoroborate/1,1,1,3,3,3-Hexafluoroisopropanol (Ph_4PBF_4/HFIP) Effecting Epoxide-Initiated Cation-Olefin Polycyclizations. *Org. Lett.* **2016**, *18*, 268–271. (h) Wang, Y.-Y.; Gao, Y.-X.; Gao, W.; Xu, Y.; Xu, Y.-Z.; Wang, Y.-J.; Chang, S.; Yu, L.-G.; Zhang, L.-Y.; Liao, H.; Yang, L.-F.; Pang, T.; Qiu, W.-W. Design, synthesis and biological evaluation of tricyclic diterpene derivatives as novel neuroprotective agents against ischemic brain injury. *Eur. J. Med. Chem.* **2015**, *103*, 396–408. (i) Zhao, J.-F.; Zhao, Y.-J.; Loh, T.-P. Indium tribromide-promoted arene-terminated epoxy olefin cyclization. *Chem. Commun.* **2008**, 1353–1355.

(26) van Heerden, F. R.; Dixon, J. T.; Holzapfel, C. W. Direct transformation of steroidal ethers into ketones by dimethyldioxirane. *Tetrahedron Lett.* **1992**, *33*, 7399–7402.

(27) Kamijo, S.; Matsumura, S.; Inoue, M. CCl_3CN: A Crucial Promoter of *m*CPBA-Mediated Direct Ether Oxidation. *Org. Lett.* **2010**, *12*, 4195–4197.

(28) Moriyama, K.; Nakamura, Y.; Togo, H. Oxidative Debenzylation of *N*-Benzyl Amides and *O*-Benzyl Ethers Using Alkali Metal Bromide. *Org. Lett.* **2014**, *16*, 3812–3815.

(29) Dupau, P.; Epple, R.; Thomas, A.; Fokin, V.; Sharpless, K. Osmium-Catalyzed Dihydroxylation of Olefins in Acidic Media: Old Process, New Tricks. *Adv. Synth. Catal.* **2002**, *344*, 421–433.

(30) Woodward, R. B.; Brutcher, F. V. *cis*-Hydroxylation of a Synthetic Steroid Intermediate with Iodine, Silver Acetate and Wet Acetic Acid. *J. Am. Chem. Soc.* **1958**, *80*, 209–211.

(31) Wang, L.; Sharpless, K. B. Catalytic Asymmetric Dihydroxylation of Cis-Disubstituted Olefins. *J. Am. Chem. Soc.* **1992**, *114*, 7568–7570.

(32) (a) Hoye, T. R.; Jeffrey, C. S.; Shao, F. Mosher ester analysis for the determination of absolute configuration of stereogenic (chiral) carbinol carbons. *Nat. Protoc.* **2007**, *2*, 2451–2458. (b) Dale, J. A.; Mosher, H. S. Nuclear Magnetic Resonance Enantiomer Regents. Configurational Correlations via Nuclear Magnetic Resonance Chemical Shifts of Diastereomeric Mandelate, *O*-Methylmandelate, and α-Methoxy-α-Trifluoromethylphenylacetate (MTPA) Esters. *J. Am. Chem. Soc.* **1973**, *95*, 512–519.
